# Supplementary material for: Global Action to reduce HIV stigma and discrimination
Source: J Int AIDS Soc. 2013 Nov 13;16(3Suppl 2):18934. doi: 10.7448/IAS.16.3.18934 (PMC3834870; doi:10.7448/IAS.16.3.18934)
Supplement: Related texts

# Sub article: Global action to reduce HIV stigma and discrimination

**DOI:** 10.7448/IAS.16.3.18881

**Published:** 2013-11-13

**Authors:** Cynthia I Grossman, Anne L Stangl

**Affiliations:** 1 Division of AIDS Research, National Institute of Mental Health, Bethesda, MD, USA; 2 Department of Global Health, International Center for Research on Women, Washington, DC, USA; 3 Secretariat, Stigma Action Network, Washington, DC, USA

**Keywords:** HIV, AIDS, stigma, discrimination, interventions, key populations

## Abstract

There is no question that the stigma and discrimination associated with HIV and AIDS can be reduced through intervention. The inclusion of stigma and discrimination reduction as a critical component of achieving an AIDS-free generation in recent UNAIDS, UN and PEPFAR political initiatives is promising. Yet national governments need evidence on effective interventions at the individual, community and societal levels in order to strategically incorporate stigma and discrimination reduction into national AIDS plans. Currently, the heterogeneity of stigma and discrimination reduction approaches and measurement makes it challenging to compare and contrast evaluated interventions. Moving forward, it is critical for the research community to: (1) clearly link intervention activities to the domains of stigma to be shifted; (2) assess the stigma domains in a consistent manner; and (3) link stigma and discrimination reduction with HIV prevention, care and treatment outcomes (e.g., uptake, adherence and retention of ART). These steps would further advance the scientific evidence base of stigma and discrimination reduction and allow for the identification of effective interventions that could be scaled up by national governments.

## Stigma and discrimination reduction: a critical component of achieving an AIDS-free generation

Given recent advances in biomedical prevention, the global community has begun to seriously contemplate an AIDS-free generation for the first time in three decades. The Joint United Nations Programme on HIV/AIDS (UNAIDS) outlined a vision for “getting to zero” in its strategic plan for 2011–2015, including zero new infections, zero AIDS-related deaths and zero discrimination [1]. National governments are now being encouraged and supported to scale up evidence-based, efficacious HIV prevention and treatment technologies [2] in order to achieve the ambitious goals agreed to by UN member states at the 2011 UN high-level meeting on HIV/AIDS [3]. The President's Emergency Plan For AIDS Relief (PEPFAR) Blueprint, which proposes critical steps needed to achieve an AIDS-free generation, was launched in 2012 [4]. A common element of these political initiatives is their recognition that reducing HIV-related stigma and discrimination is critical to the success of the global HIV response.

The field of stigma research related to HIV has advanced rapidly, and while many questions remain unanswered and gaps in empirically derived data exist, there is no question that stigma can be reduced [5, 6]. Specific research strides include a solid evidence base of valid measures that capture multiple domains of stigma associated with HIV [7, 8], including one measure showcased in this special issue. Although there remains a need for empirically derived data, stigma and discrimination interventions have been developed and implemented in the field [9, 10], as well as a comprehensive toolkit of stigma reduction activities [11], components of which are being tested as part of National Institutes of Health-supported research [12]. The healthcare sector has one of the strongest evidence bases regarding stigma and discrimination measurement and intervention [13–15]. Beyond the healthcare setting, addressing stigma among the general community has been a focus for research, but to varying degrees of success [6]. Much of the work has included community education campaigns associated with HIV testing, including some community mobilization strategies. With regard to stigma measurement among people living with HIV (PLHIV), several measures have been developed [16–18], with the PLHIV Stigma Index serving as both an assessment and a community engagement and empowerment tool [19]. Despite these strides, the absence of evidence demonstrating a clear link between stigma and discrimination reduction and HIV outcomes, as well as the cost-effectiveness of these approaches, is impeding their prioritization by implementing organizations, ministries of health and other entities.

## The price of inaction: the impact of stigma and discrimination on HIV prevention and care programmes

The persistence of HIV-related stigma and discrimination is evident in research and programmatic data alike, despite treatment advances that have turned HIV into a chronic, manageable condition. Thirty years into the HIV pandemic, stigma and discrimination continue to impede individuals and communities from accessing and benefiting from effective prevention and treatment strategies. There is mounting evidence that HIV-related stigma and discrimination are barriers to HIV testing [20], sero-status disclosure [21], retention in care [22] and uptake of and adherence to antiretroviral therapy (ART) [23, 24]. There is also evidence of the associations between HIV-related stigma and racism, poverty and heterosexism, although the complexities of these associations and interactions are only beginning to be unravelled via research [25–27]. In many settings, the stigma associated with HIV is fuelled by laws and policies that keep key populations at risk of HIV infection and PLHIV at the margins of society, despite evidence of the negative public health impact of criminalization [28, 29].

Included among these discriminatory laws are those that make illegal lesbian, gay and bisexual relationships; expressing one's transgender identity; drug use; and sex work, and those that create barriers to legal protections for these groups and for young people, women and migrants. At least 47 countries have used the criminal law to prosecute PLHIV for non-disclosure of their HIV status, potential exposure of others to HIV, or transmission of HIV, regardless of whether there was any intent to transmit, harm reduction measures were adopted, the person with HIV risked violence if she or he disclosed, or transmission actually occurred [30]. Many of these laws provide for criminal prosecution of PLHIV for behaviours that bear little to no risk of transmission, such as for spitting or biting [31]. Yet, as highlighted in the review by Stangl et al. [32] in this special issue, no interventions to reduce HIV-related discrimination have been assessed in the peer-reviewed or grey literature, and very few intervention tools exist for reducing intersecting HIV and key population stigmas [33, 34].

The fact that stigma associated with HIV continues to hamper prevention and treatment efforts is particularly distressing given the unprecedented number of effective tools available, including recent advances such as voluntary medical male circumcision, pre-exposure prophylaxis and ART for the purposes of extending the lives of PLHIV and providing HIV prevention benefits for their sexual partners in the context of viral suppression [5–37]. In addition, many countries are rolling out Option B+, which provides ART to expectant mothers living with HIV for their lifetime, regardless of CD4 cell count, as opposed to a shorter course around pregnancy, childbirth and breastfeeding [38]. These tools are in various stages of being brought to scale, but as the data on the role of HIV stigma as a barrier to testing, retention in care and treatment suggest, these tools may not reach their full potential if the stigma and discrimination associated with HIV remain unaddressed.

For example, in 2011 UNAIDS estimated that 46% of people eligible for ART in low- and middle-income countries did not receive it [39]. In countries where all pregnant women presenting for prenatal care are tested for HIV and provided treatment access, there is still an alarmingly high rate of women who refuse to present for prenatal care due to the stigma associated with HIV, despite access to effective treatment [20, 40]. As certain populations fail to access, or in some cases are excluded from accessing, resources, such as quality healthcare, food, housing and employment opportunities, based on factors other than HIV status (e.g., race or sexual orientation), the stigma associated with HIV acts in a compounding fashion to further exacerbate disparities [8, 41, 42].

Despite the advances in HIV stigma research over the last decade that are mentioned here, the gap in the evidence base on effective interventions is hampering national governments from integrating stigma and discrimination reduction – critical enablers of the HIV response – into national AIDS plans, and is threatening our collective ability to get to zero. As national governments seek to bring HIV prevention and treatment to a larger scale amidst multiple resource constraints, both human and financial, there needs to be strong evidence for the impact of stigma reduction efforts and high-quality data that can inform evidence-based decision making around priorities at the national level. To respond to this gap in the evidence base, a redoubling of research efforts to reduce stigma and discrimination across a variety of settings and within all populations is needed. The goal of this special issue is to enhance the peer-reviewed literature with strong, scientifically sound evidence for stigma reduction interventions. The intention is also to encourage the research field to consider additional ways to reduce stigma and discrimination, acknowledge and address the challenges with research methodology and create a sense of timeliness and urgency for the development and testing of stigma and discrimination reduction efforts.

## A common conceptualization of HIV-related stigma and discrimination: critical for generating evidence

Currently, the heterogeneity of stigma and discrimination reduction approaches and measurement makes it challenging to compare and contrast evaluated interventions, as evidenced by the lack of meta-analyses of stigma reduction interventions in the literature. While there is general agreement around four intervention categories originally described by Brown *et al*. [5] (i.e., information-based approaches, skills building, counselling and support, and contact with affected groups), there is less agreement about how to measure the success of these approaches at influencing the various domains of HIV stigma. This stems from the lack of a common conceptualization of the stigmatization process that can inform research, programmatic and evaluation efforts.

The foundation of HIV stigma research is Erving Goffman's seminal conceptualization of stigma as a discrediting attribute that creates a “spoiled identity,” which cuts the stigmatized person “off from society and from himself” [43]. More recent conceptualizations have highlighted the societal and structural nature of stigma, and have attempted to articulate the process of stigmatization [44, 45] and distinguish stigma from discrimination [46]. These conceptualizations have framed current understanding regarding the need to intervene at multiple socio-ecological levels (i.e. individual, interpersonal, organizational, community and public policy) to reduce HIV-related stigma and discrimination [47]. A recent global effort to develop standardized indicators of HIV stigma and discrimination led to the development of a practical framework to inform stigma reduction programming and measurement [48]. This framework defines specific domains, including drivers, facilitators, intersecting stigmas and manifestations of stigma, that can be shifted through programmatic efforts, and it proposes measures to assess each domain [49]. Moving forward, it is critical for the research community to (1) clearly link intervention activities to the domains of stigma to be shifted; (2) assess the stigma domains in a consistent manner; and (3) link stigma and discrimination reduction with HIV prevention, care and treatment outcomes (e.g., uptake, adherence and retention of ART). These steps would further advance the scientific evidence base of stigma and discrimination reduction and allow for the identification of effective interventions that could be scaled up by national governments.

## Global action to reduce stigma and discrimination

Given the importance of reducing HIV-related stigma and discrimination for realizing an AIDS-free generation, this special issue takes stock of current strategies for interrupting the stigmatization process, reducing the negative manifestations of stigma and discrimination, and creating an enabling environment for HIV prevention, care and treatment strategies. The articles in this issue review and organize current evidence on approaches for reducing stigma and discrimination in the healthcare setting, among the general public and among PLHIV and key populations. They draw attention to methodological and measurement challenges in evaluating stigma and discrimination reduction interventions, highlight innovative approaches for addressing stigma in a variety of populations and contexts, and identify critical gaps in these approaches that must be addressed in future research. They also provide insights into the determinants of key population stigmas to inform future intervention development to address intersecting stigmas.

In addition to academic peer reviews, all of the manuscripts benefitted from careful review by individuals directly impacted by stigma and discrimination, via a panel of reviewers from communities of PLHIV and members of key population groups. These reviews were critical to ensuring attention to terminology and the relevance of findings to programmatic efforts, and for clarifying ways in which the research would benefit individuals and communities while simultaneously advancing the science.

Several key issues are highlighted in this supplement. First, the review articles reinforce the need for effective stigma and discrimination reduction interventions that can be taken to a national-level scale, and they identify key gaps in current HIV stigma research and methodology that require intensified efforts. The review by Katz et al. [50] synthesizes the evidence for the link between stigma and adherence across a number of studies. As adherence is critical for PLHIV to achieve viral suppression and benefit fully from the individual and prevention benefits of ART, the link between stigma and adherence provides a sobering picture of the work left to do to achieve the full benefits of universal access to ART. The review by Stangl et al. [32] synthesizes evaluation data from nearly 50 interventions, documenting the considerable progress made over the past decade and identifying key gaps and impediments to the identification of effective stigma reduction strategies, including the heterogeneity of measures used to assess stigma domains, the paucity of interventions designed to address multiple sociological levels concurrently and the lack of studies comparing the effectiveness of different stigma reduction strategies and studies assessing the influence of stigma reduction on key behavioural and biomedical outcomes.

Second, at the same time that they are negatively impacted by stigma and discrimination, PLHIV are critical for the success of stigma reduction interventions. In particular, group-based approaches led by or actively involving PLHIV hold promise for responding to HIV-related stigma and discrimination at the community level. For example, an intervention in Uganda found that groups of PLHIV working collectively to reduce stigma and discrimination in their communities bolstered confidence among members, reduced self-stigma and improved group members’ ability to deal with external HIV stigma when encountered [51]. Likewise, an intervention in Thailand that paired business partners living with HIV with those who were HIV negative, and trained them to engage their communities in stigma reduction activities, appears to have led to community-level reductions in fear of HIV infection and shame associated with HIV [52].

Third, this supplement reflects the substantial progress that has been made towards reducing stigma in healthcare settings. Efficacious health facility-based [53] and medical school-based interventions [54] now exist to reduce stigma and discrimination towards PLHIV, and a standardized tool for assessing HIV-related stigma in health facilities [55] has been developed. In addition, this special issue contains the first ever evaluation of a discrimination reduction intervention, which integrated legal literacy and legal services into health facilities in Kenya. Findings suggest that legal empowerment programmes have the potential to improve access to justice and health among marginalized groups (including PLHIV), promote accountability among healthcare providers and contribute to altering unjust structures and systems [56]. These advances are timely, given the need to take effective strategies to scale, as evidenced by PLHIV Stigma Index data, which highlight how commonplace it is globally to experience stigma in healthcare settings [19, 57], and an article in this special issue that found high levels of stigma in urban health facilities in India. For example, providers expressed a willingness to prohibit women living with HIV from having children (55–80%), endorsed mandatory testing for female sex workers (94–97%) and surgery patients (90–99%) and stated that people who acquired HIV through sex or drugs “got what they deserved” (50–83%) [58].

Finally, this special issue presents new evidence to inform the development of interventions to reduce stigma towards key populations, specifically men who have sex with men, people who inject drugs and African Americans. This set of manuscripts highlights the need to integrate stigma reduction with HIV prevention messages and activities and the importance of investigating the impacts of the larger socio-political and economic contexts on stigma and healthcare utilization. Two articles have attempted to expand the reach of stigma reduction and HIV prevention for key populations in the United States, one via African American churches and one via the internet. Berkley-Patton et al. [59] piloted an intervention to increase HIV awareness and testing among members of African American churches. While attitudes and willingness to receive an HIV test improved over the course of the intervention, stigma remained unchanged. The reasons for the lack of change in stigma and the implications for HIV prevention uptake remain worthy of further investigation. Christensen et al. [60] found that a journal-based game was successful at reducing shame and sexual risk-taking behaviour among young men who have sex with men in the United States. Such technology-based interventions have the potential to greatly expand the reach of both stigma reduction and HIV prevention messages to young men who have sex with men in contexts with widespread access to the internet.

In contexts where same-sex behaviour is criminalized, different types of interventions may be needed to address both the stigma experienced by men who have sex with men and the discriminatory laws and policies that fuel the stigmatization process. Risher et al. [61] found high levels of stigma among men who have sex with men in Swaziland (i.e., 61.7% feared seeking healthcare, 44.1% experienced some form of stigma and 73.9% perceived social stigma from family and friends) and identified a number of factors associated with non-disclosure of sexual behaviour to healthcare providers and fear of seeking healthcare, including having experienced legal discrimination as a result of one's sexual orientation or practices. The analysis provides several insights for developing structural interventions to increase healthcare seeking and disclosure of sexual practices to healthcare workers and facilitate behavioural and biomedical HIV prevention approaches among men who have sex with men in Swaziland.

Also relevant to developing structural interventions, the article by Lim et al. [62] demonstrates the importance of considering education and income inequality in designing interventions to reduce stigma towards people who inject drugs in Viet Nam. The analysis revealed that individual-level educational attainment was significantly associated with less stigmatizing attitudes, and this relationship superseded community-level inequalities in education and income.

Research, policy and programmes that seek to address the HIV epidemic are in unprecedented alignment in their call to scale up the tools to bring about an AIDS-free generation. As evidenced by the foreword to this special issue, key agencies are also in alignment regarding the importance of stigma and discrimination reduction and its role in facilitating scale-up and uptake of HIV prevention, care and treatment. This supplement is as important for the advances that it highlights as well as the gaps it identifies. As the evidence base grows, so too will the ability of national governments to make data-driven decisions about scaling up stigma and discrimination reduction efforts. It is incumbent on the research community to provide data that will help governments make efficient and effective use of resources spent on stigma and discrimination reduction. That said, it is important to recognize that all programmatic efforts take resources, evidence and political will. This supplement is the start of a discussion regarding the evidence for stigma and discrimination reduction efforts. It is also a call to action for even more refined research activities, for greater community involvement (particularly of key populations in research and programmatic efforts) and for scale-up of some programmatic principles that have been identified, while including high-quality monitoring and evaluation strategies to further expand the evidence base. It is our hope that within the next decade, cost-effective interventions will be identified and countries will be collecting programmatic data demonstrating the impact of stigma and discrimination reduction on HIV prevention and care outcomes.

## References

[1] UNAIDS (2010). Getting to zero: 2011–2015 strategy. Joint United Nations Programme on HIV/AIDS.

[2] Schwartlander B, Stover J, Hallett T, Atun R, Avila C, Gouws E (2011). Towards an improved investment approach for an effective response to HIV/AIDS. Lancet.

[3] United Nations (2011). Political declaration on HIV/AIDS: intensifying our efforts to eliminate HIV/AIDS.

[4] PEPFAR (2012). PEPFAR blueprint: creating an AIDS-free generation.

[5] Brown L, Macintyre K, Trujillo L (2003). Interventions to reduce HIV/AIDS stigma: what have we learned?. AIDS Educ Prev.

[6] Sengupta S, Banks B, Jonas D, Miles MS, Smith GC (2011). HIV interventions to reduce HIV/AIDS stigma: a systematic review. AIDS Behav.

[7] Earnshaw VA, Chaudoir SR (2009). From conceptualizing to measuring HIV stigma: a review of HIV stigma mechanism measures. AIDS Behav.

[8] Nyblade L (2006). Measuring HIV stigma: existing knowledge and gaps. Psychol Health Med.

[9] Pulerwitz J, Michaelis A, Weiss E, Brown L, Mahendra V (2010). Reducing HIV-related stigma: lessons learned from horizons research and programs. Public Health Rep.

[10] Nyblade L, Hong KT, Anh NV, Ogden J, Jain A, Stangl A (2008). Communities confront HIV stigma in Viet Nam: participatory interventions reduce HIV-related stigma in two provinces.

[11] Kidd R, Clay S, Chiiya C (2007). Understanding and challenging HIV stigma: toolkit for action.

[12] Rao D, Desmond M, Andrasik M, Rasberry T, Lambert N, Cohn SE (2012). Feasibility, acceptability, and preliminary efficacy of the unity workshop: an internalized stigma reduction intervention for African American women living with HIV. AIDS Patient Care STDS.

[13] Nyblade L, Stangl A, Weiss E, Ashburn K (2009). Combating HIV stigma in health care settings: what works?. J Int AIDS Soc.

[14] Uys L, Chirwa M, Kohi T, Greeff M, Naidoo J, Makoae L (2009). Evaluation of a health setting-based stigma intervention in five African countries. AIDS Patient Care STDS.

[15] Li L, Wu Z, Liang LJ, Lin C, Guan J, Jia M (2013). Reducing HIV-related stigma in health care settings: a randomized controlled trial in China. Am J Public Health.

[16] Berger BE, Ferrans CE, Lashley FR (2001). Measuring stigma in people with HIV: psychometric assessment of the HIV stigma scale. Res Nurs Health.

[17] Kalichman SC, Simbayi LC, Cloete A, Mthembu P, Mkhonta RN, Ginindza T (2009). Measuring AIDS stigmas in people living with HIV/AIDS: the internalized AIDS-related stigma scale. AIDS Care.

[18] Holzemer WL, Uys LR, Chirwa ML, Greeff M, Makoae LN, Kohi TW (2007). Validation of the HIV/AIDS stigma instrument – PLWA (HASI-P). AIDS Care.

[19] Zamudio AR, Keovongchith B, Boisson D, Crepey P, Bagshaw K, Phongdeth K (2013). Results of the people living with HIV stigma index in Lao PDR: documenting the HIV stigma and dscirmination situtation in the country and disentangling the layers of stigma in marginlized populations.

[20] Turan JM, Bukusi EA, Onono M, Holzemer WL, Miller S, Cohen CR (2011). HIV/AIDS stigma and refusal of HIV testing among pregnant women in rural Kenya: results from the MAMAS study. AIDS Behav.

[21] 
Tsai AC, Bangsberg DR, Kegeles SM, Katz IT, Haberer JE, Muzoora C (2013). Internalized stigma, social distance, and disclosure of HIV seropositivity in rural Uganda. Ann Behav Med.

[22] Naar-King S, Bradford J, Coleman S, Green-Jones M, Cabral H, Tobias C (2007). Retention in care of persons newly diagnosed with HIV: outcomes of the outreach initiative. AIDS Patient Care STDS.

[23] Rintamaki LS, Davis TC, Skripkauskas S, Bennett CL, Wolf MS (2006). Social stigma concerns and HIV medication adherence. AIDS Patient Care STDS.

[24] Rao D, Kekwaletswe TC, Hosek S, Martinez J, Rodriguez F (2007). Stigma and social barriers to medication adherence with urban youth living with HIV. AIDS Care.

[25] Earnshaw VA, Bogart LM, Dovidio JF, Williams DR (2013). Stigma and racial/ethnic HIV disparities: moving toward resilience. Am Psychol.

[26] Logie C, James L, Tharao W, Loutfy M (2013). Associations between HIV-related stigma, racial discrimination, gender discrimination, and depression among HIV-positive African, Caribbean, and Black women in Ontario, Canada. AIDS Patient Care STDS.

[27] Bogart LM, Wagner GJ, Galvan FH, Klein DJ (2010). Longitudinal relationships between antiretroviral treatment adherence and discrimination due to HIV-serostatus, race, and sexual orientation among African-American men with HIV. Ann Behav Med.

[28] Burris S, Cameron E (2008). The case against criminalization of HIV transmission. JAMA.

[29] Mykhalovskiy E (2011). The problem of “significant risk”: exploring the public health impact of criminalizing HIV non-disclosure. Soc Sci Med.

[30] GNP + (2010). The global criminalisation scan report.

[31] Weait M (2011). The criminalisation of HIV transmission and exposure: a global review.

[32] Stangl AL, Lloyd JK, Brady LM, Holland CE, Baral CE (2013). A systematic review of interventions to reduce HIV-related stigma and discrimination from 2002 to 2013: how far have we come?. J Int AIDS Soc.

[33] Sakara P, Eng S, Barmey P, Reeves M, Nyblade L, Gregowski A (2010). Understanding and challenging stigma toward men who have sex with men: Cambodia edition toolkit for action.

[34] Khuat TH, Nguyen TVA, Khuat Thi O, Kidd RS, Brady L, Nyblade L (2011). Understanding and challenging stigma toward injecting drug users and HIV in Vietnam: toolkit for action.

[35] Dai JY, Gilbert PB, Hughes JP, Brown ER (2013). Estimating the efficacy of preexposure prophylaxis for HIV prevention among participants with a threshold level of drug concentration. Am J Epidemiol.

[36] Grant RM, Lama JR, Anderson PL, McMahan V, Liu AY, Vargas L (2010). Preexposure chemoprophylaxis for HIV prevention in men who have sex with men. N Engl J Med.

[37] Tanser F, Barnighausen T, Grapsa E, Zaidi J, Newell ML (2013). High coverage of ART associated with decline in risk of HIV acquisition in rural KwaZulu-Natal, South Africa. Science.

[38] Thyssen A, Lange JH, Thyssen E, Reddi A (2013). Toward an AIDS-free generation with option B+: reconceptualizing and integrating prevention of mother to child transmission (PMTCT) with pediatric antiretroviral therapy initiatives. J Acquir Immune Defic Syndr.

[39] UNAIDS (2012). Global report: UNAIDS report on the global AIDS epidemic.

[40] Turan JM, Miller S, Bukusi EA, Sande J, Cohen CR (2008). HIV/AIDS and maternity care in Kenya: how fears of stigma and discrimination affect uptake and provision of labor and delivery services. AIDS Care.

[41] Chan KY, Yang Y, Zhang KL, Reidpath DD (2007). Disentangling the stigma of HIV/AIDS from the stigmas of drugs use, commercial sex and commercial blood donation – a factorial survey of medical students in China. BMC Public Health.

[42] Loutfy MR, Logie CH, Zhang Y, Blitz SL, Margolese SL, Tharao WE (2012). Gender and ethnicity differences in HIV-related stigma experienced by people living with HIV in Ontario, Canada. PLoS One.

[43] Goffman E (1963). Stigma: notes on the management of spoiled identity.

[44] Link BG, Phelan JC (2001). Conceptualizing stigma. Ann Rev Sociol.

[45] Parker R, Aggleton P (2003). HIV and AIDS-related stigma and discrimination: a conceptual framework and implications for action. Soc Sci Med.

[46] Deacon H (2006). Towards a sustainable theory of health-related stigma: lessons from the HIV/AIDS literature. J Community Appl Soc Psychol.

[47] Heijnders M, van der Meij S (2006). The fight against stigma: an overview of stigma-reduction strategies and interventions. Psychol Health Med.

[48] Stangl A, Go V, Zelaya C, Brady L, Nyblade L, Stackpool-Moore L (2010). Enabling the scale-up of efforts to reduce HIV stigma and discrimination: a new framework to inform program implementation and measurement.

[49] Stangl A, Brady B, Fritz K (2012). Technical brief: measuring HIV stigma and discrimination.

[50] Katz IT, Ryu AE, Onuegbu AG, Psaros C, Weiser SD, Bangsberg DR (2013). Impact of HIV-related stigma on treatment adherence: systematic review and meta-synthesis. J Int AIDS Soc.

[51] Mburu G, Ram M, Skovdal M, Bitira D, Hodgson I, Mwai G (2013). Resisting and challenging stigma in Uganda: the role of support groups of people living with HIV. J Int AIDS Soc.

[52] Jain A, Nuankaew R, Mongkholwiboolphol N, Banpabuth A, Tuvinun R, Oranop na Ayuthaya P (2013). Community-based interventions that work to reduce HIV stigma and discrimination: results of an evaluation study in Thailand. J Int AIDS Soc.

[53] Li L, Lin C, Guan J, Wu Z (2013). Implementing a stigma reduction intervention in healthcare settings. J Int AIDS Soc.

[54] Varas-Díaz N, Neilands TB, Cintrón-Bou F, Marzán-Rodríguez M, Santos-Figueroa A, Santiago-Negrón S (2013). Testing the efficacy of an HIV stigma reduction intervention with medical students in Puerto Rico: the SPACES project. J Int AIDS Soc.

[55] Nyblade L, Jain A, Benkirane M, Li L, Lohiniva A-L, McLean R (2013). A brief, standardized tool for measuring HIV-related stigma among health facility staff: results of field testing in China, Dominica, Egypt, Kenya, Puerto Rico and St. Christopher & Nevis. J Int AIDS Soc.

[56] Gruskin S, Safreed-Harmon K, Ezer T, Gathumbi A, Cohen J, Kameri-Mbote P (2013). Access to justice: evaluating law, health and human rights programmes in Kenya. J Int AIDS Soc.

[57] UNAIDS RST Asia Pacific (2011). People Living with HIV Stigma Index – Asia Pacific regional analysis.

[58] Ekstrand ML, Ramakrishna J, Bharat S, Heyen E (2013). Prevalence and drivers of HIV stigma among health providers in urban India: implications for interventions. J Int AIDS Soc.

[59] Berkley-Patton JY, Moore E, Berman M, Simon SD, Thompson CB, Schleicher T (2013). Assessment of HIV-related stigma in a US faith-based HIV education and testing intervention. J Int AIDS Soc.

[60] Christensen JL, Miller LC, Appleby PR, Corsbie-Massay C, Godoy CG, Marsella SC (2013). Reducing shame in a game that predicts HIV risk reduction for young adult men who have sex with men: a randomized trial delivered nationally over the journal. J Int AIDS Soc.

[61] Risher K, Adams D, Sithole B, Ketende S, Kennedy C, Mnisi Z (2013). Sexual stigma and discrimination as barriers to seeking appropriate healthcare among men who have sex with men in Swaziland. J Int AIDS Soc.

[62] Lim T, Zelaya C, Latkin C, Quan VM, Frangakis C, Ha TV (2013). Individuallevel socioeconomic status and community-level inequality as determinants of stigma towards persons living with HIV who inject drugs in Thai Nguyen, Vietnam. J Int AIDS Soc.

# Sub article: Impact of HIV-related stigma on treatment adherence: systematic review and meta-synthesis

**DOI:** 10.7448/IAS.16.3.18640

**Published:** 2013-11-13

**Authors:** Ingrid T Katz, Annemarie E Ryu, Afiachukwu G Onuegbu, Christina Psaros, Sheri D Weiser, David R Bangsberg, Alexander C Tsai

**Affiliations:** 1 Connors Center for Women's Health and Gender Biology, Brigham and Women's Hospital, Boston, MA, United States; 2 Center for Global Health, Massachusetts General Hospital, Boston, MA, United States; 3 Harvard Medical School, Boston, MA, United States; 4 Harvard College, Cambridge, MA, United States; 5 Harvard School of Public Health, Boston, MA, United States; 6 Department of Psychiatry, Massachusetts General Hospital, Boston, MA, United States; 7 Division of HIV/AIDS, San Francisco General Hospital, University of California at San Francisco, California, United States; 8 Mbarara University of Science and Technology, Mbarara, Uganda

**Keywords:** HIV, stigma, disclosure, adherence, social support, poverty

## Abstract

**Introduction:**

Adherence to HIV antiretroviral therapy (ART) is a critical determinant of HIV-1 RNA viral suppression and health outcomes. It is generally accepted that HIV-related stigma is correlated with factors that may undermine ART adherence, but its relationship with ART adherence itself is not well established. We therefore undertook this review to systematically assess the relationship between HIV-related stigma and ART adherence.

**Methods:**

We searched nine electronic databases for published and unpublished literature, with no language restrictions. First we screened the titles and abstracts for studies that potentially contained data on ART adherence. Then we reviewed the full text of these studies to identify articles that reported data on the relationship between ART adherence and either HIV-related stigma or serostatus disclosure. We used the method of meta-synthesis to summarize the findings from the qualitative studies.

**Results:**

Our search protocol yielded 14,854 initial records. After eliminating duplicates and screening the titles and abstracts, we retrieved the full text of 960 journal articles, dissertations and unpublished conference abstracts for review. We included 75 studies conducted among 26,715 HIV-positive persons living in 32 countries worldwide, with less representation of work from Eastern Europe and Central Asia. Among the 34 qualitative studies, our meta-synthesis identified five distinct third-order labels through an inductive process that we categorized as themes and organized in a conceptual model spanning intrapersonal, interpersonal and structural levels. HIV-related stigma undermined ART adherence by compromising general psychological processes, such as adaptive coping and social support. We also identified psychological processes specific to HIV-positive persons driven by predominant stigmatizing attitudes and which undermined adherence, such as internalized stigma and concealment. Adaptive coping and social support were critical determinants of participants’ ability to overcome the structural and economic barriers associated with poverty in order to successfully adhere to ART. Among the 41 quantitative studies, 24 of 33 cross-sectional studies (71%) reported a positive finding between HIV stigma and ART non-adherence, while 6 of 7 longitudinal studies (86%) reported a null finding (Pearson's *χ* ^2^=7.7; *p*=0.005).

**Conclusions:**

We found that HIV-related stigma compromised participants’ abilities to successfully adhere to ART. Interventions to reduce stigma should target multiple levels of influence (intrapersonal, interpersonal and structural) in order to have maximum effectiveness on improving ART adherence.

## Introduction

Adherence to HIV antiretroviral therapy (ART) is a critical determinant of HIV-1 RNA viral suppression and health outcomes [1–3]. Early studies of ART adherence focused primarily on cognitive processes that may affect adherence, such as forgetfulness and health literacy [4–6]. More recently, investigators have shown that ART adherence in resource-limited settings, where treatment is generally provided free of charge, may be contingent upon structural barriers, such as food insecurity [7–12] or geographic isolation and lack of resources to pay for transportation to clinic [13–17].

The stigma of HIV and AIDS is one social process that has been broadly assumed to adversely affect multiple facets of engagement in HIV-related care as well as other factors that may undermine ART adherence, including HIV serostatus disclosure [18–20], social support [18, 21] and mental wellbeing [21, 22]. Goffman [23] conceptualized stigma as an “attribute that is deeply discrediting” imposed by society that reduces someone “from a whole and usual person to a tainted, discounted one” (p. 3). When the attribute becomes linked to “discrediting dispositions” (e.g., negative evaluations or stereotypes), these may come to be widely believed in the community [24]. During the labelling process [25–27], persons with and without the stigmatized attribute are separated into “them” and “us” [28] and may be subjected to overt acts of hostility and discrimination (enacted stigma) [29]. To avoid the potentially unpleasant consequences of revealing their discredited status, stigmatized persons may elect to conceal their seropositivity from others [20, 30]. Stigmatized persons may also internalize the beliefs held in the community and develop self-defacing internal representations of themselves (internalized stigma) – possibly leading to demoralization, diminished self-efficacy and emotional distress [31, 32].

Despite substantive advances in our understanding of the stigma process, the mechanisms through which stigma compromises ART adherence are not well understood. From a public health perspective, this is an important gap in the literature because sustained adherence [33] is a critical step in the spectrum of engagement in HIV-related care [34, 35]. Although the “test-and-treat” approach [36] has achieved a great deal of popularity in a brief amount of time, observers have expressed concerns that persisting stigma may pose a major obstacle to its success [37]. Therefore, we undertook this review to systematically assess the relationship between HIV-related stigma and ART adherence.

## Methods

### Search strategy and study selection

Three study authors (AER, AGO, ACT) searched nine electronic databases for published and unpublished literature: BIOSIS Previews, the Cumulative Index to Nursing and Allied Health Literature (CINAHL), Embase, the Educational Resources Information Center (ERIC), the Medical Literature Analysis and Retrieval System Online (MEDLINE), ProQuest Dissertations & Theses, PsycINFO, Web of Science (Science Citation Index Expanded, Social Sciences Citation Index, and Arts & Humanities Citation Index) and the World Health Organization African Index Medicus. In general, each set of search terms applied to these databases was oriented towards identifying studies of ART adherence among HIV-positive adults (Box S1). We conducted all searches in May 2011, with the exception of the ProQuest search, which was performed in June 2011. In February 2013, one study author (ACT) updated the MEDLINE search to identify more recent articles published since the study was initiated. We also consulted with experts in the field to identify additional studies that our systematic evidence search may have missed.

First we imported all records into EndNote reference management software (version X4.0.2, Thomson Reuters, Philadelphia, Penn.) and used the automated “Find Duplicates” function to exclude any duplicates. Then we screened the titles and abstracts of all records to identify studies that appeared to be potentially related to ART adherence among HIV-positive persons. We then obtained the full text of these articles for review, specifically to identify articles that reported either a quantitative estimate of association between a measure of stigma or disclosure and a measure of adherence, or qualitative findings about how stigma or lack of disclosure affected adherence. Although our review was focused on the relationship between stigma and adherence, we also chose to include studies examining the impacts of serostatus non-disclosure because it is a proximate consequence of stigma [19, 20]. Our goal in including qualitative studies as part of this systematic review was to inductively develop an in-depth understanding of persistent themes and assess the transferability of these themes across contexts [38]. Due to our interest in describing relationships between stigma and adherence across a wide range of countries, we chose not to exclude any study based on quality, country of origin or language.

### Quality assessment

To assess the quality of the included qualitative studies, we adapted questions representing the three key conceptual domains described in the Critical Appraisal Skills Programme quality assessment tool [39, 40]. These domains also mapped onto prominent criteria employed by previous researchers as identified in the review of qualitative quality assessment tools by Tong *et al*. [41]. The criteria we used were as follows: (1) the role of the researcher was clearly described; (2) the sampling method was clearly described; (3) the method of data collection was clearly described; and (4) the method of analysis was clearly described. We found that the included qualitative studies consistently described the role of the research and the method of data collection, but many studies reported neither the sampling method nor the method of analysis. Overall, 15 studies were assessed to be at low risk of bias (Table S1).

To assess the quality of the included quantitative studies, we developed an assessment tool based on the six major conceptual domains identified by Sanderson *et al*. [42]. The criteria we used were as follows: (1) the study was based on a probability sample of participants; (2) the study used a validated self-report scale to measure stigma or disclosure; (3) the study used a validated self-report scale or objective count (e.g., pill count, pharmacy refill) to measure ART adherence; (4) the statistical analysis accounts for missingness at random (MAR) or missingness not at random (MNAR) (longitudinal studies only); (5) the study design or statistical analysis controls or adjusts for potential confounding; and (6) competing interests were declared. Overall, all studies except for one were assessed to be at risk of bias (Table S2).

### Data synthesis

We organized studies by year of publication, country of origin, study design and types of measures employed. For the quantitative studies, due to substantial heterogeneity in the measures of stigma, serostatus disclosure and ART adherence that were employed, we did not attempt to summarize the data using meta-analysis. However, we examined patterns across studies with respect to the estimated associations and the precision of these estimates.

For the subset of qualitative studies, our goal was to generate new theoretical insights. Therefore, we used the iterative process of meta-synthesis proposed by Noblit and Hare [43] to identify themes that recurred frequently or were prominently featured throughout the data. Meta-synthesis (also described as meta-ethnography) is an interpretive approach to summarizing qualitative research that has been employed to understand vaginal practices in sub-Saharan Africa [44], delays in presentation for cancer care [45] and adherence to tuberculosis treatment [46]. Key themes and concepts were collected and peer-reviewed for inclusiveness. First-order findings (quotations) were used to support second-order interpretations (authors’ analyses) to gain new insight into the relationships between stigma and ART adherence. A summary definition of second-order constructs was generated for further clarification and then consolidated into a line of argument that led to a third-order analysis, which we describe below. Based upon the data set, we achieved theoretical saturation within the first 10 manuscripts, although basic elements for meta-themes were evident as early as six manuscripts. Variability within the data followed similar patterns, consistent with prior qualitative meta-synthesis research [47].

## Results

Our initial search yielded 14,854 records, of which 9009 were identified as duplicates through the use of automated software (Figure 1). After screening the titles and abstracts of the remaining 5845 records, we eliminated 4000 records that did not appear to contain relevant data on adherence or provided potentially relevant adherence data specific to a specialized population (e.g., children or pregnant women), eight unpublished conference abstracts or dissertations matched to subsequently published peer-reviewed journal articles in our database of records, 199 reviews that did not report original data, and 678 additional duplicates that had been misclassified as non-duplicates by the automated software. We retrieved 960 journal articles, unpublished dissertations and conference abstracts for full text review. Of these, 889 did not contain quantitative or qualitative data relating stigma or disclosure to ART adherence and were therefore excluded. Expert review suggested four additional articles for inclusion. The final sample included 75 studies: 34 qualitative studies and 41 quantitative studies.

**Figure 1:** Flow diagram. We identified 14,854 records by searching nine electronic databases, yielding 34 qualitative studies and 41 quantitative studies.

### Synthesis of qualitative studies

Thirty-four qualitative studies conducted during 1999–2013 were included in the review, including one written in French. Represented in these manuscripts were views from 1328 study participants in 26 countries. Of note, only one country from the UNAIDS Eastern Europe and Central Asia region was represented: Serbia and Montenegro. The median number of participants was 38 (interquartile range (IQR), 27 to 48; range, 6 to 118). Participants included adult men and women ranging in age from 18 years to over 60 years old, HIV-positive persons as well as providers of HIV care, single persons and those in intimate partnerships, and persons with and without children. Specific high-risk groups were well represented and included men who have sex with men, injection drug users and commercial sex workers.

After reviewing each of the qualitative studies in detail, we identified 24 second-order constructs, supported by original quotes, in multiple manuscripts. Second-order constructs relevant to ART adherence were identified, and key themes were generated into a line of argument that led to 15 third-order constructs. These were grouped into five distinct third-order labels that we categorized as themes, all of which are described in detail in Table 1.

**Table 1:** Qualitative studies on stigma, disclosure and ART adherence (*N*=34)

Third-order labels	Third-order constructs	Second-order constructs	Summary definition	First-order constructs	Source(s)
Social support	Intimate and familial relationships	Spousal, peer and familial support	Participants discussed support from spouses, peers and family as critical for overcoming stigma and maintaining adherence, as was having a sense of obligation to family	*Well, they encourage me, like my folks have [said] ‘you took your medication today?’* [55, p. 5] *I am thankful to God for giving me such a good husband. He takes care of me well. I have given him a lot of trouble. He has spent so much money for my treatment*. [54, p. 496]	[48–70, 78, 79]
		Context of male-dominated household decision-making	In cultures where men are typically heads of their households, women fear disclosing their serostatus as they fear social isolation and abandonment. Women may choose to have providers give the test information to their husbands by bringing them in for testing. In addition, in some cultures, women cannot travel alone to clinic to pick up their medications.	*[After testing positive] I went back home and first kept quiet for two days. I asked myself, how can I approach him to tell him? One day when he came back, I told him, they checked my blood but they refused to give me the results until I take my spouse in for testing. I convinced him and he accompanied me*. [57, p. S88]	[49, 52, 54, 57, 64, 68, 72]
		Healthy children reducing stigma	Clinical response to ART in children of HIV-positive mothers reduces stigma and often re-establishes mother's role in family	*Then when she saw that since giving birth, my baby was not falling sick (the other children used to be sickly), that my baby was looking nice, did not have a rash, and was growing fast she said ‘I used to think you were infected. I had taken you out of all of my plans.’* *I responded that, ‘I am not infected, don't you see my baby?’ So that's where I ended her suspicions about my being sick. Now she knows that I am not infected, which is not true*. [57, p. S88]	[57]
	Compromised relationships	Physical manifestations of HIV and AIDS leads to social isolation	Physical signs of ill health may lead to abandonment or to the belief that the HIV-positive person is already dead	*These days when people come to know that you have AIDS they don't want to come near you, as if you are an abominable thing (‘bakwenyinyala’). You cannot feel free. Wherever you go they start talking, ‘See that one, she is sick.’* [57, p. S88])	[55–57, 64–67, 69, 71, 72]
		Complex regimens with large numbers of medications	Complex regimens characterized by a large pill burden that required undesired disclosure in order to adhere	*… things got messed up, like my schedule, wherever you go, you got to bring the medicine pack, it's even upsetting to open a bunch of medicines*. [53, p. 3] *Our guests were at my home; I didn't feel comfortable pulling out my drug boxes, then I forgot and missed my drugs*. [74, p. 467]	[12, 52, 53, 55, 59–61, 63–65, 69, 72–74]
		Social rejection	Participants adopted strategies of concealment because they feared ridicule or discrimination if they disclosed their HIV status or if they were seen taking their medications	*My company made it hard. You know, because I felt like I had to hide my medicine, you know? All, you know, for shame*. [55, p. 5] *Ordinary public thinks that if they mingle along with the patient means they will get HIV*. [48, p. 532]	[12, 48, 49, 54–56, 59, 64–72, 76, 77]
		Treatment side-effects	Observable side-effects of medications (e.g., dysmorphic body changes) carried stigma	*It wasn't hard for me to take my medicines; it was the things that people would say …* [55, p. 5] *The medications compounded the way I felt, how badly I felt, but I kept taking them because I knew it was temporary*. [74, p. 466]	[12, 53, 55, 56, 60, 61, 63–66, 68, 71, 73, 74, 76]
	Negotiating disclosure to a child	Stigma associated with a child's HIV status	Maternal shame and stigma related to perinatal acquisition of HIV kept them from informing HIV-positive children about their seropositivity, with attendant challenges in ART adherence	*The thing that disturbs me is that I always think what will I tell my child when he grows to a level of understanding and he asks me why he is taking drugs. Because even now he asks me, ‘Mummy I no longer cough but why am I still taking drugs every day?’ What will I tell the child?’* [57, p. S88]	[48, 53, 57, 64]
Self-Identity	Race/minority status	Outsider status based on race	HIV-positive persons who belonged to racial minority groups felt further stigmatized and socially isolated		[49, 55]
	Sexual orientation/relationship status	Impact of social norms on stigma and willingness to disclose	Social norms further stigmatized HIV-positive persons if the mode of acquisition was not regarded as socially acceptable behavior	*In the gay community, I can't go to somebody and say, ‘I'm HIV.’ People avoid the subject. They do not disclose it*. [51, p. 906]	[50, 51, 54, 61–63, 71–74, 76, 77]
	Substance abuse	Social marginalization of injection drug use intensified for HIV-positive users	Participants who actively used illicit substances discussed being unable to establish relationships with HIV-negative persons or non-injection drug users, and feeling socially isolated	*Drug users, it's a group that right now everyone in society hates. Including myself, I hate myself. But the problem is [that] there is nothing I can do*. [77, p. 1244]	[51, 77]
	Redefining healthy living	Self-perception as pro-active/choosing to be healthy	Participants described knowing friends who died from AIDS and not wanting to be like them; the notion of “choosing to live” [74, p. 466]	*Then I had some friends die of full-blown AIDS, and I looked around and seen what a horrible death that was* … *And so I know I wanted to live, and I wouldn't want to send my family through that. So I knew I had to take my medicine*. [55, p .4] *I didn't want to start drugs, but I had seen two AIDS patients dead. They hadn't used drugs*. [74, p. 466]	[52–56, 58, 59, 61, 66, 70, 72–74]
	Acceptance of status	Self-identifying as someone who is HIV-positive	Participants who had accepted their status found it easier to adhere vs. those who had difficulty taking medications because it reminded them of their seropositivity	*The thing is it's my life, you know. I don't see it much if somebody comes to me and tells me that, ‘you've got HIV – you are HIV’. I don't have a problem with that because that's not his problem, that's my problem you know. As long as I know how I manage it, I don't give a damn about any other person*. [56, p. 303]	[50, 56, 67, 69, 70, 73, 74]
Poverty	Economic implications of HIV	Mutually reinforcing relationship between poverty and stigma	HIV-related illness and perceived economic inadequacy leading to social exclusion	*They see it as useless to assist someone who has a shorter time to live. It's like wasting money. Why assist someone who is going to die?* [67, p. 1311] *There is no need to waste any more money on her, give me this lady and I will put her in the car and take her to her rural home with her children*. [72, p. 875] *With ART, I have returned to work and earn money; friends who avoided me in the past are now more accepting of me* … *If I do not take this medicine as I am told, I will get sick and*	[54, 56, 67, 72]
				*won't be able to work again. People will also begin to avoid me again*. [72, p. 877]	
			Economic insecurity resulting from HIV-related stigma	“I thought that people would know my HIV status when I have illnesses regularly and am out of the office several times.” [67, p. 1311]	[54, 67, 72]
		Costs associated with treatment	Costs associated with purchasing medications or with travel to the treatment centre (along with loss of wages) made even free ART prohibitively expensive for some, leading to treatment interruptions	*Even if I go for work I get Rs 100 in which 60 goes for tablets. So in the rest I have to manage the other expenses, which is very difficult. Medicines for HIV infection should be like other general medicines where everyone can afford to buy. Now I am not sure I can continue the treatment for a long time*. [48, p. 529]	[12, 48, 54, 60, 61, 64, 67, 68, 70, 72, 76, 77]
Coping	Maladaptive strategies	Anger at diagnosis	Inability to accept diagnosis and anger at diagnosis, with associated inability to engage in HIV care and adhere to ART	*I was mad, and I was upset, and I was in denial. And it took me five years to tell anybody that was close to me. So I kept that to myself for a long time, and I was very angry. Right now, I still don't take [the medicines] like I should*. [55, p. 4]	[55, 72]
		Substance use and abuse	Consumption of alcohol and use of drugs provided a temporary refuge but also made ART adherence more difficult	… *I began to skip the medication. I said to myself, ‘Well, today I'm not taking it, ‘cause I'm gonna party* … *[drink] Come on, I was born to party* … [53, p. 3]	[52, 53, 59, 73, 78]
		Fear that drugs are dangerous and/or that HIV is a curse fuelled by stigma	Participants expressed concerns about taking medications feared to be dangerous or toxic	*Rural people do still not believe this medicine [ART] works for HIV patients. HIV people will die eventually either taking or not taking ART. Why should I die by taking these malicious pills?* [68, p. 3]	[12, 68, 71, 72]
	Acceptance	Knowledge that taking medications will provide benefits	Acceptance of the diagnosis counter-balanced stigma, as participants described moving on a continuum from willingness to take medications, to engagement in pro-active healthy lifestyle changes	*This is your own responsibility. You know what you got. You know you got medicine to take. No matter what nobody else say or how peoples feel about it, you got to take care of yourself first*. [55, p. 4]	[54–56, 58, 59, 66, 67, 69, 70, 72–74]
				*During [the] last 5 years, taking medications showed me its benefits. My CD4 cells [sic] count was 80, with high viral loads, but now I am okay. They actually helped and gave me more longevity*. [74, p. 467]	
	Mental wellbeing	Treatment of depression and anxiety related to diagnosis	Treatment of depression resulting from HIV diagnosis could ameliorate stigma and social isolation		[49, 57, 65, 67, 69, 72, 73, 77]
	Morality and spirituality	Notion of God's will	Participants discussed relinquishing control of their lives to God and putting their faith in a higher power to help them overcome adversity	*I just want to be a living witness, that God has all power. He can do all things, and I put my faith and trust in Him*. [55, p. 4–5] *I believe in the power of prayers – I believe in my church. It's got hope for me … because I have a feeling that God loves us … God is the person that gave you that disease, and God is the person who can take it out from you* … *You have to have faith in that*. [56, p. 305]	[12, 52, 54–61, 67, 69, 72]
Health systems	Importance placed in clinical support staff	Nursing and physician support to gain trust and overcome social isolation associated with stigma	Programs supporting social support and building trust with the adherence nurse or doctor were described as essential for people who reported stigma as a barrier to ART adherence	*I felt so alone. It's nice to know that somebody does understand what it is all about and you can depend on that person*. [75, p. 117] *I trust the doctors and nurses. Therefore I started the drugs*. [74, p. 466]	[50, 55, 58–60, 62, 63, 67, 69, 70, 72–75, 80]
		Support in designing tolerable combination of medications that are easily available	Participants felt it was easiest to adhere if they were on tolerable medications and if providers were available in the event of adverse side effects vs. those who feared taking medications because of potential side effects or complications. It was also important to ensure that there were no stock-outs and that medications were easily available.	*I didn't know the advantages of medications, I feared the complications; therefore, I started it very late. Actually, it was [a] wasting of my time*. [74, p. 466] *We can't have any plan, because we don't know when supplies will fail. Some people can get medicine and some can't*. [80, p. 317]	[55, 58–60, 73, 74, 80]
	Family-driven treatment	Establishing treatment for all members of the household	Treatment to all HIV-positive members of a family (including spouse and children) provided support to overcome stigma and improve medication adherence		[54, 57]

#### Theme 1: social support

The most commonly cited theme related to ART adherence was the role of social support. Specifically, participants described spousal or familial support as being critical for enabling them to overcome enactments of HIV-related stigma and other obstacles to care and successfully adhere to treatment [48–70]. As noted by one 45 year-old HIV-positive rice dealer in Chennai, India,

A person without a family is like a single tree struggling for life. My children and my wife are my backbone. Now I have brought changes in myself and want to achieve many things. [54, p. 496]

Compromised relationships could result from either HIV illness or HIV treatment. Many participants described being socially isolated due to the physical manifestations of HIV-related illness [55–57, 64–67, 69, 71, 72] . As described by one HIV-positive mother in Kampala, Uganda,

These days when people come to know that you have AIDS they don't want to come near you, as if you are an abominable thing (‘bakwenyinyala’). You cannot feel free. Wherever you go they start talking, ‘See that one, she is sick’. [57, p. S88]

On the other hand, HIV treatment could also undermine social relationships. Unintended disclosure was viewed as a consequence of being on complex regimens that often needed to be taken multiple times per day [12, 52, 53, 55, 59–61, 63–65, 69, 72–74]. This was commonly discussed in some of the older studies, which were conducted during a time when pill burden was high and participants reported difficulty in understanding when and how to take their medications [12, 50, 52, 58, 60, 61, 64, 67, 68, 70, 74, 75]. Attempts at concealment, such as by hiding medications or furtively taking medications, were described as contributing to treatment interruptions [12, 48, 49, 54–56, 64–72, 76, 77].

In addition, some participants felt that the medications themselves were associated with side effects that had unwelcome physical manifestations:

[ART] has given more side-effects for me such as vomiting, herpes/zoster, and skin rashes. I have lost my sight in my right eye and my left eye also has poor vision.

– HIV-positive woman from far western Nepal [68, p. 7]

Desire to avoid these physical stigmas, or fear of “the thing [sic] that people would say” [55, p. 102], motivated some participants to avoid taking medications and evade detection.

A more circumscribed discussion in the literature related to norms about gender roles, particularly in patriarchal cultures. Byakika-Tusiime *et al*. [57] explained how HIV-positive women were better able to adhere to ART when others did not identify them as being infected with HIV. An HIV-positive mother could evade detection by giving birth to an uninfected child and establishing her role as a caretaker. This was discussed by an HIV-positive mother in Kampala, Uganda, who described how giving birth to a healthy baby changed her family's assumptions about the inevitability of her death:

When [my sister] saw that since giving birth, my baby was not falling sick (the other children used to be sickly), that my baby was looking nice, did not have a rash, and was growing fast she said ‘I used to think you were infected. I had taken you out of all my plans.’ I responded that ‘I am not infected, don't you see my baby?’ So that's where I ended her suspicions about my being sick. Now she knows that I am not infected, which is not true. [57, p. S88]

Other authors mentioned the importance of women being able to hide their seropositivity in settings where men dominated household decision-making, so as to avoid social isolation and/or abandonment [49, 52, 54, 64, 68, 72]. In these settings, some women reported relying on healthcare providers to inform their sexual partners of their HIV status rather than informing their partners directly themselves.

Women who gave birth to an HIV-positive child experienced feelings of shame and social rejection, both within and outside of the family. Participants in these studies discussed the difficulty associated with disclosing the status of an HIV-positive child, particularly in communities where HIV was highly stigmatized and where appearing ill often led to abandonment by one's family and community [48, 53, 55–57, 64–67, 69, 71, 72] .

The thing that disturbs me is that I always think what will I tell my child when he grows to a level of understanding and he asks me why he is taking drugs. Because even now he asks me, ‘Mummy, I no longer cough but why am I still taking drugs every day?’ What will I tell the child?’

– HIV-positive mother from Kampala, Uganda [57, p. S88]

#### Theme 2: self-identity

Self-identity was another prominent theme identified in these studies. Multiple studies elaborated on how social norms intensified the stigma of HIV and undercut participants’ willingness to disclose to others [50, 51, 54, 61–63, 71–74, 76, 77] . In many settings, study participants described HIV-related stigma as being layered on top of pre-existing inequalities, such as those related to gender, race or sexual minority status:

I often hear my friends speak negatively about people being HIV-positive. They always have degrading or negative remarks to make. What I dislike most is when they call people names (e.g., fagot, whore, and junkie). Whenever I go out with them or they come over to visit, I don't take my medications. I could never let them know I'm positive.

– HIV-positive African-American woman living in Baltimore, U.S. [49, p. 684]

Konkle-Parker *et al*. [55] and Edwards [49] both discussed the difficulty that persons in a minority group experienced when self-identifying as HIV-positive, since it often led to further enactments of stigma, including overt discrimination and/or acts of hostility. In such a setting (and consistent with Theme 1), many participants opted not to take their medications for fear of disclosure. Ware *et al*. [51] and Sabin *et al*. [77] described the added burden and social isolation that accompanied an HIV diagnosis among participants who actively used illicit substances. In these cases, self-efficacy was often low, and the lifestyle modifications required to achieve consistent adherence proved to be challenging for participants.

Drug users, it's a group that right now everyone in society hates. Including myself, I hate myself. But the problem is [that] there is nothing I can do.

– 40-year-old, injection drug using, HIV-positive married man living in Old Dali, Yunnan Province, China [77, p. 1244]

The experiences of persons who had internalized the stigma of HIV was contrasted with reports of persons who had accepted their HIV status and who had successfully cultivated a self-perception of being pro-active and “choosing to live” [74, p. 466]. These participants were able to successfully adhere to their ART regimens [52–56, 58, 59, 61, 66, 72–74] . In these studies, participants described how the deaths of HIV-positive friends motivated them to take responsibility for their own treatment. Some participants also described feeling strong enough to continue to work and provide for their families.

Then I had some friends die of full-blown AIDS, and I looked around and seen what a horrible death that was … And so I know I wanted to live, and I wouldn't want to send my family through that. So I knew I had to take my medicine and … I know I wants to live

– HIV-positive African-American study participant from Mississippi [55, p. 4]

#### Theme 3: poverty

In several studies, participants also described how poverty and stigma were intertwined in a reciprocal and mutually reinforcing relationship (Figure 2). Participants spoke of being viewed as weak, unproductive members of society and of being excluded from informal networks of mutual aid:

They see it as useless to assist someone who has a shorter time to live. It's like wasting money. Why assist someone who is going to die?

– HIV-positive person living in Dar es Salaam, Tanzania [67, p. 1311]

Thus, conditions of poverty worsened stigma by emphasizing one's economic worth (or lack thereof) to the community. In resource-limited settings where social networks serve as a form of informal risk-sharing (consistent with Theme 1), and where neighbours often live in close proximity to each other, participants reported feeling ashamed and ultimately more stigmatized by the public nature of unwanted disclosures:

I used to have a neighbour … who knew my status. At times, I used to get porridge from KENWA and bring it home. She had a child who was my kid's friend and age mate. One day, I gave the porridge to her child and [she] was furious and shouted at the little girl; ‘where did you get that porridge? Take it back! You are taking porridge from people with AIDS,’ she was shouting outside and I was in the house.

– HIV-positive woman living in a slum community in Nairobi, Kenya [72, p. 874]

Conversely, stigma was also found to exacerbate the economic impacts of HIV. Economic insecurity resulting from stigma and social isolation was particularly challenging for widowed women who had lost their husbands to AIDS. Tarakeshwar *et al*. [54] described 9 out of 10 widowed women living in Chennai, India, who were discriminated against, experienced housing insecurity and were isolated by their in-laws after their husbands’ deaths. Stigma was also cited as leading to embarrassment at work, and ultimately causing participants to stop working in order to avoid disclosure, leading to further economic insecurity:

I was on 5 days leave [when I came to test for HIV] and I stayed another week. They were looking for me at work … I was staying [away] because I was sort of embarrassed by my own things. I was embarrassed by my own fate.

– 39-year-old HIV-positive unmarried man living in Gaborone, Botswana [56, p. 304]

Lastly, for participants in resource-limited settings, financial burdens posed a significant barrier to adherence due to costs of the medications themselves, the costs of transportation to pick up free medications from clinic, or wages foregone when attending clinic [12, 48, 54, 60, 61, 64, 67, 68, 70, 72, 76, 77]. These treatment interruptions further compromised participants’ health, reinforcing their status as unproductive members of the community.

**Figure 2:** Reciprocal relationships between poverty and stigma. HIV-associated illness reinforces the perceived economic inadequacy of HIV-positive persons, who are excluded from networks of mutual aid. Stigmatized persons are excluded from the community, undermining their social support and worsening economic insecurity.

#### Theme 4: coping

Coping emerged as a means by which participants attempted to manage stigma and adhere to ART. At times, these coping strategies were maladaptive and detrimental to health. Many participants reported low self-esteem, depressed mood or anger related to their diagnosis, citing their inability to cope with their HIV status as the reason they failed to take their medications [49, 55, 57, 65, 67, 69, 72, 73, 77]:

I was mad, and I was upset, and I was in denial. And it took me five years to tell anybody that was close to me. So I kept that to myself for a long time, and I was very angry. Right now, I still don't take [the medicines] like I should.

– HIV-positive study participant recruited from a large public infectious disease clinic in Mississippi [55, p. 4]

In addition, ART misconceptions (e.g., “Why should I die by taking these malicious pills?”[68, p. 3]) and HIV conspiracy beliefs that were often fuelled by stigma led to ART non-adherence [12, 68, 71, 72]. Participants who lacked the internal resources to cope adaptively described how they self-medicated with alcohol or illicit substances, but these behaviours further compromised their abilities to consistently adhere to treatment [52, 53, 73].

Adaptive coping strategies included those that supported adequate treatment for depression and anxiety, along with acceptance of one's diagnosis. These strategies appeared to provide a protective buffer against stigma and promote acceptance of lifelong treatment [12, 54–56, 58, 61, 67, 69, 72–74] , particularly for those who were able to incorporate these into their new self-identities (consistent with Theme 2). Likewise, spirituality and faith in God enabled some participants to overcome adversity associated with disclosure and HIV-related stigma and to consistently take their medications [12, 52, 54–56, 61, 67, 69, 72] :

I am a Christian and a believer, I know that God exists but those medicines also were inspired by God. God is the one who gave inspiration to doctors to make those medicines for us.

– 59-year-old man on ART, from the Democratic Republic of Congo [12, p. 4]

#### Theme 5: health systems

A theme common to several studies was that different aspects of the health system could help to moderate the impacts of HIV-related stigma on ART adherence. Specifically, compassionate human capital elements could establish a supportive clinical environment for patients, while certain clinical programs could be designed to address care for the entire family. As noted by one HIV-positive participant in Connecticut,

[The nurses] take care of me, I love the people, they go to your home, like they're my friends. Every time they say, how are you doing? Do you need anything? [75, p. 117].

Doctors and nurses engaged in patient-centred care could help to establish bonds of trust and empower patients to overcome the stigma associated with taking medications [50, 55, 60, 62, 63, 67–70, 72–75, 80]. Some participants described how medication regimens optimized for tolerability, with the fewest side effects and lowest pill burden, allowed them to minimize the possibility that others in the community might recognize their HIV status; this, in turn, decreased stigma and increased participants’ chances of successfully adhering to treatment [55, 58, 60, 73, 74, 80]. Lastly, family-driven treatment programs designed to bring all HIV-positive members of the family into care were thought of as cultivating greater social support, reducing stigma and improving ART adherence [54, 57].

### Synthesis of quantitative studies

Data from the quantitative studies were consistent with these lines of inquiry. Our systematic search protocol identified 34 cross-sectional and seven longitudinal studies conducted between 1997 and 2009 that examined the association between either stigma or disclosure and ART adherence (Table 2). These studies included data from 25,387 participants living in 18 different countries, with the largest proportion of studies (15/41 (37%)) based on data collected in the United States. The median number of participants was 300 (IQR, 201–439; range, 65–5760). Twenty-three studies (56%) measured HIV-related stigma, while 21 studies (51%) measured disclosure of seropositivity and three studies (7%) included a measure of both. Most of the studies examining the effect of HIV-related stigma (18/23 (78%)) on ART adherence employed a scale for which some evidence of reliability and/or validity had previously been obtained. In five studies, a multifactor scale was used (28%), while in others specific aspects of HIV-related stigma were measured, including enacted stigma (2/18 (11%)), disclosure concerns (3/18 (16%)), perceived stigma (3/18 (16%)) and internalized stigma (11/18 (61%)) (total percentage exceeds 100% as some studies administered more than one scale). Of the 18 studies that used a formal scale for measuring stigma, only three studies (17%) were conducted in a sub-Saharan African setting, and each of these used a newly developed stigma scale. The most widely used scale, administered in six studies, was the four-factor HIV Stigma Scale developed by Berger *et al*. [81]. To measure ART adherence, most studies used self-report (30/41 (73%)). Of these, slightly more than half (16/30 (53%)) employed a scale with previously demonstrated evidence of reliability or validity; the AIDS Clinical Trials Group measure developed by Chesney *et al*. [94] was the most frequently used among these (10/16 (63%)).

**Table 2:** Studies reporting a quantitative measure of association between stigma or disclosure and ART adherence (*N*=41)

Citation	Study design and population	Study period	Primary stigma or disclosure measure	Primary adherence measure	Findings
Birbeck *et al*. [82]	Cross-sectional study of 255 outpatients from 3 clinics in rural Zambia	2005–06	Disclosure of HIV seropositivity to spouse, family, friend, or no one	“Good adherence” was defined as (a) attendance at all ART clinic visits, (b) no lapse in drug collection, and (c) no clinic documentation indicating adherence problems	Of those who had not disclosed to anyone, only 17% had good adherence, whereas 50–66% of those who had disclosed to a spouse, family member or friend had good adherence (*p*=0.047)
Adeyemi *et al*. [83]	Cross-sectional study of 320 outpatients on ART for at least 12 months, recruited in 2 cities in Nigeria	2009	Unclear measure (“stigma and discrimination”)	Greater than one week delay in ART refill, as determined by comparison of date of scheduled appointment and date of actual refill	“Stigma and discrimination” was associated with increased odds of delayed ART refill (AOR=1.4; 95% CI=1.1–1.7), after adjusting for distance to clinic and occupation
Boyer *et al*. [84]	Cross-sectional study of 2381 inpatients in 27 national, provincial and district hospitals throughout Cameroon	2006–07	Personal experience of HIV-related stigma from partner or close family members	Self-reported ART adherence based on a 14-item scale related to dose-taking and dosing schedule [85], with “non-adherent” persons defined as those who had taken<100% of prescribed doses in the past four weeks but did not report any treatment interruptions lasting>2 consecutive days	Experience of discriminatory behaviours was associated with increased odds of non-adherence (AOR=1.74, 95% CI=1.14–2.65), after adjusting for household income, binge drinking, food insecurity, social support and healthcare supply-related factors
Cardarelli *et al*. [86]	Cross-sectional study of 103 outpatients at a preventive medicine clinic for low-income persons in Texas	2008^a^	40-item HIV stigma scale [81]	Non-adherence was defined as a positive screen on the simplified medication adherence questionnaire, a modified version of the Morisky scale, which contains 6 items related to forgetfulness or carelessness about ART dose taking behavior [87, 88]	The stigma score did not have a statistically significant association with non-adherence (AOR=1.01; 95% CI=0.98–1.03), after adjusting for race, education, racial discrimination, social support, perceived stress or sense of control
Carlucci *et al*. [89]	Cross-sectional study of 424 outpatients at a mission hospital in rural Zambia	2006	Single-item question about perceived stigma	Pill count adherence measured over a median of 84 days (interquartile range, 56–98 days), with optimal adherence defined as≥95% doses taken	Perceived stigma did not have a statistically significant association with adherence (AOR=1.1; 95% CI=0.55-2.1), after adjusting for travel time and transportation cost
Charurat *et al*. [90]	Cross-sectional study of 5760 persons initiating ART at five university teaching hospitals in urban Nigeria	2005–06	HIV disclosure to spouse or family members	Pharmacy refill adherence rate (days of medication dispensed divided by days between visits), with poor refill adherence defined as<95% adherence	Disclosure was associated with decreased odds of low adherence (AOR=0.85; 95% CI=0.75–0.97), after adjusting for education, employment, distance to clinic
					and time on ART. There was no univariable association with loss to follow up (OR=0.96; 95% CI=0.82–1.12)
Colbert [91]	Cross-sectional analysis of baseline data on 335 persons participating in a 5-year randomized clinical trial conducted in clinics and HIV service organizations in western Pennsylvania and northeast Ohio	2003–07	40-item HIV stigma scale [81]	30-day adherence as measured with electronic event monitoring, with poor adherence defined as<85% adherence	Neither personalized stigma (AOR=0.98; 95% CI=0.95-1.02) nor negative self-image (AOR=1.00; 95% CI=0.94–1.06) had a statistically significant association with poor adherence, after adjusting for mental health, self-efficacy and health literacy
Diiorio *et al*. [92]	Cross sectional study of 236 outpatients (32% women) from an HIV clinic in Atlanta	2001–03	Four items related to internalized stigma from the Perceived Stigma of HIV and AIDS Scale [93]	Five items related to logistical adherence barriers from the ACTG Adherence Instrument [94]	In a structural equation model, stigma had an indirect negative association with adherence: stigma was found to erode self-efficacy, which in turn was directly associated with adherence
Dlamini *et al*. [95]	Longitudinal study of 698 persons (72.3% on ART for more than 1 year) enrolled in a larger cohort in Lesotho, Malawi, South Africa, Swaziland and Tanzania	2006–07	33-item HIV and AIDS Stigma Instrument-PLWA [96]	ACTG Adherence Instrument [94]	Persons who did not report any missing doses experienced a steeper decline in mean stigma over time, after adjusting for education, employment, food insecurity, social support and years since diagnosis
Do *et al*. [97]	Cross-sectional study of 300 outpatients from the largest ART clinic in Botswana	2005	Disclosure of seropositivity to a partner	Adherence defined as no missed doses with four-day and one-month recall, and no missed refill visits with 90-day recall	Non-disclosure was associated with an increased odds of non-adherence (*p*<0.02; AOR not shown), after adjusting for education, employment, travel time, duration of ART, depression, alcohol use and household size
Franke *et al*. [98]	2-year longitudinal study of 134 adults initiating ART in urban Peru	2005–09	40-item HIV stigma scale [81]	30-day self-report, with “suboptimal” adherence defined as<95% [94]	On univariable analysis, perceived HIV stigma was not associated with suboptimal adherence (OR=1.03, 95% CI 0.94–1.12) and was not included in the final multivariable model
Goldman *et al*. [99]	Longitudinal study of 913 treatment-naïve adults initiating ART in urban Zambia	2006–07	Disclosure of HIV status to partner or spouse	Medication possession ratio based on cumulative days late for pharmacy refill visits, with≥95% defined as optimal adherence	Disclosure did not have a statistically significant association with optimal adherence (estimates not reported)
Kalichman *et al*. [100]	Cross-sectional study of 81 adults recruited from HIV clinical and community support services in Atlanta	2005^a^	4-item self-efficacy for disclosure decisions scale	6-item standard medication adherence self-efficacy scale [101]	Self-efficacy for disclosure had a statistically significant correlation with self-efficacy for engaging in care (*r*=0.24, *p*<0.05) but not with self-efficacy for medication adherence (*r*=0.19, p>0.05)
Kalichman *et al*. [102]	Cross-sectional study of 145 adults recruited from HIV clinical and community support services in Atlanta	2008^a^	6-item Internalized AIDS-Related Stigma Scale [103]	Monthly unannounced pill count conducted by telephone, averaged over four months, with adherence defined as ≥85% of doses taken	Internalized stigma had no statistically significant association with adherence (AOR=0.99, 95% CI 0.87–1.13)
Li *et al*. [104]	Cross-sectional study of 386 adults (23.9% of whom were treatment-naïve), recruited from four district hospitals throughout Thailand	2007	8-item scale assessing serostatus disclosure to various social ties [105] and 9-item internalized stigma scale [106, 107]	30-day self-reported adherence, with good adherence defined as no missed doses	Good adherence had a statistically significant association with disclosure (AOR=1.70; 95% CI=1.07–2.70) but not internalized stigma (AOR=0.83; 95% CI=0.51–1.36), after adjusting for education, employment, instrumental social support, depression symptom severity, family functioning and years since diagnosis
Li *et al*. [108]	Cross-sectional study of 202 outpatients enrolled in the Chinese national free ART program, selected from six HIV treatment sites in Hunan Province, China	2009	34-item, five-factor HIV-related stigma scale [109]	Seven-day self-reported ART adherence as measured on a 5-point Likert scale [110]	Stigma was associated with a reduced odds of good adherence (AOR=0.96; 95% CI=0.93–0.98), after adjusting for education, family income, years since diagnosis and recent drug use
Lucero *et al*. [111]	Cross-sectional study of 65 persons aged >50 years recruited from two hospitals in New York City	2001^a^	Disclosure of HIV seropositivity to family and friends	Self-report, rated on a 4-point Likert-type scale, with good adherence defined as “taking medication all of the time”	Disclosure was associated with better adherence (estimates not shown)
Martinez *et al*. [112]	Longitudinal study of 178 girls and women aged 15-24 years recruited from 5 cities throughout the U.S.	2003–05	The disclosure concerns and negative self-image subscales of the HIV stigma scale [81]	12-item scale to measure self-reported dosing and scheduling adherence with a two-day recall	Baseline stigma did not have a statistically significant association with complete adherence at 12-month follow-up (*b*=−0.012, *p*>0.50).
Mo and Mak [113]	Cross-sectional study of 102 adults recruited from an outpatient clinic in Hong Kong	2009^a^	22-item self-stigma scale [114]	ACTG Adherence Instrument [94], with participants classified as “adherers,” “unintentional non-adherers,” or “intentional non-adherers”	Intentional non-adherers had greater self-stigma (4.11, SD 0.74) than adherers (3.78, SD 0.96) and unintentional non-adherers (3.22, SD 0.92) F[1,100]=7.58, *p*<0.001)
Molassiotis *et al*. [115]	Cross sectional study of 136 adults recruited from an outpatient clinic in Hong Kong	2002^a^	HIV disclosure to others, including spouses or partners	ACTG Adherence Instrument [94], with good adherence defined as≥95% adherence	Disclosure did not have a statistically significant association with adherence (estimates not shown)
Muyingo *et al*. [116]	Secondary analysis of data from a randomized trial of 2957 treatment-naïve adults initiating ART at two treatment centres in Uganda and one in Zimbabwe	2003–04	Disclosure of HIV serostatus	Drug possession ratio, with complete adherence defined as 100% adherence	Disclosure did not have a statistically significant association with complete adherence (estimates not shown), after adjusting for education and duration of current partnership
Nachega *et al*. [117]	Cross-sectional study of 66 outpatients at an HIV clinic in South Africa	2002	Fear of stigma from partner	ACTG Adherence Instrument [94]	On univariable analysis, fear of stigma from partner was associated with reduced odds of >95% adherence (OR=0.13; 95% CI=0.02–0.70)
Olowookere *et al*. [118]	Cross sectional study of 318 adults on ART for at least three months, recruited from a university hospital HIV clinic in Nigeria	2007	Disclosure of HIV serostatus	Seven-day self-reported adherence, with non-adherence defined as<95% doses taken	Non-disclosure was associated with increased odds of non-adherence (AOR=1.7; 95% CI=1.0–2.8), after adjusting for transportation costs
Peltzer *et al*. [119]	Cross-sectional study of 735 adults newly initiating ART at one of 3 public hospitals in KwaZulu-Natal, South Africa	2007–08	7-item version of the AIDS-Related Stigma Scale [120], modified to reflect internalized stigma; 7-item AIDS-related discrimination scale	ACTG Adherence Instrument [94] and 30-day visual analogue scale [121], with partial or full adherence defined as ≥95% adherence	Partial or full VAS adherence was associated with AIDS-related discrimination (AOR=0.60; 95% CI=0.46–0.78) but not internalized stigma (OR=1.11; 95% CI=0.97–1.27), after adjusting for alcohol use and social support; use of the ACTG Adherence Instrument yielded similar results
Penniman [122]	Secondary analysis of baseline data on 259 women enrolled in a larger cohort study in Los Angeles	2005–06	Disclosure of HIV serostatus to child	3-item self-reported dose-taking and timing adherence with two-day recall	Non-disclosure was associated with reduced odds of adherence (AOR=0.46; 95% CI=0.24–0.88), after adjusting for stress, family functioning and depression symptom severity
Peretti-Watel *et al*. [123]	Cross-sectional study of 2932 adults recruited from 102 hospitals in France	2003	Disclosure of HIV serostatus to friends and family; HIV-related discrimination by friends or family	Self-reported measure based on dose and timing adherence with one-week recall, with “high adherence” defined as no doses missed or mistimed	Poor adherence was associated with HIV-related discrimination (AOR=1.68; 95% CI=1.00–2.82) but not selective disclosure to significant others (AOR=0.73; 95% CI=0.28–1.94), after adjustment for alcohol and drug use
Rao *et al*. [124]	Cross-sectional study of 720 outpatients from a university HIV clinic in Seattle	2009	Summated rating scale of 4 items related to internalized and enacted stigma, from the 24-item Stigma Scale for Chronic Illness [125]	3 items from the ACTG Adherence Instrument [94], a one-item rating response measure [126] and a 30-day VAS [121]	In a structural equation model, stigma was associated with reduced adherence (*b*=–0.21, *p*<0.01); the authors concluded that the effect was mediated by depression symptom severity
Rintamaki *et al*. [127]	Cross-sectional study of 204 outpatients at two urban academic medical centre clinics in Illinois and Louisiana	2001	Summated rating scale of 3 items from the Patient Medication Adherence Questionnaire (PMAQ) [128, 129] related to internalized stigma and disclosure concerns	Non-adherence defined as any missed doses in the prior four days, assessed using the PMAQ	High stigma was associated with greater odds of non-adherence (AOR=3.3; 95% CI=1.4–8.1), after adjusting for race & education
Rotheram-Borus *et al*. [130]	Secondary analysis of baseline data from a randomized controlled trial of 409 adults recruited from 4 district hospitals in northern Thailand	2009^a^	7-item summative rating scale assessing extent of HIV serostatus disclosure to social network ties	Self-reported lifetime adherence, with good adherence defined as never having missed a dose	Disclosure had a statistically significant association with adherence (*b*=0.11, *p*<0.05); the authors concluded that disclosure operates primarily through its effect on family functioning
Rougemont *et al*. [131]	Longitudinal study of 312 treatment-naïve adults initiating ART in Yaoundé, Cameroun	2006–07	Disclosure of HIV serostatus to family	Pharmacy refill, with “non-adherers” defined as “renewal of prescriptions of later than two weeks”	Non-disclosure did not have a statistically significant association with non-adherence (AOR=0.98; 95% CI=0.81–1.18), after adjustment for income, education and distance to clinic
Sayles *et al*. [132]	Cross-sectional study of 202 adults recruited from 5 community organizations and 2 HIV clinic sites in Los Angeles	2007	28-item internalized stigma scale [133]	Seven-day self-reported ART adherence as measured on a 5-point Likert scale [110], with suboptimal adherence as defined as any response other than “all of the time”	A high level of internalized stigma was not associated with suboptimal adherence (AOR=2.09; 95% CI=0.81–5.39), after adjusting for mental health, race, education, income, insurance and years since diagnosis
Spire *et al*. [134]	Longitudinal study of 445 treatment-naïve adults initiating ART, recruited from 47 hospitals across France	1997	Disclosure of HIV serostatus to a family member	Self-reported adherence over prior four days, with “adherent” defined as 100% adherence	71% of participants who had disclosed to a family member at baseline were classified as adherent four months later, compared to 76% of those who had not disclosed (*p*=0.26)
Stirratt *et al*. [135]	Cross-sectional study of 215 adults recruited from 2 outpatient HIV clinics in New York City	2000–04	Disclosure of HIV serostatus to up to 15 family members and 15 personal contacts [136]	14-day ART adherence as measured by electronic event monitoring	Percentage of informed family members had a statistically significant association with ART adherence (*b*=0.21, *p*<0.05)
					after adjusting for self-efficacy, motivation and outcome expectancies
Sumari-de Boer *et al*. [137]	Cross-sectional study of 201 outpatients at an academic medical centre HIV clinic in Amsterdam, the Netherlands	2008–09	Personalized stigma and disclosure concerns sub-scales of the HIV stigma scale [81]	30-day pharmacy refill adherence, with non-adherence defined as<100% adherence	Non-adherence had a statistically significant association with disclosure concerns (AOR=1.1; 95% CI=1.01–1.2) but not personalized stigma (AOR not reported), after adjusting for years since diagnosis, quality of life and depression symptom severity
Van Dyk [138]	Cross-sectional study of 439 adults recruited from public health HIV clinics and hospitals in Pretoria, South Africa	2008	Disclosure of HIV serostatus to partner	30-day self-reported adherence as elicited through a visual assessment scale [121], with optimum adherence defined as >90% adherence	41% of participants who had disclosed to partners reported optimum adherence, compared to 21% of participants who had not disclosed (*p*=0.006)
Vanable *et al*. [139]	Cross sectional study of 221 outpatients in central New York state	2001	Five-item frequency of stigma-related experiences scale	Summary self-reported adherence measure averaged across 4 items based on a seven-day recall period	Stigma-related experiences had a negative association with self-reported adherence (*b*=−0.20, *p*<0.01), after adjusting for income, employment status and time since diagnosis
Waite *et al*. [140]	Cross-sectional study of 204 outpatients at two urban academic medical centre clinics in Illinois and Louisiana	2001	Summated rating scale of 3 items from the Patient Medication Adherence Questionnaire (PMAQ) [128, 129] related to internalized stigma and disclosure concerns	Non-adherence defined as any missed doses in the prior four days, assessed using a modified version of the PMAQ	A high level of stigma was associated with increased odds of non-adherence (AOR=3.1; 95% CI=1.3–7.7), after adjusting for insurance coverage, employment, mental disorder and history of alcohol or drug treatment
Wang *et al*. [141]	Cross-sectional study of 308 adults recruited from seven treatment sites in China	2006	Disclosure of HIV serostatus	Seven-day self-reported adherence, with good adherence defined as>90% of doses taken	Disclosure did not have a statistically significant association with adherence (estimates not shown)
Watt [142]	Cross sectional study of 340 persons in Tanzania	2007^a^	10-item perceived stigma scale [143], and number of social network ties to whom the participant had disclosed his or her seropositivity	Self-reported missed doses in the prior four days [94], and 30-day self-reported adherence using a modified visual analogue scale [121], with optimal adherence defined as≥95% adherence on both instruments	On univariable analysis, neither stigma nor disclosure had statistically significant associations with optimal adherence (estimates not shown)
Weiser *et al*. [144]	Cross-sectional study of 109 persons recruited from three private clinics in Botswana	2000	Disclosure of HIV serostatus	12-month self-reported adherence [94], with good adherence defined as≥95% of doses taken	On univariable analysis, disclosure did not have a statistically significant association with good adherence (OR=3.55; 95% CI=0.91–13.92)
Wolitski *et al*. [145]	Cross-sectional study of 637 homeless or unstably housed persons in three U.S. cities	2004	Modified 6-item internalized and 6-item perceived HIV stigma scales [81]	Self-reported missed doses in the prior two days and seven days	Perceived stigma, but not internalized stigma, was associated with increased odds of missed doses in the past two days (AOR=1.40; 95% CI=1.00–1.95) and past seven days (AOR=1.41; 95% CI=1.05–1.89), after adjusting for housing status, education, and years since HIV diagnosis

a Refers to date of publication, as dates of data collection were not clearly described.

Among the 41 studies, 25 (61%) reported a positive finding (i.e., showing that stigma was associated with reduced ART adherence or that disclosure was associated with improved adherence) while 16 (39%) reported a null finding. No studies reported that better ART adherence was paradoxically associated with greater intensity of stigma or less disclosure. A roughly equal proportion of studies conducted outside of the United States reported a positive finding compared to US-based studies (16/26 (62%) vs. 9/15 (60%); Pearson's *χ* ^2^=0.01, *p*=0.92).

When the studies were disaggregated by study design, most of the cross-sectional studies (24/34 (71%)) reported a positive finding, while most of the longitudinal studies (6/7 (86%)) reported a null finding (Pearson's *χ* ^2^=7.7; *p*=0.005). When disaggregated by exposure, these differences were slightly attenuated. Among studies examining the impact of a stigma variable on adherence, 15/20 (75%) cross-sectional studies vs. 1/3 (33%) longitudinal studies reported a positive finding (Pearson's *χ* ^2^=2.14; *p*=0.14). Among studies examining the impact of disclosure on adherence, 11/17 (65%) cross-sectional studies vs. 0/4 (0%) longitudinal studies reported a positive finding (Pearson's *χ* ^2^=5.4; *p*=0.02).

In three cross-sectional studies, the authors fit structural equation models to investigate the relationships between study variables. Diiorio *et al*. [92] concluded that the association between stigma and ART adherence was mediated by self-efficacy: perceived stigma eroded one's confidence about adhering to a treatment regimen, which in turn undermined treatment adherence. Rao *et al*. [124] did not measure self-efficacy but concluded that internalized stigma worsened symptoms of depression, like fatigue and concentration difficulties, which in turn compromised one's ability to adhere to a complex treatment regimen. In the study by Rotheram-Borus *et al*. [130], disclosure had a statistically significant association with ART adherence; the authors concluded that the effect was mediated principally by improvements in family function.

## Conceptual model

To integrate our core findings from the qualitative and quantitative studies, we propose a conceptual model described in Figure 3, citing areas of congruence between our empirically derived themes and theoretical frameworks previously published by others. In our model, structural and economic barriers associated with poverty undermine ART adherence. Enacted stigma undermines ART adherence through psychological processes specific to HIV-positive persons as well as through general psychological processes that are common to HIV-positive and HIV-negative persons alike. Stigma and poverty have mutually reinforcing relationships with each other, particularly in resource-limited settings [146]: stigma and social isolation have adverse economic impacts and, conversely, poverty worsens stigma by highlighting the economic aspects of HIV's perceived association with premature morbidity and mortality.

**Figure 3:** Conceptual model. This figure summarizes the findings of our meta-synthesis of 34 qualitative studies and analysis of 41 quantitative studies. The stigma of HIV was found to compromise ART adherence through general as well as group-specific psychological processes. Adaptive coping and social support were critical determinants of participants’ ability to overcome structural and economic barriers associated with poverty to successfully adhere to ART.

Internalized stigma may result when HIV-positive persons accept as valid the stigmatizing beliefs of the majority group. Because HIV infection is a potentially concealable stigma, HIV-positive persons may attempt to delay disclosure until disease progression renders further concealment impossible [147]. As elaborated in the stress process model [148, 149] and as described by the participants in the studies summarized in this review, HIV-positive persons draw on adaptive coping and social support to minimize the harmful effects of life stressors.

Adaptive coping and social support partially moderate the harmful effects of poverty on adherence and are represented in the diagram as effect modifiers: in the presence of adaptive coping or strong social support networks, the negative impacts of poverty on adherence are reduced. In this regard our synthesis is consistent with the social support model described by Ware *et al*. [150], who found that HIV-positive persons in Nigeria, Tanzania and Uganda relied heavily on social support to overcome structural and economic barriers to care. The authors concluded that the stigma of HIV was feared specifically because it weakened relationships that proved to be critical for everyday survival. In addition, as supported by both the qualitative and the quantitative studies summarized in this review, these general and group-specific psychological processes can directly benefit or undermine ART adherence. For example, in the setting of enacted stigma, many HIV-positive participants adopted strategies of concealment, which led directly to treatment interruptions.

The qualitative studies we identified also suggested a number of extensions to the model, namely that certain factors can moderate the severity of enacted stigma and their ultimate impacts on ART adherence. One such factor is the health system, which can be configured to support patients and minimize the harmful influences of stigma on ART adherence. Although resistance to stigma has been described [151], in countries with fragile healthcare systems resistance to stigma can be weakened as HIV-positive persons struggle with the anxieties of uncertain and unstable access to treatment [80]. Another factor involves social norms, which were described by participants in the qualitative studies as potentially intensifying the harmful influences of stigma. HIV-positive persons who belonged to sexual minority groups or who had acquired HIV through socially unacceptable means, in particular, experienced greater stigma because their self-identities and behaviours were defined by the majority as being inconsistent with social norms.

## Discussion

In this systematic review of both qualitative and quantitative studies conducted among 26,715 HIV-positive persons living in 32 countries worldwide, we found that HIV-related stigma compromised ART adherence, primarily by undermining social support and adaptive coping. Our analysis is consistent with prior work demonstrating the importance of social ties in promoting adherence, particularly in resource-limited settings [33, 152], and reflects the centrality of social integration to the experience of HIV-positive persons engaged in treatment. These themes are all the more prominent in settings of extreme poverty where treatment barriers are highly prevalent [8, 14, 153] and where social ties may be essential for survival [72, 154, 155]. Our findings have implications for public health strategies now being explored in high-HIV prevalence regions, such as universal voluntary testing with immediate treatment [36]. The evidence search protocol was not designed to identify studies examining the influences of stigma on HIV testing [156, 157], pre-ART linkage to care [158, 159], ART refusal [160], or other treatment- and care-related behaviours along the entire continuum of engagement in care [35]. However, HIV-related stigma has been shown to adversely affect these treatment- and care-related behaviours in a wide range of settings [35, 161–166]. Optimization of the entire continuum of care is needed to maximize the public health impact of test-and-treat [34], thereby underscoring the importance of our findings.

Several limitations are important to consider when assessing this systematic review. First, it is well known that qualitative studies can be difficult to locate using conventional search strategies [167]. Although we adopted a purposefully broad search protocol that involved the full text review of 960 journal articles, unpublished dissertations and conference abstracts, we cannot exclude the possibility that we may have missed some relevant studies. Second, and related to the previous, we only identified one (qualitative) study from the UNAIDS Eastern Europe and Central Asia region. The HIV epidemic follows a different pattern in these countries, with concentrated epidemics most notably driven by injection drug use but also by prison overcrowding and unprotected sexual intercourse among men who have sex with men and sex workers [168–170]. For people belonging to these already marginalized subgroups, the stigma of their HIV serostatus is layered upon these pre-existing inequalities, thereby displacing them further downward in the status hierarchy. If we had been able to identify more studies from this region, it is possible that different themes could have been identified in the qualitative synthesis or that an even stronger association between stigma and ART adherence would have been described. Third, heterogeneity in the types of exposures and outcomes used in the quantitative studies precluded a formal meta-analysis. The vote counting-styled procedures we employed to synthesize their findings could not generate effect size estimates, are characterized by low statistical power [171] and cannot assess the magnitude of the purported relationship. As the field converges on the use of standardized and validated measures of stigma, disclosure and adherence, we expect that the methods of meta-analysis can be increasingly applied. Fourth, a greater proportion of longitudinal studies reported a null association between ART adherence and either stigma or disclosure. The difference appeared to be driven by studies examining the impact of disclosure on adherence. The single longitudinal study that documented a positive finding employed validated instruments to measure both stigma and self-reported ART adherence, but in general the relatively small number of longitudinal studies limited our ability to draw strong conclusions. Fifth, the majority of studies included in this review were assessed to be at risk of bias. A key reporting deficiency in the qualitative studies was lack of detail on the method of analysis. The majority of quantitative studies did not use validated exposure and outcome measures. Although these factors could exert unpredictable biases, we acknowledge they could have biased the qualitative and quantitative findings towards the null, with attendant effects on our conceptual model.

These caveats aside, the conceptual model that emerged from our synthesis of the literature has several important implications for programming and policy. At the individual level, interventions focused on enhancing social support by activating [172] or strengthening existing ties [173, 174], or facilitating either of these through the encouragement of serostatus disclosure [175–177], may be expected to improve ART adherence. These behaviours may in turn yield health and mental health dividends. Although our meta-synthesis highlighted positive self-identity as an important factor related to greater adherence, more research is needed to understand the conditions under which HIV-related outcomes are better than expected despite the experiences of HIV- and stigma-related adversity (which can be thought of as being related to the concept of resilience [178–180]). It should be acknowledged here that social ties are not uniformly beneficial. This was observed in our data showing that all relationships were not necessarily described as supportive and that some study participants’ experiences suggested positive benefits from concealment. There have been few intervention studies where disclosure was emphasized as a primary outcome [181], but the outcomes of HIV serostatus disclosure are not unambiguously positive. Due to HIV-related stigma, significant others may react in negative ways after learning about a loved one's seropositivity [182–184]. In order to avoid these undesirable outcomes, interventions targeting disclosure behaviours should be sensitive to these potential negative consequences.

At the structural level, our model suggests that structural interventions (which target the context in which people live, including social ties, resources and institutions [185]) to enhance the capacity of health systems for providing quality care may help to minimize the adverse effects of HIV-related stigma on ART adherence. Structural interventions that strengthen the livelihoods of HIV-positive persons may also be a promising avenue for subverting HIV-related stigma, particularly in resource-limited settings where contributing to local solidarity networks is a core social function [186] and where the economic impacts of HIV and AIDS have exacerbated both the instrumental and symbolic aspects of stigma attached to HIV [187]. Castro and Farmer [188] advanced the argument that “structural violence determines, in large part, who suffers from AIDS-related stigma and discrimination” (p. 55). Although some observers have speculated that economic strengthening or livelihood interventions may play a role in reducing HIV-related stigma [146], to our knowledge these hypotheses have not been formally tested [189, 190]. Related work suggests that these may spark a “virtuous” cycle: as stigma-related barriers are levelled and as HIV testing, treatment and other care-related behaviours become more widespread, the stigma of HIV and AIDS can be reduced [188, 191–195].

Notably, our conceptual model also suggests several promising points of intervention to improve ART adherence that *have not* consistently yielded benefits when tested for their impacts on ART adherence. For example, several studies described how effective treatment of depression could potentially improve treatment adherence, consistent with the positive prevention model elaborated by Sikkema *et al*. [196]. However, depression intervention studies have yielded mixed findings to date with regards to HIV treatment adherence outcomes [197–199]. Likewise social support interventions should also be expected to improve adherence, but these have also proved inconclusive [200–203]. The lack of consistent findings may potentially be explained by the fact that interventions targeting intrapersonal or interpersonal processes fail to address the larger social forces undermining adherence to HIV treatment. We emphasize here that the concepts embedded in our conceptual model span multiple levels of analysis [204, 205], ranging from intrapersonal processes (self-identity, coping), to interpersonal processes (social support, concealment), to structural factors (health systems, poverty, stigma). We therefore expect that interventions spanning multiple levels would yield the greatest impacts on reducing stigma [206], but these approaches have been rarely employed.

## Conclusions

In this review of both qualitative and quantitative studies, we found that HIV-related stigma compromises ART adherence through general as well as group-specific psychological processes. Adaptive coping and social support were critical determinants of participants’ ability to overcome structural and economic barriers associated with poverty to successfully adhere to ART. Our conceptual model, which integrates the results of both quantitative and qualitative studies, suggests that the effects of stigma operate at multiple levels (intrapersonal, interpersonal and structural). Interventions to reduce stigma should target these multiple levels of influence in order to have maximum effectiveness on improving ART adherence.

## References

[1] Paterson DL, Swindells S, Mohr J, Brester M, Vergis EN, Squier C (2000). Adherence to protease inhibitor therapy and outcomes in patients with HIV infection. Ann Intern Med.

[2] Bangsberg DR, Hecht FM, Charlebois ED, Zolopa AR, Holodniy M, Sheiner L (2000). Adherence to protease inhibitors, HIV-1 viral load, and development of drug resistance in an indigent population. AIDS.

[3] Bangsberg DR, Perry S, Charlebois ED, Clark RA, Roberston M, Zolopa AR (2001). Non-adherence to highly active antiretroviral therapy predicts progression to AIDS. AIDS.

[4] Singh N, Squier C, Sivek C, Wagener M, Nguyen MH, Yu VL (1996). Determinants of compliance with antiretroviral therapy in patients with human immunodeficiency virus: prospective assessment with implications for enhancing compliance. AIDS Care.

[5] Mehta S, Moore RD, Graham NM (1997). Potential factors affecting adherence with HIV therapy. AIDS.

[6] Maggiolo F, Ripamonti D, Arici C, Gregis G, Quinzan G, Camacho GA (2002). Simpler regimens may enhance adherence to antiretrovirals in HIV-infected patients. HIV Clin Trials.

[7] Au JT, Kayitenkore K, Shutes E, Karita E, Peters PJ, Tichacek A (2006). Access to adequate nutrition is a major potential obstacle to antiretroviral adherence among HIV-infected individuals in Rwanda. AIDS.

[8] Weiser SD, Tuller DM, Frongillo EA, Senkungu J, Mukiibi N, Bangsberg DR (2010). Food insecurity as a barrier to sustained antiretroviral therapy adherence in Uganda. PLoS One.

[9] Kalofonos IA (2010). “All I eat is ARVs”: the paradox of AIDS treatment interventions in central Mozambique. Med Anthropol Q.

[10] Nagata JM, Magerenge RO, Young SL, Oguta JO, Weiser SD, Cohen CR (2012). Social determinants, lived experiences, and consequences of household food insecurity among persons living with HIV/AIDS on the shore of Lake Victoria, Kenya. AIDS Care.

[11] Weiser SD, Palar K, Frongillo EA, Tsai AC, Kumbakumba E, dePee S Longitudinal assessment of associations between food insecurity, antiretroviral adherence and HIV treatment outcomes in rural Uganda. AIDS.

[12] Musumari PM, Feldman MD, Techasrivichien T, Wouters E, Ono-Kihara M, Kihara M (2013). “If I have nothing to eat, I get angry and push the pills bottle away from me”: A qualitative study of patient determinants of adherence to antiretroviral therapy in the Democratic Republic of Congo. AIDS Care.

[13] Hardon AP, Akurut D, Comoro C, Ekezie C, Irunde HF, Gerrits T (2007). Hunger, waiting time and transport costs: time to confront challenges to ART adherence in Africa. AIDS Care.

[14] Tuller DM, Bangsberg DR, Senkungu J, Ware NC, Emenyonu N, Weiser SD (2010). Transportation costs impede sustained adherence and access to HAART in a clinic population in southwestern Uganda: a qualitative study. AIDS Behav.

[15] Taiwo BO, Idoko JA, Welty LJ, Otoh I, Job G, Iyaji PG (2010). Assessing the viorologic and adherence benefits of patient-selected HIV treatment partners in a resource-limited setting. J Acquir Immune Defic Syndr.

[16] Pyne-Mercier LD, John-Stewart GC, Richardson BA, Kagondu NL, Thiga J, Noshy H (2011). The consequences of post-election violence on antiretroviral HIV therapy in Kenya. AIDS Care.

[17] Siedner MJ, Lankowski A, Tsai AC, Muzoora C, Martin JN, Hunt PW (2013). GPS-measured distance to clinic, but not self-reported transportation factors, are associated with missed HIV clinic visits in rural Uganda. AIDS.

[18] Smith R, Rossetto K, Peterson BL (2008). A meta-analysis of disclosure of one's HIV-positive status, stigma and social support. AIDS Care.

[19] Steward WT, Herek GM, Ramakrishna J, Bharat S, Chandy S, Wrubel J (2008). HIV-related stigma: adapting a theoretical framework for use in India. Soc Sci Med.

[20] Tsai AC, Bangsberg DR, Kegeles SM, Katz IT, Haberer JE, Muzoora C Internalized stigma, social distance, and disclosure of HIV seropositivity in rural Uganda. Ann Behav Med.

[21] Logie C, Gadalla TM (2009). Meta-analysis of health and demographic correlates of stigma towards people living with HIV. AIDS Care.

[22] Mak WW, Poon CY, Pun LY, Cheung SF (2007). Meta-analysis of stigma and mental health. Soc Sci Med.

[23] Goffman E (1963). Stigma: notes on the management of spoiled identity.

[24] Jones EE, Farina A, Hastorf AH, Markus H, Miller DT, Scott RA (1984). Social stigma: the psychology of marked relationships.

[25] Scheff TJ (1966). Being mentally ill: a sociological theory.

[26] Scambler G, Hopkins A (1986). Being epileptic: coming to terms with stigma. Sociol Health Illn.

[27] Link BG, Cullen FT, Struening E, Shrout PE (1989). A modified labeling theory approach to mental disorders: an empirical assessment. Am Sociol Rev.

[28] Devine PG, Plant EA, Harrison K (1999). The problem of ‘us’ versus ‘them’ and AIDS stigma. Am Behav Sci.

[29] Allport GW (1954). The nature of prejudice.

[30] Pennebaker JW, Berkowitz L (1989). Confession, inhibition, and disease. Advances in experimental social psychology.

[31] Simbayi LC, Kalichman S, Strebel A, Cloete A, Henda N, Mqeketo A (2007). Internalized stigma, discrimination, and depression among men and women living with HIV/AIDS in Cape Town, South Africa. Soc Sci Med.

[32] Tsai AC, Bangsberg DR, Frongillo EA, Hunt PW, Muzoora C, Martin JN (2012). Food insecurity, depression and the modifying role of social support among people living with HIV/AIDS in rural Uganda. Soc Sci Med.

[33] Tsai AC, Bangsberg DR (2011). The importance of social ties in sustaining medication adherence in resource-limited settings. J Gen Intern Med.

[34] Gardner EM, McLees MP, Steiner JF, Del Rio C, Burman WJ (2011). The spectrum of engagement in HIV care and its relevance to test-and-treat strategies for prevention of HIV infection. Clin Infect Dis.

[35] Kranzer K, Govindasamy D, Ford N, Johnston V, Lawn SD (2012). Quantifying and addressing losses along the continuum of care for people living with HIV infection in sub-Saharan Africa: a systematic review. J Int AIDS Soc.

[36] Granich RM, Gilks CF, Dye C, De Cock KM, Williams BG (2009). Universal voluntary HIV testing with immediate antiretroviral therapy as a strategy for elimination of HIV transmission: a mathematical model. Lancet.

[37] Jurgens R, Cohen J, Tarantola D, Heywood M, Carr R (2009). Universal voluntary HIV testing and immediate antiretroviral therapy. Lancet.

[38] Dixon-Woods M, Fitzpatrick R (2001). Qualitative research in systematic reviews. Has established a place for itself. BMJ.

[39] National CASP Collaboration for Qualitative Methodologies (2006). 10 questions to help you make sense of qualitative research.

[40] Atkins S, Lewin S, Smith H, Engel M, Fretheim A, Volmink J (2008). Conducting a meta-ethnography of qualitative literature: lessons learnt. BMC Med Res Methodol.

[41] Tong A, Sainsbury P, Craig J (2007). Consolidated criteria for reporting qualitative research (COREQ): a 32-item checklist for interviews and focus groups. Int J Qual Health Care.

[42] Sanderson S, Tatt ID, Higgins JP (2007). Tools for assessing quality and susceptibility to bias in observational studies in epidemiology: a systematic review and annotated bibliography. Int J Epidemiol.

[43] Noblit GW, Hare RD (1988). Meta-ethnography: synthesizing qualitative studies.

[44] Martin Hilber A, Kenter E, Redmond S, Merten S, Bagnol B, Low N (2012). Vaginal practices as women's agency in sub-Saharan Africa: a synthesis of meaning and motivation through meta-ethnography. Soc Sci Med.

[45] Smith LK, Pope C, Botha JL (2005). Patients’ help-seeking experiences and delay in cancer presentation: a qualitative synthesis. Lancet.

[46] Munro SA, Lewin SA, Smith HJ, Engel ME, Fretheim A, Volmink J (2007). Patient adherence to tuberculosis treatment: a systematic review of qualitative research. PLoS Med.

[47] Guest G, Bunce A, Johnson L (2006). How many interviews are enough? An experiment with data saturation and variability. Field Method.

[48] Kumarasamy N, Safren SA, Raminani SR, Pickard R, James R, Krishnan AK (2005). Barriers and facilitators to antiretroviral medication adherence among patients with HIV in Chennai, India: a qualitative study. AIDS Patient Care STDs.

[49] Edwards LV (2006). Perceived social support and HIV/AIDS medication adherence among African American women. Qual Health Res.

[50] Nachega JB, Knowlton AR, Deluca A, Schoeman JH, Watkinson L, Efron A (2006). Treatment supporter to improve adherence to antiretroviral therapy in HIV-infected South African adults. A qualitative study. J Acquir Immune Defic Syndr.

[51] Ware NC, Wyatt MA, Tugenberg T (2006). Social relationships, stigma and adherence to antiretroviral therapy for HIV/AIDS. AIDS Care.

[52] Skhosana NL, Struthers H, Gray GE, McIntyre JA (2006). HIV disclosure and other factors that impact on adherence to antiretroviral therapy: the case of Soweto, South Africa. Afr J AIDS Res.

[53] Melchior R, Nemes MI, Alencar TM, Buchalla CM (2007). Desafios da adesão ao tratamento de pessoas vivendo com HIV/AIDS no Brasil. Rev Saúde Pública.

[54] Tarakeshwar N, Srikrishnan AK, Johnson S, Vasu C, Solomon S, Merson M (2007). A social cognitive model of health for HIV-positive adults receiving care in India. AIDS Behav.

[55] Konkle-Parker DJ, Erlen JA, Dubbert PM (2008). Barriers and facilitators to medication adherence in a southern minority population with HIV disease. J Assoc Nurses AIDS Care.

[56] Nam SL, Fielding K, Avalos A, Dickinson D, Gaolathe T, Geissler PW (2008). The relationship of acceptance or denial of HIV-status to antiretroviral adherence among adult HIV patients in urban Botswana. Soc Sci Med.

[57] Byakika-Tusiime J, Crane J, Oyugi JH, Ragland K, Kawuma A, Musoke P (2009). Longitudinal antiretroviral adherence in HIV+ Ugandan parents and their children initiating HAART in the MTCT-Plus family treatment model: role of depression in declining adherence over time. AIDS Behav.

[58] Watt MH, Maman S, Earp JA, Eng E, Setel PW, Golin CE (2009). “It's all the time in my mind”: facilitators of adherence to antiretroviral therapy in a Tanzanian setting. Soc Sci Med.

[59] Kalanzi DJN (2009). Adherence behavior and the impact of HAART on quality of life of Ugandan adults [Ph.D. dissertation].

[60] Ruanjahn G, Roberts D, Monterosso L (2010). An exploration of factors influencing adherence to highly active anti-retroviral therapy (HAART) among people living with HIV/AIDS in Northern Thailand. AIDS Care.

[61] Badahdah AM, Pedersen DE (2011). “I want to stand on my own legs”: a qualitative study of antiretroviral therapy adherence among HIV-positive women in Egypt. AIDS Care.

[62] Gusdal AK, Obua C, Andualem T, Wahlstrom R, Chalker J, Fochsen G (2011). Peer counselors’ role in supporting patients’ adherence to ART in Ethiopia and Uganda. AIDS Care.

[63] Van Tam V, Pharris A, Thorson A, Alfven T, Larsson M (2011). “It is not that I forget, it's just that I don't want other people to know”: barriers to and strategies for adherence to antiretroviral therapy among HIV patients in Northern Vietnam. AIDS Care.

[64] Daftary A, Padayatchi N (2012). Social constraints to TB/HIV healthcare: accounts from coinfected patients in South Africa. AIDS Care.

[65] Matovu SN, La cour K, Hemmingsson H (2012). Narratives of Ugandan women adhering to HIV/AIDS medication. Occup Ther Int.

[66] Nyanzi-Wakholi B, Lara AM, Munderi P, Gilks C (2012). The charms and challenges of antiretroviral therapy in Uganda: the DART experience. AIDS Care.

[67] O'Laughlin KN, Wyatt MA, Kaaya S, Bangsberg DR, Ware NC (2012). How treatment partners help: social analysis of an African adherence support intervention. AIDS Behav.

[68] Wasti SP, Simkhada P, Randall J, Freeman JV, van Teijlingen E (2012). Factors influencing adherence to antiretroviral treatment in Nepal: a mixed-methods study. PLoS One.

[69] Okoror TA, Falade CO, Olorunlana A, Walker EM, Okareh OT (2013). Exploring the cultural context of HIV stigma on antiretroviral therapy adherence among people living with HIV/AIDS in southwest Nigeria. AIDS Patient Care STDs.

[70] Portelli MS, Tenni B, Kounnavong S, Chanthivilay P Barriers to and facilitators of adherence to antiretroviral therapy among people living with HIV in Lao PDR: a qualitative study. Asia Pac J Public Health.

[71] Murray LK, Semrau K, McCurley E, Thea DM, Scott N, Mwiya M (2009). Barriers to acceptance and adherence of antiretroviral therapy in urban Zambian women: a qualitative study. AIDS Care.

[72] Izugbara CO, Wekesa E (2011). Beliefs and practices about antiretroviral medication: a study of poor urban Kenyans living with HIV/AIDS. Sociol Health Illn.

[73] Brion JM, Menke EM (2008). Perspectives regarding adherence to prescribed treatment in highly adherent HIV-infected gay men. J Assoc Nurses AIDS Care.

[74] Mohammadpour A, Yekta ZP, Nikbakht Nasrabadi AR (2010). HIV-infected patients’ adherence to highly active antiretroviral therapy: a phenomenological study. Nurs Health Sci.

[75] Bontempi JM, Burleson L, Lopez MH (2004). HIV medication adherence programs: the importance of social support. J Community Health Nurs.

[76] Mouala C, Roux P, Okome M, Sentenac S, Okome F, Nziengui U (2006). Bilan de quelques études sur l'observance aux ARV en Afrique. Med Trop (Mars).

[77] Sabin LL, Desilva MB, Hamer DH, Keyi X, Yue Y, Wen F (2008). Barriers to adherence to antiretroviral medications among patients living with HIV in southern China: a qualitative study. AIDS Care.

[78] Dahab M, Charalambous S, Hamilton R, Fielding K, Kielmann K, Churchyard GJ (2008). “That is why I stopped the ART”: patients’ & providers’ perspectives on barriers to and enablers of HIV treatment adherence in a South African workplace programme. BMC Public Health.

[79] Gilbert L, Walker L (2010). ‘My biggest fear was that people would reject me once they knew my status …’: stigma as experienced by patients in an HIV/AIDS clinic in Johannesburg, South Africa. Health Soc Care Community.

[80] Bernays S, Rhodes T (2009). Experiencing uncertain HIV treatment delivery in a transitional setting: qualitative study. AIDS Care.

[81] Berger BE, Ferrans CE, Lashley FR (2001). Measuring stigma in people with HIV: psychometric assessment of the HIV stigma scale. Res Nurs Health.

[82] Birbeck GL, Chomba E, Kvalsund M, Bradbury R, Mang'ombe C, Malama K (2009). Antiretroviral adherence in rural Zambia: the first year of treatment availability. Am J Trop Med Hyg.

[83] Adeyemi A, Olubunmi F, Oluseyi A (2010). Predictors of adherence for patients on highly active antiretroviral therapy in HIV treatment program.

[84] Boyer S, Clerc I, Bonono C-R, Marcellin F, Bile P-C, Ventelou B (2011). Non-adherence to antiretroviral treatment and unplanned treatment interruption among people living with HIV/AIDS in Cameroon: individual and healthcare supply-related factors. Soc Sci Med.

[85] Carrieri P, Cailleton V, Le Moing V, Spire B, Dellamonica P, Bouvet E (2001). The dynamic of adherence to highly active antiretroviral therapy: results from the French National APROCO cohort. J Acquir Immune Defic Syndr.

[86] Cardarelli R, Weis S, Adams E, Radaford D, Vecino I, Munguia G (2008). General health status and adherence to antiretroviral therapy. J Int Assoc Physicians AIDS Care (Chic).

[87] Morisky DE, Green LW, Levine DM (1986). Concurrent and predictive validity of a self-reported measure of medication adherence. Med Care.

[88] Knobel H, Alonso J, Casado JL, Collazos J, Gonzalez J, Ruiz I (2002). Validation of a simplified medication adherence questionnaire in a large cohort of HIV-infected patients: the GEEMA Study. AIDS.

[89] Carlucci JG, Kamanga A, Sheneberger R, Shepherd BE, Jenkins CA, Spurrier J (2008). Predictors of adherence to antiretroviral therapy in rural Zambia. J Acquir Immune Defic Syndr.

[90] Charurat M, Oyegunle M, Benjamin R, Habib A, Eze E, Ele P (2010). Patient retention and adherence to antiretrovirals in a large antiretroviral therapy program in Nigeria: a longitudinal analysis for risk factors. PLoS One.

[91] Colbert AM (2007). Functional health literacy, medication-taking self-efficacy and HIV medication adherence [Ph.D. dissertation].

[92] Diiorio C, McCarty F, Depadilla L, Resnicow K, Holstad MM, Yeager K (2009). Adherence to antiretroviral medication regimens: a test of a psychosocial model. AIDS Behav.

[93] Pequegnat W, Bauman LJ, Bray JH, DiClemente R, DiIorio C, Hoppe SK (2001). Measurement of the role of families in prevention and adaptation to HIV/AIDS. AIDS Behav.

[94] Chesney MA, Ickovics JR, Chambers DB, Gifford AL, Neidig J, Zwickl B (2000). Self-reported adherence to antiretroviral medications among participants in HIV clinical trials: the AACTG adherence instruments. Patient Care Committee & Adherence Working Group of the Outcomes Committee of the Adult AIDS Clinical Trials Group (AACTG). AIDS Care.

[95] Dlamini PS, Wantland D, Makoae LN, Chirwa M, Kohi TW, Greeff M (2009). HIV stigma and missed medications in HIV-positive people in five African countries. AIDS Patient Care STDs.

[96] Holzemer WL, Uys LR, Chirwa ML, Greeff M, Makoae LN, Kohi TW (2007). Validation of the HIV/AIDS stigma instrument – PLWA (HASI-P). AIDS Care.

[97] Do NT, Phiri K, Bussmann H, Gaolathe T, Marlink RG, Wester CW (2010). Psychosocial factors affecting medication adherence among HIV-1 infected adults receiving combination antiretroviral therapy (cART) in Botswana. AIDS Res Hum Retroviruses.

[98] Franke MF, Murray MB, Muñoz M, Hernandez-Diaz S, Sebastian JL, Atwood S (2011). Food insufficiency is a risk factor for suboptimal antiretroviral therapy adherence among HIV-infected adults in urban Peru. AIDS Behav.

[99] Goldman JD, Cantrell RA, Mulenga LB, Tambatamba BC, Reid SE, Levy JW (2008). Simple adherence assessments to predict virologic failure among HIV-infected adults with discordant immunologic and clinical responses to antiretroviral therapy. AIDS Res Hum Retroviruses.

[100] Kalichman SC, Cain D, Fuhrel A, Eaton L, Di Fonzo K, Ertl T (2005). Assessing medication adherence self-efficacy among low-literacy patients: development of a pictographic visual analogue scale. Health Educ Res.

[101] Gifford AL, Lorig K, Chesney M, Laurent D, Gonzalez V (1996). Patient education to improve health-related quality of life in HIV/AIDS: a pilot study. 11th International Conference on AIDS.

[102] Kalichman SC, Pope H, White D, Cherry C, Amaral CM, Swetzes C (2008). Association between health literacy and HIV treatment adherence: further evidence from objectively measured medication adherence. J Int Assoc Physicians AIDS Care (Chic).

[103] Kalichman SC, Simbayi LC, Cloete A, Mthembu PP, Mkhonta RN, Ginindza T (2009). Measuring AIDS stigmas in people living with HIV/AIDS: the Internalized AIDS-Related Stigma Scale. AIDS Care.

[104] Li L, Lee SJ, Wen Y, Lin C, Wan D, Jiraphongsa C (2010). Antiretroviral therapy adherence among patients living with HIV/AIDS in Thailand. Nurs Health Sci.

[105] Lee SJ, Li L, Jiraphongsa C, Iamsirithaworn S, Khumtong S, Rotheram-Borus MJ (2010). Regional variations in HIV disclosure in Thailand: implications for future interventions. Int J STD AIDS.

[106] Herek GM, Capitanio JP (1993). Public reactions to AIDS in the United States: a second decade of stigma. Am J Public Health.

[107] Apinundecha C, Laohasiriwong W, Cameron MP, Lim S (2007). A community participation intervention to reduce HIV/AIDS stigma, Nakhon Ratchasima province, northeast Thailand. AIDS Care.

[108] Li X, Huang L, Wang H, Fennie KP, He G, Williams AB (2011). Stigma mediates the relationship between self-efficacy, medication adherence, and quality of life among people living with HIV/AIDS in China. AIDS Patient Care STDs.

[109] Li X, He G, Wang H, Huang L, Liu L (2010). Development and evaluation of HIV/AIDS-related stigma and discrimination scale. Chin J Nurs.

[110] Mannheimer S, Friedland G, Matts J, Child C, Chesney M (2002). The consistency of adherence to antiretroviral therapy predicts biologic outcomes for human immunodeficiency virus-infected persons in clinical trials. Clin Infect Dis.

[111] Lucero AF, Smith C, Ufford LJ, Leipzig RM (2001). Poor adherence to HAART in older adults with HIV infection. J Am Geriatr Soc.

[112] Martinez J, Harper G, Carleton RA, Hosek S, Bojan K, Glum G (2012). The impact of stigma on medication adherence among HIV-positive adolescent and young adult females and the moderating effects of coping and satisfaction with health care. AIDS Patient Care STDs.

[113] Mo PK, Mak WW (2009). Intentionality of medication non-adherence among individuals living with HIV/AIDS in Hong Kong. AIDS Care.

[114] Mak WW, Cheung RY, Law RW, Woo J, Li PC, Chung RW (2007). Examining attribution model of self-stigma on social support and psychological well-being among people with HIV+/AIDS. Soc Sci Med.

[115] Molassiotis A, Nahas-Lopez V, Chung WY, Lam SW, Li CK, Lau TF (2002). Factors associated with adherence to antiretroviral medication in HIV-infected patients. Int J STD AIDS.

[116] Muyingo SK, Walker AS, Reid A, Munderi P, Gibb DM, Ssali F (2008). Patterns of individual and population-level adherence to antiretroviral therapy and risk factors for poor adherence in the first year of the DART trial in Uganda and Zimbabwe. J Acquir Immune Defic Syndr.

[117] Nachega JB, Stein DM, Lehman DA, Hlatshwayo D, Mothopeng R, Chaisson RE (2004). Adherence to antiretroviral therapy in HIV-infected adults in Soweto, South Africa. AIDS Res Hum Retroviruses.

[118] Olowookere SA, Fatiregun AA, Akinyemi JO, Bamgboye AE, Osagbemi GK (2008). Prevalence and determinants of nonadherence to highly active antiretroviral therapy among people living with HIV/AIDS in Ibadan, Nigeria. J Infect Dev Countr.

[119] Peltzer K, Friend-du Preez N, Ramlagan S, Anderson J (2010). Antiretroviral treatment adherence among HIV patients in KwaZulu-Natal, South Africa. BMC Public Health.

[120] Kalichman SC, Simbayi LC, Jooste S, Toefy Y, Cain D, Cherry C (2005). Development of a brief scale to measure AIDS-related stigma in South Africa. AIDS Behav.

[121] Walsh JC, Mandalia S, Gazzard BG (2002). Responses to a 1 month self-report on adherence to antiretroviral therapy are consistent with electronic data and virological treatment outcome. AIDS.

[122] Penniman TV (2010). The impact of family functioning on depression and medication adherence among mothers infected with HIV.

[123] Peretti-Watel P, Spire B, Pierret J, Lert F, Obadia Y (2006). Management of HIV-related stigma and adherence to HAART: evidence from a large representative sample of outpatients attending French hospitals (ANRS-EN12-VESPA 2003). AIDS Care.

[124] Rao D, Feldman BJ, Fredericksen RJ, Crane PK, Simoni JM, Kitahata MM (2012). A structural equation model of HIV-related stigma, depressive symptoms, and medication adherence. AIDS Behav.

[125] Rao D, Choi SW, Victorson D, Bode R, Peterman A, Heinemann A (2009). Measuring stigma across neurological conditions: the development of the stigma scale for chronic illness (SSCI). Qual Life Res.

[126] Lu M, Safren SA, Skolnik PR, Rogers WH, Coady W, Hardy H (2008). Optimal recall period and response task for self-reported HIV medication adherence. AIDS Behav.

[127] Rintamaki LS, Davis TC, Skripkauskas S, Bennett CL, Wolf MS (2006). Social stigma concerns and HIV medication adherence. AIDS Patient Care STDs.

[128] DeMasi R, Tolson J, Pham S, Capuano G, Graham N, Fisher R (1999). Self-reported adherence to HAART and correlation with HIV RNA: initial results with the patient medication adherence questionnaire.

[129] DeMasi RA, Graham NM, Tolson JM, Pham SV, Capuano GA, Fisher RL (2001). Correlation between self-reported adherence to highly active antiretroviral therapy (HAART) and virologic outcome. Adv Ther.

[130] Rotheram-Borus MJ, Stein JA, Jiraphongsa C, Khumtong S, Lee SJ, Li L (2010). Benefits of family and social relationships for Thai parents living with HIV. Prev Sci.

[131] Rougemont M, Stoll BE, Elia N, Ngang P (2009). Antiretroviral treatment adherence and its determinants in Sub-Saharan Africa: a prospective study at Yaounde Central Hospital, Cameroon. AIDS Res Ther.

[132] Sayles JN, Wong MD, Kinsler JJ, Martins D, Cunningham WE (2009). The association of stigma with self-reported access to medical care and antiretroviral therapy adherence in persons living with HIV/AIDS. J Gen Intern Med.

[133] Sayles JN, Hays RD, Sarkisian CA, Mahajan AP, Spritzer KL, Cunningham WE (2008). Development and psychometric assessment of a multidimensional measure of internalized HIV stigma in a sample of HIV-positive adults. AIDS Behav.

[134] Spire B, Duran S, Souville M, Leport C, Raffi F, Moatti JP (2002). Adherence to highly active antiretroviral therapies (HAART) in HIV-infected patients: from a predictive to a dynamic approach. Soc Sci Med.

[135] Stirratt MJ, Remien RH, Smith A, Copeland OQ, Dolezal C, Krieger D (2006). The role of HIV serostatus disclosure in antiretroviral medication adherence. AIDS Behav.

[136] el-Bassel N, Cooper DK, Chen DR, Schilling RF (1998). Personal social networks and HIV status among women on methadone. AIDS Care.

[137] Sumari-de Boer IM, Sprangers MA, Prins JM, Nieuwkerk PT (2012). HIV stigma and depressive symptoms are related to adherence and virological response to antiretroviral treatment among immigrant and indigenous HIV infected patients. AIDS Behav.

[138] Van Dyk AC (2010). Treatment adherence following national antiretroviral rollout in South Africa. Afr J AIDS Res.

[139] Vanable PA, Carey MP, Blair DC, Littlewood RA (2006). Impact of HIV-related stigma on health behaviors and psychological adjustment among HIV-positive men and women. AIDS Behav.

[140] Waite KR, Paasche-Orlow M, Rintamaki LS, Davis TC, Wolf MS (2008). Literacy, social stigma, and HIV medication adherence. J Gen Intern Med.

[141] Wang H, He G, Li X, Yang A, Chen X, Fennie KP (2008). Self-reported adherence to antiretroviral treatment among HIV-infected people in Central China. AIDS Patient Care STDs.

[142] Watt MH (2008). Understanding patients' adherence to antiretroviral therapy: a mixed-methods study in Arusha, Tanzania.

[143] Mbwambo J, Kilonzo G, Kopoka P, Nyblade L (2003). Understanding HIV-related stigma in Tanzania.

[144] Weiser S, Wolfe W, Bangsberg D, Thior I, Gilbert P, Makhema J (2003). Barriers to antiretroviral adherence for patients living with HIV infection and AIDS in Botswana. J Acquir Immune Defic Syndr.

[145] Wolitski RJ, Pals SL, Kidder DP, Courtenay-Quirk C, Holtgrave DR (2009). The effects of HIV stigma on health, disclosure of HIV status, and risk behavior of homeless and unstably housed persons living with HIV. AIDS Behav.

[146] Tsai AC, Bangsberg DR, Weiser SD Harnessing poverty alleviation to subvert the stigma of HIV in sub-Saharan Africa. PLoS Med.

[147] Alonzo AA, Reynolds NR (1995). Stigma, HIV and AIDS: an exploration and elaboration of a stigma trajectory. Soc Sci Med.

[148] Pearlin LI, Lieberman MA, Menaghan EG, Mullan JT (1981). The stress process. J Health Soc Behav.

[149] Pearlin LI (1989). The sociological study of stress. J Health Soc Behav.

[150] Ware NC, Idoko J, Kaaya S, Biraro IA, Wyatt MA, Agbaji O (2009). Explaining adherence success in sub-Saharan Africa: an ethnographic study. PLoS Med.

[151] Abrahams N, Jewkes R (2012). Managing and resisting stigma: a qualitative study among people living with HIV in South Africa. J Int AIDS Soc.

[152] Bangsberg DR, Deeks SG (2010). Spending more to save more: interventions to promote adherence. Ann Intern Med.

[153] Tsai AC, Bangsberg DR, Emenyonu N, Senkungu JK, Martin JN, Weiser SD (2011). The social context of food insecurity among persons living with HIV/AIDS in rural Uganda. Soc Sci Med.

[154] Samuels FA, Rutenberg N (2011). “Health regains but livelihoods lag”: findings from a study with people on ART in Zambia and Kenya. AIDS Care.

[155] Gausset Q, Mogensen HO, Yameogo WM, Berthe A, Konate B (2012). The ambivalence of stigma and the double-edged sword of HIV/AIDS intervention in Burkina Faso. Soc Sci Med.

[156] Wong LH, Rooyen HV, Modiba P, Richter L, Gray G, McIntyre JA (2009). Test and tell: correlates and consequences of testing and disclosure of HIV status in South Africa (HPTN 043 Project Accept). J Acquir Immune Defic Syndr.

[157] Young SD, Hlavka Z, Modiba P, Gray G, Van Rooyen H, Richter L (2010). HIV-related stigma, social norms, and HIV testing in Soweto and Vulindlela, South Africa: National Institutes of Mental Health Project Accept (HPTN 043). J Acquir Immune Defic Syndr.

[158] Bassett IV, Wang B, Chetty S, Mazibuko M, Bearnot B, Giddy J (2009). Loss to care and death before antiretroviral therapy in Durban, South Africa. J Acquir Immune Defic Syndr.

[159] Losina E, Bassett IV, Giddy J, Chetty S, Regan S, Walensky RP (2010). The “ART” of linkage: pre-treatment loss to care after HIV diagnosis at two PEPFAR sites in Durban, South Africa. PLoS One.

[160] Katz IT, Essien T, Marinda ET, Gray GE, Bangsberg DR, Martinson NA (2011). Antiretroviral therapy refusal among newly diagnosed HIV-infected adults. AIDS.

[161] Weiser SD, Heisler M, Leiter K, Percy-de Korte F, Tlou S, DeMonner S (2006). Routine HIV testing in Botswana: a population-based study on attitudes, practices, and human rights concerns. PLoS Med.

[162] Wolfe WR, Weiser SD, Bangsberg DR, Thior I, Makhema JM, Dickinson DB (2006). Effects of HIV-related stigma among an early sample of patients receiving antiretroviral therapy in Botswana. AIDS Care.

[163] Geng EH, Nash D, Kambugu A, Zhang Y, Braitstein P, Christopoulos KA (2010). Retention in care among HIV-infected patients in resource-limited settings: emerging insights and new directions. Curr HIV/AIDS Rep.

[164] Musheke M, Bond V, Merten S (2012). Individual and contextual factors influencing patient attrition from antiretroviral therapy care in an urban community of Lusaka, Zambia. J Int AIDS Soc.

[165] Bogart LM, Chetty S, Giddy J, Sypek A, Sticklor L, Walensky RP (2013). Barriers to care among people living with HIV in South Africa: contrasts between patient and healthcare provider perspectives. AIDS Care.

[166] Musheke M, Ntalasha H, Gari S, McKenzie O, Bond V, Martin-Hilber A (2013). A systematic review of qualitative findings on factors enabling and deterring uptake of HIV testing in sub-Saharan Africa. BMC Pub Health.

[167] Wong SS, Wilczynski NL, Haynes RB (2004). Developing optimal search strategies for detecting clinically relevant qualitative studies in MEDLINE. Stud Health Technol Inform.

[168] Bridge J, Lazarus JV, Atun R (2010). HIV epidemics and prevention responses in Asia and Eastern Europe: lessons to be learned? AIDS.

[169] Thorne C, Ferencic N, Malyuta R, Mimica J, Niemiec T (2010). Central Asia: hotspot in the worldwide HIV epidemic. Lancet Infect Dis.

[170] Jolley E, Rhodes T, Platt L, Hope V, Latypov A, Donoghoe M (2012). HIV among people who inject drugs in Central and Eastern Europe and Central Asia: a systematic review with implications for policy. BMJ Open.

[171] Hedges LV, Olkin I (1980). Vote-counting methods in research synthesis. Psychol Bull.

[172] Lin N (1999). Building a network theory of social capital. Connect (Tor).

[173] The ENRICHD Investigators (2001). Enhancing Recovery in Coronary Heart Disease (ENRICHD) study intervention: rationale and design. Psychosom Med.

[174] Berkman LF, Blumenthal J, Burg M, Carney RM, Catellier D, Cowan MJ (2003). Effects of treating depression and low perceived social support on clinical events after myocardial infarction: the Enhancing Recovery in Coronary Heart Disease Patients (ENRICHD) Randomized Trial. JAMA.

[175] Holt R, Court P, Vedhara K, Nott KH, Holmes J, Snow MH (1998). The role of disclosure in coping with HIV infection. AIDS Care.

[176] Paxton S (2002). The paradox of public HIV disclosure. AIDS Care.

[177] Kalichman SC, DiMarco M, Austin J, Luke W, DiFonzo K (2003). Stress, social support, and HIV-status disclosure to family and friends among HIV-positive men and women. J Behav Med.

[178] Garmezy N, Masten AS, Tellegen A (1984). The study of stress and competence in children: a building block for developmental psychopathology. Child Dev.

[179] Rutter M (1987). Psychosocial resilience and protective mechanisms. Am J Orthopsychiatry.

[180] Masten AS, Garmezy N, Tellegen A, Pellegrini DS, Larkin K, Larsen A (1988). Competence and stress in school children: the moderating effects of individual and family qualities. J Child Psychol Psychiatry.

[181] Wolitski RJ, Gomez CA, Parsons JT (2005). Effects of a peer-led behavioral intervention to reduce HIV transmission and promote serostatus disclosure among HIV-seropositive gay and bisexual men. AIDS.

[182] Simoni JM, Mason HR, Marks G, Ruiz MS, Reed D, Richardson JL (1995). Women's self-disclosure of HIV infection: rates, reasons, and reactions. J Consult Clin Psychol.

[183] Mansergh G, Marks G, Simoni JM (1995). Self-disclosure of HIV infection among men who vary in time since seropositive diagnosis and symptomatic status. AIDS.

[184] Medley A, Garcia-Moreno C, McGill S, Maman S (2004). Rates, barriers and outcomes of HIV serostatus disclosure among women in developing countries: implications for prevention of mother-to-child transmission programmes. Bull World Health Organ.

[185] Tsai AC (2012). A typology of structural approaches to HIV prevention: a commentary on Roberts and Matthews. Soc Sci Med.

[186] Seeley J, Russell S (2010). Social rebirth and social transformation? Rebuilding social lives after ART in rural Uganda. AIDS Care.

[187] Neuberg SL, Smith SM, Asther T, Heatherton TF, Kleck RE, Hebl MR, Hull JG (2000). Why people stigmatize: toward a biocultural framework. The social psychology of stigma.

[188] Castro A, Farmer P (2005). Understanding and addressing AIDS-related stigma: from anthropological theory to clinical practice in Haiti. Am J Public Health.

[189] Sengupta S, Banks B, Jonas D, Miles MS, Smith GC (2011). HIV interventions to reduce HIV/AIDS stigma: a systematic review. AIDS Behav.

[190] Brown L, Macintyre K, Trujillo L (2003). Interventions to reduce HIV/AIDS stigma: what have we learned?. AIDS Educ Prev.

[191] Farmer P, Leandre F, Mukherjee JS, Claude M, Nevil P, Smith-Fawzi MC (2001). Community-based approaches to HIV treatment in resource-poor settings. Lancet.

[192] Farmer P, Leandre F, Mukherjee J, Gupta R, Tarter L, Kim JY (2001). Community-based treatment of advanced HIV disease: introducing DOT-HAART (directly observed therapy with highly active antiretroviral therapy). Bull World Health Organ.

[193] Wolfe WR, Weiser SD, Leiter K, Steward WT, Percy-de Korte F, Phaladze N (2008). The impact of universal access to antiretroviral therapy on HIV stigma in Botswana. Am J Public Health.

[194] Baranov V, Bennett D, Kohler H-P (2012). The indirect impact of antiretroviral therapy.

[195] Tsai AC, Bangsberg DR, Bwana M, Haberer JE, Frongillo EA, Muzoora C How does antiretroviral treatment attenuate the stigma of HIV? Evidence from a cohort study in rural Uganda. AIDS Behav.

[196] Sikkema KJ, Watt MH, Drabkin AS, Meade CS, Hansen NB, Pence BW (2010). Mental health treatment to reduce HIV transmission risk behavior: a positive prevention model. AIDS Behav.

[197] Safren SA, O'Cleirigh C, Tan JY, Raminani SR, Reilly LC, Otto MW (2009). A randomized controlled trial of cognitive behavioral therapy for adherence and depression (CBT-AD) in HIV-infected individuals. Health Psychol.

[198] Tsai AC, Weiser SD, Petersen ML, Ragland K, Kushel MB, Bangsberg DR (2010). A marginal structural model to estimate the causal effect of antidepressant medication treatment on viral suppression among homeless and marginally housed persons with HIV. Arch Gen Psychiatry.

[199] Tsai AC, Karasic DH, Hammer GP, Charlebois ED, Ragland K, Moss AR (2013). Directly observed antidepressant medication treatment and HIV outcomes among homeless and marginally housed HIV-positive adults: a randomized controlled trial. Am J Public Health.

[200] Simoni JM, Huh D, Frick PA, Pearson CR, Andrasik MP, Dunbar PJ (2009). Peer support and pager messaging to promote antiretroviral modifying therapy in Seattle: a randomized controlled trial. J Acquir Immune Defic Syndr.

[201] Pearson CR, Micek MA, Simoni JM, Hoff PD, Matediana E, Martin DP (2007). Randomized control trial of peer-delivered, modified directly observed therapy for HAART in Mozambique. J Acquir Immune Defic Syndr.

[202] Simoni JM, Pantalone DW, Plummer MD, Huang B (2007). A randomized controlled trial of a peer support intervention targeting antiretroviral medication adherence and depressive symptomatology in HIV-positive men and women. Health Psychol.

[203] Decroo T, Telfer B, Biot M, Maikere J, Dezembro S, Cumba LI (2011). Distribution of antiretroviral treatment through self-forming groups of patients in Tete Province, Mozambique. J Acquir Immune Defic Syndr.

[204] Krieger N (2008). Proximal, distal, and the politics of causation: what's level got to do with it?. Am J Public Health.

[205] Krieger N (2012). Methods for the scientific study of discrimination and health: an ecosocial approach. Am J Public Health.

[206] Link BG, Phelan JC (2001). Conceptualizing stigma. Annu Rev Sociol.

# Sub article: A systematic review of interventions to reduce HIV-related stigma and discrimination from 2002 to 2013: how far have we come?

**DOI:** 10.7448/IAS.16.3.18734

**Published:** 2013-11-13

**Authors:** Anne L Stangl, Jennifer K Lloyd, Laura M Brady, Claire E Holland, Stefan Baral

**Affiliations:** 1 Department of Global Health, International Center for Research on Women, Washington, DC, USA; 2 Department of Epidemiology, Johns Hopkins School of Public Health, Baltimore, MD, USA

**Keywords:** systematic review, HIV, stigma reduction, discrimination reduction, interventions, measurement

## Abstract

**Introduction:**

HIV-related stigma and discrimination continue to hamper efforts to prevent new infections and engage people in HIV treatment, care and support programmes. The identification of effective interventions to reduce stigma and discrimination that can be integrated into national responses is crucial to the success of the global AIDS response.

**Methods:**

We conducted a systematic review of studies and reports that assessed the effectiveness of interventions to reduce HIV stigma and discrimination between 1 January 2002 and 1 March 2013. Databases searched for peer-reviewed articles included PubMed, Scopus, EBSCO Host –CINAHL Plus, Psycinfo, Ovid, Sociofile and Popline. Reports were obtained from the www.HIVAIDSClearinghouse.eu, USAID Development Experience Clearinghouse, UNESCO HIV and AIDS Education Clearinghouse, Google, WHO and UNAIDS. Ancestry searches for articles included in the systematic review were also conducted. Studies of any design that sought to reduce stigma as a primary or secondary objective and included pre- and post-intervention measures of stigma were included.

**Results:**

Of 2368 peer-reviewed articles and reports identified, 48 were included in our review representing 14 different target populations in 28 countries. The majority of interventions utilized two or more strategies to reduce stigma and discrimination, and ten included structural or biomedical components. However, most interventions targeted a single socio-ecological level and a single domain of stigma. Outcome measures lacked uniformity and validity, making both interpretation and comparison of study results difficult. While the majority of studies were effective at reducing the aspects of stigma they measured, none assessed the influence of stigma or discrimination reduction on HIV-related health outcomes.

**Conclusions:**

Our review revealed considerable progress in the stigma-reduction field. However, critical challenges and gaps remain which are impeding the identification of effective stigma-reduction strategies that can be implemented by national governments on a larger scale. The development, validation, and consistent use of globally relevant scales of stigma and discrimination are a critical next step for advancing the field of research in this area. Studies comparing the effectiveness of different stigma-reduction strategies and studies assessing the influence of stigma reduction on key behavioural and biomedical outcomes are also needed to maximize biomedical prevention efforts.

## Introduction

More than two decades into the HIV epidemic, stigma and discrimination continue to hamper efforts to prevent new infections and engage people in HIV treatment, care and support programmes. Numerous studies have linked HIV-related stigma with refusal of HIV testing, non-disclosure to partners and poor engagement in biomedical prevention approaches [1–6]. Similarly, internalized stigma, which refers to the negative consequences that result when people believe that stigmatizing public attitudes apply to them [7, 8], is a well-established barrier to medication adherence [9–13]. In response to this evidence, stigma reduction is now a key priority in PEPFAR's Blueprint for Achieving an AIDS-Free Generation [14] and UNAIDS’ HIV investment framework [15].

The recent shift in the global AIDS response to biomedical prevention will require acceptance and uptake of prevention approaches, such as voluntary medical male circumcision, pre-exposure prophylaxis and universal testing and treatment, at the population level [16, 17]. Effective interventions to reduce stigma and discrimination are crucial to the success of biomedical prevention [15, 18]. Such interventions need to be integrated into national responses and address the stigmatization process [19].

### Stigma conceptualizations and terminology

Stigma has been conceptualized from the perspective of both the individual and the society. According to Erving Goffman, stigma occurs when an attribute creates a deeply discrediting gap between who we think we are – our “actual social identity” – and how we are seen by others – our “virtual social identity” [20]. This gap creates a “spoiled identity” that cuts the stigmatized person “off from society and from himself, so that he stands as a discredited person against an unaccepting world” [20]. Building on Goffman's work, Link and Phelan described stigma as a harmful societal phenomenon – enabled by underlying social, political and economic powers – that begins when a difference is labelled, then is linked to negative stereotypes, leading to a separation of “us” from “them,” and finally to status loss and discrimination for those carrying the trait [21]. Deacon suggested that HIV-related discrimination be analyzed separately from stigma to explore the range of stigma-related disadvantages that may result from the stigmatization process, as well as positive responses such as resilience and activism [22].

The stigmatization process can be broken into specific domains, each of which can be addressed through programmatic and policy efforts [19, 23]. These domains are: drivers, facilitators, intersecting stigmas and manifestations of stigma [19]. Drivers are individual-level factors that negatively influence the stigmatization process such as: lack of awareness of stigma and its harmful consequences, fear of HIV infection through casual contact with people living with HIV (PLHIV), fear of economic ramifications or social breakdown due to HIV-positive family and community members, and prejudice and stereotypes towards PLHIV and key populations at highest risk of HIV infection [24–27]. Facilitators are societal-level factors that influence the stigmatization process either negatively or positively, including: protective or punitive laws, availability of grievance redressal systems, awareness of rights, structural barriers at the public policy level, cultural and gender norms, existence of social support for PLHIV, and power/powerlessness among PLHIV to resist and overcome the manifestations of stigma [19].

Drivers and facilitators combine to influence whether a stigma is applied to individuals or groups based on HIV status.

Intersecting, or layered, stigmas, refer to the multiple stigmas that people often face due to HIV status, gender, profession, migrancy, drug use, poverty, marital status, sexual and gender orientation [28–31]. Manifestations are the immediate results, mostly negative, of a stigma being applied to individuals or groups, including: anticipated stigma (fear of experiencing stigma if HIV status becomes known) [32], perceived stigma (perceptions about how PLHIV are treated in a given context) [33], internalized stigma [34], shame [35], experienced, or enacted, stigma (experiencing stigmatizing behaviours outside the purview of the law) [36, 37], discrimination (experiencing stigmatizing behaviours within the purview of the law) and resilience (ability to overcome threats to health and development after stigma is experienced) [18, 19]. Distinguishing between experienced stigma and experienced discrimination based on their legality informs the intervention strategies needed.

Individuals experience, internalize and/or perpetuate the manifestations of stigma [19]. Additionally, the social and structural environments in which individuals live and work influence the drivers and manifestations of stigma [38, 39], indicating the need for interventions that target multiple levels [40]. The socio-ecological framework [41], which recognizes that societal norms and structures influence individual attitudes and behaviours, identifies key levels at which stigma-reduction activities can be targeted: individual (knowledge, attitudes, skills), interpersonal (family, friends, social networks), organizational (organizations, social institutions, workplace), community (cultural values, norms, attitudes) and public policy (national and local laws and policies) [42].

### Previous reviews

Brown et al. [43] conducted the first global review of interventions to reduce HIV-related stigma in 2003. The authors articulated four intervention categories based on psychosocial conceptualizations of the stigmatization process that have remained applicable a decade later. The categories include:

1. information-based approaches (e.g., written information in a brochure),
2. skills building (e.g., participatory learning sessions to reduce negative attitudes),
3. counselling/support (e.g., support groups for PLHIV), and
4. contact with affected groups (e.g., interactions between PLHIV and the general public).

Most of the 22 studies reviewed attempted to increase the general public's tolerance or health providers’ willingness to treat PLHIV by changing individual-level fears, attitudes or behaviours. Two studies sought to improve coping strategies among PLHIV or key populations. The authors concluded that some stigma-reduction interventions appeared to work in the short term, but that more research was needed to understand the effectiveness of various intervention components, the scale and length of interventions required, and the gendered impacts [43].

The second review by Sengupta et al. in 2011 examined 19 HIV-prevention interventions that measured HIV stigma pre- and post-intervention, 11 of which had one or more components that directly targeted HIV stigma [44]. The review found that information, skills-building, counselling and PLHIV testimonials were associated with less stigmatizing attitudes among participants. The authors noted several gaps in the evidence base, including the poor quality of the majority of studies reviewed and the lack of standardized measurement [44].

### Current review

Our goal in the current systematic review was to obtain a more complete picture of the full range of intervention efforts and their effectiveness in interrupting the stigmatization process, minimizing negative manifestations of stigma and/or bolstering positive manifestations, such as resilience. An important distinction from previous reviews was the inclusion of search terms to capture discrimination-reduction interventions separately from stigma-reduction interventions. Another unique feature was the inclusion of structural and biomedical intervention categories.

Recent literature has focused on the role of structural and biomedical approaches in the prevention of HIV acquisition and transmission [45–48]. In the context of HIV-related stigma, structural approaches encompass activities aimed at removing, reducing or altering for the better structural factors that influence the stigmatization process, such as laws that criminalize HIV [49], hospital or workplace policies that institutionalize discrimination of PLHIV (e.g., labelling of beds, mandatory HIV testing prior to employment), or a lack of supplies to allow healthcare workers to practice universal precautions [40]. Structural approaches can also include efforts to ensure that grievance redressal systems and legal aid are available for PLHIV to seek justice if discriminated against [50, 51]. The emergence of structural interventions to reduce HIV-related stigma and discrimination is in direct response to the underlying power structures that enable the stigmatization process [21, 52]. The expansion of biomedical prevention approaches may influence HIV-related stigma, either positively, by normalizing HIV infection, or negatively, by leading to unwanted disclosure of sero-positive status and resulting discrimination [53, 54]. However, this relationship has yet to be explored quantitatively in the literature.

To identify interventions targeting HIV-related stigma and/or discrimination, we systematically reviewed peer-reviewed and grey literature. Our objectives were to document the stigma domains addressed, socio-ecological levels targeted, types of strategies employed to reduce stigma and discrimination, stigma-specific outcomes of these efforts and study quality.

## Methods

### Search strategy and selection criteria

This review followed PRISMA guidelines. Search terms included MESH or other associated terms for HIV cross-referenced with “stigma reduction” OR “discrimination reduction” (see Supplementary files). Databases for peer-reviewed articles included PubMed, Scopus, EBSCO Host – CINAHL Plus, Psycinfo, Ovid, Sociofile and Popline. Grey literature was obtained from the www.HIVAIDSClearinghouse.eu, USAID Development Experience Clearinghouse, UNESCO HIV and AIDS Education Clearinghouse, Google, WHO and UNAIDS. Ancestry searches of the 48 articles included in the review were also conducted.

Inclusion criteria included pre- and post-test data, clear descriptions of the intervention and sampling methods, and publication in English. We limited our search to articles published between 1 January 2002 and 1 March 2013 to exclude articles included in the Brown et al. review (2003) [43]. Studies of any design from any country that listed HIV stigma and/or discrimination reduction as a primary or secondary outcome were included. Studies were excluded if none of the intervention components aimed to reduce HIV stigma and/or discrimination. We did not exclude studies that lacked a clear description of the measures used or those that used non-validated measures, as historically these issues have been inconsistently addressed [36, 43, 44].

### Screening and data abstraction

Article citations were organized, uploaded and reviewed using the reference manager programme Endnote X5 from their respective databases. The title, author, journal and year of publication were then exported to an excel spreadsheet for title and abstract review. Articles were screened by two of three reviewers (JKL, LMB, CEH) to determine whether they included relevant information. If the article was deemed relevant by at least one reviewer, the abstract was retrieved. The same two reviewers screened the abstracts for relevant information. If at least one reviewer deemed the abstract relevant, or if the full text had to be obtained to determine if the abstract was relevant, the full text was reviewed. Discrepancies were discussed with a third senior reviewer (ALS) and consensus was reached as to whether or not to include the article. Data were abstracted using a standardized abstraction form (see Supplementary files). For studies that did not specify the validity or number of stigma measures used, the corresponding author was contacted. For measures coded as “not specified” (NS) in Table 1, we did not receive a response.

**Table 1:** Study and intervention characteristics, description of stigma measures and study findings from 48 studies

First author, publication date, country, study design^a^	Study population^b^	Sample	Intervention strategies^c^, intervention duration	Stigma domains^d^, socio-ecological levels^e^	Validated/un-validated, no. of items^f^	Results
Intervention strategy used						
Adam, 2011 [81], Canadian web-based, RXS	MSM	1942	I, 4 months	D, individual	Un-validated, 5 items	Stigma decreased
Al-Mazrou, 2005 [92], Saudi Arabia, QE/NC	Students (paramedical)	653	I, 1 year	D, individual	NS, NS	Stigma decreased
Bell, 2008 [72], South Africa, RCT	Students (primary), caregivers	557, 478	SB, 10 weekends, 90 minutes sessions	D, individual	Validated, NS	Stigma decreased
Esu-Williams, 2004 [77], Zambia, QE/C	Youth club members	60	SB, 3 years	D, individual	NS, NS	Stigma decreased
Li, 2011 [65], China, QE/C	Students (high school)	287	I, 8 sessions, 90 minutes	D, individual	Un-validated, 1 item	Stigma decreased
Maughan-Brown, 2010 [102], South Africa, RXS	Young adults	1074	B, 3 years	D, public policy	Un-validated, 8 items	Stigma increased
Nambiar, 2011 [69], India, QE/C	PLHIV	257	I, 14 days	M, individual	Un-validated, 36 items	Enacted stigma reduced. No change in felt or disclosure stigma
Neema, 2012 [99], Uganda, RXS	PLHIV	475	SB, 1 year, 6 months	F, organizational	NS, NS	Stigma decreased
Norr, 2012 [91], Chile, QE/C	HCWs	555	I, 8 sessions, 3 month F-U	D, individual	Un-validated, 7 items	Stigma decreased
Paxton, 2002 [66], Australia, QE/C	Students (secondary)	1397	C, 12 talks, 3 month F-U	D, individual	Validated, 15-item scale	Stigma decreased, but the impact was reduced after 3 months.
Sorcar, 2009 [67], India, QE/C	Students, (high school and college)	386	I, 3 stages, 1 year	D, individual	Un-validated, 17 items	Stigma decreased
Wang, 2009 [61], China, QE/NC	HCWs	69	SB, 10 days	D, individual	NS, NS	Stigma decreased
Intervention strategies used						
Bekele, 2008 [73], Ethiopia, QE/NC	Students, (high school)	373	I, SB, 8 hours	D, individual	Un-validated, 61 items	Stigma decreased
Biradavolu, 2012 [104], India, Pre- post- qualitative IDIs	FSW	55	ST, SB, 1 year, 5 months	D, M, organizational	N/A*	Stigma decreased
Boulay, 2008 [85], Ghana, RXS	Community members	5672	I, SB, 2 months	D, community	NS, 8 items	Stigma decreased
Brown, 2009 [74], South Africa, QE/C	Students, (university)	237	I, C, 3 weeks, 1 hour sessions	D, individual	Validated, 10-item scale	Stigma decreased
Deutsch, 2007 [82], USA, QE/C	Students (university)	77	I, SB, 2 sessions, 2 weeks	D, individual	Validated, 54-item scale	Stigma decreased
Denison, 2012 [79], Zambia, QE/C	Students (grade 8–9)	2133	I, SB, 1 month	D, organizational	Un-validated, 4 items	Stigma decreased
Ezedinachi, 2002 [87], Nigeria, RCT	HCWs	1552	I, SB, 30 workshops, 1 year F-U	D, individual	Un-validated, 14	Stigma decreased
Fakolade, 2010 [86], Nigeria, RXS	Community members	31,692	I, C, 4 years	D, community	NS, NS	Stigma decreased
Jurgensen, 2013 [80], Zambia, RCT	Community members	2607	CS, B, 2 years	D, public policy	Validated, 8-item scale	Stigma decreased in both intervention and control arm
Kaponda, 2009 [71], Malawi, QE/NC	HCWs	855	I, SB, 10, 90–120 minutes workshops	D, individual	NS, 2 items	Stigma decreased
Lau, 2005 [64], Hong Kong, QE/NC	Students, (grade 9–10)	1153	I, C, 2 weeks	D, individual	Un-validated, 19 items	Stigma decreased
Li, 2010 [60], China, RCT	Market workers	4510	I, SB, 2 years	D, community	Un-validated, 4 items	Stigma decreased
Norr, 2007 [76], Malawi, QE/NC	Teachers	328	I, SB, 6, 2-hour sessions	D, individual	Un-validated, 6 items	Stigma decreased
Richter, 2012 [103], Angola, Cameroon, Chad, Cote D'Ivoire, Equatorial Guinea, Kenya, Nigeria, QE/NC	Employees	993	I, SB, 15 sessions, 12–18 mos.	D, F, individual, organizational	Validated and un-validated, 11 items	Stigma decreased
Rimal, 2008 [70], Malawi, RXS	Community members	1771	I, C, 2 years	D, community	Un-validated, 14 items	Stigma decreased for those with high efficacy only; no change for those with low efficacy
Saad, 2012 [88], Nigeria, RCT	Students (university)	235	I, SB, 8-hour programme, 3 and 6-month F-U	D, community	Validated, 9-item scale	No change
Smith Fawzi, 2012 [89], Haiti, QE/NC	HIV+youth and their caregivers	168, 130	I, SB, 1 year	M, interpersonal	Validated, 22-item scale	Stigma decreased
Tshabalala, 2011 [100], South Africa, QE/C	PLHIV	20	I, SB, 8 sessions	D, M, individual	Validated, 16-item scale	Internalized stigma decreased. No change in enacted stigma
Williams, 2006 [62], China, QE/NC	HCWs	208	I, SB, 5-day workshop	D, individual	Validated, 34-item scale	Stigma decreased
Wu, 2008 [68], China, QE/C	HCWs	138	I, SB, 1, 4-hour session, 3 and 6-month F-U	D, individual	Un-validated, 3 items	Stigma decreased
Yiu, 2010 [68], Hong Kong, QE/NC	Students, (nursing)	89	I, C, 50-minute lecture, 6-week F-U	D, individual	Un-validated, 15 items	Stigma decreased
Young, 2010 [90], Peru, RCT	Community members	3049	I, SB, 24 months	D, community	Un-validated, 5 items	Stigma decreased for men, not for women
Intervention strategies used						
Apinundecha, 2007 [101], Thailand, QE/C	PLHIV, caregivers, and community leaders	425	SB, C, ST, 8 months	D, community	Un-validated, 30 items	Stigma decreased
Chao, 2010 [75], South Africa, QE/NC	Teachers	120	I, SB, C, CD or 2-day workshop	D, individual	Un-validated, 13 items	Stigma decreased
Gordon-Garofalo, 2004 [83], USA, QE/NC	Family members	28	I, SB, CS, 8 weeks, 2-month F-U	M, interpersonal	Un-validated, 3 items	Stigma decreased
Hosek, 2011 [84], USA, QE/NC	PLHIV	50	I, SB, CS, 12 sessions, 3 months	M, individual	Validated, 40-item scale	Stigma decreased
Lakshmi, 2013 [98], India, QE/C	PLHIV	120	I, SB, CS, 6, 60-minute sessions	M, individual	Validated, 40-item scale	Stigma decreased
Li, 2013 [94], China, RCT	HCWs	1760	I, SB, ST, 1 year, 2 months	D, F, individual, organizational	Un-validated, 30 items	Stigma decreased
Mall, 2013 [78], South Africa, RXS	Community members	1921	I, SB, B, 2 years	D, individual, public policy	Un-validated, NS	Stigma decreased
Nuwaha, 2012 [97], Uganda, RXS	Community members	1402	I, CS, B, 2-year period	D, M, individual, interpersonal, public policy	Validated, 3-item scale	Stigma decreased
Pisal, 2007 [59], India, QE/NC	HCWs	480	I, SB, C, 4 days	D, individual	NS, NS	Stigma decreased, with the exception of comfort cleaning up stool and urine of PLHIV
Uys, 2009 [95], Lesotho, Malawi, South Africa, Swaziland, Tanzania, QE/NC	Setting nurses, team Nurses, PLHIV	134, 43, 41	I, SB, C, 5 days	D, M, individual, organizational	Validated (HASI-P and HASI-N), 52-item scale	Perceived stigma decreased for PLHIV. No change in stigma for nurses
Intervention strategies used						
Gurnani, 2011 [58], India, Programme monitoring data	FSWs, government officials, Police, Journalists	60,000, 175, 13,500 950	SB, CS, C, ST, 4 years	D, M, individual, organizational	Un-validated, 2 items	Stigma decreased
Khuat Thi Hai, 2008 [93], Vietnam, QE/NC	HCWs	1592	I, SB, C, ST, 1-day workshop, 1.5-day training (Arm A), 2-day training (Arm B)	D, F, M, individual, organizational	NS, NS	Stigma decreased
Nyblade, 2008 [96], Vietnam, QE/NC	Community members	2,885	I, SB, C, ST, 1 year, 8 months	D, F, M, Community	Validated and un-validated, 21 items	Stigma decreased
Rao, 2012 [34], USA, QE/NC	PLHIV	24	I, SB, CS, C, 2 days	M, Individual	Validated, 14-item scale	Stigma decreased

a Study design abbreviations: RXS=repeated cross-sectional surveys; QE/NC=quasi-experimental with no control group; QE/C=quasi-experimental with a control group; RCT=randomized controlled trial

b Study population abbreviations: MSM=men who have sex with men; FSW=female sex workers; PLHIV=people living with HIV; HCWs=healthcare workers

c Intervention strategy abbreviations: I=information-based; SB=skills building; CS=counselling/support; C=contact; ST=structural; B=biomedical

d Stigma domain abbreviations: D=drivers; F=facilitators; M=manifestation

e Individual; interpersonal; organizational; community; and public policy

f NS=not specified

* *This study included qualitative data only

### Quality assessment

Two reviewers (JKL and CEH) assessed the quality of quantitative data from studies with randomized controlled trial (RCT), quasi-experimental or mixed-methods study designs (Table 2) using a modified Downs and Black checklist [55]. The checklist consisted of 26 items representing five sub-scales: reporting, external validity, bias, confounding and power [55]. Few of the 48 studies reported power calculations to determine if they had sufficient sample sizes to assess the effectiveness of their interventions. Therefore, we removed the power question (#27) from the standard checklist. The maximum score for the modified checklist was 26. Although the Downs and Black checklist does not have a pre-specified cutoff for acceptable studies, the mid-point score of 13 was used as a guideline to distinguish between low- and high-quality studies [56].

**Table 2:** Quality assessment of the 48 studies

First author, publication date	Study design^a^	Summary score for quality critique
Quantitative (modified Downs and Black, 1998)		
Al-Mazrou, 2005 [92]	QE/NC	62% (16/26)
Apinundecha, 2007 [101]	QE/C	62% (16/26)
Bekele, 2008 [73]	QE/NC	65% (17/26)
Bell, 2008 [72]	RCT	73% (19/26)
Boulay, 2008 [85]	RXS	73% (19/26)
Brown, 2009 [74]	QE/C	58% (15/26)
Chao, 2010 [75]	QE/NC	50% (13/26)
Denison, 2012 [79]	QE/C	50% (13/26)
Deutsch, 2007 [82]	QE/C	65% (17/26)
Esu-Williams, 2004 [77]	QE/C	46% (12/26)
Ezedinachi, 2002 [87]	RCT	58% (15/26)
Fakolade, 2010 [86]	RXS	62% (16/26)
Gordon-Garofalo, 2004 [83]	QE/C	54% (14/26)
Hosek, 2011 [84]	QE/NC	54% (14/26)
Jurgensen, 2013 [80]	RCT	73% (19/26)
Kaponda, 2009 [71]	QE/NC	46% (12/26)
Lakshmi, 2013 [98]	QE/C	50% (13/26)
Lau, 2005 [64]	QE/NC	58% (15/26)
Li, 2010 [60]	RCT	65% (17/26)
Li, 2011 [65]	QE/C	65% (17/26)
Li, 2013 [94]	RCT	73% (19/26)
Mall, 2013 [78]	RXS	58% (15/26)
Maughan-Brown, 2010 [102]	RXS	46% (12/26)
Nambiar, 2011 [69]	QE/C	54% (14/26)
Norr, 2007 [76]	QE/NC	50% (13/26)
Norr, 2012 [91]	QE/C	65% (17/26)
Nuwaha, 2012 [97]	RXS	69% (18/26)
Rao, 2012 [34]	QE/NC	58% (15/26)
Richter, 2012 [103]	QE/NC	46% (12/26)
Rimal, 2008 [70]	RXS	62% (16/26)
Saad, 2012 [88]	RCT	73% (19/26)
Wang, 2009 [61]	QE/NC	42% (11/26)
Williams, 2006 [62]	QE/NC	46% (12/26)
Wu, 2008 [63]	QE/C	62% (16/26)
Yiu, 2010 [68]	QE/NC	77% (20/26)
Young, 2010 [90]	RCT	65% (17/26)
Qualitative (Spencer et al. 2003)		
Biradavolu, 2012 [104]	Qualitative pre- post-	44% (8/18)
Mixed methods (Modified Downs and Black, 1998)		
Adam, 2011 [81]	QE/NC	50% (13/26)
Khuat Thi Hai, 2008 [93]	QE/C	58% (15/26)
Neema, 2012 [99]	RXS	42% (11/26)
Nyblade, 2008 [96]	QE/NC	54% (14/26)
Paxton, 2002 [66]	QE/C	62% (16/26)
Pisal, 2007 [59]	QE/NC	42% (11/26)
Smith Fawzi, 2012 [89]	QE/NC	54% (14/26)
Sorcar, 2009 [67]	QE/C	69% (18/26)
Tshabalala, 2011 [100]	QE/C	54% (14/26)
Uys, 2009 [95]	QE/NC	58% (15/26)
Other		
Gurnani, 2011 [58]	Monitoring data	n/a

a Study design abbreviations: RXS=repeated cross-sectional surveys; QE/NC=quasi-experimental with no control group; QE/C=quasi-experimental with a control group; RCT=randomized controlled trial.

N/a=this study could not be scored using either method as it lacked a research study design and used quantitative program monitoring data only to assess the intervention.

A guide for critically appraising qualitative research was used to appraise the qualitative study [57]. Quality was assessed with 18 items representing nine sub-scales: findings, design, sample, data collection, analysis, reporting, reflexivity and neutrality, ethics and auditability [57]. A score greater than 9, the mid-point for the Spencer guide, was considered high quality. We were unable to assess the quality of one study using either checklist, as the article presented programme monitoring data to assess the structural approach employed [58].

### Data synthesis

Due to the lack of standardized reporting of primary and secondary outcomes, a meta-analysis was not conducted. Instead, we categorized studies by their intervention strategies, and the stigma domains and socio-ecological levels targeted.

Four intervention categories originally described by Brown et al. (2003) were used, including:

1. information-based approaches,
2. skills building,
3. counselling/support, and
4. contact with affected groups.

We included two additional categories: structural approaches and biomedical, to capture new stigma-reduction strategies. Stigma domains assessed were: drivers, facilitators, intersecting stigmas and manifestations [19]. Socio-ecological levels assessed were: individual (knowledge, attitudes, skills); interpersonal (family, friends, social networks); organizational (organizations, social institutions, work place); community (cultural values, norms, attitudes); and public policy (national and local laws and policies) [42].

## Results

The search criteria identified 4032 potentially relevant articles and reports. After removing 927 duplicates and 737 articles published before 2002, 2096 peer-reviewed articles and 272 grey literature reports were included in the title review phase (Figure 1). A total of 48 (40 peer-reviewed articles, 6 grey literature reports and 2 dissertations) met the inclusion criteria and were included for further analysis.

**Figure 1:** Flowchart of search strategy.

### Study and intervention characteristics

The studies spanned a large geographical area. Eighteen studies were conducted in the Asia and Pacific region [59–69] and 17 were conducted in the East and Southern Africa [70–80]. Five studies were conducted in North America, Western and Central Europe [34, 81–84] and four were conducted in West and Central Africa [85–88]. Two studies were conducted in Latin America, one study in the Caribbean [89–91] and one study in the Middle East and North Africa [92]. No studies from Eastern Europe and Central Asia were identified. The most represented countries were South Africa (7 studies), China (6 studies), India (6 studies), Malawi (4 studies), and Nigeria (4 studies) (Table 1).

The interventions targeted a wide variety of populations. The most common target populations were students [64–68, 73, 74, 79, 82, 88, 92], healthcare workers [59, 61–63, 71, 87, 91, 93–95], community members [70, 78, 80, 85, 86, 90, 96, 97] and PLHIV [34, 69, 84, 95, 98–101]. Other target populations included youth [72, 77, 102], caregivers [72, 89, 101], teachers [75, 76], market workers [60], family members [83], employees [103] and journalists, police, and community leaders [58, 101]. Three interventions targeted key populations, including female sex workers (FSW) [58, 104] and men who have sex with men (MSM) [81] (Table 1).

Interventions typically included two or more approaches to reducing HIV-related stigma and discrimination. Forty-six percent used two approaches, 21% used three approaches and 8% used four approaches. However, 12 interventions (27%) employed a single approach (Figure 2a). Information-based approaches were the most common (38 studies), followed by skills-building (32 studies) and contact strategies (14 studies). Only seven studies included counselling/support, six employed structural approaches and four included a biomedical component. All of the studies with a structural component combined it with one or more other intervention strategies [58, 93, 94, 96, 101, 104]. For example, Li et al. combined information and skills building for healthcare workers with provision of universal precaution supplies at intervention hospitals in China [94] and Biradavolu et al. combined skills building and collectivization (into community-based organizations) of FSWs in India [104]. Three of the four studies with a biomedical component also combined it with one or more strategies [78, 80, 97]. For example, Jurgensen et al. and Nuwaha et al. combined community-wide availability of home-based HIV counselling and testing with counselling and support for PLHIV in Zambia [80] and counselling and support and information-based strategies in Uganda [97], respectively. One study assessed a biomedical approach, wider availability of antiretroviral therapy (ART) in South Africa, as a stand-alone stigma-reduction intervention [102] (Table 1).

**Figure 2:** Domains and levels targeted and approaches employed in the 48 studies.

Most studies (81%) targeted a single stigma domain. Thirty-two studies targeted drivers, one targeted facilitators [99] and six targeted manifestations of the stigmatization process [34, 69, 83, 84, 89, 98]. Only nine studies (19%) targeted multiple stigma domains: five targeted drivers and manifestations [58, 95, 97, 100, 104], two targeted drivers and facilitators [94, 103], and two targeted drivers, facilitators and manifestations [93, 96] (Figure 2b and Table 1). None of the interventions targeted intersecting stigmas.

Forty-one studies (85%) intervened at a single socio-ecological level. Individual-level interventions were the most common (27 studies), followed by community (7 studies), organizational (3 studies), interpersonal (2 studies) and public policy-level (2 studies) interventions. Seven studies targeted multiple levels. The most commonly combined levels were individual and organizational [58, 93–95, 103]. For example, the studies in healthcare settings tended to combine individual-level information provision and/or skills building with organizational-level activities, such as revising hospital policies and/or providing supplies for universal precautions [93–95]. One study by Mall et al. intervened at the individual and public policy levels [78], combining individual-level information and skills building with provision of ART mandated at the public policy level. Finally, the study by Nuwaha et al. targeted the individual, interpersonal and public policy levels [97] (Figure 2c and Table 1).

### Study design and measures

Only 7 of the 48 studies employed a randomized controlled study design [60, 72, 80, 87, 88, 90, 94]. The majority (65%) used quasi-experimental designs either with (13 studies) or without (18 studies) a control group. Another eight used repeated cross-sectional surveys [70, 78, 81, 85, 86, 97, 99, 102], one used programme monitoring data [58] and one used qualitative in-depth interviews collected pre- and post-intervention [104] (Table 1).

The measures used to assess stigma varied considerably across the 47 quantitative studies. Sixteen studies used validated measures, 22 studies (47%) used unvalidated measures or scales, and nine did not specify whether the measures used had been validated previously. Among the 36 studies that described the stigma measures used, only 12 measured the stigma domains that intervention activities were intended to shift. For example, several studies that targeted the drivers of stigma (e.g., fear, prejudice, stereotypes) measured only manifestations of stigma (e.g., agreement with discriminatory statements) [59, 61, 65, 70, 73, 78–80, 86, 91, 99]. The range of items used also differed substantially across studies, with one study using a single measure to assess stigma [65] and one using 61 items [73]. Only two of the seven RCTs reviewed used validated measures [72, 80] and the number of items ranged from 4 to 30 (Table 1).

### Study duration and outcomes

Intervention duration varied widely independent of intervention strategies employed. The shortest intervention tested was a single, 50-minute lecture for nursing students in Hong Kong that employed information-based contact strategies [68]. In contrast, an intervention in Nigeria used the same strategies, but these were implemented over four years [86].

The majority of studies reviewed (79%) reported statistically significant reductions in all stigma measures. Additionally, five studies observed reductions for some stigma measures but not others [59, 69, 70, 95, 100], one study reported reductions for men but not women [90], one reported reductions in both the treatment and control arms [80], and one reported no change in stigma [88]. Only one study in South Africa, which compared discriminatory attitudes reported by young adults in cross-sectional surveys administered before and after ART became widely available in the country, found a significant increase in stigma [102] (Table 1). The biomedical strategy was not combined with any other strategies (e.g., contact, skills building) that have previously demonstrated some effect at reducing stigma [43].

### Quality assessment

Forty-six studies employed quantitative methods and were assessed with the Downs and Black checklist. The average quality score was 15.4 with a median of 15.5. The scores ranged from 11 to 20. The qualitative study was assessed as “low quality” based on the Spencer et al. checklist [104]. Overall, we found the majority of studies to be of high quality, with only nine scoring in the low-quality range. Thirty-seven of the 45 studies (82%) that demonstrated significant reductions in some or all of the stigma measures assessed were considered “high-quality” studies. The study that observed an increase in stigma following the intervention was assessed as a “low-quality” study [102] (Table 2).

## Discussion

This systematic review revealed considerable progress in the stigma-reduction field over the last decade. Yet critical challenges and gaps remain which are impeding the identification of effective stigma and discrimination-reduction strategies that can be implemented by national governments on a larger scale.

### Progress in the field

The number, geography and complexity of interventions studied have expanded considerably. A very high percentage of studies that showed reductions in stigma were of high quality, which is a marked improvement from previous reviews [43, 44, 105]. There has been a substantial shift in the geography of stigma-reduction research. The interventions summarized in our review were conducted predominantly in low- and middle-income countries and targeted a much wider variety of populations. Only 5 of the 48 studies were conducted in the North America, Western and Central Europe region [34, 81–84]. The populations targeted with stigma and discrimination-reduction interventions have also expanded in the past decade. While students and healthcare workers continue to be heavily studied populations, studies among community members [70, 78, 80, 85, 86, 90, 96, 97] and PLHIV [34, 69, 84, 89, 98–100] are becoming more common.

Our review demonstrated that the socio-ecological levels targeted by stigma-reduction interventions have expanded over the past decade to include all five levels of influence. While individual-level interventions remained the most common, several community-level efforts have been tested [60, 70, 85, 86, 90, 96, 101] and a few interventions at the organizational-level have been studied [79, 99, 104]. In addition, interventions targeting multiple socio-ecological levels are beginning to emerge [58, 78, 93–95, 97, 103]. The stigma domains targeted have also expanded to include the facilitators [99] and manifestations of stigma [34, 69, 83, 84, 89, 98] as well as the drivers, sometimes in combination [58, 93–97, 100, 103, 107].

These findings are encouraging, given recent conceptualizations of the stigmatization process that highlight the importance of combining multiple intervention strategies to address multiple stigma domains across multiple socio-ecological levels [36, 52].

### Challenges and gaps

#### Intervention

Despite these improvements, most of the 48 studies targeted a single domain of stigma (drivers) and a single socio-ecological level (individual-level). While these studies provide important insights about potential strategies for improving the attitudes of a variety of individuals and groups (e.g., youth, healthcare workers, employees, students), they do not adequately address stigma manifestations, such as shame and discrimination, or community-level attitudes and social norms that shape individuals’ attitudes and behaviours. This finding calls into question the longer term utility of the interventions described for interrupting the stigmatization process.

Individual-level drivers of stigma, such as knowledge, fear and attitudes, are only part of the stigmatization process. Also critical to address are individual-level manifestations of stigma, such as the anticipation of experiencing stigma if positive or the perception that stigma towards PLHIV is high in a given community, which prevent people from testing for HIV or disclosing their HIV-positive status to a sexual partner or family member [1, 106]. Interventions that fail to address these concerns are unlikely to lead to increased and sustained health seeking behaviour or inspire the adoption of preventive behaviours, two of the key goals of stigma-reduction interventions.

Rigorous evaluations of multi-faceted interventions, designed to target the individual-level manifestations and drivers of stigma, are needed to inform the most efficacious and effective approaches for achieving longer term health outcomes. In addition, more research is needed to explore the individual and combinations of strategies that are most effective at improving community attitudes and creating an enabling environment for PLHIV and key populations to engage with healthcare and social support systems.

There are limited data assessing the influence of stigma-reduction interventions on key behavioural and biomedical outcomes, such as uptake of and retention on ART, drug regimens and feeding practices to prevent vertical transmission, and vertical transmission itself. While stigma is commonly cited as a barrier to prevention efforts [12, 53, 107], and many prevention trials have collected measures of stigma and discrimination [108], no fully powered RCT or quasi-experimental trial of HIV-prevention strategies or technologies have included stigma reduction as a key component of the intervention tested. Given emerging challenges with adherence to drug-based prevention among groups most at risk of HIV infection [109], such data are needed to inform appropriate national responses to the HIV epidemic.

Another gap is the absence of tested interventions aimed at supporting PLHIV to fulfil their human rights to care and dignity. Many countries have expanded existing laws or adopted new ones that protect PLHIV against discrimination [110]. However, for PLHIV to access their rights, they must be aware of the law and be able to access systems of redress against violations of those rights. Legal education and legal aid services are often needed to support PLHIV to access justice, and such services are recommended by UNAIDS as critical [49, 51]. Evaluation data are needed to inform the wider use of such approaches to support the positive advances that have been made in the public policy arena in many countries over the last decade.

Interventions specifically designed to reduce the intersecting stigmas that key populations often face were also absent from the literature. Such strategies will be important for maximizing the participation of key populations in biomedical prevention efforts such as universal HIV testing and treatment and topical and oral chemoprophylaxis with ART [16]. More information is needed on successful strategies to reduce intersecting stigmas in contexts where epidemics are concentrated in key populations, as well as where HIV epidemics among key populations are happening in the context of widespread generalized epidemics [111].

#### Methodology

Evaluating structural stigma-reduction interventions, particularly those targeted at the community level, poses a methodological challenge. Such interventions often involve multiple components occurring simultaneously at multiple levels, and thus are not necessarily conducive to the classic RCT design [112]. In addition, the social norm changes desired typically take longer to achieve than individual-level attitude changes [113]. Three of the studies evaluating interventions with a structural component in this review used quasi-experimental designs [93, 96, 114], one used pre- and post-in-depth interviews [104] and one reviewed programme monitoring data collected during the intervention period [58].

While these studies suggested some positive effects of structural approaches, causality is difficult to establish with these study designs in addition to the difficulties in attributing the relative effectiveness of structural approaches, as compared to the other components of the intervention. Additional research and the development of alternative or new evaluation methodologies such as propensity scores, causal inference and structural equation modelling are needed, particularly given the recent emphasis on addressing the structural causes of stigma and discrimination [115].

#### Measurement

Measurement issues continue to pose an important challenge to the field. The lack of standardized outcome measures for stigma and discrimination greatly limits our collective ability to determine which strategies work the best for addressing the various stigma domains or targeting different socio-ecological levels. While some validated scales have been developed for specific types of stigma, populations and contexts [116–120], few scales demonstrating validity in multiple contexts or across multiple populations are available [121, 122].

A priority moving forward must be the development of validated measures assessing each domain of the stigmatization process that can be shifted with programmatic efforts and/or structural interventions. An instrument similar to the MOS-HIV, which measures multiple domains of health-related quality of life, is validated for use in multiple countries and has standardized instructions for cultural adaptation [123], would greatly enhance the field of HIV stigma research. While some aspects of stigma may be culturally specific, key underlying constructs are common across contexts [24, 29], facilitating the development of standardized measurement tools. Such instruments are needed for assessing stigma and discrimination in the general population, among family and peers, among PLHIV and key populations and among healthcare workers [23, 24, 29]. The standardized survey for use in health facilities presented by Nyblade et al. in this supplement is an encouraging development. Similar efforts are now needed for other populations.

The discordance between the targeted domains of stigma and the measured domains of stigma is of concern. Across the studies reviewed, it was common for intervention activities to target drivers of stigma among individuals (e.g., fear of HIV infection through casual transmission) but only measure stigma manifestations (e.g., agreement with discriminatory statements like “teachers living with HIV should no longer be allowed to teach”) to assess intervention effectiveness [59, 61, 65, 70, 73, 78–80, 86, 91, 99]. This discordance adds another layer of uncertainty to the study findings. Let us take as an example an intervention that is successful at increasing awareness of stigma and its harmful consequences, but not at reducing fear of HIV infection through casual contact, which tends to drive avoidance behaviours. If the researcher only measures willingness to sit next to someone living with HIV and finds no significant change following intervention, she may mistakenly conclude that the intervention was not successful. The field would benefit considerably from evaluations that clearly link the stigma domains being targeted with the stigma domains measured [19]. The development of a uniform conceptualization of the stigmatization process, based on empirical evidence, could inform the development of both interventions and measurement tools.

### Limitations

There are several limitations with the approach used here. We were not able to explore the potential influence of stigma and discrimination-reduction efforts generated from and implemented by communities of PLHIV and key populations, which have been a hallmark of the HIV response in many countries, due to the lack of evaluation data on these approaches in the peer-reviewed and grey literature. Inclusion criteria limiting studies to those with pre- and post-intervention data excluded studies that only used post-intervention data to compare intervention and control groups. However, it was far more difficult to assess these studies’ quality thus limiting the utility of their inclusion for this review. Assessing study quality using the Downs and Black checklist was challenging due to the nature of most stigma-reduction interventions, precluding typical trial components such as blinding. Despite these challenges, the majority of studies reviewed were assessed as being of high quality.

A meta-analysis was not completed due to the significant heterogeneity of interventions and outcomes limiting the assessment of pooled effectiveness of interventions at reducing HIV-related stigma and discrimination. Generalizability of the findings of these interventions is limited as they have been tested only in specific sub-populations, such as students or healthcare workers. Assessment of causality of these interventions was also limited since more than half of the studies did not include a control group. Finally, some studies used un-validated scales or did not list the measurements used, which may lead to uncertainties in the reliability and validity of their measurements. Even with specific inclusion criteria and these limitations, this review draws strength from harnessing nearly 50 studies focused on the mitigation of HIV-related stigma and discrimination representing several types of interventions and populations.

## Conclusions

The field has come far in the last decade, though much remains to be done to enable the integration of proven stigma and discrimination-reduction strategies into national AIDS responses. Complex problems require complex solutions. The field of HIV-prevention research needs to embrace the importance of stigma in the HIV response, rather than shy away from it. The field must become bolder in the design and evaluation of interventions that target multiple stigma domains at multiple levels. Similarly, funding agencies should support the rigorous evaluation of multi-faceted stigma-reduction interventions, including interventions that assess the influence of stigma on behavioural and biomedical outcomes. Our collective ability to translate efficacious biomedical prevention approaches, such as ART as prevention [124–127], into effective ones at the population-level rests on whether we can remove the social and structural barriers to uptake and adherence. As such, addressing HIV-related stigma and discrimination should be at the core of the HIV response, not at the fringes. This priority should be represented in funding, policy, research and programming.

## References

[1] Abdool Karim Q, Meyer-Weitz A, Mboyi L, Carrara H, Mahlase G, Frohlich JA (2008). The influence of AIDS stigma and discrimination and social cohesion on HIV testing and willingness to disclose HIV in rural KwaZulu-Natal, South Africa. Glob Public Health..

[2] Brou H, Djohan G, Becquet R, Allou G, Ekouevi DK, Viho I (2007). When do HIV-infected women disclose their HIV status to their male partner and why? A study in a PMTCT programme, Abidjan. PLoS Med..

[3] Bwirire LD, Fitzgerald M, Zachariah R, Chikafa V, Massaquoi M, Moens M (2008). Reasons for loss to follow-up among mothers registered in a prevention-of-mother-to-child transmission program in rural Malawi. Trans Roy Soc Trop Med Hyg..

[4] Byakika-Tusiime J, Crane J, Oyugi JH, Ragland K, Kawuma A, Musoke P (2009). Longitudinal antiretroviral adherence in HIV+ Ugandan parents and their children initiating HAART in the MTCT-plus family treatment model: role of depression in declining adherence over time. AIDS Behav..

[5] Deribe K, Woldemichael K, Wondafrash M, Haile A, Amberbir A (2008). Disclosure experience and associated factors among HIV positive men and women clinical service users in southwest Ethiopia. BMC Public Health..

[6] Turan JM, Bukusi EA, Onono M, Holzemer WL, Miller S, Cohen CR (2011). HIV/AIDS stigma and refusal of HIV testing among pregnant women in rural Kenya: results from the MAMAS study. AIDS Behav..

[7] Corrigan PW, Penn DL (1999). Lessons from social psychology on discrediting psychiatric stigma. Am Psychol..

[8] Corrigan PW, Watson AC (2002). The paradox of self-stigma and mental illness. Clin Psychol: Sci Pract..

[9] Rintamaki LS, Davis TC, Skripkauskas S, Bennett CL, Wolf MS (2006). Social stigma concerns and HIV medication adherence. AIDS Patient Care STDs..

[10] Rao D, Feldman BJ, Fredericksen RJ, Crane PK, Simoni JM, Kitahata MM (2011). A structural equation model of HIV-related stigma, depressive symptoms, and medication adherence. AIDS Behav..

[11] Sabin LL, Desilva MB, Hamer DH, Keyi X, Yue Y, Wen F (2008). Barriers to adherence to antiretroviral medications among patients living with HIV in southern China: a qualitative study. AIDS Care..

[12] Dlamini PS, Wantland D, Makoae LN, Chirwa M, Kohi TW, Greeff M (2009). HIV stigma and missed medications in HIV-positive people in five African countries. AIDS Patient Care STDs..

[13] Johnson MO, Chesney MA, Neilands TB, Dilworth SE, Remien RH, Weinhardt LS (2009). Disparities in reported reasons for not initiating or stopping antiretroviral treatment among a diverse sample of persons living with HIV. J Gen Intern Med..

[14] PEPFAR (2012). PEPFAR blueprint: creating an AIDS-Free generation.

[15] Schwartlander B, Stover J, Hallett T, Atun R, Avila C, Gouws E (2011). Towards an improved investment approach for an effective response to HIV/AIDS. Lancet..

[16] Dai JY, Gilbert PB, Hughes JP, Brown ER (2013). Estimating the efficacy of preexposure prophylaxis for HIV prevention among participants with a threshold level of drug concentration. Am J Epidemiol..

[17] Roberts ET, Matthews DD (2012). HIV and chemoprophylaxis, the importance of considering social structures alongside biomedical and behavioral intervention. Soc Sci Med..

[18] Earnshaw VA, Bogart LM, Dovidio JF, Williams DR (2013). Stigma and racial/ethnic HIV disparities: moving toward resilience. Am Psychol..

[19] Stangl A, Go V, Zelaya C, Brady L, Nyblade L, Stackpool-Moore L (2010). Enabling the scale-up of efforts to reduce HIV stigma and discrimination: a new framework to inform program implementation and measurement.

[20] Goffman E (1963). Stigma: notes on the management of spoiled identity.

[21] Link BG, Phelan JC (2001). Conceptualizing stigma. Annu Rev Sociol..

[22] Deacon H (2006). Towards a sustainable theory of health-related stigma: lessons from the HIV/AIDS literature. J Community Appl Soc Psychol..

[23] Stangl A, Brady B, Fritz K (2012). Technical brief: measuring HIV stigma and discrimination.

[24] Ogden J, Nyblade L (2005). Common at its core: HIV-related stigma across contexts.

[25] Nyblade L, Pande RP, Mathur S, MacQuarrie K, Kidd R, Banteyerga H (2003). Disentangling HIV and AIDS stigma in Ethiopia, Tanzania and Zambia.

[26] Hong KT, Van Anh NT, Ogden J (2004). Because this is the disease of the century: understanding HIV and AIDS-related stigma and discrimination.

[27] Mahendra VS, Gilborn L, Bharat S, Mudoi RJ, Gupta I, George B (2007). Understanding and measuring AIDS-related stigma in health care settings: a developing country perspective. J Soc Aspects HIV/AIDS..

[28] Reidpath DD, Chan KY (2005). A method for the quantitative analysis of the layering of HIV-related stigma. AIDS Care..

[29] Nyblade L (2006). Measuring HIV stigma: existing knowledge and gaps. Psychol Health Med..

[30] Loutfy MR, Logie CH, Zhang Y, Blitz SL, Margolese SL, Tharao WE (2012). Gender and ethnicity differences in HIV-related stigma experienced by people living with HIV in Ontario, Canada. PLoS One..

[31] Logie C, James L, Tharao W, Loutfy M (2013). Associations between HIV-related stigma, racial discrimination, gender discrimination, and depression among HIV-positive African, Caribbean, and Black women in Ontario, Canada. AIDS Patient Care STDs..

[32] Quinn DM, Chaudoir SR (2009). Living with a concealable stigmatized identity: the impact of anticipated stigma, centrality, salience, and cultural stigma on psychological distress and health. J Pers Soc Psychol..

[33] Zelaya CE, Sivaram S, Johnson SC, Srikrishnan AK, Suniti S, Celentano D (2012). Measurement of self, experienced, and perceived HIV/AIDS stigma using parallel scales in Chennai, India. AIDS Care..

[34] Rao D, Desmond M, Andrasik M, Rasberry T, Lambert N, Cohn SE (2012). Feasibility, acceptability, and preliminary efficacy of the unity workshop: an internalized stigma reduction intervention for African American women living with HIV. AIDS Patient Care STDs..

[35] Burris S (2008). Stigma, ethics and policy: a commentary on Bayer's “Stigma and the ethics of public health: not can we but should we”. Soc Sci Med..

[36] Mahajan AP, Sayles JN, Patel VA, Remien RH, Sawires SR, Ortiz DJ (2008). Stigma in the HIV/AIDS epidemic: a review of the literature and recommendations for the way forward. AIDS..

[37] Earnshaw VA, Smith LR, Chaudoir SR, Lee I-C, Copenhaver MM (2012). Stereotypes about people living with HIV: implications for perceptions of HIV risk and testing frequency among at-risk populations. AIDS Educ Prev..

[38] AIDS 2031 Social Drivers Working Group (2013). Revolutionizing the AIDS response: building AIDS resilient communities. AIDS 2031, International Development, Community and Environment (IDCE), Clark University and International Center for Research on Women (ICRW)..

[39] Parkhurst JO (2012). HIV prevention, structural change and social values: the need for an explicit normative approach. J Int AIDS Soc..

[40] Nyblade L, Stangl A, Weiss E, Ashburn K (2009). Combating HIV stigma in health care settings: what works?. J Int AIDS Soc..

[41] McLeroy KR, Bibeau D, Steckler A, Glanz K (1988). An ecological perspective on health promotion programs. Health Educ Q..

[42] Heijnders M, van der Meij S (2006). The fight against stigma: an overview of stigma-reduction strategies and interventions. Psychol Health Med..

[43] Brown L, Macintyre K, Trujillo L (2003). Interventions to reduce HIV/AIDS stigma: what have we learned?. AIDS Educ Prev..

[44] Sengupta S, Banks B, Jonas D, Miles MS, Smith GC (2011). HIV interventions to reduce HIV/AIDS stigma: a systematic review. AIDS Behav..

[45] Gupta GR, Parkhurst JO, Ogden JA, Aggleton P, Mahal A (2008). Structural approaches to HIV prevention. Lancet..

[46] Tsai AC (2012). A typology of structural approaches to HIV prevention: a commentary on Roberts & Matthews. Soc Sci Med..

[47] Roberts ET, Matthews DD (2012). A reorientation towards structural solutions, considerations for HIV prevention: a response to Tsai. Soc Sci Med..

[48] Kippax S (2008). Understanding and integrating the structural and biomedical determinants of HIV infection: a way forward for prevention. Curr Opin HIV AIDS..

[49] UNAIDS (2005). HIV-related stigma, discrimination and human rights violations.

[50] UNAIDS, IPPF (2004). A brief report of the IPPF and UNAIDS Joint Consultative Meeting on PLWA rights index.

[51] UNAIDS (2006). Courting rights: case studies in litigation the human rights of people living with HIV. Geneva, Switzerland: Joint United Nations Programme on HIV/AIDS (UNAIDS).

[52] Parker R, Aggleton P (2003). HIV and AIDS-related stigma and discrimination: a conceptual framework and implications for action. Soc Sci Med..

[53] Wolfe WR, Weiser SD, Leiter K, Steward WT, Percy-de Korte F, Phaladze N (2008). The impact of universal access to antiretroviral therapy on HIV stigma in Botswana. Am J Publ Health..

[54] Roura M, Urassa M, Busza J, Mbata D, Wringe A, Zaba B (2009). Scaling up stigma? The effects of antiretroviral roll-out on stigma and HIV testing. Early evidence from rural Tanzania. Sex Transm Infect..

[55] Downs SH, Black N (1998). The feasibility of creating a checklist for the assessment of the methodological quality both of randomised and non-randomised studies of health care interventions. J Epidemiol Community Health..

[56] Livingston JD, Milne T, Fang ML, Amari E (2012). The effectiveness of interventions for reducing stigma related to substance use disorders: a systematic review. Addiction..

[57] Spencer L, Ritchie J, Lewis J, Dillon L (2003). Quality in qualitative evaluation: a framework for assessing research evidence. National Centre for Social Research.

[58] Gurnani V, Beattie TS, Bhattacharjee P, Team CFAR, Mohan HL, Maddur S (2011). An integrated structural intervention to reduce vulnerability to HIV and sexually transmitted infections among female sex workers in Karnataka state, south India. BMC Public Health..

[59] Pisal H, Sutar S, Sastry J, Kapadia-Kundu N, Joshi A, Joshi M (2007). Nurses’ health education program in India increases HIV knowledge and reduces fear. J Assoc Nurses AIDS Care..

[60] Li L, Liang LJ, Lin C, Wu Z, Rotheram-Borus MJ (2010). HIV prevention intervention to reduce HIV-related stigma: evidence from China. AIDS..

[61] Wang D, Operario D, Hong Q, Zhang H, Coates TJ (2009). Intervention to train physicians in rural China on HIV/STI knowledge and risk reduction counseling: preliminary findings. AIDS Care..

[62] Williams AB, Wang H, Burgess J, Wu C, Gong Y (2006). Effectiveness of an HIV/AIDS educational programme for Chinese nurses. J Adv Nurs..

[63] Wu S, Li L, Wu Z, Liang LJ, Cao H, Yan Z (2008). A brief HIV stigma reduction intervention for service providers in China. AIDS Patient Care STDs..

[64] Lau JTF, Tsui HY, Chan K (2005). Reducing discriminatory attitudes toward people living with HIV/AIDS (PLWHA) in Hong Kong: an intervention study using an integrated knowledge-based PLWHA participation and cognitive approach. AIDS Care..

[65] Li X, Zhang L, Mao R, Zhao Q, Stanton BF (2011). Effect of social cognitive theory-based HIV education prevention program among high school students in Nanjing, China. Health Educ Res..

[66] Paxton S (2002). The impact of utilizing HIV-positive speakers in AIDS education. AIDS Educ Prev..

[67] Sorcar P (2009). Teaching taboo topics without talking about them: an epistemic study of a new approach to HIV/AIDS prevention education in India..

[68] Yiu JW, Mak WWS, Ho WS, Chui YY (2010). Effectiveness of a knowledge-contact program in improving nursing students’ attitudes and emotional competence in serving people living with HIV/AIDS. Soc Sci Med..

[69] Nambiar D, Ramakrishnan V, Kumar P, Varma R, Balaji N, Rajendran J (2011). Knowledge, stigma, and behavioral outcomes among antiretroviral therapy patients exposed to Nalamdana's Radio and Theater Program in Tamil Nadu, India. AIDS Educ Prev..

[70] Rimal RN, Creel AH (2008). Applying social marketing principles to understand the effects of the radio diaries program in reducing HIV/AIDS stigma in Malawi. Health Market Q..

[71] Kaponda CPN, Jere DL, Chimango JL, Chimwaza AF, Crittenden KS, Kachingwe SI (2009). Impacts of a peer-group intervention on HIV-related knowledge, attitudes, and personal behaviors for urban hospital workers in Malawi. J Assoc Nurses AIDS Care..

[72] Bell CC, Bhana A, Petersen I, McKay MM, Gibbons R, Bannon W (2008). Building protective factors to offset sexually risky behaviors among black youths: a randomized control trial. J Natl Med Assoc..

[73] Bekele A, Ali A (2008). Effectiveness of IEC interventions in reducing HIV/AIDS related stigma among high school adolescents in Hawassa, Southern Ethiopia. Ethiop J Health Dev..

[74] Brown DC (2009). Reducing HIV and AIDS stigma among university students in South Africa.

[75] Chao L-W, Gow J, Akintola G, Pauly M (2010). HIV/AIDS stigma attitudes among educators in KwaZulu-Natal, South Africa. J Sch Health..

[76] Norr KF, Norr JL, Kaponda CP, Kachingwe SI, Mbweza EM (2007). Short-term effects of a peer group intervention for HIV prevention among trainee teachers in Malawi. Afr J AIDS Res..

[77] Esu-Williams E, Schenk K, Motsepe J, Geibel S, Zulu A Involving young people in the care and support of people living with HIV and AIDS in Zambia.

[78] Mall S, Middelkoop K, Mark D, Wood R, Bekker LG (2012). Changing patterns in HIV/AIDS stigma and uptake of voluntary counselling and testing services: the results of two consecutive community surveys conducted in the Western Cape, South Africa. AIDS Care..

[79] Denison JA, Tsui S, Bratt J, Torpey K, Weaver MA, Kabaso M (2012). Do peer educators make a difference? An evaluation of a youth-led HIV prevention model in Zambian Schools. Health Educ Res..

[80] Jürgensen M, Sandøy IF, Michelo C, Fylkesnes K, ZAMACT Study Group (2013). Effects of home-based voluntary counselling and testing on HIV-related stigma: findings from a cluster-randomized trial in Zambia. Soc Sci Med..

[81] Adam BD, Murray J, Ross S, Oliver J, Lincoln SG, Rynard V (2011). hivstigma.com, an innovative web-supported stigma reduction intervention for gay and bisexual men. Health Educ Res..

[82] Deutsch H (2007). Reducing HIV stigma: a common group identity perspective. Honors Projects. Paper 12. http://digitalcommons.iwu.edu/psych_honproj/12.

[83] Gordon-Garofalo VL (2004). Evaluation of a psychoeducational group for seronegative partners and spouses of persons with HIV/AIDS..

[84] Hosek SG, Lemos D, Harper GW, Telander K (2011). Evaluating the acceptability and feasibility of project accept: an intervention for youth newly diagnosed with HIV. AIDS Educ..

[85] Boulay M, Tweedie I, Fiagbey E (2008). The effectiveness of a national communication campaign using religious leaders to reduce HIV-related stigma in Ghana. Afr J AIDS Res..

[86] Fakolade R, Adebayo SB, Anyanti J, Ankomah A (2010). The impact of exposure to mass media campaigns and social support on levels and trends of HIV-related stigma and discrimination in Nigeria: tools for enhancing effective HIV prevention programmes. J Biosocial Sci..

[87] Ezedinachi ENU, Ross MW, Meremiku M, Essien EJ, Edem CB, Ekure E (2002). The impact of an intervention to change health workers’ HIV/AIDS attitudes and knowledge in Nigeria: a controlled trial. Public Health..

[88] Saad A, Lekhraj R, Sabitu K, AbdulRahman H, Awaisu A, AbuSamah B (2012). An HIV-STI risk reduction program among undergraduate students at a northern Nigerian university: a randomized controlled field trial..

[89] Smith Fawzi MC, Eustache E, Oswald C, Louis E, Surkan PJ, Scanlan F (2012). Psychosocial support intervention for HIV-affected families in Haiti: implications for programs and policies for orphans and vulnerable children. Soc Sci Med..

[90] Young SD, Konda K, Caceres C, Galea J, Sung-Jae L, Salazar X (2010). Effect of a community popular opinion leader HIV/STI intervention on stigma in urban, Coastal Peru. AIDS Behav..

[91] Norr KF, Ferrer L, Cianelli R, Crittenden KS, Irarraízabal L, Cabieses B (2012). Peer group intervention for HIV prevention among health workers in Chile. J Assoc Nurses AIDS Care..

[92] Al-Mazrou YY, Abouzeid MS, Al-Jeffri MH (2005). Impact of health education on knowledge and attitudes of Saudi paramedical students toward HIV/AIDS. Saudi Med J..

[93] Khuat TO, Ashburn K, Pulerwitz J, Ogden J, Nyblade L (2008). Improving hospital-based quality of care in Vietnam by reducing HIV-related stigma and discrimination, a Horizons final report.

[94] Li L, Wu Z, Liang LJ, Lin C, Guan J, Jia M (2013). Reducing HIV-related stigma in health care settings: a randomized controlled trial in China. Am J Public Health..

[95] Uys L, Chirwa M, Kohi T, Greeff M, Naidoo J, Makoae L (2009). Evaluation of a health setting-based stigma intervention in five African countries. AIDS Patient Care STDs..

[96] Nyblade L, Hong KT, Anh NV, Ogden J, Jain A, Stangl A (2008). Communities confront HIV stigma in Viet Nam: participatory interventions reduce HIV-related stigma in two provinces.

[97] Nuwaha F, Kasasa S, Wana G, Muganzi E, Tumwesigye E (2012). Effect of home-based HIV counselling and testing on stigma and risky sexual behaviours: serial cross-sectional studies in Uganda. J Int AIDS Soc..

[98] Lakshmi MG, Sampathkumar A (2013). Impact of psycho-education on stigma in people living with HIV/AIDS. Int J Soc Sci Tomorrow..

[99] Neema S, Atuyambe LM, Otolok-Tanga B, Twijukye C, Kambugu A, Thayer L (2012). Using a clinic based creativity initiative to reduce HIV related stigma at the Infectious Diseases Institute, Mulago National Referral Hospital, Uganda. Afr Health Sci..

[100] Tshabalala J, Visser MJ (2011). Developing a cognitive behavioural therapy model to assist women to deal with HIV and stigma. S Afr J Psychol..

[101] Apinundecha C (2007). A community participation intervention to reduce HIV/AIDS stigma, Nakhon Ratchasima province, northeast Thailand..

[102] Maughan-Brown B (2010). Stigma rises despite antiretroviral roll-out: a longitudinal analysis in South Africa. Soc Sci Med..

[103] Richter K, Phillips SC, McInnis AM, Rice DA (2012). Effectiveness of a multi-country workplace intervention in sub-Saharan Africa. AIDS Care..

[104] Biradavolu MR, Blankenship KM, Jena A, Dhungana N (2012). Structural stigma, sex work and HIV: contradictions and lessons learnt from a community-led structural intervention in southern India. J Epidemiol Community Health..

[105] Bharat S (2011). A systematic review of HIV/AIDS-related stigma and discrimination in India: current understanding and future needs. SAHARA J..

[106] Nachega JB, Stein DM, Lehman DA, Hlatshwayo D, Mothopeng R, Chaisson RE (2004). Adherence to antiretroviral therapy in HIV-infected adults in Soweto, South Africa. AIDS Res Hum Retrovir..

[107] UNAIDS (2007). Reducing stigma and discrimination: a critical part of National AIDS Programmes.

[108] Khumalo-Sakutukwa G, Morin S, Fritz K, Charlebois ED, vanRooyen H, Chingono A (1999). Project Accept (HPTN 043): a community-based intervention to reduce HIV incidence in populations at risk for HIV in sub-Saharan Africa and Thailand. Journal of acquired immune deficiency syndromes..

[109] Marazzo JM, Ramjee G, Nair G, Palanee T, Mkhize B, Nakabiito C (2013). Pre-exposure prophylaxis for HIV in women: daily oral tenofovir, oral tenofovir/emtricitabine or vaginal tenofovir gel in the VOICE study. 20th Conference on Retroviruses and Opportunistic Infections.

[110] UNAIDS (2010). Making the law work for the HIV response: country snapshot. The International Planned Parenthood Federation, the Global Network of People living with HIV, the International Lesbian, Gay, Bisexual, Trans and Intersex Association, the International Harm Reduction Association, and the Joint United Nations Programme on HIV/AIDS.

[111] UNAIDS (2012). Global report: UNAIDS report on the Global AIDS epidemic.

[112] Baral SD, Wirtz A, Sifakis F, Johns B, Walker D, Beyrer C (2012). The Highest Attainable Standard of Evidence (HASTE) for HIV/AIDS interventions: toward a public health approach to defining evidence. Publ Health Rep..

[113] Auerbach JD, Parkhurst JO, Caceres CF (2011). Addressing social drivers of HIV/AIDS for the long-term response: conceptual and methodological considerations. Glob Public Health..

[114] Apinundecha C, Laohasiriwong W, Cameron MP, Lim S (2007). A community participation intervention to reduce HIV/AIDS stigma, Nakhon Ratchasima province, northeast Thailand. AIDS Care..

[115] Seeley J, Watts CH, Kippax S, Russell S, Heise L, Whiteside A (2012). Addressing the structural drivers of HIV: a luxury or necessity for programmes?. J Int AIDS Soc..

[116] Kalichman SC, Simbayi LC, Cloete A, Mthembu P, Mkhonta RN, Ginindza T (2009). Measuring AIDS stigmas in people living with HIV/AIDS: the internalized AIDS-related stigma scale. AIDS Care..

[117] Visser MJ, Kershaw T, Makin JD, Forsyth BWC (2008). Development of parallel scales to measure HIV-related stigma. AIDS Behav..

[118] Zelaya CE, Sivaram S, Sethulakshmi CJ, Srikrishnan AK, Solomon S, Celentano DD (2008). HIV/AIDS stigma: reliability and validity of a new measurement instrument in Chennai, India. AIDS Behav..

[119] Genberg BL, Kawichai S, Chingono A, Sendah M, Chariyalertsak S, Konda KA (2007). Assessing HIV/AIDS stigma and discrimination in developing countries. AIDS Behav..

[120] Earnshaw VA, Chaudoir SR (2009). From conceptualizing to measuring HIV stigma: a review of HIV stigma mechanism measures. AIDS Behav..

[121] Berger BE, Ferrans CE, Lashley FR (2001). Measuring stigma in people with HIV: psychometric assessment of the HIV stigma scale. Res Nurs Health..

[122] Uys LR, Holzemer WL, Chirwa ML, Dlamini PS, Greeff M, Kohi TW (2009). The development and validation of the HIV/AIDS Stigma Instrument – Nurse (HASI-N). AIDS Care..

[123] Wu AW, Revicki DA, Jacobson D, Malitz FE (1997). Evidence for reliability, validity and usefulness of the Medical Outcomes Study HIV Health Survey (MOS-HIV). Qual Life Res..

[124] Tanser F, Barnighausen T, Grapsa E, Zaidi J, Newell ML (2013). High coverage of ART associated with decline in risk of HIV acquisition in rural KwaZulu-Natal, South Africa. Science..

[125] Cohen MS, Baden LR (2012). Preexposure prophylaxis for HIV. Where do we go from here?. N Engl J Med..

[126] Grant RM, Lama JR, Anderson PL, McMahan V, Liu AY, Vargas L (2010). Preexposure chemoprophylaxis for HIV prevention in men who have sex with men. N Engl J Med..

[127] Karim QA, Karim SSA, Frohlich JA, Grobler AC, Baxter C, Mansoor LE (2010). Effectiveness and safety of tenofovir gel, an antiretroviral microbicide, for the prevention of HIV infection in women. Science..

# Sub article: Resisting and challenging stigma in Uganda: the role of support groups of people living with HIV

**DOI:** 10.7448/IAS.16.3.18636

**Published:** 2013-11-13

**Authors:** Gitau Mburu, Mala Ram, Morten Skovdal, David Bitira, Ian Hodgson, Grace W Mwai, Christine Stegling, Janet Seeley

**Affiliations:** 1 International HIV/AIDS Alliance, Hove, UK; 2 Division of Health Research, Lancaster University, Lancaster, UK; 3 Institute of Social Psychology, London School of Economics and Political Science, London, UK; 4 Community Health Alliance Uganda, Kampala, Uganda; 5 Centre for Global Health, Trinity College Dublin, Dublin, Ireland; 6 Division of Medical Education, Brighton and Sussex Medical School, University of Brighton, Brighton, UK; 7 School of International Development, University of East Anglia, Norwich, UK; 8 MRC UVRI Uganda Research Unit on AIDS, Entebbe, Uganda

**Keywords:** HIV, stigma, Uganda, Africa

## Abstract

**Introduction:**

Global scale up of antiretroviral therapy is changing the context of HIV-related stigma. However, stigma remains an ongoing concern in many countries. Groups of people living with HIV can contribute to the reduction of stigma. However, the pathways through which they do so are not well understood.

**Methods:**

This paper utilizes data from a qualitative study exploring the impact of networked groups of people living with HIV in Jinja and Mbale districts of Uganda. Participants were people living with HIV (*n=*40), members of their households (*n=*10) and their health service providers (*n=*15). Data were collected via interviews and focus group discussions in 2010, and analyzed inductively to extract key themes related to the approaches and outcomes of the groups’ anti-stigma activities.

**Results:**

Study participants reported that HIV stigma in their communities had declined as a result of the collective activities of groups of people living with HIV. However, they believed that stigma remained an ongoing challenge. Gender, family relationships, social and economic factors emerged as important drivers of stigma. Challenging stigma collectively transcended individual experiences and united people living with HIV in a process of social renegotiation to achieve change. Groups of people living with HIV provided peer support and improved the confidence of their members, which ultimately reduced self-stigma and improved their ability to deal with external stigma when it was encountered.

**Conclusions:**

Antiretroviral therapy and group-based approaches in the delivery of HIV services are opening up new avenues for the collective participation of people living with HIV to challenge HIV stigma and act as agents of social change. Interventions for reducing HIV stigma should be expanded beyond those that aim to increase the resilience and coping mechanisms of individuals, to those that build the capacity of groups to collectively cope with and challenge HIV stigma. Such interventions should be gender sensitive and should respond to contextual social, economic and structural factors that drive stigma.

## Introduction

HIV stigma is a clearly documented obstacle to HIV testing [1, 2], disclosure of HIV status [3, 4], uptake of antiretroviral therapy and retention in care [5]. HIV stigma can also aggravate mental health problems [6, 7] and significantly reduce the quality of life of people living with HIV [8]. There is therefore an urgent need to de-stigmatize HIV.

HIV stigma exists worldwide, and common drivers and manifestations of HIV stigma are recognized across different settings [9]. At the same time, the extent to which HIV stigma is experienced by people living with HIV varies considerably within and across different contexts. Experiences of HIV stigma may be shaped, for instance, by underlying stigmatization of specific behaviours such as sex work and injecting drug use, as well as by individual resilience [10].

There is a wide body of literature exploring HIV stigma, which is now recognized as a complex multidimensional phenomenon [5, 11, 12]. As such, it has proved challenging to define. Deacon *et al*. [12, p. 19] identify core elements of HIV stigma when they propose defining it as “an ideology that claims that people with a specific disease are different from ‘normal’ society, more than simply through their infection with a disease agent,” and also as a “social process by which people use shared social representations to distance themselves and their in-group from the risk of contracting a disease.” An exploration of this social process shows that HIV stigma is often influenced by the contribution an individual makes to society, that is, whether he or she is regarded as a drain on communal resources [13, 14].

Such material symbolism of stigma is pertinent as more people living with HIV enrol for treatment, live longer and gain employment [15, 16]. Widespread availability of treatment has been associated with an improved or so called “Lazarus” health outcomes, regained self-esteem [11], improved life expectancy [17] and reduced HIV stigma, for instance in Uganda and Botswana [18, 19]. These findings, which appear to confirm prior predictions that antiretroviral therapy could reduce HIV stigma [13], have led some researchers to question the extent to which HIV stigma persists in countries such as Uganda and its relevance to future HIV programming [20].

In a review of interventions targeting HIV-related stigma, Brown *et al*. [21] describe a conceptual framework that includes four types of approaches for de-stigmatizing HIV: first, information-based approaches, such as brochures; second, skills-building activities and other hands-on learning strategies that counter negative attitudes; third, counselling approaches; and fourth, contact with people living with HIV, for instance through testimonials and interaction with the general public.

In this paper, we consider the fourth approach, that is, pathways through which contact between people living with HIV and their communities could contribute to de-stigmatizing HIV. In particular, we explore the extent to which these interactions are influenced by the collective efficacy or resistance of people living with HIV, that is, the extent to which they take action to change their own circumstances [22].

This is important given that recent studies conducted in Zimbabwe, Tanzania and Botswana have shown that simply increasing the availability of antiretroviral treatment and counselling may not, on its own, be sufficient to reduce HIV stigma. Rather, in order to have an impact on stigma, antiretroviral therapy should be coupled with strategies that enable people living with HIV to better cope with and resist stigma, such as peer support groups [23, 24]. In this paper, we build on these findings by exploring how people living with HIV in Uganda contribute collectively to countering stigma. Based on recommendations from Brown *et al*. [21], we examine how groups of people living with HIV can nurture a collective efficacy that protects their members from the negative effects of stigma, while at the same time contributing to the de-stigmatization of HIV. Our focus is on “groups” as the unit of analysis rather than individual-level support, which is already well documented in Uganda, for instance in relation to The AIDS Support Organisation (TASO) model [25].

## Methods

### Setting

Data presented in this paper were collected as part of a qualitative study documenting the model and activities of networked groups of people living with HIV in Uganda, whose main findings are reported elsewhere [26, 27]. This paper focuses specifically on stigma reduction, based on previously unpublished data. Data were collected between June and October 2010 in Uganda's Mbale and Jinja districts, where the International HIV/AIDS Alliance had implemented a community-based HIV initiative known as the “Networks project” during the preceding four years, whose aim was to increase access to a comprehensive continuum of HIV services.

### Intervention

Central to the Networks project was the concept of meaningful involvement of groups of people living with HIV, which empowered them to be engaged as *partners* in the delivery of HIV services, as opposed to being *passive recipients* of services [28]. This was achieved through three approaches: first, mapping and supporting 750 existing groups of people living with HIV to organize themselves into a network of 120 larger sub-national clusters; second, training the groups on comprehensive HIV prevention and care, record keeping, income generation, advocacy and financial and general project management; and third, implementing community-based HIV prevention, care and treatment referral activities with the groups as partners, as described in detail elsewhere [27]. These groups were functional in 40 districts, with a total membership of more than 40,000 people living with HIV [27, 28].

### Group activities

Groups of people living with HIV mobilized their peers; provided community education; acted as patient ushers at HIV clinics; visited homes of people living with HIV; counselled household members on how to care for people living with HIV without prejudice; and performed HIV sensitization campaigns aimed at their communities. All of these activities were intended to increase HIV service uptake, but some may also have contributed to countering HIV stigma. Following the implementation of the project, this qualitative study was performed to explore processes leading to change, using two districts that represent diverse rural (Mbale) and urban (Jinja) settings.

### Participants

This paper, which focuses on HIV stigma, includes data from all 65 participants in the larger qualitative study: 40 people living with HIV (*n=*40), members of their households (*n=*10) and their health service providers (*n=*15), who were initially selected based on their previous involvement with the Networks project and their willingness to participate. Diverse participants were selected to enable triangulation of findings and to ensure that a wide range of perspectives would be captured [29], given that perceptions of HIV stigma in Uganda can differ between health service providers and family members [20]. A total of 25 study participants provided interviews, and the other 40 participants contributed to focus group discussions (Table 1).

**Table 1:** Study participants and methodology

	Interviews		Focus group discussions	
Population	People living with HIV*	Key informants**	People living with HIV*	Members of households with people with HIV
Sample size	10	15	3 sessions; *n=*30	1 session; *n=*10
Location	Jinja, Mbale	Jinja, Mbale	Jinja, Mbale	Jinja

* Examples of groups of people living with HIV from which participants were selected include Jinja People Living with HIV/AIDS Drama Group, Positive Men's Union, WIDE, Abatwogerera, NAKOLO, Khulirire Adwela, Mukwano Women's Association and Food Security TASO Group.

** Key informants included district health officers, district HIV focal persons, district AIDS coordinators, community leaders, medical superintendents of district hospitals, antiretroviral therapy clinic supervisors and leaders of groups of people living with HIV.

### Data collection

Interview guides and topics for the focus group discussions were developed in reference to existing gaps in the literature and the study objectives. These included exploring why people living with HIV formed (or joined) groups with others; how groups related to each other; how groups facilitated disclosure and visibility for people living with HIV; and how group activities influenced stigma and uptake of services (*see* Additional file 1 for topic guides). The tools were validated during a pilot phase that took into account the contextual environment of the study setting. These tools were then translated into Luganda and Lusoga for use when participants preferred to be interviewed in local languages instead of English. In these instances, a researcher who could speak that language conducted the interviews or focus group discussions. Researchers back-translated the local versions of the study tools to ensure that the meaning of the questions had not been altered. Interviews lasted 25–50 minutes, while focus group discussions lasted 45–60 minutes. Interviews and focus group discussions were conducted by researchers who were trained on ethical study conduct. Interviews and focus group discussions were audio recorded and transcribed. Data in Luganda and Lusoga were translated into English.

### Data analysis

Data were reviewed and all text segments subjected to a thematic analysis using QSR International's NVivo 7 [30], based on the initial study questions. These questions focused on the role of groups of people living with HIV in disclosure, visibility and HIV prevention and care, and the relationships between these groups and households of people living with HIV (see Supplementary files for topic guides). Data were systematically classified and organized by major themes and concepts [31] relating to collective efficacy and resistance to stigma, and the outcomes of these; factors that perpetuate stigma; and activities through which people living with HIV contribute to de-stigmatizing HIV.

### Ethical considerations

The study was approved by the Science and Ethics Committees of the Uganda Virus Research Institute and the Uganda National Council for Science and Technology. All personally identifiable information was deleted and data were held in a secure, password-protected computer at all times.

## Results

### Collective efficacy and resistance to stigma

In this study, challenging stigma transcended individual experiences and united people living with HIV in a process of social renegotiation. They sought to empower themselves and change their collective standing in the community. Challenging stigma transitioned from the individual to the collective domain.

People living with HIV wanted to mobilise so that they could come together and fight stigma and discrimination. (Focus group discussion, household members of people living with HIV, Jinja)

What motivated me to join this group was because we were isolated and stigma was too much in the community. (Focus group discussion, people living with HIV, Mbale)

Findings also suggest that increased interaction between people openly living with HIV and other community members through testimonials and other forms of interaction may have contributed to the perceived decline in stigma by demystifying HIV, as suggested by Brown *et al*. [21].

It has reduced because of the interaction between group members and community people. (Interview, male key informant, Jinja)

Involvement of people living with HIV in income-generating activities (within the Networks project) offered an opportunity for them to interact with their communities. This was particularly important given the relationship between poverty and HIV-related stigma in this setting, and more generally in sub-Saharan Africa [16].

Their success in … animal rearing and vegetable growing encouraged other community people to come and learn from the group, thereby increasing interaction between the community and the group members. (Interview, man living with HIV, Jinja)

People living with HIV who were successful in income-generating activities were no longer perceived as draining community resources, but as making a contribution instead, which underpins the material symbolism of HIV stigma [16].

Nowadays people in the community have realised the importance and usefulness of people living with HIV. They appreciate the role of the groups. This has reduced stigma. (Interview, man living with HIV, Jinja)

When the community members see the work we are doing in our groups, yet they didn't initially think we were capable of doing it, they start believing and having confidence in us. (Interview, woman living with HIV, Jinja)

Frequent notions emerged of the ways in which groups of people living with HIV increased their social capital through enhanced social inclusion and cohesion with their communities. This was determined by the contribution that the groups were perceived to be making, hence their “usefulness” to the larger community. Thus, being economically well-off appeared to cushion people living with HIV from being stigmatized, especially men.

I was not stigmatised or discriminated [against] because I was doing well financially and supporting my family ably. (Interview, man living with HIV, Jinja)

Not surprisingly, collective resistance was shaped by important factors driving stigma and self-stigma (feelings of shame, guilt and self-blame), including gender, family relationships and (as noted above) material wellbeing. Groups of people living with HIV responded to these factors either *directly*, for instance, by engaging in income generation to counter poverty, or *indirectly*, for instance, by proving a social space in which the impact of gender as a driver of stigma could be countered through peer support. This was particularly relevant given that social norms relating to men's role in society often contributed to self-stigma. Our study showed that it was men who had most difficulty in joining groups.

As men, we are [expected] to take care of our families. But because of poor health and stigma, we are unable to fulfil these family obligations. I had a lot of self-stigma and needed to join people with whom I could share the problem. (Focus group discussion, people living with HIV, Jinja)

There were many [people living with HIV] who were in hiding, especially men. Positive Men's Union encouraged them to come out. Men have been poor to join groups but this [group] will attract them more. (Interview, female key informant, Jinja)

Once mobilized, people living with HIV became involved in a number of activities that they saw as having an impact either on the level of stigma or on the way in which members coped with stigma (Table 2).

**Table 2:** Approaches and activities employed by groups of people living with HIV to counter HIV stigma

Approach	Illustrative quote
Peer support and counselling	*We needed to come together so that we could mobilise other people living with HIV in the communities, so that we could discuss and counsel one another to cope with stigma*. (Interview, woman living with HIV, Jinja)
	*The group members also go and reach out to people living with HIV in households who are facing problems like stigma and discrimination; support those on treatment to adhere to it; and also check on the general hygiene in the home*. (Focus group discussion, household members of people living with HIV, Jinja)
Community education and sensitization	*We have a drama group that goes around mobilising and sensitising people to create awareness*. (Focus group discussion, household members of people living with HIV, Jinja)
	*They also help bridge gaps of knowledge and clear myths that people have about HIV to reduce stigma*. (Interview, male key informant, Jinja)
	*The group has helped educate us and the community on issues like why test and how to overcome stigma and get self-confidence*. (Interview, male key informant, Mbale)
Media and printed information	*They are in [a drama group that] prepares songs [and] plays on HIV topics like [prevention of mother-to-child transmission] and the use of [antiretrovirals] and [their] benefits, and also on stigma and discrimination*. (Focus group discussion – household members of people living with HIV, Jinja)
	*We even talk on the radio and tell people we are … living with HIV*. (Interview, female key informant, Jinja)
Public testimonials and role modelling	*We also encourage giving of testimonies by people living with HIV in public*. (Focus group discussion – household members of people living with HIV, Jinja)
	*Public disclosure enabled me to reach out to others, to sensitise and educate them about HIV and to change people's attitudes towards people living with HIV*. (Interview, man living with HIV, Jinja)
	*They see me as an example and role model to copy from*. (Focus group discussion, people living with HIV, Jinja)

### Outcomes of collective efficacy and resistance

According to some study participants, groups’ activities had positive impacts on both self-stigma and stigma in the community.

Stigma amongst ourselves has reduced. There were members who had self-stigma, [but] today they are able to move out and talk about themselves. (Focus group discussion, people living with HIV, Jinja)

These groups have had an impact on communities’ attitudes towards people living with HIV. This has brought down the level of stigma and discrimination. (Interview, female key informant, Mbale)

Study participants reported that false beliefs regarding HIV were diminishing in the community.

They no longer think HIV is due to witchcraft because of an improved health-seeking culture, rather than going to shrines. (Interview, male key informant, Mbale)

While study participants reported that HIV stigma in their communities had generally declined over time, they believed it remained a powerful force in the lives of people living with HIV, even at the household level.

One of our members died recently as a result of being discriminated [against] and neglected by her family, who isolated her and failed to remind her to take her drugs. (Interview, man living with HIV, Jinja)

In addition, groups did not always have a positive impact on stigma. There were instances, especially initially, where association with groups was stigmatizing.

Many people feared coming to us openly, thinking that when others see them with us, they will be branded having HIV. (Interview, man living with HIV, Mbale)

## Discussion

Contrary to assertions that stigma may no longer be relevant in the face of a mature HIV epidemic and widespread antiretroviral access [13, 20], our study found that stigma remains a concern among people living with HIV in Uganda, where antiretroviral coverage is estimated to be between 52 and 81% [32]. We argue that our study captures a dynamic period in which stigma has started to diminish but has not yet been fully eliminated in the study districts. A recent study in Uganda showed that the impact of antiretroviral therapy on stigma is most marked during the first two years of treatment, after which its effect on stigma declines significantly [33]. This could account for the apparent paradox that stigma is both in decline and yet persistent in our study setting. This resonates with the traditionally held view that stigma is dynamic [12], and as such it could persist or even increase in the context of wider availability of antiretroviral therapy, as demonstrated in recent studies from Botswana [19] and South Africa [34].

An important finding from our study relates to how groups of people living with HIV can contribute to protecting their members from HIV stigma while at the same time de-stigmatizing HIV in their communities. Our study demonstrates that groups of people living with HIV can directly address factors known to influence HIV stigma, such as poverty [16], through collective participation in livelihood activities that would otherwise be difficult to accomplish individually, or through collective resistance by challenging stigma publicly. In our study, the collective activities of these groups (for instance, drama and income generation) provided practical skills to cope with external stigma, and confidence to overcome self-stigma. This pooling of labour and resources is a distinctive advantage of a “group” approach [35].

Our findings build and expand on the conceptual framework of effective approaches for reducing HIV stigma by Brown *et al*. [21]. This framework suggests that a high level of interaction and proximity between people with HIV and their communities demystifies HIV and reduces stigma [21]. While support groups of people living with HIV have been known to exist elsewhere [36], what was different about the groups in this study was how they were meaningfully involved not just in *receiving* but also in *providing* HIV services [28], as shown in Table 2 and in the intervention section of this paper. This provided them greater visibility and opportunities to interact with their communities, and empowered them to educate their communities and change their stigmatizing values. In that sense, they became agents of social change, as described by Parker and Aggleton [37]: they took active control of their health by collectively resisting factors undermining it. They also leveraged social capital to bridge their acceptability within their communities [38] by engaging in what were seen as “useful” activities, such as income generation and provision of HIV services.

These findings reinforce suggestions by Pulerwitz *et al*. [39] that engaging people living with HIV in programmes could be an effective strategy to reduce HIV stigma. This transformative social and economic participation of people living with HIV as a strategy to counter stigma is supported by evidence from India, Tanzania and Zambia showing that collective efficacy or resistance can improve the ability of marginalized groups to change their situation. Examples of this include sex workers confronting frequent arrests [40] and adolescents with HIV demanding services appropriate to their needs [41, 42].

This is not to suggest that groups of people living with HIV are sufficient alone to eliminate stigma. Rather, multiple approaches are required. Our study confirms that groups of people living with HIV in the two study districts were making a valuable contribution towards reducing stigma via collective efficacy – in effect, a demand-side initiative. However, this should be accompanied by other, supply-side interventions, such as sensitization training for teachers, health service providers, employers, law enforcement personnel, religious leaders and others, for an effective multi-sectoral mitigation of HIV stigma [8, 43, 44]. In addition, the environment in which such groups operate could determine their impact. Our study was conducted in Uganda, which has been hailed as a success in its response to HIV partly due to an “open general environment which allows open discussions surrounding HIV” [45, p. 2]. This may have created an enabling environment for the groups to have an impact.

While our findings suggest that community-based groups of people living with HIV could enable their members to better cope with stigma, the limitations of such groups should be noted. For instance, there is the risk of further alienating groups of people with HIV from their communities through the creation of new notions of social citizenship [46] that could emerge from their collective identity and shared responsibility to sensitize and ‘educate’ others. Roopnaraine *et al*. [35, p. 649] warn that the “problem of stigma inherent in joining groups defined by HIV status” must be carefully balanced with the benefits of such groups.

### Implications for programming and research

These findings have important implications for programming and research. First, they provide a basis for extending current approaches to reducing stigma beyond interventions that seek to increase the resilience and coping mechanisms of *individuals* to those that strengthen the capacity of groups to *collectively* challenge stigma. This could enable people living with HIV who participate in networked groups to leverage social capital, cope with stigma, participate in HIV programmes and enhance their uptake of HIV services [28, 37]. Our findings also inform gender constructs around HIV stigma. Wyrod [47] argues that the inextricable link between the experiences of men with regard to HIV stigma and conceptions of masculinity highlights challenges to, *and opportunities* for, addressing stigma. In our study, societal expectations of men contributed in distinctive ways to their experiences of HIV stigma, suggesting that as HIV programmes in sub-Saharan Africa strive to engage men in HIV care [47, 48], interventions to address HIV stigma should be gender sensitive. This is particularly relevant considering that men in our study were reluctant to join groups, which often prompted creation of men-only groups such as Positive Men's Union (*see* Table 1).

### Limitations

The qualitative nature of our data restricts generalizability, although the study does provide important in-depth insight into the potential of engaging people living with HIV as agents of change in challenging stigma. Our findings relate to two of the 40 districts in which the intervention was implemented, further limiting generalizability of our findings to the remaining districts, especially considering that experiences of stigma could differ between urban and rural contexts. However, our findings could complement those from other stigma studies and stigma index surveys, (for example those that were being conducted by the National Forum for Networks of people living with HIV in Uganda at the time of writing this manuscript), in informing future interventions. Finally, our data did not capture information relating to the process and challenges of setting up groups, which could be valuable in interpreting our findings. Future research should explore long-term impacts of the collective activities of groups of people living with HIV.

## Conclusions

Meaningful engagement of people living with HIV can contribute to interventions to mitigate HIV stigma. Antiretroviral therapy and group-based approaches are opening up new avenues for the collective participation of people living with HIV to change community attitudes towards HIV. Current approaches to reducing stigma should be extended beyond interventions that seek to increase the resilience and coping mechanisms of individuals, to those that build the capacity of groups to collectively challenge stigma.

## References

[1] Sambisa W, Curtis S, Mishra V (2010). AIDS stigma as an obstacle to uptake of HIV testing: evidence from a Zimbabwean national population-based survey. AIDS Care.

[2] Koku EF (2011). Desire for, and uptake of HIV tests by Ghanaian women: the relevance of community level stigma. J Community Health.

[3] Rwemisisi J, Wolff B, Coutinho A, Grosskurth H, Whitworth J (2008). ‘What if they ask how I got it?’ Dilemmas of disclosing parental HIV status and testing children for HIV in Uganda. Health Policy Plan.

[4] Tsai AC, Bangsberg DR, Kegeles SM, Katz IT, Haberer JE, Muzoora C (2013). Internalized stigma, social distance, and disclosure of HIV seropositivity in rural Uganda. Ann Behav Med.

[5] Mahajan AP, Sayles JN, Patel VA, Remien RH, Sawires SR, Ortiz DJ (2008). Stigma in the HIV/AIDS epidemic: a review of the literature and recommendations for the way forward. AIDS.

[6] Akena D, Musisi S, Joska J, Stein DJ (2012). The association between aids related stigma and major depressive disorder among HIV-positive individuals in Uganda. PLoS One.

[7] Simbayi LC, Kalichman S, Strebel A, Cloete A, Henda N, Mqeketo A (2007). Internalized stigma, discrimination, and depression among men and women living with HIV/AIDS in Cape Town, South Africa. Soc Sci Med.

[8] Nattabi B, Li J, Thompson SC, Orach CG, Earnest J (2012). Between a rock and a hard place: stigma and the desire to have children among people living with HIV in northern Uganda. J Int AIDS Soc.

[9] Ogden J, Nyblade L (2005). Common at its core: HIV-related stigma across contexts.

[10] Thomas BE, Rehman F, Suryanarayanan D, Josephine K, Dilip M, Dorairaj VS (2005). How stigmatizing is stigma in the life of people living with HIV: a study on HIV positive individuals from Chennai, South India. AIDS Care.

[11] Sengupta S, Banks B, Jonas D, Miles MS, Smith GC (2011). HIV interventions to reduce HIV/AIDS stigma: a systematic review. AIDS Behav.

[12] Deacon H, Stephney I, Prosalendis S (2005). Understanding HIV/AIDS stigma: a theoretical and methodological analysis.

[13] Castro A, Farmer P (2005). Understanding and addressing AIDS-related stigma: from anthropological theory to clinical practice in Haiti. Am J Public Health.

[14] Reidpath DD, Chan KY, Gifford SM, Allotey P (2005). ‘He hath the French pox’: stigma, social value and social exclusion. Sociol Health Illn.

[15] Hontelez JA, de Vlas SJ, Baltussen R, Newell ML, Bakker R, Tanser F (2012). The impact of antiretroviral treatment on the age composition of the HIV epidemic in sub-Saharan Africa. AIDS.

[16] Campbell C, Skovdal M, Mupambireyi Z, Madanhire C, Robertson L, Nyamukapa CA (2012). Can AIDS stigma be reduced to poverty stigma? Exploring Zimbabwean children's representations of poverty and AIDS. Child Care Health Dev.

[17] Mills EJ, Bakanda C, Birungi J, Chan K, Ford N, Cooper CL (2011). Life expectancy of persons receiving combination antiretroviral therapy in low-income countries: a cohort analysis from Uganda. Ann Intern Med.

[18] Nuwaha F, Kasasa S, Wana G, Muganzi E, Tumwesigye E (2012). Effect of home-based HIV counselling and testing on stigma and risky sexual behaviours: serial cross-sectional studies in Uganda. J Int AIDS Soc.

[19] Wolfe WR, Weiser SD, Leiter K, Steward WT, Percy-de Korte F, Phaladze N (2008). The impact of universal access to antiretroviral therapy on HIV stigma in Botswana. Am J Public Health.

[20] Kipp W, Bajenja E, Karamagi E, Tindyebwa D (2007). AIDS-related stigma: perceptions of family caregivers and health volunteers in western Uganda. World Health Popul.

[21] Brown L, Macintyre K, Trujillo L (2003). Interventions to reduce HIV/AIDS stigma: what have we learned?. AIDS Educ Prev.

[22] Li X, Huang L, Wang H, Fennie KP, He G, Williams AB (2011). Stigma mediates the relationship between self-efficacy, medication adherence, and quality of life among people living with HIV/AIDS in China. AIDS Patient Care STDS.

[23] Midtbø V, Shirima V, Skovdal M, Daniel M (2012). How disclosure and antiretroviral therapy help HIV infected adolescents in sub-Saharan Africa cope with stigma. Afr J AIDS Res.

[24] Campbell C, Skovdal M, Madanhire C, Mugurungi O, Gregson S, Nyamukapa C (2011). “We, the AIDS people …”: how antiretroviral therapy enables Zimbabweans living with HIV/AIDS to cope with stigma. Am J Public Health.

[25] Abimanyi-Ochom J, Lorgelly P, Hollingsworth B, Inder B (2013). Does social support in addition to ART make a difference? Comparison of households with TASO and MOH PLWHA in Central Uganda. AIDS Care.

[26] Hodgson I, Nakiyemba A, Seeley J, Bitira D, Gitau-Mburu D (2012). Only connect – the role of PLHIV group networks in increasing the effectiveness of Ugandan HIV services. AIDS Care.

[27] Mburu G, Iorpenda K, Muwanga F (2012). Expanding the role of community mobilization to accelerate progress towards ending vertical transmission of HIV in Uganda: the Networks model. J Int AIDS Soc.

[28] Kim YM, Kalibala S, Neema S, Lukwago J, Weiss DC (2012). Meaningful involvement of people living with HIV/AIDS in Uganda through linkages between network groups and health facilities: an evaluation study. Psychol Health Med.

[29] Patton M (1990). Qualitative evaluation and research methods.

[30] Bazeley P (2007). Qualitative data analysis with NVivo.

[31] Silverman D (2001). Interpreting qualitative data: methods for analyzing talk text and interaction.

[32] WHO, UNICEF, UNAIDS (2013). Global update on HIV treatment 2013: results, impacts and opportunities.

[33] Tsai AC, Bangsberg DR, Bwana M, Haberer JE, Frongillo EA, Muzoora C (2013). How does antiretroviral treatment attenuate the stigma of HIV? Evidence from a cohort study in rural Uganda. AIDS Behav.

[34] Maughan-Brown B (2010). Stigma rises despite antiretroviral roll-out: a longitudinal analysis in South Africa. Soc Sci Med.

[35] Roopnaraine T, Rawat R, Babirye F, Ochai R, Kadiyala S (2012). “The group” in integrated HIV and livelihoods programming: opportunity or challenge?. AIDS Care.

[36] Hardon A, Gomez GB, Vernooij E, Desclaux A, Wanyenze RK, Ky-Zerbo O (2013). Do support groups members disclose less to their partners? The dynamics of HIV disclosure in four African countries. BMC Public Health.

[37] Parker R, Aggleton P (2003). HIV and AIDS-related stigma and discrimination: a conceptual framework and implications for action. Soc Sci Med.

[38] Gregson S, Mushati P, Grusin H, Nhamo M, Schumacher C, Skovdal M (2011). Social capital and women's reduced vulnerability to HIV infection in rural Zimbabwe. Popul Dev Rev.

[39] Pulerwitz J, Michaelis A, Weiss E, Brown L, Mahendra V (2010). Reducing HIV-related stigma: lessons learned from Horizons research and programs. Public Health Rep.

[40] Reza-Paul S, Lorway R, O'Brien N, Lazarus L, Jain J, Bhagya M (2012). Sex worker-led structural interventions in India: a case study on addressing violence in HIV prevention through the Ashodaya Samithi collective in Mysore. Indian J Med Res.

[41] Carlson M, Brennan RT, Earls F (2012). Enhancing adolescent self-efficacy and collective efficacy through public engagement around HIV/AIDS competence: a multilevel, cluster randomized-controlled trial. Soc Sci Med.

[42] Mburu G, Hodgson I, Teltschik A, Ram M, Haamujompa C, Bajpai D (2013). Rights-based services for adolescents living with HIV: adolescent self-efficacy and implications for health systems in Zambia. Reprod Health Matters.

[43] Otolok-Tanga E, Atuyambe L, Murphy CK, Ringheim KE, Woldehanna S (2007). Examining the actions of faith-based organizations and their influence on HIV/AIDS-related stigma: a case study of Uganda. Afr Health Sci.

[44] Neema S, Atuyambe LM, Otolok-Tanga E, Twijukye C, Kambugu A, Thayer L (2012). Using a clinic based creativity initiative to reduce HIV related stigma at the Infectious Diseases Institute, Mulago National Referral Hospital, Uganda. Afr Health Sci.

[45] Muriisa RK, Jamil I (2011). Addressing HIV/AIDS challenges in Uganda: does social capital generation by NGOs matter?. SAHARA J.

[46] Cataldo F (2008). New forms of citizenship and socio-political inclusion: accessing antiretroviral therapy in a Rio de Janeiro favela. Sociol Health Illn.

[47] Wyrod R (2011). Masculinity and the persistence of AIDS stigma. Cult Health Sex.

[48] Skovdal M, Campbell C, Madanhire C, Mupambireyi Z, Nyamukapa C, Gregson S (2011). Masculinity as a barrier to men's use of HIV services in Zimbabwe. Global Health.

# Sub article: Community-based interventions that work to reduce HIV stigma and discrimination: results of an evaluation study in Thailand

**DOI:** 10.7448/IAS.16.3.18711

**Published:** 2013-11-13

**Authors:** Aparna Jain, Ratana Nuankaew, Nungruthai Mongkholwiboolphol, Arunee Banpabuth, Rachada Tuvinun, Pakprim Oranop na Ayuthaya, Kerry Richter

**Affiliations:** 1 International Center for Research on Women,Washington, DC, USA; 2 Population & Community Development Association (PDA), Bangkok, Thailand; 3 Pact Thailand/Greater Mekong Region, Bangkok, Thailand; 4 Institute for Population and Social Research, Mahidol University, Salaya, Thailand

**Keywords:** evaluation, HIV, AIDS, stigma, discrimination, Thailand, PLHIV

## Abstract

**Introduction:**

HIV stigma and discrimination are major issues affecting people living with HIV in their everyday lives. In Thailand, a project was implemented to address HIV stigma and discrimination within communities with four activities: (1) monthly banking days; (2) HIV campaigns; (3) information, education and communication (IEC) materials and (4) “Funfairs.” This study evaluates the effect of project interventions on reducing community-level HIV stigma.

**Methods:**

A repeated cross-sectional design was developed to measure changes in HIV knowledge and HIV-related stigma domains among community members exposed to the project. Two cross-sectional surveys were implemented at baseline (respondent *n*=560) and endline (respondent *n*=560). *T*-tests were employed to assess changes on three stigma domains: fear of HIV infection through daily activity, shame associated with having HIV and blame towards people with HIV. Baseline scales were confirmed at endline, and each scale was regressed on demographic characteristics, HIV knowledge and exposure to intervention activities.

**Results:**

No differences were observed in respondent characteristics at baseline and endline. Significant changes were observed in HIV transmission knowledge, fear of HIV infection and shame associated with having HIV from baseline to endline. Respondents exposed to three specific activities (monthly campaign, Funfair and IEC materials) were less likely to exhibit stigma along the dimensions of fear (3.8 points lower on average compared to respondents exposed to none or only one intervention; 95% CI: −7.3 to −0.3) and shame (4.1 points lower; 95% CI: −7.7 to −0.6), net of demographic controls and baseline levels of stigma. Personally knowing someone with HIV was associated with low fear and shame, and females were less likely to possess attitudes of shame compared to males.

**Conclusions:**

The multivariate linear models suggest that a combination of three interventions was critical in shifting community-level stigma – monthly campaign, Funfair and IEC materials. This is especially important given Thailand's new national AIDS strategy to reduce HIV-related stigma and discrimination by half by 2016. Knowing which interventions to invest in for HIV stigma reduction is crucial for country-wide expansion and scale-up of intervention activities.

## Introduction

In Thailand, the first reported case of HIV dates back to 1984 [1]. Since that time, the primary mode of transmission has been unprotected sex [2]. In the early 1990s, prevention and control of HIV infections became national priorities, and mass communication campaigns were implemented to increase awareness about HIV and AIDS. Prevalence rates had exceeded 30% among female sex workers by 1995 [3], and the “100% Condom Campaign” was implemented to encourage condom use among all female sex workers and their clients [4]. Widespread condom distribution was instituted throughout the country. Significant progress has been made to curb the spread of HIV; in 2009, the prevalence rate was at 1.3%, with roughly 530,000 living with HIV and an estimated 12,000 new infections [5].

Despite this progress, HIV stigma and discrimination are major issues affecting people living with HIV (PLHIV) in their everyday lives. Worldwide, HIV-related stigma and discrimination are recognized as facilitators in the spread of HIV infections, barriers in the practice of safe and effective HIV-prevention behaviours and significant obstacles in the access of HIV care, treatment and support services [6–9]. HIV stigma and discrimination are the disapproval or devaluation of PLHIV where members of society set PLHIV apart from ordinary activities. In an earlier study conducted in Thailand, researchers found that community members believed that PLHIV should not participate in community activities and should be restricted to their homes [10]. Research in Thailand and Vietnam has shown that the consequences of HIV stigma are severe and may lead to loss of livelihood, refusal of care and depression [11–13]. A 2009 study found that Thai PLHIV have high levels of self-stigma, and that they suffer job loss and refusal of healthcare services, including family planning services [14].

In this study, a repeated cross-sectional design was used to measure increases in HIV knowledge and reductions in HIV-related stigma domains among community members who were exposed to a community-based economic development project. Besides exploring the effect of the project activities on changes in knowledge and stigma, attention is paid to identifying the specific activities that appear to be responsible for the changes.

## Project overview

Launched in 2002, the Population and Community Development Association (PDA) implemented the Positive Partnership Project (PPP), which was designed to economically empower PLHIV, increase their quality of life and reduce the stigma and discrimination they encounter. To economically empower and increase the quality of life of PLHIV, the project provided low-interest loans to a buddy pair consisting of a person living with HIV and an individual without HIV. PLHIV chose their buddy, who was often someone they already knew like friends or family members, and who they had already disclosed their status to. The pair received training to build their skills in marketing, accounting and business management to ensure the success of their commercial endeavours. An additional objective focused on reducing HIV stigma and discrimination within the communities where the loan recipients lived [15]. The project was documented by UNAIDS as a “Best Practice” of PLHIV economic empowerment [16].

Phase II of the project began in April 2008, and two project models were developed and implemented to ensure sustainability beyond the project period: (1) PPP clubs that formed organically by PLHIV support groups; and (2) village development banks (VDBs) that formed mostly by community members and leaders. Both models were responsible for dispersing loans to the buddy pairs, collecting savings, and conducting HIV awareness-raising activities in their communities. Eleven PPP clubs and 12 VDBs were established in 23 communities across six Thai provinces: Bangkok, Chiang Mai, Chiang Rai Chonburi, Khon Kaen and Nakhon Ratchasima.

From September 2009 to September 2010, the project implemented specific HIV-stigma reduction interventions in the communities, including (1) monthly banking days; (2) HIV campaigns; (3) information, education and communications (IEC) materials; and (4) “Funfair” events. The monthly banking days were an important mechanism to continuously mobilize and unite the community. During these days, financial activities for the buddy pairs and other VDB/PPP club members were undertaken (e.g., deposits and loan repayments). In addition, HIV education activities were conducted, including, for example, inviting a PLHIV who was open about his or her status to share experiences with HIV stigma and discrimination. The PPP club and VDB members (consisting of both individuals living with HIV and those without HIV) developed HIV campaigns that were disseminated in their communities. These campaigns were conducted continuously throughout the project period and included activities such as condom distribution as well as household visits to share HIV information, engage community members in discussions around HIV, provide community members opportunities to discuss concerns and doubts, and raise awareness of HIV stigma. The IEC materials were developed based on the baseline survey results and in close collaboration with PLHIV involved in the project. Three IEC materials were developed specifically under this project with key HIV stigma and discrimination messaging. The IEC materials included posters, banking slips with key messages and radio dramas. Examples of key messages include *Being infected with HIV and AIDS is not shameful* and *We should not blame PLHIV and think of them as promiscuous*. Finally, “Funfair” events were held every six months; these were a combination of education and entertainment activities, such as quizzes, role plays and exhibitions.

The paired buddies supported one another to repay their joint loan as well as participate in HIV and AIDS awareness-raising activities with PDA staff and VDB and PPP club committee members. The intervention emphasized contact strategies of working together and supporting one another on these activities to model productive and supportive interactions between individuals living with HIV and those without HIV. These interactions were intended to model positive relationships with PLHIV so that community members could overcome their fears around casual contact with PLHIV and reduce negative attitudes and stereotypes towards PLHIV. These activities were also intended to address the negative attitudes of buddies towards PLHIV and the internalized stigma among PLHIV.

## Methodology

### Data

This study uses community-level surveys that were part of a broader evaluation study aimed at assessing overall project activities, including increases in quality of life of PLHIV, and reductions in HIV stigma and discrimination among buddies. The broader evaluation study implemented surveys with PLHIV involved in the project, the buddies and the family members of project participants (PLHIV and buddies) [17]. The current study only uses data collected from community members. For the community-level survey, the same 11 communities were surveyed, and two cross-sectional surveys were implemented at baseline and endline. A sampling frame of households was developed in each community, and households were selected using systematic random sampling. All individuals 15 years and older were interviewed in each household. Data collection for the baseline and endline survey were conducted from October 2008 to March 2009 and from November 2010 to January 2011, respectively. An equal number of community respondents were interviewed at baseline (*n*=560) and endline (*n*=560).

### Ethical review

The Institutional Review Board at Mahidol University (Salaya, Thailand) reviewed and approved the baseline and endline study designs. Data collectors were trained in implementing the informed consent procedure, and verbal informed consent was obtained from all respondents at baseline and endline.

### Stigma measures

A series of previously validated and tested stigma measures [18, 19] and new items specific to the Thai context were used in the survey. The measures were developed to capture two drivers of stigma: fear of HIV infection and social judgement. Fear of infection items capture fear of HIV transmission in specific casual encounters (e.g., exposure to the saliva or sweat of a PLHIV, or sharing a meal with a PLHIV). The social judgement items include attitude questions related to blaming PLHIV for acquiring the disease and feelings of shame or disgrace associated with having HIV. Fear of infection associated with casual encounters plus social judgement might lead to damaging behaviours, such as avoidance, isolation or gossip.

### Dependent variables

We developed scales using principal component factor analysis to identify uni-dimensional constructs at baseline. The first scale captured fear-based stigma, while the second scale measured attitudes related to shame. These factors were confirmed at endline using confirmatory factor analysis. We tested the reliability of the scales using Cronbach's alpha with a cutoff of 0.7 [20]. Scale validity was assessed, and predicted regression scores were obtained. The scales were standardized to have a mean of 50 and a standard deviation of 10, and they ranged from 0 to 100 where higher scores indicated higher levels of stigma.

### Main predicator

The main predictor was respondents’ reported exposure to the interventions. Four interventions were assessed: (1) monthly meetings on banking days, (2) HIV campaign, (3) IEC materials with key messages (posters, messages on banking slips and radio dramas) and (4) Funfair events. Each intervention was assessed individually and as a dose–response relationship. The dose–response variable is an additive variable of exposure to one, two, three or all four activities. In the analysis, we also identified specific combinations of interventions to predict levels of stigma. We obtained intervention exposure from respondents with both unprompted and prompted questions to determine whether exposure was to PPP project activities or something else. We first asked, for example, “In the past 12 months, have you participated in any Positive Partner Project activities?” If yes, we asked respondents to describe the project activities that they have participated in. If no, we asked, “In the past 12 months, have you ever participated in the following activities?” and then we asked about each activity that was not spontaneously recalled. For the IEC materials, we used a similar process where we first asked respondents to recall specific messages spontaneously. Of the messages that were not recalled spontaneously, we asked them whether they remembered seeing and being exposed to specific messages. Additional predictors of interest included personally knowing someone living with HIV and HIV transmission knowledge.

### Covariates

Study covariates included gender, marital status, age, education, residence, personal income level, occupation and media exposure to HIV messaging. Gender is classified as male or female. A four-category age variable was constructed of 15–29, 30–39, 40–49 and 50+ years old. Marital status was grouped into married versus single, divorced or widowed, and residence was grouped into urban versus rural. Education is classified into no education or primary, and secondary or higher. Personal average monthly income was categorized into four groups: less than 3000 Baht (equivalent to less than $102), 3000–4999 Baht (approximately $102–170), 5000–6999 Baht (approximately $170–238) and 7000 Baht or more ($238 or more). Six occupational types were formed: farmers, small business owners, government employees, factory workers or casual labourers, students, and housewives or unemployed.

We included respondents’ exposure to non-project-related HIV and AIDS information in the past 12 months as another covariate. Four information sources were assessed: radio, television, newspapers and posters. Finally, we created two continuous baseline community-level variables to account for potential differences in community-level stigma before the interventions. We created an average score of fear at baseline in each community surveyed. Across all communities, the fear score ranged from 44.5 to 54.1. A similar variable was generated for baseline community-level shame. The score ranged from 43.0 to 57.0 across all communities.

### Statistical analysis

We compared baseline and endline respondent characteristics and assessed change from baseline to endline in items comprising the fear scale and the shame scale using Wald Chi-square analysis and a two-tailed significance level with a *p*<0.05. Bivariate linear regression analyses for each respondent characteristic and the outcome scales were conducted. Multivariate linear regression models were run of three main predictor variables on the fear and shame scales, adjusted for respondent characteristics and a baseline community average of stigma. All analyses are conducted in STATA.SE, Version 12 [21].

## Results

### Respondent profile

Table 1 presents frequency distributions of respondents’ background characteristics by survey round. A slightly larger proportion of females were interviewed at baseline and endline (58.6% and 58.8%), and an equal proportion of individuals were interviewed in rural and urban areas in both survey rounds. The majority of respondents at baseline and endline were married, were over the age of 40, had less than primary education and were employed as farmers, factory workers or casual labourers. A greater proportion of respondents made 7000 Baht ($238) or more per month at endline when compared to baseline (*p*=0.00). Aside from personal income, there were no statistically significant differences in respondent characteristics at baseline and endline.

**Table 1:** Frequency distributions of respondent characteristics by survey round

	Baseline (*n*=560)	Endline (*n*=560)
Gender		
Female	58.6	58.8
Male	41.4	41.2
Residence		
Urban	50.0	50.0
Rural	50.0	50.0
Marital status		
Married	79.5	75.4
Single, divorced or widowed	20.5	24.6
Age		
15–29	22.5	20.0
30–39	17.3	19.5
40–49	23.0	23.2
50 and above	37.2	37.3
Mean age	43.0	43.7
Median age	44.0	45.0
Education		
None or primary	58.1	55.5
Secondary, high school or vocational	33.9	36.6
University or BA	8.0	7.9
Occupation		
Farmer	23.9	20.7
Small business owner	17.5	17.0
Private or government employee	10.0	11.9
Factory worker or casual labourer	26.1	25.9
Student	5.5	6.8
No occupation, or housewife	17.0	17.7
Average monthly income		
<3000 Baht	37.9	35.6
3000–4999 Baht	21.6	14.6
5000–6999 Baht	18.0	15.2
≥7000 Baht	22.5	34.6

### HIV knowledge

Respondents’ knowledge of HIV transmission, prevention and treatment was assessed at baseline and endline. Across nine questions, correct knowledge increased significantly on the first five questions presented in Figure 1. For example, more respondents at endline knew that all babies do not get HIV from a mother living with HIV and that exposure to the sweat or saliva of a person living with HIV cannot transmit HIV. Low levels of knowledge persisted at endline for the difference between HIV and AIDS at 22.5%. One HIV knowledge question did not change from baseline to endline: 77% of respondents reported that HIV and AIDS is only transmitted among people who inject drugs (PWID), female and male sex workers (F/MSW) and men who have sex with men (MSM) (data not shown).

**Figure 1:** Change in frequency distributions on correct HIV knowledge at baseline and endline.

### Fear of HIV infection and social judgement stigma

The stigma items measured for the fear scale were associated with fear of HIV transmission through casual encounters and everyday contact with PLHIV. The factor loadings and alphas of the fear scale from baseline to endline are presented in Table 2.

**Table 2:** Fear of HIV infection stigma scale: factor loadings and Cronbach's alpha

	Baseline	Endline
Being exposed to saliva of PLHIV	0.752	0.715
Being exposed to sweat of PLHIV	0.772	0.770
Having a meal or sharing food with PLHIV	0.710	0.723
Using the same plate, spoons or forks as PLHIV	0.732	0.733
Taking care of PLHIV	0.726	0.722
Carrying PLHIV	0.734	0.754

Cronbach's alpha	0.83	0.85

Figure 2 presents the change in fear of HIV infection from baseline to endline on six measures. While reductions in fear were observed across all six stigma items, statistically significant declines were seen in four items: (1) exposure to the saliva of an individual living with HIV, (2) sharing cutlery with an individual living with HIV, (3) exposure to the sweat of an individual living with HIV and (4) having a meal (sharing food) with an individual living with HIV. When disaggregated by gender, similar patterns of significance were observed for females and males, except that physically *carrying a PLHIV who is ill* declined significantly among females but not males.

**Figure 2:** Change in percentage of fear in fear of HIV infection stigma items from baseline to endline. **p<0.01; *p<0.05.

Table 3 presents the factor loadings and alphas for the social judgement scales at baseline and endline. Figure 3 presents the percentage of agreement with shame scale items at baseline and endline. At baseline, 39.8% of respondents agreed with the statement that they would feel ashamed if someone in their family had HIV. This dropped slightly to 35.2% at endline (*p*<0.05). A non-statistically significant decline was observed in the other two social judgement stigma measures. The three shame scale items were disaggregated by gender, and results showed declines on all three items among females and males, but significance was observed only across the three items with females (data not shown). Two items were assessed to measure attitudes that blamed individuals for contracting HIV. The distribution frequencies of these two items did not change from baseline to endline (data not shown), and therefore a blame scale was not developed or assessed in the multivariate models.

**Figure 3:** Change in percentage of agreement on social judgement stigma from baseline to endline. **p<0.01; *p<0.05.

**Table 3:** Social judgement stigma scales: factor loadings and Cronbach's alpha

	Baseline		Endline	
		
	Shame	Blame	Shame	Blame
PLHIV should be ashamed of themselves	**0.769**	−0.013	**0.701**	0.046
I would feel ashamed if someone in my family had HIV and AIDS	**0.841**	−0.010	**0.842**	−0.021
It is the promiscuous men who spread HIV in your community	0.006	**0.955**	−0.018	**0.950**
It is the promiscuous women who spread HIV in your community	0.005	**0.954**	0.025	**0.942**
I would feel ashamed if I was infected with HIV	**0.768**	0.046	**0.811**	0.002

Cronbach's alpha	0.71	0.90	0.69	0.88

The bold numbers indicate which factor the item loaded on.

### Intervention exposure

Figures 4 and 5 show frequency distributions of exposure to project interventions. At endline, 10.9% of respondents reported participating in a banking day meeting, 26.6% reported exposure to the HIV campaigns and 17.9% reported participation in a Funfair event. In terms of IEC materials, over half of respondents reported exposure to at least one poster (62.3%), at least one radio drama (65.4%) and at least one message on a banking slip (58.6%; Figure 4). Only 1 in 10 respondents was not exposed to any project activities (10.5%; Figure 5). The majority of respondents were exposed to one activity (52.5%), while only a small proportion was exposed to all four activities (2.9%).

**Figure 4:** Frequency distributions of exposure to specific project interventions among endline respondents (*n*=560).

**Figure 5:** Frequency distributions of exposure to multiple project interventions among endline respondents (*n*=560).

### Multivariate analyses

For both scales, we first ran unadjusted models with each intervention separately and found no significant associations. Then, we looked at the unadjusted dose–response relationship and found that exposure to three interventions was significant for both stigma scales (data not shown). We were interested in identifying the three interventions that yielded these results and learned that respondents who reported participation in or exposure to HIV campaign, Funfair, and IEC scored 5.21 points (95% CI: −8.86 to −1.54) lower on the fear scale and 5.14 points (95% CI: −8.80 to −1.49) lower on the shame scale (data not shown). Next, we adjusted the multivariate linear regression models for fear and shame stigma scales on project intervention exposure, HIV and AIDS knowledge and personal association with someone living with HIV, net of respondent's gender, marital status, age, education, residence, personal income, occupation, media exposure to non-project HIV messages and baseline community average of fear (Table 4). Table 4 shows the beta coefficients and 95% confidence intervals of the main predictors. Models I and II present the dose–response association of the number of project activities exposed to and reductions in fear of HIV infection stigma and social judgement stigma, respectively. The association between participation and exposure to HIV campaign, Funfair and IEC materials remains in the adjusted model for fear (3.81 points lower; 95% CI: −7.32 to −0.30) and shame (4.12 points lower; 95% CI: −7.67 to −0.58).

**Table 4:** Adjusted* multivariate linear regressions of fear of HIV infection stigma and social judgement stigma among endline respondents (*n*=560)

	Model I: fear of HIV infection (fear scale)		Model II: social judgement (shame scale)	
		
	*β*	(95% CI)	*β*	(95% CI)
Intervention exposures				
None or one	Ref		Ref	
Two	−1.13	(−3.12: 0.86)	−0.60	(−2.62: −1.43)
Three (campaign, Funfair and IEC)	−3.81	(−7.32: −0.30)	−4.12	(−7.67: −0.58)
Three (other intervention combinations)	−2.62	(−6.25: 1.02)	−1.24	(−4.93: 2.46)
Four	−0.93	(−5.73: 3.88)	−3.87	(−8.74: 1.01)
HIV and AIDS knowledge				
0–3 correct responses	Ref		Ref	
4–8 correct responses	−4.92	(−6.57: −3.27)	−3.76	(−5.42: −2.09)
Personally know someone living with HIV				
No	Ref		Ref	
Yes	−2.62	(−4.51: −0.72)	−1.89	(−3.80: 0.03)
Constant	66.46	(42.09: 90.84)	42.94	(28.86: 57.02)

* Models were adjusted for respondent's gender, marital status, age, education, residence, personal income, occupation, media exposure to HIV messages and baseline community average of fear.

In terms of gender, no significant differences were observed between females and males on the fear of infection scale. However, females are less likely to possess attitudes of shame compared to males (2.41 points lower; 95% CI: −4.14 to −0.68; data not shown).

The adjusted multivariate linear models also showed that when respondents personally know someone living with HIV, they are more likely to have lower scores on the fear of HIV infection stigma scale (2.62 points lower; 95% CI: −4.51 to −0.72). Higher HIV knowledge at endline predicted lower levels of fear and social judgement. Respondents with higher knowledge [i.e., those who answered 4–8 knowledge questions correctly (58.2%)] scored 4.83 points (95% CI: −6.48 to −3.19) lower on the fear scale and 3.79 points (95% CI:−5.46 to−2.12) lower on the social judgement scale than respondents with lower knowledge. Further analysis showed that the effect of personally knowing someone living with HIV on fear of HIV infection was significant only among those with low HIV knowledge, whereas the effect of high knowledge was significant, regardless of whether or not the respondent knows someone living with HIV (data not shown).

We explored the relationship of HIV knowledge as a potential mediator of the relationship between intervention exposure and fear of HIV infection and social judgement. The results of this analysis suggest that when HIV knowledge was included in the multivariate analysis, it attenuated the observed relationship between intervention exposure and fear and social judgement slightly (regression coefficient reduces from −5.21 to −4.89 for fear and from −5.14 to −4.91 for shame; data not shown).

## Discussion

The results of this study show that participation and exposure in project activities are associated with declines in fear of HIV infection and social judgement stigmas. The dose–response relationship assumes that different levels of exposure influence changes in fear of HIV infection and social judgement stigmas. While incremental changes in the outcome were not observed with each additional exposure, three interventions were identified as necessary for addressing fear of HIV infection and social judgement stigmas in Thailand. The three interventions – HIV campaigns, IEC materials and the Funfair – provide information about what actions and behaviours are stigmatizing, the consequences of stigma experienced by a person living with HIV, resources for treatment and care, and methods to prevent transmission, among other information. These three interventions communicate these types of information through various modes, including opportunities for community members to receive answers to questions and alleviate doubts (through the household visits and banking days), personal interactions with PLHIV (VDB and PPP clubs), anti-HIV stigma messaging reinforced through a wide array of communication modes (posters, banking slips and radio dramas) and hosted events that engaged the community in fun activities such as games and quizzes in addition to staging role-plays that addressed these issues (Funfairs). As documented elsewhere [20] and revealed in this study, by addressing stigma through a combination of activities, individuals were offered a variety of mechanisms and opportunities to be exposed to HIV stigma-reduction issues, which resulted in reductions in fear of HIV transmission and stigmatizing attitudes.

Our findings demonstrate that although community members’ knowledge of HIV transmission, prevention and treatment increased significantly on certain items, there is still considerable work to be done to improve knowledge levels. Roughly one in two individuals in our endline sample did not know that HIV cannot be transmitted through sweat or saliva, and one in four individuals did not know that HIV cannot be transmitted through skin contact. The study findings also show that increasing HIV knowledge may be an initial stage in addressing fear of HIV transmission and stigmatizing attitudes. The links between increase in HIV knowledge and decrease in fear, and increase in HIV knowledge and decrease in negative attitudes, have been demonstrated in Chiang Rai, Thailand [22] and Ethiopia [23]. Once individuals possess correct information about how HIV can and cannot be transmitted, fears of HIV infection in daily interactions with PLHIV can be diminished. Further analysis of knowledge as a potential mediator suggests that intervention exposure, in part, influences stigma through HIV knowledge but does not fully explain the effect of intervention activities on stigma.

The multivariate analysis revealed that individuals who know someone living with HIV have less fear of HIV transmission and less stigmatizing attitudes towards PLHIV. This has been shown in Thailand elsewhere [24, 25] and in several other studies [23, 26]. Researchers of a multi- country study found a negative correlation between HIV prevalence and negative attitudes towards PLHIV [27], and concluded that contact with PLHIV is more common in high-prevalence settings because HIV is normative, which might influence attitudes. While our study was in a low-prevalence setting, contact with PLHIV reduces stigma only among those who have low HIV knowledge. The effect of high knowledge on stigma, however, is significant, regardless of whether or not the respondent knows someone living with HIV, suggesting that knowledge has a greater influence on fear of HIV infection stigma than knowing someone living with HIV.

There are several limitations that need to be considered while interpreting the results. First, no control communities were included in the original design of the study. Therefore, we cannot claim that the interventions are responsible for the observed changes in HIV stigma. In our analysis, however, we did adjust for exposure to other HIV messaging that might have coincided with the intervention activities. Second, we were unable to account for correlation among individuals residing in the same household in this analysis. Also, the analysis does not reflect the level of participation in interventions or intensity of exposure. Finally, the results may also be subject to social desirability given that this topic is highly sensitive. The estimates on the fear and shame scales might be an underestimate if respondents are unwilling to share stigmatizing attitudes in one-on-one interviews.

## Conclusions

The purpose of this study was to determine changes in HIV knowledge and negative attitudes towards PLHIV among community members exposed to the PPP project. The results of this study suggest that programmes that choose to focus on HIV-related stigma reduction need to address the issue in multiple ways. Developing three interventions – Funfair, IEC materials and the HIV campaign (condom distribution and household visits) – led to a shift in knowledge and attitudes associated with fear of HIV and shame. Programmes also need to address multiple domains of stigma – knowledge, fear, shame and blame –simultaneously while recognizing that blame is a harder construct to reduce. Social judgement stigma is a harder construct to shift, as also shown in a Vietnam study [19] where interventions reduced fear and shame but the reductions in shame were smaller. Social judgement stigma tends to be deep rooted, and for future programming a longer intervention period may be needed. Also, specific interventions designed to tackle blame may be more effective. For example, programme might develop safe, nonthreatening spaces for honest and open discussion among individuals to better understand what drives these blaming attitudes.

This research is especially important given Thailand's new national AIDS strategy aligned with the UNAIDS vision of “getting to zero” – zero new HIV infections, zero discrimination and zero AIDS-related deaths. One of the goals of this new strategy is to reduce HIV-related stigma and discrimination by half by 2016 [28]. The findings of this study provide a good starting point for the programme to consider expanding to achieve this goal.

## References

[1] Thailand Ministry of Public Health (MOPH) (1984). Acquired immunodefciency syndrome (AIDS) – the first reported case in Thailand. Wkly Epidemiol Surveill Rep.

[2] Thailand Ministry of Public Health (MOPH) (2012). Thailand AIDS response progress report 2012: status at a glance. http://www.aidsdatahub.org/dmdocuments/UNGAA_2012_Thailand_Narrative_Report.pdf.

[3] UNAIDS (1998). Relationships of HIV and STD declines in Thailand, a synthesis of existing studies.

[4] Rojanapithayakorn W, Hanenberg R (1996). The 100% condom program in Thailand. AIDS.

[5] World Health Organization (2010). Global report: UNAIDS report on the global AIDS epidemic.

[6] Brown L, Macintyre K, Trujillo L (2003). Interventions to reduce HIV/AIDS stigma: what have we learned?. AIDS Educ Prev.

[7] Kalichman SC, Simbayi LC (2003). HIV testing attitudes, AIDS stigma, and voluntary HIV counselling and testing in a black township in Cape Town, South Africa. Sex Transm Infect.

[8] Mahajan AP, Sayles JN, Patel VA, Remien RH, Ortiz D, Szekeres G (2008). Stigma in the HIV/AIDS epidemic: a review of the literature and recommendations for the way forward. AIDS.

[9] Nyblade L, Stangl A, Weiss E, Ashburn K (2009). Combating HIV stigma in health care settings: what works?. J Int AIDS Soc.

[10] Songwathana P, Manderson L (2001). Stigma and rejection: living with AIDS in villages in southern Thailand. Med Anthropol.

[11] Liamputtong P, Haritavorn N, Kiatying-Angsulee N (2009). HIV and AIDS, stigma and AIDS support groups: perspectives from women living with HIV and AIDS in central Thailand. Soc Sci Med.

[12] Thi MDA, Brickley DB, Vinh DTN, Colby DJ, Sohn AH, Trung NQ (2008). A qualitative study of stigma and discrimination against people living with HIV in Ho Chi Minh City, Vietnam. AIDS Behav.

[13] Li L, Lee S, Thammawijaya P, Jiraphongsa C, Rotheram-Borus MJ (2009). Stigma, social support, and depression among people living with HIV in Thailand. AIDS Care.

[14] Thai Network of People Living with HIV/AIDS (TNP+) (2009). Index of stigma and discrimination against people living with HIV/AIDS in Thailand. http://www.aidsdatahub.org/dmdocuments/Stigma_Index_Thailand.pdf.

[15] Viravaidya M, Wolf R, Guest P (2008). An assessment of the Positive Partnership Project in Thailand: key considerations for scaling-up micro credit loans for HIV-positive and negative pairs in other settings. Glob Public Health.

[16] UNAIDS (2007). The Positive Partnerships Program in Thailand: empowering people living with HIV.

[17] Richter K, Nuankaew R, Jain A, Ayuthaya PO (2011). Positive Partnership Project, new phase. Endline study report.

[18] Nyblade L, MacQuarrie K, Phillip F, Kwesigabo G, Mbwambo J, Ndega J (2005). Working report measuring HIV stigma: results of a field test in Tanzania.

[19] Nyblade L (2008). Communities confront HIV stigma in Viet Nam: participatory interventions reduce HIV-related stigma in two provinces.

[20] DeVellis RF (2011). Scale development: theory and applications.

[21] StataCorp (2011). Stata statistical software: release 2012.

[22] Boer H, Emons P (2004). Accurate and inaccurate HIV transmission beliefs, stigmatizing and HIV protection motivation in northern Thailand. AIDS Care.

[23] Lifson A, Demissie W, Tadesse A, Ketema K, May R, Yakob B (2012). HIV/AIDS stigma-associated attitudes in a rural Ethiopian community: characteristics, correlation with HIV knowledge and other factors, and implications for community intervention. BMC Int Health Hum Right.

[24] Takai A, Wongkhomthong SA, Akabayashi A, Kai I, Ohi G, Naka K (1998). Correlation between history of contact with people living with HIV AIDS PWAs and tolerant attitudes toward HIV AIDS and PWAs in rural Thailand. Int J STD AIDS.

[25] Genberg BL, Kawichai S, Chingono A, Sendah M, Chariyalertsak S, Konda KA (2008). Assessing HIV/AIDS stigma and discrimination in developing countries. AIDS Behav.

[26] Visser MJ, Makin JD, Lehobye K (2006). Stigmatizing attitudes of the community towards people living with HIV/AIDS. J Community Appl Soc Psychol.

[27] Genberg BL, Hlavka Z, Konda KA, Maman S, Chariyalertsak S, Chingono A (2009). A comparison of HIV/AIDS-related stigma in four countries: negative attitudes and perceived acts of discrimination towards people living with HIV/AIDS. Soc Sci Med.

[28] National AIDS Committee (2012). National AIDS strategy for 2012–2016 in brief.

# Sub article: Implementing a stigma reduction intervention in healthcare settings

**DOI:** 10.7448/IAS.16.3.18710

**Published:** 2013-11-13

**Authors:** Li Li, Chunqing Lin, Jihui Guan, Zunyou Wu

**Affiliations:** 1 Semel Institute for Neuroscience and Human Behavior, Center for Community Health, University of California at Los Angeles Los Angeles, CA, USA; 2 Fujian Provincial Center for AIDS/STD Control and Prevention, Fuzhou, China; 3 National Center for AIDS/STD Control and Prevention, Chinese Center for Disease Control and Prevention, Beijing, China

**Keywords:** HIV-related stigma, implementation, China, intervention

## Abstract

**Introduction:**

Globally, HIV-related stigma is prevalent in healthcare settings and is a major barrier to HIV prevention and treatment adherence. Some intervention studies have showed encouraging outcomes, but a gap continues to exist between what is known and what is actually delivered in medical settings to reduce HIV-related stigma.

**Methods:**

This article describes the process of implementing a stigma reduction intervention trial that involved 1760 service providers in 40 hospitals in China. Guided by Diffusion of Innovation theory, the intervention identified and trained about 15–20% providers as popular opinion leaders (POLs) to disseminate stigma reduction messages in each intervention hospital. The intervention also engaged governmental support in the provision of universal precaution supplies to all participating hospitals in the trial. The frequency of message diffusion and reception, perceived improvement in universal precaution practices and reduction in the level of stigma in hospitals were measured at 6- and 12-month follow-up assessments.

**Results:**

Within the intervention hospitals, POL providers reported more frequent discussions with their co-workers regarding universal precaution principles, equal treatment of patients, provider-patient relationships and reducing HIV-related stigma. Service providers in the intervention hospitals reported more desirable intervention outcomes than providers in the control hospitals. Our evaluation revealed that the POL model is compatible with the target population, and that the unique intervention entry point of enhancing universal precaution and occupational safety was the key to improved acceptance by service providers. The involvement of health authorities in supporting occupational safety was an important element for sustainability.

**Conclusions:**

This report focuses on explaining the elements of our intervention rather than its outcomes. Lessons learned from the intervention implementation will enrich the development of future programs that integrate this or other intervention models into routine medical practice, with the aim of reducing HIV-related stigma and improving HIV testing, treatment and care in medical settings.

## Introduction

Several decades into the HIV pandemic, HIV-related stigma continues to be a major challenge to prevention and treatment efforts worldwide [1–5]. Stigma in the general population has been well-documented, but its impact is also felt in healthcare settings [6, 7], where it can lead to testing avoidance, barriers to health counselling and a lack of adherence to antiretroviral therapies [8–10]. There is an urgent need for intervention efforts focused on reducing HIV-related stigma and discrimination, especially among frontline health service providers.

Globally, there has been substantial research on HIV-related stigma in healthcare settings. Previous studies have identified factors associated with stigma among service providers, including a lack of knowledge, fear related to the incurability of AIDS and prejudice toward marginalized behaviours [11, 12]. Our previous work identified a lack of institutional support and self-protection supplies as major reasons for avoiding service for people living with HIV in China [13, 14]. In 2009, Nyblade and colleagues conducted a literature review that identified strategies to combat stigma in healthcare facilities; their recommendations included using a participatory method, involving people living with HIV and training service providers on universal precautions [3]. Some intervention programs and activities tested in small-scale studies have shown encouraging outcomes [1, 15, 16]. For example, an intervention combining AIDS knowledge dissemination and contact with people living with HIV among 102 nursing students showed enhanced emotional competence to serve people living with HIV [16]. Our study team also conducted an intervention pilot in 2006 among 138 providers from four county hospitals in Yunnan, China. During the intervention delivery, people living with HIV acted as intervention trainers to share their experiences and facilitate discussions. Preliminary findings showed that provider participants in the intervention group reported better protection of patients’ confidentiality and lower levels of negative feelings toward people living with HIV [14]. However, one of the most critical issues impeding stigma reduction today is the gap between what is known about stigma and the systematic utilization of the evidence in full-scale intervention efforts in healthcare settings [17].

In light of our exploratory study findings and the promising outcomes of the pilot work [13, 14, 18], we implemented a randomized intervention trial that involved 1760 service providers in 40 hospitals in two provinces of China, with the objective to reduce service providers’ stigmatizing attitudes and behaviours towards people living with HIV in healthcare settings. The intervention efficacy was reported in another article published by the research team [19]. Rather than present the intervention outcomes, this article focuses on describing the implementation procedures in detail, reporting operational outcomes and sharing lessons learned.

## Methods

### Intervention framework

The intervention was designed using Diffusion of Innovation theory [20]. Instead of training every service provider in the hospitals, we identified, recruited and trained a subset of providers as popular opinion leaders (POLs) to communicate intervention messages to peers during everyday conversation and worked with them to sustain their advocacy activities [21]. The POL model has successfully been used to improve the quality of care by service providers in the United States [22, 23]. Our previous studies showed that the POL model is also applicable for service providers in China, given they are a stable population with an established network, and that some active and respected providers could potentially be effective change agents in stigma reduction within their professional community [18].

### Site selection and randomization

The study was conducted in Fujian and Yunnan provinces, China, from October 2008 to February 2010. These two provinces were selected because they represent the varied HIV rates and infection routes seen in the country [24, 25]. County hospitals were included because they are public facilities and easily accessed by most Chinese residents, and they are where many HIV infection cases are first detected. A total of 40 county hospitals were randomly selected from the 214 eligible hospitals in the two provinces.

The 20 hospitals from each province were first matched as pairs under comparable conditions such as number of beds, size of service provider staff, medical services offered and number of patients with HIV infection. After the baseline assessment, the two hospitals in each pair underwent a randomization process to assign them to an intervention group or control group. The geographic distance between the intervention and control hospitals was considered to avoid potential contamination.

### Identification of potential POL providers

To reach the goal of social norm shifting within the hospital, we targeted approximately 20 to 25 POLs from each intervention hospital, which covered about 15–20% of all providers [21, 26]. POL service providers in this study were deemed trustworthy, influential and reliable by their coworkers. Most importantly, the POL providers had to express care for their hospital and be willing to make an effort to improve the service quality of the facility. Three strategies were used to identify the POLs: 1) recommendations from department heads in the hospitals; 2) recommendations from co-workers; and 3) observations of the study's research staff.

The process was carried out as follows: department heads and other hospital administrators nominated persons they knew to be socially influential; then, the randomly selected providers who participated in the baseline assessment were asked to nominate the three most popular and influential providers in their hospital; and finally, our research staff observed the potential candidates’ interactions with their coworkers in order to verify the popularity of nominees and the strength of their social networks. To maintain balance and wide coverage, POLs were chosen from multiple departments in order to achieve broad coverage within each facility. The POL providers were recruited in two waves from each hospital, with about 10 to 13 POLs in each wave. The POLs in the first wave also participated in nominating POLs in the second wave.

### Recruitment of POL providers

The project recruiters approached potential POL providers after they were identified. Recruiters introduced the intervention as an opportunity to improve the POLs’ medical community, emphasizing that they were selected because of their influence and trustworthiness among colleagues. POLs were informed of their ethical rights, counselled on voluntary informed consent and invited to attend four weekly training sessions and bi-monthly reunion sessions. The refusal rate for POLs was less than 3%.

### Training of POL providers

Intervention facilitation teams were formed in both Fujian and Yunnan. The team consisted of local health educators, AIDS specialists and project staff. Prior to the intervention, all facilitators were given thorough training regarding institutional review board procedures, facilitator roles and responsibilities, intervention skills and protocol for emergency situations.

The selected POL providers attended four weekly group training sessions over a one-month period. Each session lasted about 1.5 hours and was held in a conference room at the county hospital where the providers worked. The participants were seated in a circle so that the facilitators could make eye contact with every person in the group. The titles of the four sessions were: 1) Complying with Universal Precaution Procedures and Ensuring Occupational Safety; 2) Fighting Against Stigma and Improving the Provider-Patient Relationship; 3) Taking Actions and Making Efforts to Care for Patients; and 4) Overcoming Difficulties and Building Up a Better Medical Environment. The intervention incorporated engaging activities such as discussion, games and role playing to encourage the trainees’ full participation. For example, a game called “Rescue Mission” conveyed the message of equal medical treatment of everyone regardless of their social status, type of disease, or infection route; and in a group discussion, “Discrimination Around Us,” providers were asked to identify discriminatory language and behaviours, especially in medical settings. Local elements were incorporated into the intervention materials. For example, local HIV-positive advocates and AIDS specialist stories were made into videos and demonstrated in the intervention sessions as real-life examples. The POLs received a small gift for each session of training activities they attended. The local teams selected inexpensive items, such as a pen or key chain, as a token of appreciation for their time and participation. To ensure the fidelity of intervention delivery, project evaluators observed every intervention session, assessed the quality of the intervention with a checklist and provided suggestions for improvement after each session.

### Dissemination of intervention messages from POL to peer providers

POL providers were encouraged to deliver intervention messages to their co-workers. The messages revolved around universal precautions and occupational safety, equal treatment of all patients, improvement of the provider-patient relationship and reduction of HIV-related stigma. To ensure broad message diffusion, POL providers were encouraged to talk to their coworkers not only within the same department, but also from other departments. Interactive techniques such as facilitator demonstration, group discussion, pair sharing and role playing were used to refine each POL's communication skills so that they could comfortably deliver messages. At the end of each intervention session, the POLs set goals to engage in conversations with coworkers, and the conversational outcomes were reviewed and discussed at subsequent sessions.

To ensure the sustainability of message dissemination, three reunion sessions were conducted after completion of the four initial training sessions. The first reunion was conducted one month after the initial training, while the second and the third reunions occurred four months after the previous reunion. The reunion sessions focused on group sharing, continued problem solving and skill building. For message delivery, the POLs reported in detail who they communicated with, under what circumstances, the contents of the conversation, challenges encountered and possible solutions. A group discussion about ways to improve the message delivery followed each POL's report.

### Provision of universal precaution supplies

To make structural changes in accessibility to supplies for self-protection, both intervention and control hospitals received information packages on general safety in medical procedures and universal precaution supplies from the National Center for AIDS/STD Control and Prevention (NCAIDS), Chinese Center for Disease Control and Prevention (CCDC). A Universal Precaution Oversight Committee was organized in each hospital under the supervision of the Infection Control Department to manage the dissemination of the supplies. Two or three volunteers from each hospital were assigned the role of supply managers; the supplies were distributed to the departments based on necessity; and supply managers were expected to report a shortage of universal precaution supplies to the Oversight Committee when necessary.

### Evaluation

At baseline, 44 providers were randomly selected from a publicly available staff roster of each participating hospital (total sample size=1760). In order to be eligible for the study, potential participants had to be aged 18 or above and work as a service provider (i.e., doctor, nurse, or lab technician) who had regular contact with patients. The POLs who were trained were not necessarily included in the assessments. At the time of recruitment, research staff followed a standardized script to explain the purpose of the study, procedures, confidentiality, voluntary participation and potential risks and benefits. Written informed consent was obtained prior to the data collection and study activities. The refusal rate was as low as 3%, and the follow-up rate was higher than 99% in both the intervention and control hospitals. At each assessment, providers completed a self-administrated paper-and-pencil questionnaire in a private room, with a trained interviewer available to answer questions. The survey took an average of 30 to 45 minutes to complete. Participants received 50 yuan (U.S. $8.00) for each assessment. All study documents and procedures were approved by the Institutional Review Boards of the University of California, Los Angeles, and the NCAIDS, CCDC.

Background information such as age, gender, profession and prior experience in treating people living with HIV was collected. The providers in the intervention hospital reported the frequency of intervention message diffusion and reception during the past six months at the 6- and 12-month follow-up assessments. Providers in all hospitals (both intervention and control) reported their perceived change in terms of universal precaution compliance, equal treatment of patients, provider-patient relationship and reduction in HIV-related stigma.

### Data analysis

Data were analyzed using SAS System for Windows (Version 9.2). We descriptively reported the background characteristics of POL providers in the sample. The times of message dissemination and reception during the past six months were compared between POL and non-POL providers in the intervention hospitals using a *t*-test; the perceived improvement in the hospital was compared between the intervention and control groups with a Chi-square test.

## Results

### Characteristics of POL providers

A total of 456 POL providers were included in the sample, the majority of whom were women (69.3%). The average age of the POL providers was 37.2 years at baseline. About one-third (38.6%) of providers had obtained an undergraduate medical degree or above. Two-thirds of the POL providers had worked in the medical field for more than 10 years. Slightly less than half (44.4%) of the POL providers were doctors, and 44.08% were nurses. The POLs were distributed among several departments: surgery, internal medicine, obstetrics-gynaecology, laboratory, emergency, STD and dermatology, otolaryngology, infectious diseases and paediatrics. Approximately 60% of POLs had prior contact with people living with HIV (Table 1).

**Table 1:** Characteristics of POL providers at baseline

	Number (Total=456)	%
Age (Mean=37.16, SD=8.35)		
Equal to or less than 30 years	91	19.96
31 to 40 years	206	45.17
41 years and above	159	34.87
Female	316	69.30
Medical education		
Vocational high school or below	91	19.96
Associate medical degree	187	41.01
Undergraduate medical degree or above	176	38.60
Years of medical service (Mean=15.04, SD=8.63)		
Equal to or less than 10 years	147	32.24
11 to 20 years	185	40.57
21 years and above	124	27.19
Profession		
Doctor	216	47.37
Nurse	201	44.08
Others	39	8.55
Department		
Surgery	99	21.71
Internal medicine	87	19.08
Obstetrics-Gynaecology (OBGYN)	86	18.86
Laboratory	37	8.11
Emergency	32	7.02
STDs and dermatology	29	6.36
Otolaryngology	24	5.26
Infectious diseases	23	5.04
Paediatrics	11	2.41
Others	28	6.14
Previous contact with people living with HIV	274	60.09

### Message dissemination and reception in intervention hospitals

Within the intervention hospitals, the POL providers reported more frequent message diffusion than non-POLs. For POLs, the average time spent discussing universal precaution compliance during the past six months was 9.29 minutes at the 6-month assessment and 10.45 minutes at the 12-month assessment, respectively. Conversely, the number was only 4.58 minutes at the 6-month assessment and 5.54 minutes at the 12-month assessment for non-POLs (*p*<0.0001 for both assessments). POLs disseminated messages of reducing HIV-related stigma at more than double the rate of non-POLs (29.45% vs. 10.21% at 6-months; 32.75% vs. 13.51% at 12-months). The POLs also discussed equal treatment of all patients and how to improve the provider-patient relationship significantly more often than non-POLs, at both the 6- and 12-month follow-up assessments (*p*<0.0001). In general, the message diffusion was more frequent at the 12-month than the 6-month assessment (Table 2).

**Table 2:** Message dissemination among intervention hospital providers

	6-Month follow up			12-Month follow up		
		
	Non-POL	POL		Non-POL	POL	
				
	N (%)	N (%)	*p* *	N (%)	N (%)	*p* *
In the past six months, how many times have *you talked to other providers in hospital* about …						
1. Universal precaution and occupational safety						
Mean±SD	4.58±7.28	9.29±9.65	<.0001	5.54±5.96	10.45±13.06	<.0001
0–2 times	182 (43.23)	77 (16.92)		142 (33.65)	64 (14.07)	
3–9 times	185 (43.94)	208 (45.71)		204 (48.34)	206 (45.27)	
10 times and above	54 (12.83)	170 (37.36)		76 (18.01)	185 (40.66)	
2. Equal treatment to all patients						
Mean±SD	4.13±7.37	8.27±9.54	<.0001	5.22±6.78	9.48±12.65	<.0001
0–2 times	217 (51.67)	94 (20.66)		165 (39.10)	95 (20.88)	
3–9 times	161 (38.33)	214 (47.03)		194 (45.97)	201 (44.18)	
10 times and above	42 (10.00)	147 (32.31)		63 (14.93)	159 (34.95)	
3. Improving provider-patient relationship						
Mean±SD	6.00±9.49	9.07±9.57	<.0001	6.57±8.08	10.27±12.34	<.0001
0–2 times	161 (38.24)	80 (17.58)		117 (27.73)	79 (17.36)	
3–9 times	177 (42.04)	202 (44.40)		218 (51.66)	193 (42.42)	
10 times and above	83 (19.71)	173 (67.58)		87 (20.62)	183 (40.22)	
4. Reducing HIV-related stigma						
Mean±SD	3.76±6.77	7.55±8.36	<.0001	4.78±5.41	8.25±9.59	<.0001
0–2 times	229 (54.39)	112 (24.62)		170 (40.28)	108 (23.74)	
3–9 times	149 (35.39)	209 (45.93)		195 (46.21)	198 (43.52)	
10 times and above	43 (10.21)	134 (29.45)		57 (13.51)	149 (32.75)	

* Two sample *t*-test.

The POLs also reported more reception of intervention messages from other providers in the hospital. At the 12-month assessment, POLs reported that peer providers in their hospital had talked to them an average of 10.25 times about universal precaution and occupational safety, 8.94 times about equal treatment, 10.13 times about improving provider-patient relationships and 8.31 times about reducing HIV-related stigma; while for non-POLs the numbers were 6.40, 5.41, 7.18 and 5.24, respectively (*p*<0.0001) (Table 3). There was no significant difference in message dissemination or reception between male and female providers.

**Table 3:** Message reception among intervention hospital providers

	6-Month follow up			12-Month follow up		
		
	Non-POL	POL		Non-POL	POL	
				
	N (%)	N (%)	*p* *	N (%)	N (%)	*p* *
In the past six months, how many times have *other providers in hospital talked to you* about …						
1. Universal precaution and occupational safety						
Mean±SD	5.38±7.54	8.15±8.01	<.0001	6.40±7.06	10.25±11.70	<.0001
0–2 times	153 (36.34)	63 (13.85)		105 (24.88)	56 (12.31)	
3–9 times	189 (44.89)	228 (50.11)		228 (54.03)	200 (43.96)	
10 times and above	79 (18.76)	164 (36.04)		89 (21.09)	199 (43.74)	
						
2. Equal treatment to all patients						
Mean±SD	4.46±7.05	7.08±6.77	<.0001	5.41±5.74	8.94±10.97	<.0001
0–2 times	196 (46.56)	104 (22.86)		147 (34.83)	81 (17.80)	
3–9 times	166 (39.43)	221 (48.57)		197 (46.68)	221 (48.57)	
10 times and above	59 (14.01)	130 (18.57)		78 (18.48)	153 (33.63)	
3. Improving provider-patient relationship						
Mean±SD	6.59±10.00	8.81±9.19	0.0006	7.18±8.94	10.13±12.80	<.0001
0–2 times	147 (34.92)	86 (18.90)		103 (24.41)	66 (14.51)	
3–9 times	181 (42.99)	210 (46.15)		207 (49.05)	209 (45.93)	
10 times and above	93 (22.09)	159 (34.95)		112 (26.54)	180 (39.56)	
4. Reducing HIV-related stigma						
Mean±SD	3.89±6.64	6.94±8.04	<.0001	5.24±6.22	8.31±11.14	<.0001
0–2 times	226 (53.68)	112 (24.62)		156 (36.97)	111 (24.40)	
3–9 times	156 (37.05)	231 (50.77)		199 (47.16)	202 (44.40)	
10 times and above	39 (9.26)	112 (24.62)		67 (15.88)	142 (31.21)	

* Two sample *t*-test.

### Distribution of universal precaution supplies

During the 12-month follow-up period, each of the 40 participating hospitals received 100 disposable sharp containers, 50 disposable cloths, 50 disposable waterproof aprons, 15 pairs of protection goggles and 100 pairs of rubber gloves. The amount of supply distribution was the same for the intervention and control hospitals. For the hospitals, this was the first time to see a gesture from the government to promote universal precaution practice.

### Perceived improvement in the hospitals

Compared to the control group, the intervention hospital providers perceived more improvement in universal precaution and occupational safety, equal treatment of all patients, provider-patient relationship and reduction in HIV-related stigma. For example, more than half (55.19%) of the providers in the intervention hospitals reported significant improvement in universal precaution and occupational safety in their hospitals at the 12-month assessment, while only 28.18% of the control hospital providers felt that way. The proportion of the intervention providers who perceived a significant reduction in HIV-related stigma at the 12-month assessment was more than double the number among the control providers (45.50 vs. 20.68%). The perceived improvement was sustained and augmented at 12 months (Table 4).

**Table 4:** Perception of improvement in the hospital

	6-Month follow up			12-Month follow up		
		
	Control	Intervention		Control	Intervention	
				
	N (%)	N (%)	*p* *	N (%)	N (%)	*p* *
In the past six months, do you think *your hospital has improvement* in terms of …						
1. Universal precaution and occupational safety						
Significantly	241 (27.48)	376 (42.92)	<.0001	248 (28.18)	484 (55.19)	<.0001
Some	547 (63.37)	463 (52.85)		557 (63.30)	368 (41.96)	
No improvement	51 (5.82)	20 (2.28)		45 (5.11)	10 (1.14)	
No judgment	38 (4.33)	17 (1.94)		30 (3.41)	15 (1.71)	
2. Equal treatment to all patients						
Significantly	216 (24.63)	307 (35.05)	<.0001	224 (25.45)	405 (46.18)	<.0001
Some	538 (61.35)	501 (57.19)		562 (63.86)	434 (49.49)	
No improvement	65 (7.41)	25 (2.85)		57 (6.48)	12 (1.37)	
No judgment	58 (6.61)	43 (4.91)		37 (4.20)	26 (2.96)	
3. Provider-patient relationship						
Significantly	366 (41.73)	394 (44.98)	0.2994	364 (41.36)	489 (55.76)	<.0001
Some	449 (51.20)	431 (49.20)		459 (52.16)	355 (40.48)	
No improvement	38 (4.33)	26 (2.97)		38 (4.32)	19 (2.17)	
No judgment	24 (2.74)	25 (2.85)		19 (2.16)	14 (1.60)	
4. Reducing HIV-related stigma						
Significantly	188 (21.44)	273 (31.16)	<.0001	182 (20.68)	399 (45.50)	<.0001
Some	477 (54.39)	488 (55.71)		513 (58.30)	410 (46.75)	
No improvement	109 (12.43)	43 (4.91)		103 (11.70)	24 (2.74)	
No judgment	103 (11.74)	72 (8.22)		82 (9.32)	44 (5.02)	

* Chi-square test.

## Discussion

This article describes the process of implementing a large-scale stigma reduction intervention trial in general health settings in China. This study has limitations. For example, the frequency of message diffusion relied on self-reports, making social-desirability bias a concern. Also, as the POLs took part in the intervention, they might be more sensitive to the intervention messages and tend to report more frequent message dissemination than non-POLs. Additionally, we were not able to measure the real usage of universal precaution supplies in the facilities. In spite of these limitations, we learned a number of lessons in the course of implementing the project.

There were some difficulties we encountered during the POL training. First, the provider participants all had busy work schedules. To ensure that all POLs could participate, the field staff communicated with the POL participants beforehand to seek their opinion on the preferred time for conducting sessions. The sessions were usually conducted in late afternoons after work or during midday breaks. Second, some providers were not used to the interactive format and reluctant to talk at the beginning, so the facilitators re-emphasized that there was no right or wrong answer, and encouraged the participants by giving positive reinforcement and recognition throughout the sessions to prompt optimum sharing. Third, some POL providers insisted that no stigma exists in their facility, or that people living with HIV deserved to be discriminated against because of their “immoral” behaviours. In these cases, the facilitators still showed respect for the participants and used games and group discussion to address their attitudes.

The reunion sessions proved to be an important platform to share experiences and skill building among POL providers, and also served as a source for feedback collection for the researchers. During reunion sessions, the POL providers reported that they conveyed stigma reduction message not only in words but in their personal actions, and the messages were generally well-accepted by their audience. Some POL providers encountered peers who perceived HIV to be far-removed from their lives and the topic was irrelevant, especially in areas with low HIV prevalence, but their perception and awareness of the issue could be changed through repeated conversation.

One lesson we learned from this study was to find a unique entry point when implementing the intervention. It is genuinely challenging to engage service providers in a stigma reduction intervention because they are regarded as experts in the medical field. Instead of solely disseminating knowledge and identifying stigmatizing attitudes and behaviours, the intervention addressed occupational safety concerns by promoting universal precaution as a way of self-protection at work. This strategy built upon our previous studies that discovered a lack of universal precaution knowledge and supplies among providers, and its relationship with the providers’ avoidance attitudes to serve people living with HIV [27, 28]. This approach was well accepted by the participating providers, and we received feedback from participants that the intervention message was very relevant to their self-interests. By adhering to universal precautions in their medical practice, the service providers released fear of occupational exposure and became more willing to serve HIV-positive patients.

During the implementation of an intervention, it is important to recognize its community context and use culturally appropriate intervention strategies [29–31]. For this project, we focused on preserving the fidelity of the intervention component while also incorporating local elements. The involvement of experienced local educators and use of local language enhanced the acceptability and sustainability of the intervention. In addition, we identified HIV specialists and local representatives of people living with HIV and presented their personal stories during the intervention sessions. Such real-life stories reminded service providers of the existence of stigma and inspired them to follow the community role model to make changes in their professional environment.

Governmental support in making changes at the structural level was crucial to the stigma reduction project [31]. From a previous study, we discovered that stigma among service providers was largely influenced by structural barriers such as the availability of universal precaution [13]. The intervention project successfully engaged NCAIDS, CCDC, the leading HIV/STD control agency in China, to allocate about 100,000 yuan (approximately U.S. $15,000) in subventions for universal precaution supplies to the participating hospitals. Although this funding was insufficient to meet the demand for universal precaution supplies in all hospitals, the action was regarded as a clear gesture of the involvement of health authorities in supporting occupational safety, which further initiated safer medical practice conversations among local hospital administrators. Following the action of NCAIDS, CCDC, hospitals in the intervention condition made further purchases of universal precaution supplies. The structural change was thus sustained beyond the project period and was translated into the service providers’ routine medical practice.

## Conclusions

This article describes the implementation process of an intervention program that has the potential to reduce HIV-related stigma in medical settings. Since the intervention focuses on equal treatment for all patients, it can easily be applied to stigma reduction programs in a number of different populations. During the adaptation, however, one should consider the participants’ needs and recognize culture and community contexts. Policy support in structural change is warranted to incorporate the intervention into existing healthcare settings to ensure sustained outcomes.

## References

[1] Jürgensen M, Sandøy IF, Michelo C, Fylkesnes K, ZAMACT Study Group (2013). Effects of home-based voluntary counselling and testing on HIV-related stigma: findings from a cluster-randomized trial in Zambia. Soc Sci Med.

[2] Mahajan AP, Sayles JN, Patel VA, Remien RH, Sawires SR, Ortiz DJ (2008). Stigma in the HIV/AIDS epidemic: a review of the literature and recommendations for the way forward. AIDS.

[3] Nyblade L, Pande R, Mathur S, MacQuarrie K, Kidd R, Banteyerga H (2003). Disentangling HIV and AIDS stigma in Ethiopia, Tanzania and Zambia.

[4] Ogden J, Nyblade L (2005). Common at its core: HIV-related stigma across contexts.

[5] Rahmati-Najarkolaei F, Niknami S, Aminshokravi F, Bazargan M, Ahmadi F, Hadjizadeh E (2010). Experiences of stigma in healthcare settings among adults living with HIV in the Islamic Republic of Iran. J Int AIDS Soc.

[6] Reidpath DD, Chan KY (2005). HIV discrimination: integrating the results from a six-country situational analysis in the Asia Pacific. AIDS Care.

[7] Thanh DC, Moland KM, Fylkesnes K (2012). Persisting stigma reduces the utilisation of HIV-related care and support services in Viet Nam. BMC Health Serv Res.

[8] Bogart LM, Chetty S, Giddy J, Sypek A, Sticklor L, Walensky RP (2013). Barriers to care among people living with HIV in South Africa: contrasts between patient and healthcare provider perspectives. AIDS Care.

[9] Ma W, Detels R, Feng Y, Wu Z, Shen L, Li Y (2007). Acceptance of and barriers to voluntary HIV counselling and testing among adults in Guizhou province, China. AIDS.

[10] Ti L, Hayashi K, Kaplan K, Suwannawong P, Wood E, Montaner J (2013). HIV test avoidance among people who inject drugs in Thailand. AIDS Behav.

[11] Rutledge SE, Abell N, Padmore J, McCann TJ (2009). AIDS stigma in health services in the Eastern Caribbean. Sociol Health Illn.

[12] UNAIDS (2005). HIV – related stigma, discrimination and human rights violations [Internet]. http://data.unaids.org/publications/irc-pub06/jc999-humrightsviol_en.pdf.

[13] Li L, Liang LJ, Wu ZY, Lin C, Wu S (2008). Institutional support for HIV/AIDS care in China: a multilevel analysis. AIDS Care.

[14] Wu S, Li L, Wu Z, Liang LJ, Cao H, Yan Z (2008). A brief HIV stigma reduction intervention for service providers in China. AIDS Patient Care STDS.

[15] Uys L, Chirwa M, Kohi T, Greeff M, Naidoo J, Makoae L (2009). Evaluation of a health setting-based stigma intervention in five African countries. AIDS Patient Care STDS.

[16] Yiu JW, Mak WW, Ho WS, Chui YY (2010). Effectiveness of a knowledge-contact program in improving nursing students’ attitudes and emotional competence in serving people living with HIV/AIDS. Soc Sci Med.

[17] Meissner HI, Glasgow RE, Vinson CA, Chambers D, Brownson RC, Green LW (2013). The U.S. training institute for dissemination and implementation research in health. Implement Sci.

[18] Li L, Cao H, Wu Z, Wu S, Xiao L (2007). Diffusion of positive AIDS care messages among service providers in China. AIDS Educ Prev.

[19] Li L, Wu Z, Liang LJ, Lin C, Guan J, Jia M (2013). Reducing HIV-related stigma in health care settings: a randomized controlled trial in China. Am J Public Health.

[20] Rogers EM (1983). Diffusion of Innovations.

[21] Kelly J (2004). Popular opinion leaders and HIV peer education: resolving discrepant findings, and implications for the implementation of effective community programmes. AIDS Care.

[22] Berner ES, Baker CS, Funkhouser E, Heudebert GR, Allison JJ, Fargason CA (2003). Do local opinion leaders augment hospital quality improvement efforts? A randomized trial to promote adherence to unstable angina guidelines. Med Care.

[23] Soumerai SB, McLaughlin TJ, Gurwitz JH, Guadagnoli E, Hauptman PJ, Borbas C (1998). Effect of local medical opinion leaders on quality of care for acute myocardial infarction: a randomized controlled trial. JAMA.

[24] Jia M, Luo H, Ma Y, Wang N, Smith K, Mei J (2010). The HIV epidemic in Yunnan Province, China, 1989–2007. J Acquir Immune Defic Syndr.

[25] Yao X, Wang H, Yan P, Lu Y, Lin H, Chen L (2012). Rising epidemic of HIV-1 infections among general populations in Fujian, China. J Acquir Immune Defic Syndr.

[26] Kelly JA, St Lawrence JS, Diaz YE, Stevenson LY, Hauth AC, Brasfield TL (1991). HIV risk behavior reduction following intervention with key opinion leaders of population: an experimental analysis. Am J Public Health.

[27] Li L, Lin C, Wu Z, Guan J, Jia M, Yan Z (2011). HIV-related avoidance and universal precaution in medical settings: opportunities to intervene. Health Serv Res.

[28] Wu S, Li L, Wu Z, Cao H, Lin C, Yan Z (2008). Universal precautions in the era of HIV/AIDS: perception of health service providers in Yunnan, China. AIDS Behav.

[29] Bernal G, Jiménez-Chafey MI, Domenech Rodríguez MM (2009). Cultural adaptation of treatments: a resource for considering culture in evidence-based practice. Prof Psychol Res Pr.

[30] Gearing RE, Schwalbe CS, Mackenzie MJ, Brewer KB, Ibrahim RW, Olimat HS (2012). Adaptation and translation of mental health interventions in Middle Eastern Arab countries: a systematic review of barriers to and strategies for effective treatment implementation. Int J Soc Psychiatry.

[31] Nyblade L, Stangl A, Weiss E, Ashburn K (2009). Combating HIV stigma in health care settings: what works?. J Int AIDS Soc.

# Sub article: Testing the efficacy of an HIV stigma reduction intervention with medical students in Puerto Rico: the SPACES project

**DOI:** 10.7448/IAS.16.3.18670

**Published:** 2013-11-13

**Authors:** Nelson Varas-Díaz, Torsten B Neilands, Francheska Cintrón-Bou, Melissa Marzán-Rodríguez, Axel Santos-Figueroa, Salvador Santiago-Negrón, Domingo Marques, Sheilla Rodríguez-Madera

**Affiliations:** 1 Center for Social Research, University of Puerto Rico, Río Piedras Campus, San Juan, Puerto Rico; 2 Center for AIDS Prevention Studies (CAPS), University of California, San Francisco, CA; 3 Clinical Psychology Program, Ponce School of Medicine and Health Sciences, Ponce, Puerto Rico; 4 Social Sciences Department, Graduate School of Public Health, University of Puerto Rico, Medical Sciences Campus, San Juan, Puerto Rico

**Keywords:** HIV, stigma, intervention, reduction, Puerto Rico

## Abstract

**Introduction:**

Stigma associated with HIV has been documented as a barrier for accessing quality health-related services. When the stigma manifests in the healthcare setting, people living with HIV receive substandard services or even be denied care altogether. Although the consequences of HIV stigma have been documented extensively, efforts to reduce these negative attitudes have been scarce. Interventions to reduce HIV stigma should be implemented as part of the formal training of future healthcare professionals. The interventions that have been tested with healthcare professionals and published have several limitations that must be surpassed (i.e., lack of comparison groups in research designs and longitudinal follow-up data). Furthermore, Latino healthcare professionals have been absent from these intervention efforts even though the epidemic has affected this population disproportionately.

**Methods:**

In this article, we describe an intervention developed to reduce HIV stigma among medical students in Puerto Rico. A total of 507 medical students were randomly introduced into our intervention and control conditions.

**Results:**

The results show statistically significant differences between the intervention and control groups; intervention group participants had lower HIV stigma levels than control participants after the intervention. In addition, differences in HIV stigma levels between the groups were sustained for a 12-month period.

**Conclusions:**

The results of our study demonstrate the efficacy of the modes of intervention developed by us and serve as a new training tool for future healthcare professionals with regard to stigma reduction.

## Introduction

### The burden of HIV in the Caribbean and Puerto Rico

The Caribbean is the second most HIV-affected region in the world with an estimated prevalence of 1% [1]. The island of Puerto Rico, a non-incorporated territory of the United States with a population of 3.7 million, has been heavily affected by the epidemic with more than 40,000 reported infections [2, 3]. Puerto Rico holds the fifth position in AIDS diagnosis rates (26.4/100,000) in the United States and the fourth position in its prevalence among those older than 13 years (335.1/100,000) [4]. A mainly male-driven epidemic (74%), the most common modes of transmission of HIV are needle sharing for illegal drug use (45%), unprotected heterosexual contact (27%), and unprotected relations between men who have sex with men (17%). Recent research has documented that an estimated 1% of the Puerto Rican population is living with HIV [5].

### HIV stigma and its consequences

HIV stigma remains one of the most challenging barriers to maintaining the overall health of people living with HIV (PLHIV). The stigma affects mental health by fostering depression, low self-esteem and anxiety [6, 7]. It also influences physical health by hindering adherence to antiretroviral treatment, accelerating disease progression [8, 9]. Finally, HIV stigma has been shown to hinder social interaction because PLHIV can feel ostracized, which leads to significant reduction in or complete elimination of their social networks [10].

The consequences of HIV stigma worsen when stigmatizing behaviour originates from people who are important in the lives of PLHIV, such as healthcare professionals [7]. This is particularly true for physicians, who play such a pivotal role in treatment. These professionals represent the first line of contact for treatment and the basis of knowledge of effective strategies to restrain disease progression. They can also be an important source of support for PLHIV [11]. When physicians stigmatize PLHIV, access to effective treatment can be limited. Previous studies carried out in Puerto Rico have documented how healthcare professionals, particularly physicians, manifest HIV stigma [12]. Because physicians play an essential role in the lives of PLHIV in Puerto Rico, scientifically tested strategies to reduce stigma among them are urgently needed.

A review of the published literature on HIV stigma reduction efforts reveals important gaps that must be addressed, including lack of efficacious interventions to reduce stigma among healthcare professionals from Latino backgrounds [13, 14].

### HIV stigma reduction interventions with health professionals

Reducing HIV stigma in healthcare scenarios has been a public health concern in multiple countries [10, 11, 15–20]. These efforts have pointed towards the need for developing interventions that place emphasis on the individuals, facility environments and policies [16, 21].

A review of published scientific studies on HIV demonstrates that existing interventions to reduce HIV stigma are scarce, and those that focus specifically on physicians are few [22]. This gap is worsened by the unsystematic use of measures to evaluate HIV stigma reduction and problems with internal validity of research designs. These challenges were initially posed in a meta-analysis published by Brown and colleagues [13], which yielded only 22 published articles documenting scientifically tested interventions. Only 5 of the 22 interventions were developed specifically for healthcare professionals. Some of the limitations identified are: (1) using small samples, (2) conducting interventions that did not reduce fear of PLHIV, (3) not measuring HIV stigma with reliable and valid scales, and (4) seeing little evidence of sustained intervention impact beyond three months.

A recent intervention carried out in China has addressed structural changes by making universal precaution supplies accessible in hospitals and documented significant differences in attitudinal and behavioural changes of its participants [23–25]. Although such efforts are encouraging and scientifically sound, similar strategies have not been implemented with Latino health professionals. Considering that HIV has impacted the Latino community disproportionately, including Puerto Ricans, the need to develop interventions to reduce HIV stigma among physicians caring for Latinos is urgent [12].

### The SPACES intervention

The developed intervention is based on extensive qualitative work with healthcare professionals and medical students in Puerto Rico [12, 26–28]. We named our intervention SPACES in line with our promotional tagline of fostering “stigma-free spaces in medical scenarios.” The intervention is a nine-hour workshop divided into three sessions (three hours each). SPACES addresses the sources and functions of HIV stigma based on Goffman's theoretical contributions [29], issues that can worsen its consequences based on Jones’ stigma dimensions [30] and focuses on both instrumental and symbolic stigmas manifested for HIV [31, 32]. A description of the content for each session can be found in Table 1.

**Table 1:** Overview of the SPACES intervention

Session	Content and educational technique
1	Content: Information on HIV stigma and its consequences on service delivery. Educational technique: In this session, we addressed participants’ knowledge of HIV epidemiological data in Puerto Rico. Small groups were asked to outline the social groups that were most impacted by the epidemic. We later contrasted their perceptions of impacted groups with actual epidemiological data. This process allowed us to discuss how HIV stigma can influence medical students’ perceptions of the epidemic and the role stigma plays in this process. Furthermore, we discuss how social stigma related to HIV is also intertwined with other pre-existing stigmas related to illegal drug use, homosexuality and gender roles.
2	Content: The role of negative emotions in HIV stigma. Educational technique: In this second session, we addressed the role of negative emotions in fostering HIV stigma attitudes and behaviours when interacting with PLHIV. Participants were exposed to clinical vignettes of HIV infection cases and asked to complete charts detailing the types of emotions they experienced when discussing the cases (e.g., fear, shame, disgust, admiration). Small groups discussions on these cases were complemented with whole group sessions in which the role of emotions in stigma was explained. Culturally accepted emotions such as “pity” were discussed as potential sources of stigma.
3	Content: Skills for stigma-free interaction with PLHIV. Educational technique: In this last session, we discussed specific behavioural skills for interacting with PLHIV in clinical scenarios in a non-stigmatizing manner. Furthermore, we discussed how HIV stigma that is manifested in society could also be manifested in clinical encounters by both the physician (i.e., denying services, providing sub-standard services) and PLHIV (i.e., low self-esteem, self-stigmatizing attitudes). Examples of stigma manifestations through media outlets and policies were discussed in order to provide an overview of a social scenario in which clients might feel stigmatized. We stressed the importance of providing stigma-free spaces and interactions in medical settings.

The SPACES workshops were offered to students as extracurricular activities to ensure that their ongoing classwork and clinical practice were not affected. To facilitate attendance and participation, the workshops were provided within the students’ medical schools. Participants were provided a stipend each time they completed a questionnaire for a total sum amount of $125 ($25 at T1–T3 and $50 at T4). The workshops were facilitated by six health professionals with advanced degrees (MA and PhDs) and previous experience with HIV-related patients. As part of our process evaluation, participants mentioned that the intervention allowed them to correct information about HIV. One participant mentioned: “It was very important because some of the information I had was totally wrong.” They also described the intervention as a positive experience, and another participant reported the following: “I feel more open minded to working with patients.” Our study aimed at assessing the efficacy of the SPACES intervention in reducing HIV stigma attitudes among medical students in Puerto Rico.

## Methods

To achieve the aim of our study, we implemented a randomized controlled trial with group randomization to the SPACES intervention and a non-stigma control group. Details are presented below.

### Participants

Our sample consisted of 507 second year medical students. The sample characteristics are described below in the results section.

### Procedure

Participants were recruited from the four largest medical schools in Puerto Rico. Our team visited medical schools throughout the Island to meet with students and invite them to participate. Their participation was voluntary and we ensured them that it would not influence their evaluation by other professors in their courses. Groups of 20 were randomized into our intervention and control conditions. We implemented a basic HIV epidemiology workshop as a time- and attention-matched control group experience. Participants completed our baseline measure (T1) before engaging in the workshops and immediately after (T2) completing the third and last sessions. They were contacted by phone and via email in order to complete the 6- (T3) and 12-month follow-up (T4) over the web. Attendance to our intervention workshops was high with 86% of participants completing all three sessions, 10% completing two sessions and 4% completing one session. Drop-out rates at T2 and T3 measurements were low, with 92% of the participants completing T2 measures and 85% completing T3. A total of 385 out of the 507 participants completed the T4 measure with a 24% attrition rate. We implemented our intervention from January, 2008, through April, 2011.

### Measures

Participants completed a self-administered questionnaire containing several scales, including HIV knowledge, perceptions of self-efficacy for providing services, social desirability and HIV stigma, the primary outcome variable. The Spanish HIV Stigma Scale (SHASS) is a reliable and culturally appropriate scale previously developed in Puerto Rico, which measures 11 dimensions of HIV stigma: 1) restriction of PLHIV's rights, 2) PLHIV obliged to reveal HIV status, 3) responsibility of PLHIV for their HIV infection, 4) lack of productivity of PLHIV, 5) personal characteristics of PLHIV, 6) fear of infection, 7) emotions associated with HIV, 8) closeness to death, 9) need to control PLHIV, 10) PLHIV as vectors of infection and 11) body signs of HIV. All items are measured by a 5-point Likert-type scale ranging from strongly agree (5) to strongly disagree (1) [33].

### Data analysis

*Descriptive Analyses –* Initial descriptive analyses characterized the sample using one-way and cross-tabular frequency tables with counts and percentages displayed by control versus intervention group. Likelihood ratio chi-square tests were used to compare percentages across groups on unordered categorical variables. Mantel-Haenszel chi-square tests were used to test for control versus intervention group differences on the following ordinal variables: importance of religion, income, risk for HIV infection and perceptions of medical students’ attitudes towards PLHIV. Exact chi-square test statistics were substituted for the default asymptotic chi-square tests, if the expected cell counts were less than five. Means and standard deviations were generated for the HIV-stigma measure for each group at each measurement wave.

*Comparisons of Means –* The primary analysis was a comparison of means performed using a 2 (Group: Intervention vs. Control) by 4 (Wave: 1, 2, 3 or 4) repeated measures analysis. Group and Wave were treated as fixed effects. The covariance structure among the repeated measurements was set to unstructured as recommended by Diggle et al. (2002) [34] for designs with few fixed measurement points. Model-based means were estimated and compared using restricted maximum likelihood (REML) in SAS PROC MIXED with the Kenward-Roger method used to compute the denominator degrees of freedom [35]. The Group-by-Wave interaction omnibus test and its constituent components were used to determine whether the differences between the intervention and control groups differed over time. These tests were followed up with paired comparisons of the intervention and control group means within each of the four waves of measurement; these paired comparisons’ *p*-values were adjusted using Sidak's method to control the Type 1 error rate [36]. Cohen's standardized effect size *d* was computed for the post-intervention mean differences as an index of the magnitude of the intervention effect.

## Results

*Sample Characteristics –* The sample was approximately gender balanced, heterosexual and Puerto Rican (Table 2). Nearly half the sample (46%) had tested for HIV and none reported being HIV-positive. However, more than one-fourth of the sample knew someone with HIV and the vast majority reported that HIV was discussed in their medical school coursework, yet more than 90% of the participants believed other medical students discriminated against PLHIV. Approximately two-thirds indicated religion as being important or very important, and 80% either disagreed or felt unsure that they were prepared to provide services to PLHIV (Table 2).

**Table 2:** Sample characteristics

	Intervention		Control			
				
Variable	*N*	%	*N*	%	χ^2^ (DF)	*p*
Male gender	123	45.4	109	46.2	0.03 (1)	0.86
Heterosexual orientation	264	97.8	233	98.7	0.67 (1)	0.51
National origin					7.69 (3)	0.07
Puerto Rican	213	78.9	204	86.4		
Dominican	1	0.4	3	1.3		
Cuban	10	3.7	5	2.1		
Other	46	17.0	24	10.2		
Ever tested for HIV	125	46.3	108	46.4	0.0002 (1)	0.99
HIV negative test result (among those who tested)	123	96.1	99	94.3	0.42 (1)	0.55
Knew someone with HIV	79	29.7	61	26.9	0.48 (1)	0.49
Taken a class where HIV was discussed	239	88.2	196	83.4	2.39 (1)	0.12
Believe other medical students discriminate	245	90.7	216	92.3	0.40 (1)	0.53
Religion importance						
Not important	29	10.7	18	7.6	0.67 (1)	0.41
Somewhat important	69	25.6	57	24.2		
Important	89	33.0	90	38.1		
Very important	83	30.7	71	30.1		
Annual income						
<$10,000	81	31.6	89	39.6	0.08 (1)	0.77
$10,001–$20,000	30	11.7	17	7.6		
$20,001–$30,000	26	10.2	16	7.1		
$30,001–$40,000	37	14.5	23	10.2		
$40,001–$50,000	30	11.7	15	6.7		
$50,001–$60,000	8	3.1	12	5.3		
>$60,000	44	17.2	53	23.6		
Perception of risk of HIV infection						
Not at all	73	27.2	56	23.9	2.96 (1)	0.09
A little	150	56.0	116	49.6		
A regular amount	31	11.6	52	22.2		
A lot	14	5.2	10	4.3		
Medical students attitudes towards PLHIV						
Totally positive	12	4.4	12	5.1	0.26 (1)	0.61
Partially positive	72	26.6	56	23.8		
Neutral	123	45.4	106	45.1		
Partially negative	64	23.6	61	26.0		
Prepared to provided services to PLHIV						
Totally agree	13	4.8	7	3.0	1.40 (1)	0.24
Partially agree	42	15.5	39	16.5		
Undecided	178	65.7	146	61.9		
Partially disagree	38	14.0	44	18.6		

Notes: Percentages and *N*s will not always sum to 100% due to small amounts of missing data. For sexual orientation, the comparison category is homosexual/lesbian/bisexual. For HIV testing, the comparison group was “Don't know” (no respondents reported an HIV-positive test result).

*Comparisons of Means –* Statistically significant main effects for intervention group (*F*(1, 499)=14.97, *p=*0.0001) and wave of measurement (*F*(3, 439)=22.23, *p*<0.0001) were found. These main effects were qualified by a statistically significant Group-by-Wave interaction (*F*(3, 439)=8.82, *p<*0.0001). The three individual components of the interaction effect were then examined to determine whether the mean differences between the groups at baseline were statistically different from the same group differences at each follow-up point, with the difference between the two differences quantified as *D*. The comparison of the baseline group difference with the immediate follow-up group difference was significant (*D*=0.16, *t*(472.7)=4.91, *p*<0.0001). The comparison of the baseline group difference with the six-month group difference was also significant, though the effect was weaker (*D*=0.08, *t*(460.8)=2.18, *p*=0.03). Finally, the comparison of the baseline group difference with the 12-month group difference was also significant (*D*=0.12, *t*(431.5)=2.90, *p*=0.004). Follow-up paired comparisons of the group's means within each time point revealed no significant difference between the groups at baseline, but the mean levels of HIV stigma were significantly lower in the intervention group at each of the three follow-up measurement waves (see Table 3). The Cohen's *d* value for the comparison of intervention and control group means immediately following the intervention was −0.40. At the six month follow-up, the corresponding *d* value was −0.29, and at the 12 month follow-up, the *d* value was −0.30. Benchmark values for *d* are *d*=0.20 for a small effect and *d*=0.50 for a medium effect. Therefore, the intervention exhibited an effect between small and medium in reducing HIV stigma (see Table 4).

**Table 3:** Means and differences for HIV stigma scores

	Sample means (SD)								
				
	Control			Intervention					
					
Wave	*N*	*M*	*SD*	*N*	*M*	*SD*	*t*	*df*	*p*
1	234	2.88	(0.48)	269	2.79	(0.51)	−1.82	501.5	0.25
2	219	2.83	(0.51)	241	2.61	(0.58)	−4.83	494	<0.0001
3	197	2.80	(0.54)	225	2.64	(0.55)	−3.22	487	0.0055
4	179	2.77	(0.57)	206	2.59	(0.59)	−3.74	473	0.0002

Notes: M=Mean; SD=Standard Deviation. *t*, *df* and *p*-values are estimated from SAS PROC MIXED with the Kenward-Roger degrees-of-freedom method, which features non-integer DF values. *p*-Values for paired comparisons of group means were adjusted for multiple comparisons using Sidak's method.

## Discussion

The results of our study suggest that the SPACES intervention is an efficacious tool for stigma reduction among medical students. Results evidenced significantly lower stigma levels immediately following intervention among persons who completed the intervention, irrespective of starting levels of stigma. Furthermore, significantly lower stigma levels were documented at 6 and 12 months. Therefore, the SPACES intervention is the first stigma reduction effort to be systematically tested in Puerto Rico via a randomized controlled trial with demonstrated stigma reduction.

**Table 4:** Model-implied means and differences from latent growth model analysis

	At mean baseline stigma					
		
Wave	Control	Intervention	*D*	*Z*	*p*	*d*

1	2.83	2.83	0	–	–	0
2	2.80	2.63	−0.17	−6.69	<.001	−0.30
3	2.79	2.68	−0.10	−3.33	<.001	−0.15
4	2.75	2.61	−0.14	−3.30	.001	−0.15
	At 1 SD below baseline stigma					
		
Wave	Control	Intervention	*D*	*Z*	*p*	*d*

1	2.39	2.39	0	–	–	
2	2.36	2.10	−0.26	−6.63	<0.001	−0.30
3	2.34	2.17	−0.17	−3.85	<0.001	−0.17
4	2.31	2.08	−0.23	−4.40	<0.001	−0.20
	At 1 SD above baseline stigma					
		
Wave	Control	Intervention	*D*	*Z*	*p*	*d*

1	3.27	3.27	0	–	–	
2	3.25	3.17	−0.08	−2.00	0.045	−0.09
3	3.22	3.18	−0.04	−1.07	0.28	−0.05
4	3.20	3.15	−0.05	−0.98	0.33	−0.04

Notes: *N*=506. *D*=Difference defined as control group mean minus intervention group mean. *d*=approximate standardized *D* defined as *D*/(SE*sqrt(*N*)), where *SE* is the standard error of the estimate and *N* is the sample size. Model-implied means, differences and associated test statistics were estimated using full-information maximum likelihood (FIML) with residual bootstrap-based standard errors in M*plus* 7.

This initial evaluation of the SPACES intervention provides the medical training community with a promising tool for HIV stigma reduction. We understand that several strengths in the process of development and implementation of the program will allow medical schools to easily uptake the SPACES intervention. For example, the SPACES intervention can be incorporated into existing medical coursework on issues of cultural competence and ethical treatment of patients due to its closely interrelated content. Also, the workshops are similar in length and structure to medical classroom activities and therefore could easily be incorporated into existing classwork. Finally, the intervention can strengthen the portfolios of medical schools on evidence-based training of medical students, which is an important aspect of curriculum accreditation. For these reasons, we believe that medical schools are in an advantageous position to incorporate the SPACES intervention into their academic sequences and institutional training policies.

The SPACES intervention also has the potential to address stigma reduction at institutional and government policy levels. For example, institutional policies within medical schools could focus on the need for stigma reduction as part of medical training and integrate SPACES as a stigma reduction tool. This potential policy level decision within medical training scenarios could help to ensure that participation in stigma reduction efforts is not simply voluntary but an integral aspect of medical training in which all students and faculty members should engage. Furthermore, the SPACES intervention is an important initial step in the development of stigma reduction efforts for already practicing physicians, which could benefit from this effort with minor modifications to our existing intervention. This initial effort has the potential to introduce stigma reduction as a vital subject of medical school training and, in the future, accreditations for practicing physicians.

Although these results are promising, and provide HIV stigma reduction practitioners with a new tool for action, several steps need to be taken in the future in order to continue innovating in the field of stigma reduction. Some examples include 1) exploring by which mechanisms the intervention changes attitudes (i.e., mediators) and for whom the intervention is most efficacious (i.e., moderators), 2) tailoring the SPACES intervention to reduce stigma combinations (i.e., homophobia, stigma towards illegal drug users), 3) documenting the sustained effects of the intervention through longer periods of time (i.e., 24 and 36 months) when medical students are exposed to experiences that can foster negative attitudes towards PLHIV and 4) exploring the consequences of stigma attitude reduction on its behavioural manifestations (e.g., denying care, providing substandard care) when interacting with PLHIV.

We understand that some of these recommended steps will guide the development of future stigma reduction interventions in the coming years due to their importance for HIV prevention and treatment. It is of vital importance to continue generating efforts that produce data that demonstrate stigma reduction via a plurality of mechanisms. For example, although the reduction of stigma attitudes has been utilized as an indicator of interventions’ efficacy, we still know very little about the implications of stigma attitude reduction on health professionals’ specific behaviours in the clinical encounter. The behavioural implication of stigma attitude reduction needs to be better explored though interventions that use both attitudinal and observational measurements to assess their joint impact on stigma reduction. In this same line, future stigma reduction interventions need to report their standardized effect sizes, which are seldom included in scientific papers, in order for the field to develop a base rate to which new interventions can be compared.

The stigmatization of HIV continues to be a problem for PLHIV at a global level. Stigma reduction interventions need to be tested and disseminated in order to have them widely available and potentially impact the lives of PLHIV. This entails adopting a global perspective when scaling up stigma reduction interventions. It is our hope that our work in Puerto Rico will serve as a model for future stigma reduction interventions with medical students and that in unison with other tested efforts, we can collectively impact the training of stigma-free physicians.

## References

[1] UNAIDS (2012). Global fact sheet. http://www.unaids.org/en/media/unaids/contentassets/documents/epidemiology/2012/gr2012/20121120_FactSheet_Global_en.pdf.

[2] U.S. Census Bureau (2010). Características Sociales Seleccionadas en Puerto Rico. http://factfinder2.census.gov/faces/nav/jsf/pages/community_facts.xhtml.

[3] Puerto Rico AIDS Surveillance (2013) (2013). Cumulative HIV/AIDS cases diagnosed as of February.

[4] CDC (2010). Epidemiology of HIV infection. http://www.cdc.gov/hiv/topics/surveillance/resources/slides/general/slides/general.ppt#.

[5] Perez CM, Marrero E, Meléndez M, Adrovet S, Colón H, Ortiz AP (2010). Seroepidemiology of viral hepatitis, HIV and herpes simplex type 2 in the household population aged 21–64 years in Puerto Rico. BMC Infect Dis.

[6] Kuang W, Li J, Ma Y, Liao J (2005). Survey on psychologic status and quality of life for HIV infected people or AIDS patients. J Sichuan University.

[7] Li L, Lin C, Wu Z, Rotheram-Borus MJ, Detels R, Jia M (2007). Stigmatization and shame: consequences of caring for HIV/AIDS patients in China. AIDS Care.

[8] Malavé S, Varas-Díaz N (2006). Regulando la enfermedad a través de la definición y la restricción: Profesionales de la salud hablan sobre el VIH/SIDA. Ciencias de la Conducta.

[9] Sayles JN, Wong MD, Kinsler JJ, Martins D, Cunnigham WE (2009). The association of stigma with self reported access to medical care and antiretroviral therapy adherence in persons living with HIV/AIDS. J Gen Intern Med.

[10] Rahmati-Najarkolaei F, Shamsaddin N, Aminshokravi F, Bazargan M, Ahmadi F, Hadjizadeh E (2010). AIDS society: experience stigma in healthcare settings among adults living with HIV in the Islamic Republic of Iran. J Int AIDS Soc.

[11] Hossain MB, Kippax S (2011). Stigmatized attitudes toward people living with HIV in Bangladesh: health care workers’ perspectives. Asia Pac J Publ Health.

[12] Varas-Díaz N, Neilands TB, Malavé-Rivera S, Betancourt E (2010). Religion and HIV stigma: implications for health professionals in Puerto Rico. Global Publ Health.

[13] Brown L, Mcintyre K, Trujillo L (2003). Interventions to reduce HIV/AIDS stigma: what have we learned?. AIDS Educ Prev.

[14] Ho Y, Li X (2009). HIV/AIDS behavioral interventions in China: a literature review and recommendation for future research. AIDS Behav.

[15] Nyblade L, Stangl A, Weiss E, Ashburn K (2009). Combating HIV stigma in healthcare settings: what works?. J Int AIDS Soc.

[16] Mahendra VS, Gilborn L, George B, Samson L, Mudoi R, Jadav S (2006). Reducing AIDS-related stigma and discrimination in Indian hospitals.

[17] Apinundecha C, Laohasiriwong W, Cameron MP, Lim S (2007). A community participation intervention to reduce HIV/AIDS stigma, Nakhon Ratchasima province, northeast Thailand. AIDS Care.

[18] Uys L, Chirwa M, Kohi T, Greeff M, Naidoo J, Makoae L (2009). Evaluation of a health setting-based stigma intervention in five African countries. AIDS Patient Care STDS.

[19] Rutledge SE, Abell N, Padmore J, McCann TJ (2009). AIDS stigma in health services in the Eastern Caribbean. Sociol Health Illn.

[20] Zhang KL, Detels R, Liao S, Cohen M, Yu DB (2008). China's HIV/AIDS epidemic: continuing challenges. Lancet.

[21] Oanh KTH, Ashburn K, Pulerwitz J, Ogden J, Nyblade L (2008). Improving hospital-based quality of care in Vietnam by reducing HIV-related stigma and discrimination, a Horizons final report.

[22] Sengupta S, Banks B, Jonas D, Miles M, Smith S (2011). HIV interventions to reduce HIV.AIDS stigma: a systematic review. AIDS Behav.

[23] Neema S, Atuyambe LM, Otolok-Tanga E, Twijukye C, Kambugu A, Thayer L (2012). Using a clinic based creativity initiative to reduce HIV related stigma at the Infectious Diseases Institute, Mulago National Referral Hospital, Uganda. Afr Health Sci.

[24] Li L, Zunyou W, Li-Jung L, Chunqing L, Jihui G, Manhong J (2013). Reducing HIV-related stigma in healthcare settings: a randomized controlled trial in China. Am J Publ Health.

[25] Li L, Li-Jung L, Chunqing L, Zunyou W, Rotheram-Borus M (2010). HIV prevention intervention to reduce HIH-related stigma: evidence from China. AIDS.

[26] Varas-Díaz N, Neilands TB, Cintrón-Bou F, Santos-Figueroa A, Marzán-Rodríguez M, Marqués D (2013). Religion and HIV stigma in Puerto Rico: a cultural challenge for training future physicians. J Int Assoc Phys AIDS Care.

[27] Varas-Díaz N, Neilands TB, Cintrón-Bou F, Santos-Figueroa A, Rodríguez-Madera S, Santiago-Negrón S (2012). The role of gender on HIV stigma among medical students in Puerto Rico: implications for training and service delivery. Puerto Rico Health Sci J.

[28] Varas-Díaz N, Santiago-Negrón S, Torsten TB, Cintrón-Bou F, Malavé-Rivera S (2010). Stigmatization of illicit drug use among Puerto Rican health professionals in training. Puerto Rico Health Sci J.

[29] Goffman E (1963). Stigma: notes on the management of spoiled identity.

[30] Jones EE, Farina A, Hastorf A, Markus H, Miller DT, Scott RA (1984). Social stigma: the psychology of marked relationships.

[31] Herek GM, Glunt EK (1988). An epidemic of stigma: public reactions to AIDS. Am Psychol.

[32] Herek GM (1999). AIDS and stigma. Am Behav Sci.

[33] Varas-Díaz N, Torsten TB (2009). Development and validation of a culturally appropriate HIV stigma scale for Puerto Rican health professionals in training. AIDS Care.

[34] Diggle PJ, Heagarty P, Liang K-Y, Zeger SL (2002). Analysis of longitudinal data.

[35] Littell RC, Milliken GA, Stroup WW, Wolfinger RD, Schabenberger O (2006). SAS system for mixed models.

[36] Šidák Z (1967). Rectangular confidence regions for the means of multivariate normal distributions. J Am Stat Assoc.

# Sub article: A brief, standardized tool for measuring HIV-related stigma among health facility staff: results of field testing in China, Dominica, Egypt, Kenya, Puerto Rico and St. Christopher & Nevis

**DOI:** 10.7448/IAS.16.3.18718

**Published:** 2013-11-13

**Authors:** Laura Nyblade, Aparna Jain, Manal Benkirane, Li Li, Anna-Leena Lohiniva, Roger McLean, Janet M Turan, Nelson Varas-Díaz, Francheska Cintrón-Bou, Jihui Guan, Zachary Kwena, Wendell Thomas

**Affiliations:** 1 Health Policy Project and RTI International, Washington, DC, USA; 2 Health Policy Project, Futures Group,Washington, DC, USA; 3 Global Disease Detection and Response Programme at the U.S. Naval Medical Research Unit No. 3, Cairo, Egypt; 4 Department of Psychiatry and Biobehavioural Sciences, School of Public Health, Semel Institute – Center for Community Health, University of California, Los Angeles, CA, USA; 5 Department of Epidemiology, School of Public Health, Semel Institute – Center for Community Health, University of California, Los Angeles, CA, USA; 6 Faculty of Social Sciences, Centre for Health Economics, University of the West Indies, Port-of-Spain, Trinidad and Tobago; 7 Department of Health Organization and Policy, School of Public Health, University of Alabama at Birmingham, Birmingham, AL, USA; 8 Center for Social Research, Social Sciences Faculty, University of Puerto Rico, San Juan, PR, USA; 9 Fujian Provincial Centre for Disease Control and Prevention, Fuzhou, China; 10 Centre for Microbiology Research (CMR), Kenya Medical Research Institute (KEMRI), Kisumu, Kenya; 11 Caribbean Data Management Systems, Port-of-Spain, Trinidad and Tobago

**Keywords:** stigma, discrimination, measurement, stigma-reduction programmes, monitoring, evaluation, health facilities, HIV, AIDS, HIV stigma

## Abstract

**Introduction:**

Within healthcare settings, HIV-related stigma is a recognized barrier to access of HIV prevention and treatment services and yet, few efforts have been made to scale-up stigma reduction programs in service delivery. This is in part due to the lack of a brief, simple, standardized tool for measuring stigma among all levels of health facility staff that works across diverse HIV prevalence, language and healthcare settings. In response, an international consortium led by the Health Policy Project, has developed and field tested a stigma measurement tool for use with health facility staff.

**Methods:**

Experts participated in a content-development workshop to review an item pool of existing measures, identify gaps and prioritize questions. The resulting questionnaire was field tested in six diverse sites (China, Dominica, Egypt, Kenya, Puerto Rico and St. Christopher & Nevis). Respondents included clinical and non-clinical staff. Questionnaires were self- or interviewer-administered. Analysis of item performance across sites examined both psychometric properties and contextual issues.

**Results:**

The key outcome of the process was a substantially reduced questionnaire. Eighteen core questions measure three programmatically actionable drivers of stigma within health facilities (worry about HIV transmission, attitudes towards people living with HIV (PLHIV), and health facility environment, including policies), and enacted stigma. The questionnaire also includes one short scale for attitudes towards PLHIV (5-item scale, *α =* 0.78).

**Conclusions:**

Stigma-reduction programmes in healthcare facilities are urgently needed to improve the quality of care provided, uphold the human right to healthcare, increase access to health services, and maximize investments in HIV prevention and treatment. This brief, standardized tool will facilitate inclusion of stigma measurement in research studies and in routine facility data collection, allowing for the monitoring of stigma within healthcare facilities and evaluation of stigma-reduction programmes. There is potential for wide use of the tool either as a stand-alone survey or integrated within other studies of health facility staff.

## Introduction

HIV-related stigma is a recognized barrier to HIV testing, disclosure of sero-status, linkage to care and adherence to anti-retroviral treatment (ART) [1–6]. While present in all spheres of life, stigma is particularly damaging within health facilities, where people living with or at risk of HIV must seek essential medical care, including ART. Stigma has been well documented within health facilities around the world [7–13], and in the past decade recognition of the importance of providing stigma-free health services has increased, which has led to progress in developing and testing different tools and intervention models for reducing stigma in such settings. These advances, however, have yet to be institutionalized as routine practice or implemented on a large scale.

Scale-up of stigma-reduction programmes in healthcare settings has been slow in part due to the lack of a brief, standardized tool for measuring stigma that works across diverse HIV prevalence, language and healthcare settings. While there exist a few validated research tools [9, 13–17], further use of them in research, evaluation or routine monitoring is hindered by several factors. Most of the tools have been tested in only one country or language, and ease of translation, understandability and local relevance of the tools across diverse contexts is unknown. In addition, though the validated tools often ask similar questions that capture the same stigma domains, the combination of items, the specific question wording and response categories vary. As a result, deciding which tool or items to use can be difficult. In addition, these variations pose challenges for national and/or global reporting systems that seek to track stigma within health facilities in a systematic, comparable way and over time.

Most validated tools focus exclusively on medical staff (e.g., doctors and nurses). However, studies have shown that people living with HIV (PLHIV) also encounter stigma and discrimination from administrators and non-medical staff [10]. Therefore, it is important to address and measure stigma among all levels of facility staff, including non-clinical personnel. Furthermore, most tools were developed for stigma-specific research studies and tend to be long and difficult to incorporate as a module into broader research or evaluation studies or to utilize for routine monitoring purposes.

To fill this measurement gap, a collaborative international effort led by the Health Policy Project (HPP) and composed of a broad range of individuals representing international programme-implementing agencies, university and non-university-based researchers, the global network of PLHIV (GNP + ) and UNAIDS, developed, tested and refined two brief tools for measuring HIV stigma among all levels of health facility staff. The first of these tools, the focus of this article, is tailored to evaluation and research needs. The second is suited for monitoring and situations where there are limited resources to collect data; it is a shorter version of the first [18]. Building on existing measures and with a focus on programmatic action to reduce stigma within health facilities, the tools cover multiple domains that capture enacted (experienced or manifested) stigma as well as the drivers of stigma within health facilities. These drivers include concern about HIV transmission when caring for PLHIV, attitudes towards PLHIV and a supportive health facility environment – a key factor in creating an enabling facility environment that supports staff to offer non-stigmatizing care. An enabling environment includes facility-level policies, safety supplies and training. This article describes a multi-year process and its key result – a brief questionnaire to measure stigma among health facility staff.

## Methods

Our methodological approach included a multi-step process: develop an item pool; review and prioritize items by experts through a workshop to develop the content of the questionnaire; field test the questionnaire in six countries; and analyze the data across sites to examine item performance. The objectives of the analysis across sites were to remove non-performing items and prioritize the remaining items to shorten the questionnaire while ensuring that it still captured the essential domains of stigma within health facilities.

### Item pool

The item pool was developed through a comprehensive literature search using PubMed and other bibliographic databases and included both published and grey literature, as well as some pre-publication questionnaires provided by workshop attendees [8, 9, 13, 16, 17, 19–26]. Seeking as wide an item pool as possible, broad inclusion criteria were applied. Articles, reports or unpublished questionnaires had to include quantitative measures implemented among at least one category of health facility staff and in one of the following domains: fear of HIV infection (including transmission knowledge); attitudes towards PLHIV and key populations (stereotypes and prejudice); observed (enacted stigma) and anticipated discrimination (which includes secondary stigma experienced by health facility staff); and institutional-level facilitators and barriers (facility policy and work environment). No geographic or date restrictions were applied. The final item pool was drawn from 10 peer-reviewed articles, 3 agency reports and 2 unpublished questionnaires. Of these only two were multi-country studies: one was an online study administered only in English and the other was concentrated in East and Southern Africa. In regard to study populations, six questionnaires collected data from a single discipline of medical practitioners, seven from multi-disciplinary medical practitioners and two from all levels of health facility staff. The length of surveys was often difficult to assess comparatively as many published articles only presented final scales, while others presented their full questionnaires. Length ranged significantly from 17 to 81 items or questions, with the majority being on the higher side (40–80 items).

### Content-development workshop

The content-development workshop brought together 22 international stigma measurement and programmatic experts, including PLHIV, in a 2.5-day workshop to review the item pool. This group brought experience from past or current work on stigma-reduction programming or measurement in Brazil, the Caribbean, China, Egypt, India, Kenya, Lesotho, Malawi, Mexico, Puerto Rico, South Africa, Swaziland, Tanzania, Vietnam and Zambia. In small working groups, participants reviewed, assessed and prioritized a comprehensive list of stigma items in key stigma domains that were specified in the item pool. The groups were asked to select items based on seven criteria:

1. Response is clearly attributed to or related to stigma.
2. Applicable across all categories of staff in a facility.
3. Relevant to diverse HIV prevalence, health systems and cultural contexts.
4. Ease of translation.
5. Potential for the questions to be influenced by gender, either of the respondent (healthcare provider) or of the client (if the question asks about actions or attitudes towards a client).
6. Potential of the question to cause/lead/reinforce stigma or discrimination.
7. Overall balance in the set to ensure data on measures are relevant to inform design and measure progress of stigma-reduction programmes.

Based on these criteria, each group was tasked with prioritizing the top two, five and 10 questions in a specific domain, and presented their recommendations back to the larger group for further discussion. Groups were also asked to consider whether there were any gaps in the existing item pool and if so, to propose new questions to fill these. Full workshop deliberations are available in an HPP report [27].

### Measures

Based on the outcomes of the content-development workshop, a questionnaire was developed for field testing [28] that included a background and four core content areas. Table 1 provides all the measures by questionnaire section, including: demographic, job type, and facility-related questions; drivers of stigma; observed and secondary stigma; and measures of stigma towards key populations and pregnant women living with HIV. Enacted stigma in health facilities was also measured by asking respondents whether they had observed specific behaviours or experienced secondary stigma related to caring for patients living with HIV.

**Table 1:** Summary of field-tested measures

Section	Category	Number of questions	Description
Background section	Demographic	6	Age, sex, relationship status, religion, education
	Job duties and facility-related	9	Current position, length of employment in the current job and in healthcare, type and location of facility, type of services provided by respondent, HIV patient case load and types of training received in the past 12 months

Drivers	Health facility policies and work environment	7	Availability of protective supplies (e.g., gloves and post-exposure prophylaxis), training (e.g., on confidentiality), existence and implementation of policies to protect PLHIV, how supportive the facility environment is for staff living with HIV
	Fear	1	Worry of contracting HIV while working with PLHIV; ranging from non-invasive (touching clothing) to invasive (drawing blood). Measures nine different situations (items)
	Attitudes towards PLHIV	1	Attitudes about PLHIV measured through agreement with six different statements (items)
	Shame	2	Two shame questions (e.g., I would be ashamed if I were infected with HIV)
	Willingness to treat key populations	1	Willingness to treat six different key populations including men who have sex with men, sex workers, people who inject drugs. Respondents who indicate unwillingness to treat, then asked whether it was for one of the four reasons

Enacted stigma	Observed	1	Specific behaviours (e.g., denial of care to PLHIV) that have been observed by the respondent in their facility in the last 12 months. Measures eight different behaviours (items)
	Extra infection precautions	1	Extra infection precautions that providers take with PLHIV but not with other patients. Measures six different actions (items)
	Secondary stigma	1	Stigma experienced because of caring for PLHIV (e.g., been avoided by friends or family because of caring for PLHIV); Measures four different actions (items)

Module: stigma towards pregnant women living with HIV among facility staff who care for pregnant women	Fear	1	Worry of contracting HIV during labour and delivery if woman is known to be living with HIV, or if her HIV status is unknown (two items)
	Opinions	1	Attitudes towards pregnant women living with HIV. Measures agreement with seven different attitudinal items.
	Observed	1	Specific behaviours (e.g., neglecting a women living with HIV during labour and delivery) that have been observed in the last 12 months. Measures five different behaviours (items)

### Field testing

The questionnaire was field tested in six sites: China (*n =* 300), Dominica (*n =* 335), Egypt (*n =* 300), Kenya (*n =* 350), Puerto-Rico (*n =* 301) and St. Christopher & Nevis (*n =* 307) between February 2012 and January 2013 (see Table 2 for country-specific dates). Sites for field testing were selected based on groups who participated in the content-development meeting and were able to raise funds to leverage their existing stigma research or programmatic efforts to field test the questionnaire. While the same core questionnaire and minimum sample size (300) were standard across sites, there were variations in types of facilities selected, categories of staff interviewed and methods of survey administration to accommodate site-specific contextual issues (Table 2). A key goal of this process was to develop and test a tool for all levels of facility staff, whether they are clinically trained or not. Therefore, respondents included all staff in a facility, from those who were medically trained at different levels (e.g., doctors, nurses, nurses assistants, dentists, pharmacists) to those who were not (e.g., receptionists, cleaning staff, ward attendants).

**Table 2:** Background information on questionnaire pilot sites

	China	Dominica	Egypt	Kenya	Puerto Rico	St Christopher & Nevis
HIV prevalence	Low	Low	Low	High	Low	Low

Questionnaire language	Chinese	English	Arabic	English, Dholuo, Swahili	Spanish	English

Mode of administration	Self (paper)	Self (paper), interviewer	Interviewer	Self (paper), interviewer	Self (iPad and paper)	Self (paper), interviewer

Date of data collection	April–May 2012	December 2012–January 2013	December 2012	May–June 2012	February–April 2012	November 2012

Ethical approvals from Institutional Review Boards	University of California, Los Angeles (UCLA), the Chinese Center for Disease Control and Prevention (CCDC)	National Human Research Ethics Committee of the Ministry of Health and the Health Media Lab's IRB	Egyptian Ministry of Health, Naval Medical Research Unit No. 3	Kenya Medical Institute (KEMRI) Ethical Review Committee and the University of Alabama at Birmingham (UAB)	University of Puerto Rico's Institutional Committee for the Protection of Human Subjects in Research (CIPSHI)	St. Christopher and Nevis Ministry of Health, Office of the Chief Medical Officer and the Health Media Lab's IRB

Type of facilities	Government County-level Hospitals	National Referral & District Hospitals Health Centers Clinics	Government Infectious Disease Hospital	Government District & Sub-district Hospitals, Health Centers, Dispensaries	Government HIV and STD Clinics, Private Hospitals and Clinics, Religious Community Based Organizations	National Referral & District Hospitals Health Centers Clinics

Number of respondents	300	335	300	350	301	307

Type of respondents^1^	Clinical	Clinical and non-clinical	Clinical and non-clinical	Clinical and non-clinical	Clinical and non-clinical	Clinical and non-clinical

Gender of respondents	Female: 65%; Male: 35%	Female: 82.1%; Male: 17.9%	Female: 74.7%; Male: 25.3%	Female: 56.3%; Male: 43.7%	Female: 72.8%; Male: 27.2%	Female: 81.9%; Male: 18.1%

1 Clinical staff includes those who are medically trained like doctors, nurses, nurse assistants, dentists, pharmacists, and non-clinical staff includes those who were not like receptionists, cleaning staff, ward attendants.

Questionnaires were self- or interviewer-administered, depending on literacy levels, respondent comfort levels with self-completion of the questionnaires, and site-specific contextual needs (Table 2). Interviewers introduced themselves, explained the survey, obtained informed consent and answered any questions that arose in the process of self-completion of the questionnaire. Confidentiality of responses was maintained by not collecting any personal identifiers and by respondents placing completed questionnaires in a sealed envelope or box. Each site obtained ethical clearance from their respective relevant country-level and institutional-level review boards (Table 2).

### Data analysis

Data entry and initial data cleaning were completed at each site and then sent to the global coordinating group for further cleaning and merging into a single, combined data set.

All analyses are conducted in STATA.SE, Version 12 [29]. Performance of the survey items across the six sites was assessed through both examination of psychometric properties and consideration of contextual issues. Initial analysis was conducted by the global coordinating team in preparation for the 2.5-day cross-site analysis workshop that brought together all the principal investigators for each site. During the workshop, the full team considered and discussed several aspects of each question when determining which ones to keep in the brief questionnaire. These aspects included:

1. Variable distributions by country to ascertain reasonable variability in responses.
2. Each site's experience implementing the questions.
3. Exploratory factor analysis or principle component analysis.

These three aspects were reviewed simultaneously and given equal weight when deciding the items that remained in the brief questionnaire.

Exploratory factor analysis was used when exploring the scale associated with attitudes towards PLHIV. For each country, we first ran exploratory factor analysis followed by a scree plot for eigenvalues to determine the number of factors in the scale. We considered potential items for removal if their factor loading was less than 0.35. Scale reliability was analyzed with Cronbach's alpha. Alphas of at least 0.7 are typically used as a cutoff to establish internally consistent scales. Given the goal to reduce the number of items in the scales and to make comparisons among groups, it was resolved to go with a lower yet acceptable cutoff of 0.6 at each of the sites [30–32] for the attitudinal scale.

The worry of HIV infection items included a “not applicable” response category because the items were related to job duty. If a respondent did not typically conduct the activity, they were prompted to select “not applicable.” As a result, when we ran exploratory factor analysis and scree plots by country on the nine items, our sample sizes were reduced considerably; in Egypt we found that none of the respondents answered all items. Therefore, we did not use factor analysis as a method for reducing items, but instead identified two criteria: all staff type can at least answer one item and identify a range of items based on procedure invasiveness to capture/reflect a continuum of worry.

Principle component analysis was used to reduce items in the remaining sections: observed stigma, secondary stigma, and health facility policies and work environment.

Combined with the above analyses, each site's experience implementing the questions was influential in determining inclusion status of each question. Consideration was given to question relevance across settings, in different levels of health facilities, for different levels of staff (ensuring a mix that was relevant to clinical and non-clinical staff), ease of translation and clarity of understanding. For example, if a question was not understood properly in one country, or it required additional explanation by interviewers, then there was a higher likelihood that the question was removed. In some sites, where questions were deemed important to retain in the brief questionnaire, but where choice of wording had compromised comprehension in some sites, the group rephrased the question based on recommendations from the field teams.

## Results

The main result of this collaborative process was a brief questionnaire that measures actionable drivers of stigma within health facilities.

### Questionnaires

The outcome of the content-development workshop was the field-tested questionnaire that combined the groups’ prioritized questions in each domain, plus background demographic information (see Table 1 for details of specific items). This questionnaire [28] has 18 core questions and, with sub-items included, 71–95 total items, depending on skip patterns (inclusive of the module). Workshop participants were also asked to identify any critical gaps in existing measures. Stigma towards key populations and health facility policies were two identified gaps. Questions were developed and field-tested to fill these gaps. In addition, workshop participants developed a module for measuring stigma towards pregnant women living with HIV to be implemented only among health facility staff providing services to pregnant women because of the added potential negative consequences of stigma for the health of pregnant women living with HIV and vertical transmission of HIV [33].

The finalized brief questionnaire for research and evaluation [34] is summarized in Table 3, which shows how many, and in which sections, questions were reduced from the field-tested questionnaire. This questionnaire has 17 core questions and, with sub-items included, 39–49 total items, depending on skip patterns (inclusive of the module). The questionnaires are available in five languages – Arabic, Chinese, English, Spanish and Swahili – along with an implementation guide in English. These are available at www.healthpolicyproject.com.

**Table 3:** Results of questionnaire item reduction by question types and totals

Section	Category	Field-tested questionnaire	Final brief questionnaire
Background section	Demographic	6 Questions	2 Questions
	Job duties and facility-related	9 Questions; 1 with 9 sub-items	5 Questions; 1 with 4 sub-items

Drivers	Health facility policies and work environment	7 Questions; 1 with 6 sub-items	5 Questions; 1 with 2 sub-items
	Fear	1 Question with 9 sub-items	1 Question with 4 sub-items^1^
	Attitudes towards PLHIV	1 Question with 6 sub-items	1 Question with 5 sub-items; 1 Question about HIV-positive women's right to have babies
	Shame	2 Questions	0 (as included as a sub-item in attitude question)
	Willingness to treat key populations	1 Question with 6 sub-items, each sub-item had, depending on answer, 4 additional possible questions	3 Questions focused on key populations of MSM, Sex workers and PWID. Each question has three possible sub-items, depending on answer

Enacted stigma	Observed	1 Question with 8 sub-items	1 Question with 3 sub-items
	Extra infection precautions	1 Questions with 6 sub-items	1 Question with 4 sub-items
	Secondary stigma	1 Question with 4 sub-items	1 Question with 3 sub-items^1^

Module: stigma towards pregnant women living with HIV among facility staff who care for pregnant women	Fear	1 Question with 2 sub-items	1 Question
	1 Question with 7 sub-items	1 Question with 4 sub-items	
	Observed	1 Question with 5 sub-items	1 Question with 5 sub-items

1 These questions are asked differently in high-prevalence and low-prevalence settings.

### Field questionnaire data

Data for the combined sample across the six sites (*n =* 1893) include the percentages for the country mean and ranges. (Each site will report separately on their individual results in future publications.) For several items large ranges were observed, a reflection of the diversity across the sites which includes HIV prevalence and health systems. The mean age of all respondents was 37.5 years, ranging from 32.5 to 40 years. The majority of respondents were female (mean=71.8%) ranging from 56.3 to 82.1%.

Table 4 presents the percentage mean and ranges for selected questions capturing drivers of stigma that were included in the brief questionnaire. Roughly, one in four respondents disagreed with the statement “I would never test a patient for HIV without the patient's informed consent.” More than half of respondents (54.5%) reported policies to protect PLHIV from discrimination in a facility. In terms of worry of HIV acquisition when caring for or providing services to PLHIV, as invasiveness of the procedure increased, worry also increased. On items in the attitude scale, the mean percentage agreement varied from a low of 15.7% for the statement “People living with HIV should feel ashamed of themselves” to 40.6% agreement to the statement “most people living with HIV do not care if they infect other people.”

**Table 4:** Stigma drivers, percentages and country ranges (*n=*1893)^1^

Health facility policies and work environment				

Level of agreement with the following statements^2^		Agree	Disagree	Do not know
I would never test a patient for HIV without the patient's informed consent	Mean	72.4	23.2	0.5
	Range	38.7–92.0	5.3–58.3	0.0–3.0
There are adequate supplies (e.g., gloves) in my health facility that reduce my risk of becoming infected with HIV	Mean	80.7	16.8	0.7
Range	53.7–96.7	2.3–46.3	0.0–4.0
There are standardized procedures/protocols in my health facility that reduce my risk of becoming infected with HIV	Mean	73.0	24.3	0.2
Range	10.0–93.4	5.3–88.7	0.0–1.3
		Yes	No	Do not know
My health facility has policies to protect patients living with HIV from discrimination	Mean	54.5	24.1	21.2
	Range	1.7–84.1	4.3–97.7	0.7–47.2
How hesitant are healthcare workers in this facility to work alongside a co-worker living with HIV regardless of their duties?^3^		Hesitant	Not hesitant	Do not know
Mean	51.5	42.3	0.4
	Range	22.0–83.4	16.3–75.3	0.0–2.7

**Worry related to contracting HIV when caring or providing services to people living with HIV**				

**Level of worry when conducting the following activities** 4		**Worried**	**Not worried**	

Took the temperature of a patient living with HIV (*n=*1205)	Mean	15.3	82.4	
	Range	5.3–43.4	56.6–90.5	
Touched the clothing of a patient living with HIV (*n=*1672)	Mean	23.3	74.7	
	Range	6.2–57.2	42.8–91.5	
Dressed the wounds of a patient living with HIV (*n=*1061)	Mean	59.6	37.5	
	Range	38.8–85.7	14.3–51.0	
Drew blood from a patient living with HIV (*n=*1052)	Mean	67.0	42.5	
	Range	44.1–83.0	17.0–49.6	

**Opinions about people living with HIV**				

**Level of agreement with the following statements** 2		**Agree**	**Disagree**	**Do not know**

HIV is a punishment for bad behaviour	Mean	16.3	82.1	
	Range	3.9–54.3	45.7–91.0	
Most people living with HIV do not care if they infect others	Mean	40.6	57.3	
	Range	15.0–69.0	31.0–84.4	
People living with HIV should feel ashamed of themselves	Mean	15.7	82.8	
	Range	5.3–54.7	45.3–94.7	
Most people living with HIV have had many sexual partners	Mean	35.8	62.4	
	Range	17.7–68.0	32.0–81.7	
People get infected with HIV because they engage in irresponsible behaviours	Mean	38.1	59.8	
	Range	21.1–69.0	31.0–78.0	
People living with HIV should be allowed to have babies if they wish	Mean	56.7	39.6	0.3
	Range	13.3–90.3	9.4–84.7	0.0–0.2
If I had a choice, I would prefer not to provide services to people who inject illegal drugs (*n=*1593)	Mean	17.6	78.4	
Range	11.9–35.7	64.3–85.1	
If I had a choice, I would prefer not to provide services to men who have sex with men (*n=*1593)	Mean	13.1	83.0	
Range	3.0–27.0	73.0–95.0	
If I had a choice, I would prefer not to provide services to sex workers (*n=*1593)	Mean	12.4	83.8	
	Range	5.7–29.7	70.3–93.4	

1 (*n*–1893) applies to each category, unless otherwise noted; % may not add to 100 because of missing data.

2 Response categories: strongly agree, agree, disagree, and strongly disagree; results collapse responses.

3 Response categories: very hesitant, somewhat hesitant, a little hesitant, and not hesitant; results collapse responses.

4 Response categories: very worried, worried, a little worried, a not worried; results collapse responses.

Table 5 presents the percentages for the mean and ranges of questions measuring enacted stigma that were included in the final questionnaire. The mean percent of respondents who reported observing a *healthcare worker talking badly about PLHIV or thought to be a PLHIV* was 29.9%. Use of extra infection precautions is present with 30.9% reporting wearing double gloves. Secondary stigma, however, is relatively low probably due to the fact that five of the six sites are in low HIV prevalence settings.

**Table 5:** Enacted stigma, combined percent (*n=*1893) and country ranges

Observed stigma (*n=*1853)			

In<12 months how often observed the following at your health facility^1^		At least once (%)	Never (%)
Healthcare workers unwilling to care for a patient living with HIV	Mean	23.4	74.4
	Range	12.7–43.1	56.9–87.3
Healthcare workers providing poorer quality of care to a patient living with HIV than to other patients	Mean	20.1	77.5
	Range	8.3–28.5	68.7–91.7
Healthcare workers talking badly about people living with or thought to be living with HIV	Mean	29.9	67.5
	Range	14.0–58.5	41.5–86.0

**Infection precaution measures**			

**Typically use any of the following measures when providing services to a patient living with HIV:**		**Yes (%)**	**No (%)**

Avoid physical contact (*n=*1575)	Mean	26.8	69.6
	Range	6.4–69.4	30.6–87.2
Wear double gloves (*n=*1506)	Mean	30.9	66.1
	Range	19.0–48.2	51.8–79.9
Use any special measures that you do not use with other patients (*n=*1495)	Mean	26.9	69.1
	Range	7.2–50.5	49.6–83.3

**Experiences with secondary stigma (*n=*1814)**			

**In the past 12 months, how often have you^1^:**		**At least once (%)**	**Never (%)**

Experienced people talking badly about you because you care for patients living with HIV	Mean	12.2	81.3
	Range	5.0–34.6	65.1–95.0
Been avoided by friends and family because you care for patients living with HIV	Mean	4.8	88.6
	Range	1.3–9.4	72.6–97.3
Been avoided by colleagues because of your work caring for people living with HIV	Mean	2.6	90.6
	Range	1.0–5.1	73.0–98.2

1 Response categories included most of the time, several times, once or twice and never.

### Attitude towards PLHIV scale

Table 6 presents the factor loadings for the attitude scale and reliability of the scale by country. The alpha for the combined sample was 0.78. Across all six countries only one factor formed but the items in the factor varied. In Kenya, Dominica and St. Christopher & Nevis all six items loaded on to the single factor, whereas in Puerto Rico and China, “PLHIV could have avoided HIV if they wanted to” (Q27a) did not load and in Egypt, “Most PLHIV do not care if they infect other people” (Q27c) did not load on the factor. While both items had reasonable variability across each country, during the content-development workshop, persons living with HIV stressed the importance of Q27c. Furthermore, the analysis workshop participants felt that Q27a was captured in another item “People get infected with HIV because they engage in irresponsible behaviors” (Q27f), and therefore, concluded to drop Q27a and keep Q27c in the attitude scale.

**Table 6:** Attitude scale: factor loadings and reliability

5-item attitude scale	China	Dominica	Egypt	Kenya	Puerto Rico	St. Christopher & Nevis
People living with HIV could have avoided HIV if they had wanted to (Q27a)	–	0.5340	0.6828	0.4588	0.3415	0.5657
HIV is a punishment for bad behaviour (Q27b)	0.5950	0.6155	0.8013	0.5152	0.6770	0.5302
Most people living with HIV do not care if they infect other people (Q27c)	0.3501	0.4383	–	0.4586	0.6202	0.6139
People living with HIV should feel ashamed of themselves (Q27d)	0.7047	0.6072	0.7308	0.4159	0.6513	0.4967
Most people living with HIV have had many sexual partners (Q27e)	0.5627	0.6434	0.6862	0.6463	0.6061	0.6759
People get infected with HIV because they engage in irresponsible behaviours (Q27f)	0.7078	0.6307	0.7737	0.6227	0.5869	0.5977

**Cronbach's *α* 5-item scale of Q27b–Q27f**	0.72	0.73	0.77	0.67	0.76	0.73

## Discussion

The results of this international multi-site collaborative effort demonstrate that it is possible to have a brief, standardized programmatic tool to measure stigma within health facilities that works well across diverse country contexts, prevalence areas, languages, healthcare settings and health worker types. The results (Tables 4 and 5) also demonstrate that while varying across sites, stigma is still prevalent across both the high- and low-prevalence sites and that there is still much work to be done to create a facility environment that fosters the delivery of stigma-free services. For example, the mean across all sites for agreement with the statement “most people living with HIV do not care if they infect others” was 40.6%, while only a little over half (54.5%) of respondents reported that their facilities had policies in place to protect patients living with HIV from discrimination. More than a third of respondents (39.6%) disagreed with the statement “People living with HIV should be allowed to have babies if they wish.” Respondents also report that they have observed healthcare workers unwilling to care for a patient living with HIV in their facility in the past 12 months (23.4% across sites) and a third (30.9%) report that they use double gloves when providing services to patients living with HIV.

The content of this tool is grounded in previous work measuring stigma among health providers, both on the level of individual questions and around the larger thematic areas of the questionnaire. Field testing of this instrument confirmed that the key domains measured and a sub-set (or similar) of the individual questions tested in previous work in single sites [8, 9, 11, 13, 14, 16, 17] worked across diverse contexts. To the best of our knowledge, only one other study [35] has tested measurement among a group of health providers (nurses) across multiple country sites (Lesotho, Malawi, South Africa, Swaziland and Tanzania). While all sites were in East and Southern Africa [13, 36], this work also demonstrated that use of a standard stigma data-collection tool for health providers across differing contexts is feasible. While not specific to healthcare providers, the work of Genberg et al. [37] also illustrated that a standard measurement tool for stigma can work across diverse settings (Thailand, Tanzania, South Africa and Zimbabwe) in the general population.

While the process demonstrated that a core set of questions works well to measure key domains for stigma-reduction programming in health facilities across diverse settings, the implementation process yielded several lessons, including lessons about the content of specific questions. This led to certain questions being dropped from the brief questionnaire, or if deemed too important to drop for programmatic reasons, being rephrased based on the field-testing experience. For example, asking about fear of HIV transmission in a high-prevalence context where many of the respondents may be living with HIV was problematic as phrased in the piloted questionnaire. Conversely, asking respondents about experiences of secondary stigma in low-prevalence settings had little relevance because respondents in these contexts provided care to so few PLHIV that it was unlikely anyone else would know to stigmatize them. However, while actual experience of secondary stigma was not particularly relevant in low-prevalence contexts, the anticipation that this might happen was considered relevant. These two issues were resolved by offering different question wordings for low- or high-prevalence HIV settings.

In addition, a few of the factor loadings and the Cronbach’s *α* for the opinion scale were slightly lower for Kenya than the other sites. As Kenya was the only high HIV-prevalence field-testing site, it could be that this reflects the respondents’ longer experience and exposure to HIV and HIV programming, higher likelihood of personally knowing PLHIV, or possibly the fact that a sub-set of the respondents were likely living with HIV. Implications for framing of attitudinal questions (apart from the distinctions described above) are unclear, however, in the absence of more field testing in additional high-prevalence countries.

The questions that were deemed too important to drop, but needed re-wording based on the field implementation experience, came from two domains that were identified as gaps during the initial content-development meeting – key populations and facility policies. They therefore comprised new questions developed by the meeting participants, as opposed to questions that had already been tested in other instruments.

An example of a facility policy question that did not work well as phrased was: “My health facility has policies to protect patients living with HIV from discrimination (response categories: Yes, No, DK).” The challenge with this question was a lack of specificity in the understanding or interpretation of what a *policy* means across the sites. The question was thus rephrased to read: “My health facility has written guidelines to protect patients living with HIV from discrimination.” Another question that required re-wording focused on willingness to provide services to a specific key population. The piloted version of the question had the following question stem: “Please tell us it you strongly agree, agree, disagree, or strongly disagree with the following statement in relation to each group listed in the table below. I would prefer not to provide services to …” (and then listed multiple key population groups). The challenge discovered with this question was that despite the use of the word “prefer,” respondents answered that they would provide services (even if they preferred not to) because they did not think they had a choice in the matter. Based on recommendations from the field testing experience, the question was re-worded to read: “If I had a choice, I would prefer not to provide services to ….”

On the implementation side, key lessons learned focused on mode of administration (self- or interviewer-administered). For example, in Egypt all data were collected through interviewer-administered questionnaires, as that was deemed most context-appropriate, while in other sites a mixture of self- and interviewer-administered was most appropriate. Anonymity was also of concern in some sites even though no identifiers were collected and self-filled questionnaires were returned in manner that ensured confidentiality. This concern seems to have stemmed from the set of background questions asked and worry that somehow this information could be pieced together to identify a particular respondent. This was of particular concern in the two island nations where small populations meant that almost half of all staff working in the health facilities in the country were interviewed. To respond to this concern, the brief questionnaire now includes only a limited number of essential background questions and the recommendation that implementers use a facility code if they require specific information on types of facilities, rather than asking respondents for this information. In Puerto Rico, half the self-administered sample was delivered with paper and pencil, the other using iPads. While further analysis needs to be conducted, the initial feedback indicates that use of iPads provides a better method of administration, both peaking respondents interest in participating in order to use the technology while also providing more trust in the anonymity of the questionnaire. In addition, the automatic skip patterns in the iPad questionnaire ensured ease of completion and reduced errors.

### Limitations

The process did have limitations. The purpose of this effort was to demonstrate feasibility and applicability of a shortened tool that could be used in programmatic applications across a diverse set of contexts and languages. It had to allow for variability by site in some key factors, and be responsive to resource constraints. It therefore was not conducted in accordance with standard methodology for scale validation. For example, the health worker sampling methods varied across sites, sites varied in their mode of administration, and the tool was not validated against any similar constructs or outcomes. As with any data collection on sensitive issues, there can be social desirability bias in responses, and this appears to have manifested in non-response to several questions in the Caribbean sites, where the most concerns around confidentiality emerged due to small size of the health facility workforce. Interestingly, the questions that field staff indicated as most likely to be subject to social desirability bias were questions that respondents perceived would put the facility, rather than themselves, in poor light. For example, some participants responded that gloves were always available in the facilities, when the research team in fact knew they were not. While the questionnaire was field tested in six sites covering diverse contexts and in multiple languages, these sites are not fully representative of all regions or languages of the world, and five of the six sites were low HIV-prevalence contexts. Therefore, it may be important to conduct brief pilots when implementing the tool in new contexts or languages to determine the interpretability of the new translation and appropriate mode of administration.

While there are some limitations with the tool, it also has many strengths including: covering the key HIV stigma domains shown to be important for stigma-reduction programming in health facilities in a brief manner; being evidence-based, drawing on validated tools from the literature; and successful administration in multiple diverse country settings and languages. A particular strength is the shorter length of the questionnaire, which is important for busy and resource-constrained health facilities. The reduced length also allows the questionnaire to be used as a stand-alone tool in routine monitoring, and/or as part of a larger evaluation of country-level or health facility-level activities.

## Conclusions

The purpose of this study was to develop and test a standardized tool that assesses HIV stigma in healthcare settings. The development, field testing and analysis process carried out by this team demonstrate that a brief yet comprehensive instrument that captures key domains of stigma for programmatic action can be successfully implemented across diverse settings and provide consistent and robust results. The brief tool is now available for government officials, policy makers and programmers to determine the amount of HIV stigma in health facilities, design evidence-based programming responses to reduce stigma, monitor stigma over time, and evaluate the effects of stigma-reduction interventions and programmes. There is potential for wide use of this tool, both as a stand-alone survey or integrated within other health facility surveys. Areas of future work for this tool are to observe how it performs with repeated administrations over time, in additional contexts (particularly high-prevalence settings), and to triangulate data collected in health facilities on stigma and discrimination with data being collected among PLHIV and key population clients of health facilities, for example by the stigma index programme (http://www.stigmaindex.org/). Further work is needed to test and expand questions measuring stigma towards key populations.

Institutionalizing the measurement of stigma as routine practice, and doing so on a large scale, could strengthen the delivery of high-quality care, improve patient outcomes and satisfaction, improve the work environment for health facility staff, and increase the effectiveness of investments in HIV prevention, care and treatment. This brief tool can thus contribute to addressing HIV stigma within health facilities and towards progress in ensuring that PLHIV, and people often associated with HIV, receive high-quality health services and that their rights and privacy are upheld.

## References

[1] Kalichman SC, Simbayi LC (2003). HIV testing attitudes, AIDS stigma, and voluntary HIV counselling and testing in a black township in Cape Town, South Africa. Sex Transm Infect.

[2] MacQuarrie K, Eckhaus T, Nyblade L (2009). HIV-related stigma and discrimination: a summary of recent literature.

[3] Mahajan A, Sayles J, Patel V, Remien R, Sawires S, Ortiz D (2008). Stigma in the HIV/AIDS epidemic: a review of the literature and recommendations for the way forward. AIDS.

[4] Nyblade L, Pande R, Mathur S, MacQuarrie K, Kidd R, Bangeyerga H (2003). Disentangling HIV and AIDS stigma in Ethiopia, Tanzania and Zambia.

[5] Pulerwitz J, Michaelis AP, Lippman SA, Chinaglia M, Diaz J (2008). HIV-related stigma, services utilization, and status disclosure among truck drivers crossing the southern borders in Brazil. AIDS Care.

[6] Smith RA, Morrison D (2006). The impact of stigma, experience, and group referent on HIV risk assessment and HIV testings intentions in Namibia. Soc Sci Med.

[7] Letamo G (2005). The discriminatory attitudes of health workers against people living with HIV. PLoS Med.

[8] Mahendra VS, Gilborn L, Barat S, Mudoi R, Gupta I, George B (2007). Understanding and measuring AIDS-related stigma in healthcare settings: a developing country perspective. SAHARA J.

[9] Nyblade L, MacQuarrie K, Phillip F, Kwesigabo G, Mbwambo J, Ndega J Measuring HIV stigma: results of a field test in Tanzania.

[10] Nyblade L, Stangl A, Weiss E, Ashburn K (2009). Combating HIV stigma in health care settings: what works?. J Int AIDS Soc.

[11] Reis C, Heisler M, Amowitz LL, Moreland RS, Mafeni JO, Anyamele C (2005). Discriminatory attitudes and practices by health workers toward patients with HIV/AIDS in Nigeria. PLoS Med.

[12] Turan JM, Miller S, Bukusi EA, Sande J, Cohen CR (2008). HIV/AIDS and maternity care in Kenya: how fears of stigma and discrimination affect uptake and provision of labor and delivery services. AIDS Care.

[13] Uys LR, Holzemer WL, Chirwa ML, Dlamini PS, Greeff M, Kohi TW (2009). The development and validation of the HIV/AIDS stigma instrument – nurse (HASI-N). AIDS Care.

[14] Froman RD, Owen SV (1997). Further validation of the AIDS attitude scale. Res Nurs Health.

[15] Rutledge SE, Whyte J, Abell N, Brown KM, Cesnales NI (2011). Measuring stigma among health care and social service providers: the HIV/AIDS provider stigma inventory. AIDS Patient Care STDs.

[16] Stein JA, Li L (2008). Measuring HIV-related stigma among Chinese service providers: confirmatory factor analysis of a multidimensional scale. AIDS Behav.

[17] Varas-Diaz N, Neilands T (2008). Development and validation of a culturally appropriate HIV/AIDS stigma scale for Puerto Rican health professionals in training. AIDS Care.

[18] Health Policy Project (2013). Measuring HIV stigma and discrimination among health facility staff: brief questionnaire.

[19] Brems C, Johnson ME, Warner TD, Roberts LW (2010). Health care providers’ reports of perceived stigma associated with HIV and AIDS in rural and urban communities. J HIV/AIDS Soc Serv.

[20] Ekstrand ML, Ramakrishna J, Bharat S, Heylen E Prevalence and drivers of HIV stigma among health providers in urban India: implications for interventions.

[21] Froman RD, Owen SV, Daisy C (1992). Development of a measure of attitudes toward persons with AIDS. J Nurs Scholarsh.

[22] Harrison M, Fusilier MR, Worley JK (1994). Development of a measure of nurses’ AIDS attitudes and conservative views. Psychol Rep.

[23] Health Policy Initiative Task Order 1 (2010). Measuring the degree of HIV-related stigma and discrimination in health facilities and providers: working report.

[24] Lohiniva AL (2011). Challenging stigma and discrimination against PLHA in Egypt, Presentation at the ‘Measuring stigma and discrimination in health care settings’ meeting.

[25] Oanh KTH, Ashburn K, Pulerwitz J, Ogden J, Nyblade L (2008). Improving hospital-based quality of care in Vietnam by reducing HIV-related stigma and discrimination. Horizons Final Report.

[26] O'Hea El, Systma SE, Copeland A, Brantley PJ (2001). The attitudes toward women with HIV/AIDS scale (ATWAS): development and validation. AIDS Educ Prev.

[27] Nyblade L, Hunger E (2011). Measuring HIV-related stigma and discrimination in healthcare settings: deliberations of an expert panel.

[28] Health Policy Project (2012). Measuring HIV stigma and discrimination among health facility staff: field-tested questionnaire.

[29] StataCorp (2011). Stata statistical software: release 12.

[30] Ware JE, Snow KK, Kosinski M, Gandek B (1993). SF-36 Health Survey: manual and interpretation guide.

[31] Nunnally JC, Bernstein IH (1994). Psychometric theory.

[32] Helmstadter GC (1964). Principles of psychological measurement.

[33] Turan JM, Nyblade L (2013). HIV-related stigma as a barrier to achievement of global PMTCT and maternal health goals: a review of the evidence. AIDS Behav.

[34] Health Policy Project (2013). Measuring HIV stigma and discrimination among Health Facility Staff: comprehensive questionnaire.

[35] Holzemer WL, Makoae LN, Greeff M, Dlamini PS, Kohi TW, Chirwa ML (2009). Measuring HIV stigma for PLHAs and nurses over time in five African countries. SAHARA J.

[36] Chirwa ML, Greef M, Kohi TW, Naidoo J, Makoae LN, Dlamini PS (2009). HIV stigma and nurse job satisfaction in five African countries. J Assoc Nurs AIDS Care.

[37] Genberg BL, Kawichai S, Chingono A, Sendah M, Chariyalertsak S, Konda KA (2008). Assessing HIV/AIDS stigma and discrimination in developing countries. AIDS Behav.

# Sub article: Access to justice: evaluating law, health and human rights programmes in Kenya

**DOI:** 10.7448/IAS.16.3.18726

**Published:** 2013-11-13

**Authors:** Sofia Gruskin, Kelly Safreed-Harmon, Tamar Ezer, Anne Gathumbi, Jonathan Cohen, Patricia Kameri-Mbote

**Affiliations:** 1 Program on Global Health and Human Rights, Institute for Global Health, University of Southern California, Los Angeles, CA; 2 Open Society Foundations, Public Health Program, Law and Health Initiative, New York, NY; 3 Open Society Foundations, Initiative for East Africa, Nairobi, Kenya; 4 Open Society Foundations, Public Health Program, New York, NY; 5 International Environmental Law Research Centre, Strathmore University, Nairobi, Kenya

**Keywords:** HIV, AIDS, human rights, programme evaluation, public health, stigma, discrimination

## Abstract

**Introduction:**

In Kenya, human rights violations have a marked impact on the health of people living with HIV. Integrating legal literacy and legal services into healthcare appears to be an effective strategy to empower vulnerable groups and address underlying determinants of health.

**Methods:**

We carried out an evaluation to collect evidence about the impact of legal empowerment programmes on health and human rights. The evaluation focused on Open Society Foundation-supported legal integration activities at four sites: the Academic Model of Providing Access to Healthcare (AMPATH) facility, where the Legal Aid Centre of Eldoret (LACE) operates, in Eldoret; Kenyatta National Hospital's Gender-based Violence Recovery Centre, which hosts the COVAW legal integration program; and Christian Health Association of Kenya (CHAK) facilities in Mombasa and Naivasha. In consultation with the organizations implementing the programs, we designed a conceptual logic model grounded in human rights principles, identified relevant indicators and then coded structure, process and outcome indicators for the rights-related principles they reflect. The evaluation included a resource assessment questionnaire, a review of program records and routine data, and semi-structured interviews and focus group discussions with clients and service providers. Data were collected in May–August 2010 and April–June 2011.

**Results:**

Clients showed a notable increase in practical knowledge and awareness about how to access legal aid and claim their rights, as well as an enhanced ability to communicate with healthcare providers and to improve their access to healthcare and justice. In turn, providers became more adept at identifying human rights violations and other legal difficulties, which enabled them to give clients basic information about their rights, refer them to legal aid and assist them in accessing needed support. Methodological challenges in evaluating such activities point to the need to strengthen rights-oriented evaluation methods.

**Conclusions:**

Legal empowerment programmes have the potential to promote accountability, reduce stigma and discrimination and contribute to altering unjust structures and systems. Given their apparent value as a health and human rights intervention, particularly for marginalized populations, further rigorous evaluations are called for to support the scale-up of such programmes.

## Introduction

Integrating legal support into health services is an important strategy for enabling people who are socially marginalized to access justice and address human rights violations that undermine their health [1, 2]. It facilitates holistic care and the realization of rights that have significance for underlying determinants of health, such as the right to education, to an adequate standard of living and to protection from violence and discrimination. It is a particularly valuable mechanism for improving access to justice in settings where people are vulnerable because of their gender, age or health condition.

“Legal integration programmes,” as defined here to mean programmes incorporating legal aid, training and representation into existing health services to improve health outcomes and advance human rights, represent a relatively new approach to addressing structural dimensions of health. The earliest examples come from the United States, where “medical legal partnerships” seek to improve the health and well-being of children, the elderly, the poor and immigrants by eliminating barriers to healthcare and addressing environmental factors that impact health [3]. More recently, the Open Society Foundations’ Law and Health Initiative has funded legal integration programmes in Georgia, Kenya, Macedonia, South Africa, Uganda and Ukraine. These programmes serve vulnerable groups such as people affected by HIV, people in need of palliative care, survivors of gender-based violence, sex workers, Roma and people who use drugs.

In the context of the global HIV response, legal empowerment has begun receiving recognition as an important indicator of health enhancement. The Joint UN Programme on HIV/AIDS cites legal empowerment as a key intervention in national HIV responses [4], while the Global Fund to Fight AIDS, Tuberculosis and Malaria has awarded funding to a number of legal empowerment projects to help them expand their reach and attain key health milestones [5, 6]. That said, it is not yet clear how legal support can best be integrated into health services, within and beyond the field of HIV. Likewise, there is not yet consensus on how best to assess the impact of this work on reducing stigma and discrimination, or on improving health outcomes. Given the key role of stigma as a barrier to HIV prevention and treatment [7, 8], investigating the potential for legal integration programmes to counter HIV-related stigma should be a high priority.

This article presents findings from an evaluation of three Open Society-funded legal integration programmes, all administered by Kenyan nongovernmental organizations (NGOs) (Box 1). The Christian Health Association of Kenya (CHAK), a major provider of HIV-related services in Kenya, implements a legal integration programme through 15 of its 76 health centres and hospitals. The Legal Aid Centre of Eldoret (LACE) is based at a single healthcare facility within the Academic Model of Providing Access to Healthcare (AMPATH) network. AMPATH, a partnership between Kenyan and US academic medical centres, established LACE to represent people whose access to justice is otherwise limited, particularly people living with HIV. The Coalition on Violence against Women (COVAW), a Kenyan human rights organization, established its legal integration programme at a post-rape care centre within Kenyatta National Hospital in 2007.

Specifically we explored how the structure and implementation of legal integration programmes can further human rights principles, how such programmes have the potential to contribute to improved health outcomes and how they advance access to judicial and other forms of redress. These findings, which represent one of the first rigorous evaluations of legal empowerment programmes, suggest that legal empowerment might be a critical health and human rights intervention, particularly for marginalized populations.

***Box 1*. Overview of Kenyan legal integration programmes included in the evaluation**

**The Legal Aid Centre of Eldoret (LACE)** was founded in 2008 to represent people whose access to justice is otherwise limited, particularly people living with HIV. LACE is based within one of the centres operated by the Academic Model of Providing Access to Healthcare (AMPATH), a partnership between Kenyan and US academic medical centres. LACE accepts client referrals from AMPATH staff and also serves clients in the Eldoret community at large.

**The Coalition on Violence against Women (COVAW)** is a Kenyan human rights organization working to eradicate all forms of violence against women. COVAW began its legal integration programme in 2007. The first legal integration site was established at the Gender-based Violence Recovery Centre, a post-rape care centre at Kenyatta National Hospital. Services include direct legal aid, referral to other sources of legal aid and training for clients and service providers on human rights, gender-based violence and related topics.

**The Christian Health Association of Kenya (CHAK)** operates 435 health facilities throughout Kenya, including 25 hospitals. CHAK provides a broad range of HIV-related services, and 20 of its hospitals offer comprehensive HIV care and support. CHAK's legal integration programme seeks to empower people living with HIV by integrating legal services and rights awareness at 10 CHAK hospitals, and a scale-up to five additional sites is underway.

## Methods

### Evaluation model

In consultation with the NGOs implementing the programmes, we designed a conceptual logic model to guide efforts to answer the research questions using both quantitative and qualitative methods (Figure 1). The structure components of the model reflect broad categories of resources utilized by the programmes, and the process and outcome components are based on commonly defined activities and objectives. Drawing on the UN Statement of Common Understanding on Human Rights-Based Approaches, we defined long-term impact as building the capacity of rights holders and duty bearers to claim and fulfil rights to improve quality of life for vulnerable groups [9].

**Figure 1:** Human rights in logic model development (CARE, CHAK and COVAW's legal integration programmes).

A unique feature of the logic model is its attention to the principles of a human rights-based approach, emphasizing participation, accountability, non-discrimination, empowerment and linkage to other rights [9, 10]. We conceptualized the components of the logic model in ways that acknowledged the centrality of specific rights to the work of the programmes – the rights to health, information, education, an adequate standard of living, justice and security of person. In accordance with General Comment 14 of the United Nations Committee on Economic, Social and Cultural Rights, we focused on four key elements of the right to health: the availability, accessibility, acceptability and quality of the goods and services delivered [11].

### Evaluation instruments

The research team identified relevant indicators for all structure, process and outcome components of the logic model, then coded them for the human rights-related principles they reflect. These principles were taken into account during the development of both the quantitative and qualitative evaluation instruments. (The evaluation instruments are described in Supplementary files)

### Data collection

The evaluation focused on Open Society Foundations-supported legal integration activities at the following sites: the AMPATH facility, where LACE operates, in Eldoret; Kenyatta National Hospital's Gender-based Violence Recovery Centre, which hosts the COVAW legal integration programme; and CHAK facilities in Mombasa and Naivasha. The first phase of data collection occurred in May–August 2010, and the second phase occurred in April–June 2011 in order to assess improvements within this time period.

NGO staff independently completed the resource assessment questionnaires, which asked about financial, human, technical and information resources. Quantitative data were captured using existing programme records and routine data. Semi-structured interviews and focus group discussions with clients and service providers were carried out in English or Swahili, depending on which language the person preferred. Swahili transcripts were later translated into English. In some instances, interview and focus group data were collected from both intervention participants and controls. The qualitative case review worksheet was completed by each programme's legal officer, and interviews with the legal officers further contributed to the case reviews.

### Data analysis

Qualitative assessments of resource availability and constraints were made on the basis of resource assessment questionnaires. Quantitative indicators for legal service provision and referral were tabulated according to the records and data provided by the programmes. Interview and focus group discussion transcripts were coded and analysed using qualitative analysis software (NVivo9), with attention to key words, phrases and themes highlighted in the logic model. Case review worksheets were analysed qualitatively, and were further analysed alongside transcripts of qualitative interviews. Findings were grouped according to the evaluation's central areas of inquiry: the nature of the interventions, in terms of the specific training, legal aid and referral activities, and the effects of the interventions, in terms of human rights knowledge and awareness, satisfaction with services and improvements in legal protection and redress.

## Results

Focusing in particular on the process and outcome elements of the logic model, this section highlights key results with potential import to the structure and implementation of legal empowerment programmes more generally.

### Training of clients and providers on legal and human rights issues

Training was a major focus of all three programmes.

LACE reported conducting several trainings on HIV and human rights for clients, most of whom are people living with HIV. The trainings addressed human rights concepts, along with practical legal skills such as writing a will. LACE also trained AMPATH health workers and community health workers in related areas, including child protection.

CHAK client-training efforts capitalized on the existence of well-established support groups made up of and led by people living with HIV. While some trainings were provided directly to support groups, others used a “training of trainers” model to prepare group leaders to educate their fellow group members about human rights. Many also had links to legal aid. For example, a 2009 training for community members, most of whom were people living with HIV, was integrated with a legal clinic at CHAK's Mombasa site. As was generally the case, the training addressed marriage law, succession law, and gender and its impacts on HIV. Immediately following the training, participants had the opportunity to obtain legal information and referrals from on-site lawyers. The training led to at least three cases being carried forward via referrals. CHAK also reported providing human rights training to staff in its HIV, malaria and tuberculosis divisions, again using a “training of trainers” model, where the heads of different clinical departments were then responsible for disseminating information within their departments.

COVAW reported that it regularly carries out legal trainings for Kenyatta National Hospital staff, and for its clients and other community members. Training results were not made available to evaluators as the trainings took place independently of the Open Society Foundations initiative.

### Provision of multiple types of legal aid

The evaluation clearly indicated that the provision of legal representation in formal judicial processes was only one of multiple types of legal empowerment welcomed by clients.

LACE often tried to help clients resolve cases through informal conflict resolution mechanisms. For example, LACE received a complaint from a woman whose husband had deserted her and seven children after learning that she was living with HIV. The husband's family had taken over land that the husband had allocated to his wife. After receiving letters from LACE, the husband's family agreed that the woman could cultivate the land. LACE was later able to persuade the husband to formally transfer the land to his wife's ownership.

LACE's legal aid records document the provision of services to almost 450 clients from the time of the programme's founding in September 2008 through the first half of 2010. LACE reported assisting clients with numerous types of legal documents, including parental responsibility agreements, letters of demand, letters inviting parties for negotiation, birth and death certificates, wills and affidavits. LACE also reported working with clients, prosecutors and the police in criminal cases to facilitate the proper handling of cases.

Most COVAW clients accessed legal services after presenting for medical care. COVAW's legal officer informed clients of legal options such as bringing charges against perpetrators of sexual violence as well as the opportunity to pursue informal conflict resolution. In some cases, the legal officer went on to provide direct legal representation for clients, and in others, COVAW helped clients acquire representation elsewhere. COVAW reported helping clients draft or obtain legal documents relevant to their cases, as well as assisting external paralegals who were working with clients on legal documents.

For evaluation purposes, COVAW was able to provide records for 73 legal aid cases that it handled between January and July 2010. Most clients received only legal information or advice. Two clients additionally received access to informal conflict resolution mechanisms. Eleven clients received referrals to non-legal services, primarily psychological support services. Although only one client received formal legal representation, COVAW was “pursuing” four other cases at the time of the evaluation.

CHAK staff highlighted the value of handling some types of cases at the community level with the assistance of chiefs and other local leaders, most commonly in inheritance and succession cases. For example, CHAK worked with a client who had been unsuccessful in securing the help of her community chief after she was disinherited by her stepsons. CHAK sent a demand letter to the stepsons, copying the letter to the chief, who then became involved in negotiations that resulted in the woman receiving a sufficient financial settlement.

CHAK was unable to provide records of individual legal cases at one of the two sites included in this evaluation, and it had records for only 18 cases at the other site, two-thirds of which occurred in the first half of 2010. Clients in one-third of the cases received access to informal conflict resolution mechanisms. Clients in three of the 18 cases received referrals to non-legal services such as medical services. None of the clients received formal legal representation.

### Referrals

Formal structures for referring clients to legal and non-legal services were found to be crucial to the ability of all three programmes to meet the demand for services. Referrals were made to legal aid organizations, pro bono lawyers, local leaders, government officials and the police. For example, LACE referred clients to the District Labour Officer, District Children's Officer and State Counsel. COVAW commonly sent clients to pre-identified pro bono lawyers for cases requiring litigation. Reflecting the range of services needed for individuals to realize their rights, the programmes’ use of non-legal referrals included referrals to medical services, counselling services, a women's shelter, a “family preservation initiative” and other sources of psychosocial and economic support.

Real and perceived corruption among community leaders, government officials and the police reportedly undermined reliance on referral structures. According to CHAK's legal officer, many clients did not follow through when they were referred to community chiefs because they perceived the chiefs to be either disinterested or corrupt. LACE reported many problems with the police, including cases in which police accused survivors of gender-based violence of giving false evidence and arrested them instead of their perpetrators.

### Knowledge and awareness of human rights and legal issues

Evaluators used focus group data to compare LACE, COVAW and CHAK clients who had received training on legal and human rights issues to control groups of untrained clients. While both sets of clients exhibited general conceptual familiarity with human rights, trained clients appeared to have greater awareness of how and where to access legal services to safeguard their rights. Some trained clients spoke emphatically about how learning about human rights had transformed their outlook or their approach to challenges.

We have been trained on our rights, and we can now confidently talk about our rights before the chief and village elders. (Trained LACE client)

Through [the legal integration program], I know I have basic rights of food, clothing and shelter … We are in hard economic circumstances … and at times we are forced to go to the streets to say that we have a right to food. I'm never afraid to go to the streets because I have been trained on my rights through COVAW. (Trained COVAW client)

[Training] has given us strength and passion to work … We can now educate a person how to live well, be secure and also how to fight for his property. (Trained CHAK client)

Evaluators also used focus group data to compare groups of service providers trained by LACE, COVAW and CHAK to untrained groups. The trained service providers appeared to be better equipped to provide legal and rights-related information and referrals to clients.

From CHAK's training, we came to understand how we can communicate to the patients. Now we have learnt that human rights apply to everybody. Everybody has a right to be treated. We are rolling down that information to the community, and the community is becoming aware of their human rights. (Trained CHAK service provider)

### Service provision and satisfaction with services

In evaluating client and service provider perspectives on the effects of legal integration on service provision and satisfaction with services, comparisons with control groups (people receiving health-related services but not legal training) suggested that legal integration programme clients developed greater access to legal and non-legal resources and felt more empowered. While the benefits of healthcare and social services also supported these feelings, focus group participants identified a clear link with some aspects of the legal services offered by LACE, COVAW and CHAK. They associated the services with, among other issues, being able to secure property, provide financially for loved ones, seek legal redress, speak out against sexual violence, form support groups and engage in advocacy on behalf of others, including people living with HIV.

Service providers who had received training from LACE, COVAW or CHAK reported multiple benefits. They described themselves as being better able to inform clients about their rights, help clients with minor legal matters and refer clients to relevant services.

Service providers’ observations about legal integration contributing to client empowerment corresponded to what clients reported about feeling more empowered as a result of their experiences with all three programmes. For example, trained CHAK providers echoed trained CHAK clients’ views that associated the programme's efforts with clients becoming more comfortable asserting their rights in their interactions with providers.

### Protection and redress for rights violations

The LACE, COVAW and CHAK programmes contributed to improving protection and redress in numerous ways. Health-related human rights issues addressed through casework included discrimination, defamation, land and property ownership, access to housing, probate, debt collection, child maintenance and support, and sexual and gender-based violence. Human rights principles addressed through these efforts included the rights to participation, education, an adequate standard of living, housing and shelter, property ownership, non-discrimination, security of person and justice. In particular, the act of helping clients gain access to legal aid is in itself a means of ensuring the right to justice.

Referrals to non-legal services constituted an important way in which LACE, COVAW and CHAK advanced a range of health-related human rights. For example, CHAK referred a 14-year-old girl who had been sexually assaulted by her stepfather to a CHAK clinic where she could access HIV care and treatment, as well as to a child services organization for psychosocial support. COVAW provided a referral for medical management for a 15-month-old girl who was in her grandmother's custody, as well as referring the grandmother to counselling.

The programmes further sought to systematically improve shared accountability for human rights and shared protection from rights violations. For example, LACE created a network of doctors, nurses, community chiefs and police officers to promote a more effective response to cases of sexual and gender-based violence. LACE's legal officer credited the network with making some chiefs and members of the police more inclined to address rights violations brought to their attention. CHAK established a human rights “watchdog” group in one community. Participants – including the chief, assistant chief, church elders, health workers and trained clients – underwent training to monitor and report human rights violations.

### Obstacles impacting programme effectiveness

Evaluation findings call attention to structural barriers impacting the effectiveness of legal empowerment programmes. Corruption, inaction and mishandling of cases by police were major concerns expressed by informants, particularly in relation to sexual and gender-based violence. Real and perceived corruption and indifference among community chiefs and government officials were other barriers cited, impacting both formal and informal legal action. There were also clients who feared utilizing formal mechanisms because of perceived resource implications or the danger of retaliation from the parties accused of wrongdoing; in some cases, clients received explicit threats.

Another notable obstacle identified through the evaluation was inadequate linkage within health facilities to the legal empowerment programmes. Finally, high staff turnover within the organizations due to high demand across Kenya for staff with legal skills was recognized as a critical barrier. This resulted in the loss of institutional knowledge, undermined programme continuity and limited the value of staff training. Closely related are the challenges associated with acquiring resources to pay staff adequately and ensure long-term programme sustainability.

## Discussion

To our knowledge, no formal evaluations of the rights and health impacts of legal integration programmes have been published to date. Such efforts are necessary not only to inform programme evaluation but also to provide guidance to those who wish to provide effective services in the future.

Evaluation of the three programmes demonstrates health and rights-related benefits associated with training clients and healthcare providers on legal and human rights issues, providing legal aid to clients and referring clients to legal and non-legal resources. The LACE, COVAW and CHAK programmes appear to make positive contributions to clients’ and providers’ awareness of human rights and legal issues, client empowerment and some aspects of health service provision. The programmes also appear to advance the rights of clients and community members at large, as well as to facilitate access to redress for rights violations.

Evaluation findings also call attention to the importance of legal integration programmes looking beyond the courtroom. As noted, it was often more appropriate for LACE, COVAW and CHAK to address clients’ legal needs without engaging with the formal judicial system. In some cases, clients simply needed help with legal documents. In others, the heavy resource requirements associated with utilizing the judicial system made working through informal channels more practical. There were also cases in which fear of retaliation kept clients from initiating civil or criminal proceedings. In those situations, informal conflict resolution offered an alternate means by which clients still might attain justice.

Informal efforts to resolve legal problems, in fact, stood out as a major strength of the three programmes. While the concept of informal conflict resolution has various meanings, evaluation findings suggest the benefits of two types of engagement: work carried out entirely by the programmes themselves, and work that engages community-based mechanisms such as negotiations involving chiefs and community elders.

Referrals may constitute another important means of providing clients with access to justice. By referring clients to both non-health-related and health-related services, these programmes help to advance clients’ rights to justice, health and other rights, with implications also for addressing the underlying social and economic determinants of health [12].

Taken together, the documented experiences point to the importance of understanding the right to justice and the provision of legal aid as multidimensional concepts that involve numerous rights and encompass important opportunities for intervention outside of the formal judicial system.

At the same time, systemic problems plague this sort of work. Most notably, in Kenya (as in much of the world), the criminal justice system must be strengthened so that perpetrators can be prosecuted more effectively and the legal protection of rights appropriately enforced [13].

The research team encountered multiple challenges in carrying out the evaluation. Our quantitative indicators sought to draw on information that often turned out to be inaccessible or missing from programme records, and qualitative data could supplement this information only to a certain extent. One of the largest information gaps resulted from a lack of available records for all but 18 individual legal cases handled by CHAK.

Control group recruitment proved unexpectedly difficult, ultimately reducing our ability to make meaningful comparisons with intervention groups.

The evaluation was also challenged by differences in how human rights terms and concepts are understood by different people. Some concepts and principles, such as “empowerment,” do not translate directly from English into Swahili, and could only be expressed with synonyms. Issues such as these required the research team to make adjustments during the data collection process and to interpret findings in ways that acknowledged differences in how human rights are understood amongst different constituencies.

Evaluation findings provided anecdotal evidence that legal integration programmes can increase access to and utilization of health services. It was, however, beyond the scope of this evaluation to address what might be considered the ultimate question regarding such programmes: whether they are also associated with measurable improvements in health outcomes. Furthermore, while this evaluation makes a compelling case for legal integration programmes as a broadly effective health and human rights intervention, there are inherent limitations to extrapolating these findings to settings with different social and cultural norms or different legal systems.

Evaluation findings overall suggest several strategies for improving future legal empowerment efforts. Optimizing how such programmes make use of referrals should be a priority. Also, better linkages are needed with health facilities where programmes are located. Evaluation data indicated that social work departments and community health workers functioning under the aegis of larger health facilities often served as first points of contact for people who had experienced rights violations. Concern exists that these first points of contact might be missing opportunities to refer clients to legal empowerment programmes.

The cultivation of networks and watchdog groups that involve duty bearers such as the police in their activities stands out as a promising strategy for improving accountability, especially in settings where real and perceived corruption and indifference to rights-related claims deter people from pursuing justice. Community chiefs potentially have a great deal to contribute to the effectiveness of informal conflict resolution, as their role places them at the intersection of formal and informal structures of power: they are simultaneously government officers and perceived upholders of community traditions and standards [14].

The results of this evaluation also have implications for the future monitoring and evaluation of legal empowerment programmes. Findings point to the need for additional rigorous evaluations to inform the scale-up of such interventions. Programmes situated within larger health facilities may be able to capture client and case outcome information more effectively if they build their efforts onto monitoring structures already in place at the larger facilities. The feasibility of plausibility evaluations and qualitative methods for evaluating small-scale programmes should be further explored. An important next step will be to develop measures to determine associations between legal empowerment activities and health outcomes, including those related to treatment adherence and other health-seeking behaviours.

## Conclusions

While the right to justice is widely recognized as a core human right, its health-related implications are only now beginning to be systematically addressed. Integrating legal services with health services does more than facilitate access to justice – an important end in itself. By providing recipients of health services with greater access to justice, legal empowerment programmes can combat a wide range of human rights violations that undermine individual and public health. These programmes have the potential to provide marginalized communities with legal and human rights knowledge, as well as with guidance from legal professionals. Such programmes therefore have the potential to promote accountability for human rights violations, to reduce stigma and discrimination and ultimately to contribute to altering unjust structures and systems that hinder people from making informed and autonomous decisions about their health.

## References

[1] Csete J, Cohen J (2010). Health benefits of legal services for criminalized populations: the case of people who use drugs, sex workers and sexual and gender minorities. J Law Med Ethics.

[2] Sandel M, Hansen M, Kahn R, Lawton E, Paul E, Parker V (2008). Medical legal partnerships: transforming health care. Lancet.

[3] Medical-legalpartnership.org [Internet] http://www.medical-legalpartnership.org/.

[4] UNAIDS (2012). Key programmes to reduce stigma and discrimination and increase access to justice in national HIV responses. Guidance note.

[5] The Global Fund to Fight AIDS, Tuberculosis and Malaria (2011). The global fund strategy 2012–2016: investing for impact.

[6] UNDP, UNAIDS, The Global Fund to Fight AIDS, Tuberculosis and Malaria (2010). Analysis of key human rights programmes in global fund-supported HIV programmes.

[7] Mahajan AP, Sayles JN, Patel VA, Remien RH, Sawires SR, Ortiz DJ (2008). Stigma in the HIV/AIDS epidemic: a review of the literature and recommendations for the way forward. AIDS.

[8] Carr D, Eckhaus T, Brady L, Watts C, Zimmerman C, Nyblade L (2010). Scaling up the response to HIV stigma and discrimination.

[9] United Nations (2003). The human rights-based approach to development cooperation: towards a common understanding among UN agencies (“Common understanding”).

[10] Kalla K, Cohen J (2007). Ensuring justice for vulnerable communities in Kenya: a review of HIV and AIDS-related legal services.

[11] United Nations Committee on Economic, Social and Cultural Rights (2000). General comment no. 14 on the right to the highest attainable standard of health E/C.12/2000/4.

[12] Knox A, Giovarelli R, Forman M, Shelton M (2011). Issue brief: land tenure, property rights, and HIV/AIDS: approaches for reducing infection and enhancing economic security.

[13] Buscaglia E, Dijk JV (2003). Controlling organized crime and corruption in the public sector. United Nations Office on Drugs and Crime, Forum on Crime and Society.

[14] Akudugu MA, Mahama ES (2011). Promoting community-based conflict management and resolution mechanisms in the Bawku traditional area of Ghana. Peace Res.

# Sub article: Prevalence and drivers of HIV stigma among health providers in urban India: implications for interventions

**DOI:** 10.7448/IAS.16.3.18717

**Published:** 2013-11-13

**Authors:** Maria L Ekstrand, Jayashree Ramakrishna, Shalini Bharat, Elsa Heylen

**Affiliations:** 1 Center for AIDS Prevention Studies, University of California, San Francisco, CA, USA; 2 St. John's Research Institute, Bengaluru, Karnataka, India; 3 School of Public Health, University of California, Berkeley, CA, USA; 4 Department of Health Education, National Institute of Mental Health and Neurosciences, Bengaluru, Karnataka, India; 5 Centre for Health and Social Sciences, School of Health Systems Studies, Tata Institute of Social Sciences, Mumbai, Maharastra, India

**Keywords:** HIV stigma, stigma drivers, healthcare workers, India

## Abstract

**Introduction:**

HIV stigma inflicts hardship and suffering on people living with HIV (PLHIV) and interferes with both prevention and treatment efforts. Health professionals are often named by PLHIV as an important source of stigma. This study was designed to examine rates and drivers of stigma and discrimination among doctors, nurses and ward staff in different urban healthcare settings in high HIV prevalence states in India.

**Methods:**

This cross-sectional study enrolled 305 doctors, 369 nurses and 346 ward staff in both governmental and non-governmental healthcare settings in Mumbai and Bengaluru, India. The approximately one-hour long interviews focused on knowledge related to HIV transmission, personal and professional experiences with PLHIV, instrumental and symbolic stigma, endorsement of coercive policies, and intent to discriminate in professional and personal situations that involve high and low risk of fluid exposure.

**Results:**

High levels of stigma were reported by all groups. This included a willingness to prohibit female PLHIV from having children (55 to 80%), endorsement of mandatory testing for female sex workers (94 to 97%) and surgery patients (90 to 99%), and stating that people who acquired HIV through sex or drugs “got what they deserved” (50 to 83%). In addition, 89% of doctors, 88% of nurses and 73% of ward staff stated that they would discriminate against PLHIV in professional situations that involved high likelihood of fluid exposure, and 57% doctors, 40% nurses and 71% ward staff stated that they would do so in low-risk situations as well. Significant and modifiable drivers of stigma and discrimination included having less frequent contact with PLHIV, and a greater number of transmission misconceptions, blame, instrumental and symbolic stigma. Participants in all three groups reported high rates of endorsement of coercive measures and intent to discriminate against PLHIV. Stigma and discrimination were associated with multiple modifiable drivers, which are consistent with previous research, and which need to be targeted in future interventions.

**Conclusions:**

Stigma reduction intervention programmes targeting healthcare providers in urban India need to address fear of transmission, improve universal precaution skills, and involve PLHIV at all stages of the intervention to reduce symbolic stigma and ensure that relevant patient interaction skills are taught.

## Introduction

Across cultures, HIV stigma has repeatedly been shown to inflict hardship and suffering on people living with HIV (PLHIV) [1], as well as to interfere with their decisions to seek HIV counselling and testing [2, 3], prevention of mother-to-child transmission (PMTCT) [4–8], and their willingness to disclose their infection to their children [9] or partners [10–13], which can in turn increase the likelihood of sexual risk taking. HIV stigma has also been found to be a barrier to participation in vaccine research [14] and to deter infected individuals from seeking timely medical treatment [15–17].

These findings have been reported in both resource-rich and resource-constrained settings. Even when treatment is sought, stigma fears can prevent individuals from following their medical regimen, which can lead to virologic failure and the development and transmission of a drug-resistant virus [18–21]. PLHIV in Senegal and Indonesia have reported avoiding or delaying treatment seeking for STI/HIV infections, out of fear of public humiliation and fear of discrimination by healthcare workers [22, 23]. Similarly, HIV stigma in Botswana, South Africa, Jamaica and India has been associated with delays in testing and treatment services, sometimes resulting in presentation beyond the point of optimal drug intervention [24, 25].

Unfortunately, health professionals are often named as one of the most important sources of stigma for PLHIV. In sub-Saharan Africa, studies have documented discriminatory practices, including patient neglect, provision of differential treatment based on HIV status, denial of care, breach of confidentiality, isolation and verbal abuse by healthcare staff [26–28]. High rates of refusal of care have also been reported among nurses in Jordan [29] and stigma and discrimination have been documented in some healthcare settings in India also [15, 30–37].

In the general population of healthcare seeking individuals, behavioural manifestations of HIV stigma appear to be driven by both fear of casual transmission (*instrumental stigma*) and pre-existing prejudice towards vulnerable groups (*symbolic stigma*) [38]. No such data are available for healthcare providers. Studies of caregivers in other resource-constrained settings suggest that unwillingness to care for PLHIV is associated with negative views, high caregiver burden, low knowledge levels and education, and having a higher income [39, 40]. Understanding the specific drivers of stigma and its effects on behaviour in each setting is vital to the development of effective stigma reduction interventions for a given population [41]. The parent study was designed to meet this need by examining levels of stigma and discrimination as well as their potential drivers among healthcare providers, patients and the general outpatient population in two large urban settings in India. Our previous papers report on stigma among PLHIV [13, 42, 43] and the general patient population [38]. This article analyzes data from the healthcare providers and is the only one to date in India, which directly examines stigma domains and their drivers in these three healthcare provider groups, allowing us to conduct inter-staff comparisons. Another unique strength of this study is that it is the only one that includes all types of hospitals (including charity, trust, non-profit, for profit, and public) available in India. It is also the only study that includes data from two high prevalence areas in India, which allows for some generalization of results.

## Methods

### Participants

The participants for this cross-sectional study were recruited in 2009 from different types of healthcare settings in Bengaluru and Mumbai, two large Indian cities located in the “HIV high prevalence” states, Maharashtra and Karnataka [44]. At the time of the study, this label was given by the Indian National AIDS Control Organization to any state reporting >5% HIV prevalence in at least one key population or >1% in antenatal clinic settings. Field sites included medical colleges, government hospitals or private facilities, both for-profit and not-for-profit. Participants’ professional experience with HIV patients ranged from none to extensive. To meet the inclusion criteria, potential participants had to have worked as a doctor, nurse or ward staff in the selected hospitals/clinics for at least six months; have direct patient contact; be at least 18 years of age; able to speak one of the study languages; and able to give informed consent. The term “ward staff” included anyone who worked on the ward at a lower level than a nurse and who had substantial patient contact (including washing, transporting, changing linens). Most ward staff have minimal education or training and typically assist nurses or doctors with medical interventions. They are also a source of information and serve as confidants to patients. Final numbers recruited in Bengaluru were 149 doctors, 195 nurses and 176 ward staff; for Mumbai the numbers were 156, 174 and 170, respectively.

### Procedures

For each institution, we initially approached the Hospital Superintendents or Medical Directors for permission and subsequently contacted the Department heads for assistance in recruiting nurses and ward staff. Doctors preferred that we contact them directly to set up individual appointments. Following recruitment, potential participants were administered informed consent by study staff and following consent, an interview was scheduled. Interviews were conducted face to face in the participant's preferred language (Marathi, Hindi or English in Mumbai; Kannada, Tamil or English in Bengaluru) by trained study staff in a private space at the work site, and lasted approximately one hour. Participants received an in-kind gift worth 250 rupees (about 5 USD) following their interview, consisting of packets of fancy nuts and dried fruits in Bengaluru and a shopping bag in Mumbai.

Study procedures were approved by the Institutional Review Boards of the University of California in San Francisco, the National Institute of Mental Health and Neurosciences in Bengaluru, the Tata Institute of Social Sciences in Mumbai and received clearance from the Indian government.

### Measures

The survey measures used in this study were based on research conducted by Herek [45–49] as well as on the theoretical model subsequently developed by our team [13]. Stigma scales and drivers found to be significantly associated with mental health outcomes and delay of care seeking in our previous research with PLHIV [13, 42] and with the general outpatient population [38] were included in these analyses.

#### Demographic information

All participants were asked about their gender, age, marital status, religion, education and HIV training.

#### Potential drivers of stigma

##### Contact with PLHIV

Participants indicated the frequency of professional contact with PLHIV (0=never to 4=daily), and whether they personally had ever known any PLHIV (0=no; 1=yes).

##### Transmission misconceptions

Four items described forms of casual social contact through which HIV cannot be transmitted. For each item, participants indicated whether they thought HIV could be transmitted this way. The number of incorrect responses was summed. A higher score reflects more misconceptions.

##### Transmission knowledge

Participants were also asked if they thought HIV could be transmitted by direct exposure to several kinds of bodily fluids, or by activities such as unprotected sex with PLHIV. The number of correct answers to 15 such items was calculated, with higher scores reflecting better knowledge.

##### Instrumental stigma

Two individual items measured how worried participants were (0=not at all to 3=very worried) about getting HIV-infected (i) at work and (ii) outside of work.

##### Negative feelings towards PLHIV

Participants reported their feelings towards PLHIV on a scale from 0 (very negative) to 100 (very positive). To control for individual tendencies to assign high or low scores in general, we subtracted the rating for PLHIV from a similar rating of feelings towards men or women in general, so that higher anchored scores reflect more negative feelings towards PLHIV.

##### Blame

Participants indicated their agreement with the statement “People who got HIV/AIDS through sex or drug use have gotten what they deserve,” on a scale from 0 (strongly disagree) to 4 (strongly agree).

##### Symbolic stigma

Six items assessed how much participants’ moral/religious beliefs and feelings towards key populations influenced their opinions about HIV (0=not at all to 4=a great deal). An overall score was computed as the mean of all items. Higher scores express greater stigma. This scale had excellent reliability, with a Cronbach's *α* of 0.93 for doctors and 0.81 for both nurses and ward staff.

##### Perceived community stigma norms

Ten items assessed participants’ perceptions of the prevalence of HIV-stigmatizing attitudes in their community on a five-point scale [13]. Answers were averaged into one score, with higher numbers indicating more perceived community stigma. Cronbach's *α* ranged from 0.85 for doctors to 0.82 for nurses.

### Stigma manifestations

#### Intent to discriminate against PLHIV in professional situations

Participants were presented with two hypothetical work situations involving care for an HIV-positive patient. One situation posed virtually no risk of contact with bodily fluids. The second situation posed a greater risk of such contact. Response options were dichotomized as stigmatizing (refusing or performing the task only with unnecessary precautions) versus non-stigmatizing (performing the task as they would with any other patient).

#### Intent to discriminate against PLHIV in non-professional contexts

This was assessed by two hypothetical situations: (1) having a child who attends a school with an HIV-positive student and (2) getting medical care at a clinic that treated PLHIV. Leaving the school/clinic or avoiding contact/demanding special precautions was scored as stigmatizing. In addition, participants expressed their agreement (0=strongly disagree to 4=strongly agree) with seven statements about avoiding social or personal contact with PLHIV. Stigmatizing responses were summed over the nine items, with higher scores indicating greater intent to discriminate.

#### Endorsement of coercive policies

Participants indicated their agreement (0=strongly disagree to 4=strongly agree) with 11 statements related to the rights of PLHIV to have a family, education, employment, and health care; the right to choose to disclose HIV status; and mandatory HIV testing. Items were dichotomized, and stigmatizing responses (strongly/somewhat agree) were summed. Higher scores reflect greater endorsement of coercive policies.

### Data analysis

Frequencies and summary statistics were used to describe participants’ responses in the three groups. Differences between the three healthcare worker types in categorical outcomes were assessed via Chi-square tests, and in continuous outcomes via analysis of variance, with Bonferroni post-hoc pairwise comparisons in case of a significant *F*-value.

Separate multiple regressions were performed for each type of healthcare worker, using endorsement of coercive policies, and intent to discriminate in personal and professional contexts as separate outcomes. Site (Bengaluru vs. Mumbai) was controlled for in all models. All predictors that were associated bivariately with an outcome at *p<*0.25 [50] were initially included in the model. In subsequent models, the variable with the largest *p*-value was removed until all remaining variables were significant at *p<*0.10. For endorsement of coercive policies and intent to discriminate in personal context, linear regressions were performed. The two items for intent to discriminate at work were modelled via separate logistic regressions. Model assumptions regarding homoscedasticity, multicollinearity and influential outliers were adequately met. The logistic regressions were performed using SAS 9.2., and all other analyses were performed using SPSS 18.0.2.

## Results

### Demographic characteristics

As can be seen in Table 1, approximately half of the doctors (46%) and ward staff (51%), and almost all of the nurses (98%) were female and most were married. The vast majority of doctors (86%) and ward staff (78%) were identified as Hindu, while 59% of the nurses reported being Hindu and 36% identified as Christians, which is common in Indian hospitals. The mean age was slightly higher among ward staff: 39, compared to 35 for nurses and 34 for doctors. Education level among ward staff varied from less than four years (32%) to more than 10 years (8%) of schooling, with 45% having at least some secondary education. By definition, education was more uniform among doctors and nurses. Median household income was Rs. 40,000 (about $735) per month for doctors, Rs. 15,000 ($276) for nurses and Rs. 6000 ($110) for ward staff.

**Table 1:** Demographic characteristics

	Doctors (*n=*305)		Nurses (*n=*369)		Ward staff (*n=*346)	

	%	*n*	%	*n*	%	*n*
Site						
Bengaluru	49	149	53	195	51	176
Mumbai	51	156	47	174	49	170
Male	54	165	2	6	49	170
Religion						
Hindu	86	262	59	219	78	269
Muslim	5	16	1	5	1	5
Christian	2	6	36	132	6	21
Buddhist	3	10	3	12	15	51
Others	4	11	0	1	0	0
Marital status						
Currently married	58	177	68	251	77	267
Never married	41	125	29	105	8	29
Previously married	1	3	3	12	15	50
Education						
≤4 years					32	110
5–7 years					23	80
8–10			0	1	37	129
>10 years	100	305	100	368	8	27
Age: mean (SD)	33.7	(9.9)	34.9	(10.3)	39.4	(9.6)
Monthly household income in rupees: median (range)	40,000	(4000–900,000)	15,000	(2700–100,000)	6000	(400–50,000)

### HIV-related knowledge and experience

As can be seen in Table 2, approximately 70% of doctors and nurses indicated that they had received some form of HIV training, compared to 44% of ward staff (*p<*0.001). Despite their higher levels of HIV education, doctors and nurses did not score significantly higher on transmission knowledge than ward staff (*p=*0.18). The mean scores on the transmission knowledge index ranged from 11.4 out of 15 (ward staff) to 11.7 (doctors). However, the groups did differ in their mean number of casual transmission misconceptions, with the highest number occurring among ward staff (mean=0.8 out of 4), lower among nurses (mean=0.6), and lowest among doctors (mean=0.4). Female ward staff reported less professional contact with PLHIV than their male colleagues (mean=2.1 vs. 2.5, respectively, *p<*0.05) and were, on average, less knowledgeable about HIV transmission (mean knowledge score=11.1 vs. 11.8; mean misconceptions=1.0 vs. 0.6 for females and males, respectively, *p<*0.001). Similarly, female doctors had slightly more misconceptions than male doctors (females: mean=0.5 vs. males: mean=0.3, *p<*0.05). There were no other gender-related differences reported.

**Table 2:** Frequencies of reported stigma and other key model variables

	Doctors (*n=*305)		Nurses (*n=*369)		Ward staff (*n=*346)		
		
	%	*n*	%	*n*	%	*n*	*χ* ^2^
Received HIV training	73	223	71	263	44	152	77.73***
Professional contact w/PLHIV							
Never	2	6	4	13	9	32	23.02***
Less than weekly	50	152	51	187	49	170	
Weekly	16	48	13	49	11	37	
Daily	32	95	32	119	31	107	
Know PLHIV personally	26	78	27	99	28	98	0.63
Instrumental stigma^a^							
Worried about infection *at work*	78	237	72	264	51	175	60.55***
Worried about infection *outside of work*	17	52	26	96	27	94	10.09**
Blame^b^	50	153	70	259	83	284	79.72***
	
	Mean	SD	Mean	SD	Mean	SD	*F* d
	
Negative feelings towards PLHIV, anchored (−100 to 100)^c^	4_A_	26	11_B_	31	13_B_	39	7.22***
Perceived community stigma norms (0–3)	2.2_A_	0.6	2.3_A_	0.5	2.5_B_	0.5	20.54***
Symbolic stigma score (0–4)	1.7_A_	1.4	2.3_B_	1.1	2.2_B_	1.1	20.27***
Transmission misconceptions (0–4)	0.35_A_	0.76	0.56_B_	0.80	0.78_C_	1.08	18.10***
Transmission knowledge: items correct (0–15)	11.7_A_	1.6	11.5_A_	1.5	11.4_A_	1.8	1.73

a Proportion of participants answering “a little bit” to “very” worried.

b Proportion of participants who (strongly) agreed with the statement “People who got HIV from sex/drugs got what they deserved.”

c Anchored: PLHIV rating subtracted from own-gender rating, so scores<0 correspond to positive feelings, and scores>0 to negative feelings towards PLHIV.

d Means with different subscripts differ significantly (*p<*0.05) from each other (Bonferroni post-hoc pairwise comparisons).

† *p<*0.10

* *p<*0.05

** *p<*0.01

*** *p<*0.001.

More than 90% of all participants reported that they had experience caring for PLHIV, with about half in each group stating that they had at least weekly contact with HIV-positive patients. Just over a quarter of the participants in each group indicated that they had known a PLHIV personally. Nurses showed similar levels of worry about HIV infection at work as doctors, with about three quarters in both groups expressing such worries, compared to 51% of ward staff (*p<*0.001). Outside of work, more nurses (26%) and ward attendants (27%) reported worrying about HIV infection than doctors (17%, *p*<0.01).

### Attitudes towards PLHIV

Participants’ attitudes towards PLHIV, compared to their feelings towards men and women in general, differed significantly between the healthcare worker groups. Overall, doctors held the least negative feelings and ward staff the most negative, with the mean level of negative feelings towards PLHIV being 4 out of 100 (SD=26) for doctors, 11 (SD=31) for nurses and 13 (SD=39) for ward staff (*p<*0.001). A high proportion of participants in all three healthcare worker types agreed with the statement that people who acquired HIV through sex or drugs, “got what they deserved” – ranging from 50% of doctors, to 70% of nurses and 83% of ward attendants (*p*<0.001). The mean scores on the symbolic stigma scale were significantly lower for doctors (mean 1.7/4.0) than for nurses (2.3) and ward staff (2.2). Similarly, HIV-stigmatizing community norms were perceived to be higher by ward staff (mean=2.5/4.0) than by nurses (mean=2.3) and doctors (mean=2.2, *p<*0.001). There were significant gender differences with respect to feelings towards PLHIV among both doctors and ward staff. Female doctors reported significantly more negative feelings towards PLHIV (females: mean=8 vs. males: mean=0, *p<*0.01). Similarly, female ward staff held significantly more negative feelings towards PLHIV than their male colleagues (mean=19 vs. 8, respectively, *p<*0.05). In addition, female ward staff scored higher on perceived stigmatizing community norms (mean=2.6 vs. 2.4, respectively, *p<*0.001) and symbolic stigma (mean=2.3 vs. 2.1, respectively, *p<*0.05) than male ward staff. There were no other significant gender differences in any healthcare provider group with respect to attitudes towards PLHIV.

### Endorsement of coercive policies regarding PLHIV

Ward staff endorsed a mean of 6.7 out of 11 coercive policies, nurses endorsed on average 6.1 and doctors 4.8 (*p<*0.001). Mandatory testing for different groups was endorsed by large majorities of each group (See Table 3). Nearly all (94% of doctors and 97% of nurses and ward staff) supported mandatory testing for female sex workers (FSW), as well as for surgery patients (90% of doctors to 99% of nurses, *p<*0.001). Mandatory testing for surgery personnel was also endorsed by a majority, ranging from 73% of doctors, to 83% of nurses and to 88% of ward staff (*p<*0.001). Significantly more doctors (13%) than nurses and ward staff (both 5%, *p<*0.001) were in agreement with the statement that “health care workers should have the right to refuse to treat PLHIV.” The groups differed more widely in their endorsement of restricting PLHIV's rights to marry and have children. Slightly over half of the doctors did not think that HIV-positive women should be allowed to have children, compared to more than three quarters of both nurses and ward staff (*p<*0.001). Forty-one percent of doctors agreed that HIV-positive men should be denied the right to marry, as did 77% of nurses and 88% of ward staff (*p<*0.001). Similar proportions held the same opinion regarding HIV-positive women and marriage (37, 73 and 86%, respectively, *p<*0.001). There were no gender differences with respect to endorsement of coercive policies among any healthcare provider group.

**Table 3:** Endorsement of coercive policies and avoidance intentions towards PLHIV

	Doctors (*n=*305)		Nurses (*n=*369)		Ward staff (*n=*346)		
		
Individual items	%	*n*	%	*n*	%	*n*	*χ* ^2^
Coercive policies							
Mandatory testing for FSW	94	287	97	358	97	337	5.38†
Mandatory testing for surgery patients	90	274	99	366	96	332	29.85***
Mandatory testing for surgery staff	73	223	83	305	88	302	22.27***
HIV-positive women banned from having children	55	168	76	279	80	275	53.98***
HIV-positive men should not be allowed to marry	41	124	77	283	88	306	186.16***
HIV-positive women not be allowed to marry	37	112	73	269	86	296	182.98***
HCW should not have to treat PLHIV	13	39	5	19	5	17	18.87***
Intent to discriminate: professional							
High likelihood of contact w/bodily fluids^a^	89	272	88	324	73	252	
Low likelihood of contact w/bodily fluids^b^	57	174	40	146	71	243	
Intent to discriminate: personal							
Change clinic or demand extra precautions if PLHIV were treated where you get care:	59	179	61	224	50	173	9.75**
Change school or avoid HIV-positive child if HIV-infected child in your child's school:	15	46	22	82	32	112	26.58***
Would not eat from plate used by PLHIV	42	128	53	193	56	195	13.62**
PLHIV should be treated in separate clinics	34	103	56	205	61	212	54.10***
Not comfortable feeding PLHIV by hand	33	98	27	101	21	72	11.04**
Not seek services from HIV-positive HCW	23	71	31	114	36	124	12.21**

a High likelihood of contact w/bodily fluids: doctors: examine open wound; nurses: draw blood; ward staff: change blood-stained linens of PLHIV; no between-group comparisons done due to different items.

b Low likelihood of contact w/bodily fluids: doctors: routine physical exam; nurses: dispense medication; ward staff: bathe PLHIV; no between-group comparisons done due to different items.

† *p<*0.10

* *p<*0.05

** *p<*0.01

*** *p<*0.001.

### Intent to discriminate

A large majority of participants responded that they would either refuse to perform, avoid physical contact or use more than standard precautions if they were asked to treat an HIV-positive patient (see Table 3). This included examining an open wound (89% of doctors), drawing blood (88% of nurses) or changing blood-stained linens (73% of ward staff). When asked about professional behaviours with a low risk of fluid contact, 57% of doctors stated that they would either refuse or take additional precautions before performing a routine physical examination. Similar responses were given by 40% of nurses before dispensing medication and 71% of ward staff before bathing a PLHIV. More than half of the doctors and ward staff and 39% of the nurses reported discriminatory intent in both situations. Only 10 to 19% of participants reported no intent to discriminate in any professional situation.

At least half of the participants in all subsamples said they would stop attending, or demand extra precautions if they were patients in clinics that served PLHIV. This proportion was higher among doctors (59%) and nurses (61%) than among ward staff (50%, *p<*0.01). But more ward staff (61%) and nurses (56%) than doctors (34%) agreed with the statement that PLHIV should be treated in separate clinics (*p<*0.001), and stated that they would not seek services from an HIV-positive healthcare provider (36, 31 and 23%, respectively; *p<*0.01). Gender differences were found only among ward staff participants, with 77% of male ward staff expressing intent to discriminate if they had to bathe an HIV-positive patient, vs. 65% of female ward staff (*p=*0.01). When asked what they would do if an HIV-infected child attended their child's school, somewhat fewer participants – 15% of doctors, 22% of nurses, and 32% of ward staff (*p<*0.001) – showed discriminatory intent. In line with results regarding misconceptions, about half of the participants stated that they would not eat from a plate used by a PLHIV and about a quarter would not feel comfortable feeding a PLHIV by hand. The former was most common among ward staff, the latter among doctors, with the proportion of nurses in between for both items (both *p<*0.01). On average, doctors endorsed fewer personal discrimination intentions (mean=2.1) than nurses (mean=2.7) and ward staff (mean=2.9, *p<*0.001).

### Drivers of stigma

Results from the bivariate and final multivariate regression models are presented in Tables 4–6. Table 4 shows that doctors with greater instrumental stigma at work (*β*=0.24, *p<*0.001) and who did not know a PLHIV personally (*β*=0.13, *p=*0.033) reported higher endorsement of coercive policies than did doctors with lower instrumental stigma and those with a personal acquaintance with PLHIV. Instrumental stigma at work was also significantly related to higher intent to discriminate in personal situations (*β*=0.19, *p=*0.001), as were higher levels of negative feelings towards PLHIV (*β*=0.13, *p=*0.019), blame (*β*=0.11, *p=*0.044), transmission misconceptions (*β*=0.36, *p<*0.001), perceived stigmatizing community norms (*β*=0.11, *p=*0.038), and less frequent professional contact with PLHIV (*β*=0.11, *p=*0.047). Those with less frequent professional contact also had higher odds of showing discriminatory behaviour while performing a routine medical examination of a PLHIV (AOR=1.35; 95% CI=1.08–1.70) or dressing an open wound of a PLHIV (AOR=1.94; 95% CI=1.32–2.98). More transmission misconceptions was also associated with higher odds of discrimination during a routine examination (AOR=1.51; 95% CI=1.03–2.32). Gender was not associated with drivers of stigma or intent to discriminate among doctors.

**Table 4:** Bivariate and multivariate associations with outcomes, for doctors

	Bivariate Pearson *r*	Multivariate^a^	

		*β*	sig.
*Outcome: endorsement of coercive policies*		(*n=*271, *R* ^2^=0.11)	
Younger age	0.09^‡^		
Higher income (log-transformed)	−0.12^†^		
More negative feelings towards PLHIV	0.12*		
More blame	0.17**		
More work-related instrumental stigma	0.26***	0.241	.000
More non-work instrumental stigma	0.07^‡^		
More transmission misconceptions (4 items)	0.13*	0.108	.071
Lower transmission knowledge (15 items)	0.13		
Less frequent professional contact w/PLHIV	0.14*		
Not knowing any PLHIV personally	0.13*	0.125	.033
More symbolic stigma	0.18**	0.120	.077
*Outcome: intent to discriminate, personal life*		(*n=*265; *R* ^2^=0.32)	
Younger age	0.08^‡^		
Higher income (log-transformed)	−0.12^†^		
More negative feelings towards PLHIV	0.22***	0.133	0.019
More blame	0.24***	0.110	0.044
More work-related instrumental stigma	0.27***	0.188	0.001
More transmission misconceptions (4 items)	0.40***	0.364	0.000
Lower transmission knowledge (15 items)	0.16**		
Less frequent professional contact w/PLHIV	0.17**	0.110	0.047
Not knowing any PLHIV personally	0.13*		
More symbolic stigma	0.25***	0.121	0.056
More stigmatizing perceived community norms	0.11*	0.113	0.038
	Pearson *r*	AOR	95% CI
	
*Outcome: intent to discriminate, professional: routine exam*		(*n=*268)	
Non-Hindu religion	0.12*		
Unmarried	0.09^‡^		
Younger age	0.17**		
Higher income (log-transformed)	−0.11^†^		
More negative feelings towards PLHIV	0.17**		
More work-related instrumental stigma	0.16**	1.28^†^	(0.96–1.71)
More non-work instrumental stigma	0.08^‡^		
More transmission misconceptions (4 items)	0.12*	1.51*	(1.03–2.32)
Lower transmission knowledge (15 items)	0.10^†^		
Less frequent professional contact w/PLHIV	0.11^†^	1.35*	(1.08–1.70)
More symbolic stigma	−0.14*		
More stigmatizing perceived community norms	−0.12*	0.64^†^	(0.37–1.07)
*Outcome: intent to discriminate, professional: open wound*		(*n=*270)	
Non-Hindu religion	0.08^‡^		
Higher income (log-transformed)	−0.07^‡^		
More negative feelings towards PLHIV	0.12*		
More blame	0.15*		
More work-related instrumental stigma	0.18**		
Lower transmission knowledge (15 items)	0.14*		
Less frequent professional contact w/PLHIV	0.17**	1.94**	(1.32–2.98)
Not knowing any PLHIV personally	0.14*	2.27^†^	(0.93–5.38)
More stigmatizing perceived community norms	−0.08^‡^		

Note: all models adjusted for site.

β standardized regression coefficient; AOR, adjusted odds ratio; CI, confidence interval.

a Multivariate regression: final model, obtained via backward elimination starting from all variables bivariately associated at *p<*0.25, until all *p<*0.10.

‡ *p<*0.25

† *p<*0.10

* *p<*0.05

** *p<*0.01

*** *p<*0.001.

The bivariate correlations and multivariate regression models in Table 5 show that nurses with lower levels of HIV transmission knowledge had significantly higher mean levels of endorsement of coercive policies (*β*=0.13, *p=*0.022), younger age (*β*=0.10, *p=*0.051), and higher mean levels of negative feelings towards PLHIV (*β*=0.10, *p=*0.050). Intent to discriminate in personal life was significantly related to nurses’ being non-Hindu (*β*=0.10, *p=*0.036), having higher levels of negative feelings towards PLHIV (*β*=0.12, *p=*0.013), of work and non-work instrumental stigma (work: *β*=0.23, *p*<0.001; non-work: *β*=0.11, *p=*0.029), and of perceived stigmatizing community norms (*β*=0.12, *p=*0.010). Finally, nurses with more misconceptions (*β*=0.20, *p<*0.001) and less transmission knowledge (*β*=0.19, *p<*0.001) also had significantly higher levels of discriminatory intent in personal situations. In both professional situations, nurses’ intent to discriminate was significantly related to higher levels of instrumental stigma at work (medication: AOR=1.37; 95% CI=1.08–1.73; draw blood: AOR=1.56; 95% CI=1.09–2.30), but the two outcomes varied in their relation to other correlates. Unmarried nurses (AOR=1.76; 95% CI=1.08–2.88) and those with lower household income (AOR=0.32; 95% CI=0.13–0.79) showed higher odds of discrimination than married nurses and nurses with higher income, respectively, when dispensing medication to PLHIV, while for the “draw blood” item, it was nurses with higher household income (AOR=4.33; 95% CI=1.21–16.12) and younger nurses (AOR=1.04; 95% CI=1.01–1.07) who were more likely to express discriminatory intent. Finally, more transmission misconceptions (AOR=1.69; 95% CI=1.27–2.26) and greater symbolic stigma (AOR=0.77; 95% CI=0.60–0.97) were associated with treating PLHIV differently when dispensing medication.

**Table 5:** Bivariate and multivariate associations with outcomes, for nurses

	Bivariate Pearson *r*	Multivariate^a^	

		*β*	sig.
*Outcome: endorsement of coercive policies*		(*n=*367; *R* ^2^=0.05)	
Younger age	0.08^‡^	0.101	.051
More negative feelings towards PLHIV	10^†^	0.104	.050
More work-related instrumental stigma	0.10^†^		
More transmission misconceptions (4 items)	0.12*	0.097	.074
Lower transmission knowledge (15 items)	0.15**	0.127	.022
*Outcome: intent to discriminate, personal life*		(*n=*362; *R* ^2^= 0.26)	
Non-Hindu religion	0.14**	0.101	.036
More negative feelings towards PLHIV	0.17***	0.119	.013
More work-related instrumental stigma	0.30***	0.234	.000
More non-work instrumental stigma	0.23***	0.112	.029
More transmission misconceptions (4 items)	0.32***	0.195	.000
Lower transmission knowledge (15 items)	0.29***	0.193	.000
Less frequent professional contact PLHIV	0.08^‡^		
More stigmatizing perceived community norms	0.11*	0.124	.010
	
	Pearson *r*	AOR	95% CI
	
*Outcome: intent to discriminate, professional: dispense medication*		(*n=*344)	
Non-Hindu religion	0.08^‡^		
Unmarried	0.16**	1.76*	(1.08–2.88)
Younger age	0.09^†^		
Higher income (log-transformed)	−0.14**	0.32*	(0.13–0.79)
More negative feelings towards PLHIV	0.09^†^		
More work-related instrumental stigma	0.15**	1.37**	(1.08–1.73)
More non-work instrumental stigma	0.10^†^		
More transmission misconceptions (4 items)	0.22***	1.69***	(1.27–2.26)
Lower transmission knowledge (15 items)	0.13*		
Less frequent professional contact PLHIV	0.11*		
More symbolic stigma	−0.09^†^	0.77*	(0.60–0.97)
*Outcome: intent to discriminate, professional: draw blood*		(*n=*359)	
Younger age	0.16**	1.04*	(1.01–1.07)
Higher income (log-transformed)	0.11*	4.33*	(1.21–16.12)
More negative feelings towards PLHIV	0.13*	1.01^†^	(1.00–1.03)
More work-related instrumental stigma	0.19***	1.56*	(1.09–2.30)
More transmission misconceptions (4 items)	0.07^†^		
Not knowing any PLHIV personally	−0.10^†^	0.47^†^	(0.18–1.07)

Note: all models adjusted for site.

*β*, standardized regression coefficient; AOR, adjusted odds ratio; CI, confidence interval.

a Multivariate regression: final model, obtained via backward elimination starting from all variables bivariately associated at *p*<0.25, until all *p*<0.10.

‡ *p<*0.25

† *p<*0.10

* *p<*0.05

** *p<*0.01

*** *p<*0.001.

The bivariate correlations and multivariate regression models for ward staff are shown in Table 6. Endorsement of coercive policies and intent to discriminate in personal situations were both significantly related to more negative feelings towards PLHIV (*β*=0.15, *p=*0.006; *β*=0.15, *p=*0.003, respectively), more blame (*β*=0.13, *p=*0.020; *β*=0.12, *p=*0.013), more misconceptions (*β*=0.13, *p=*0.014; *β*=0.34, *p<*0.001) and more symbolic stigma (*β*=0.14, *p=*0.014; *β*=0.14, *p=*0.006). In addition, intent to discriminate in personal situations also increased with younger age (*β*=0.17, *p<*0.001), decreasing frequency of professional contact with PLHIV (*β*=0.11, *p=*0.025), and increasing levels of instrumental stigma at work (*β*=0.22, *p<*0.001). Male ward staff were twice as likely (AOR=1.98; 95% CI=1.21–3.28) to discriminate when bathing patients than female ward staff. Males were also more likely than females (AOR=1.95; 95% CI=1.13–3.45) to discriminate when asked to change a PLHIV's blood-stained linens. Having more negative feelings towards PLHIV (AOR=1.10; 95% CI=1.00–1.02), greater work-related instrumental stigma (AOR=1.66; 95% CI=1.28–2.19) and less frequent professional contact with PLHIV (AOR=1.22; 95% CI=1.00–1.49) were also associated with higher odds of discrimination in this situation.

**Table 6:** Bivariate and multivariate associations with outcomes, for ward staff

	Bivariate Pearson *r*	Multivariate^a^	

		*β*	sig.
*Outcome: endorsement of coercive policies*		(*n=*318; *R* ^2^=0.14)	
Non-Hindu religion	0.12*		
More negative feelings towards PLHIV	0.21***	0.151	0.006
More blame	0.20***	0.126	0.020
More work-related instrumental stigma	0.22***	0.111	0.073
More non-work instrumental stigma	0.12*		
More transmission misconceptions (4 items)	0.20***	0.134	0.014
Less frequent professional contact w/PLHIV	0.15**		
More symbolic stigma	0.12*	0.143	0.014
*Outcome: intent to discriminate, personal life*		(*n=*314; *R* ^2^=0.37)	
Younger age	0.13*	0.169	0.000
More negative feelings towards PLHIV	0.24***	0.145	0.003
More blame	0.22***	0.120	0.013
More work-related instrumental stigma	0.30***	0.216	0.000
More non-work instrumental stigma	0.18***		
More transmission misconceptions (4 items)	0.42***	0.335	0.000
Lower transmission knowledge (15 items)	0.24***	0.091	0.072
Less frequent professional contact w/PLHIV	0.22***	0.107	0.025
More symbolic stigma	0.20***	0.140	0.006
More stigmatizing perceived community norms	0.14**		
	
	Pearson *r*	AOR	95% CI
	
*Outcome: intent to discriminate, professional: bathe PLHIV*		(*n=*336)	
Male gender	0.14*	1.98**	(1.21–3.28)
Unmarried	0.06^‡^		
Younger age	0.09^†^		
Not knowing any PLHIV personally	−0.08^‡^		
More stigmatizing perceived community norms	−0.08^‡^	1.59^†^	(0.97–2.62)
*Outcome: intent to discriminate, professional: change blood-stained linens*		(n=314)	
Male gender	0.06^‡^	1.95*	(1.13–3.45)
Younger age	0.06^‡^		
Lower education	−0.06^‡^		
More negative feelings towards PLHIV	0.14*	1.10**	(1.00–1.02)
More work-related instrumental stigma	0.15**	1.66***	(1.28–2.19)
More non-work instrumental stigma	0.09^†^		
More transmission misconceptions (4 items)	0.10^†^		
Less frequent professional contact w/PLHIV	0.10^†^	1.22*	(1.00–1.49)
Not knowing any PLHIV personally	−0.06^‡^	0.57^†^	(0.29–1.05)
More symbolic stigma	0.11*		
More stigmatizing perceived community norms	0.06^‡^		

Note: all models adjusted for site.

β standardized regression coefficient; AOR, adjusted odds ratio; CI, confidence interval.

a Multivariate regression: final model, obtained via backward elimination starting from all variables bivariately associated at *p<*0.25, until all *p<*0.10.

‡ *p<*0.25

† *p<*0.10

* *p<*0.05

** *p*<0.01

*** *p*<0.001.

## Discussion

The results reveal disturbingly high rates of stigma attitudes and intent to discriminate among doctors, nurses and ward staff in these urban healthcare settings. The rates are similar to those reported by outpatients in these settings as well as to results of studies conducted in other parts of the country [11–13, 30, 32–36, 38, 42] and may thus represent wider community norms. The almost universal endorsement of mandatory testing for FSW and surgery patients may be one of the reasons why testing is now routinely performed in Indian healthcare settings for surgery patients and pregnant women. Routine periodic testing of key populations is also currently done in some areas. Although doctors were less likely (37 to 55%) than nurses (73 to 76%) and ward staff (80 to 88%) to endorse the different coercive measures in relation to marriage and children, their rates were still surprisingly high. These endorsements are of particular concern since they involve the denial of basic human rights of PLHIV to enjoy marital status and parenthood, which are crucial aspects of Indian culture. These findings highlight the need for a rights-based approach to addressing stigma in future regional and national intervention programmes.

Participants also reported high rates of intent to treat HIV-positive patients differently from uninfected patients, both in situations that involved a risk of fluid exposure and in situations that are typically considered low risk. Since female ward staff reported more transmission misconceptions and a more negative view of PLHIV, the finding that male ward staff were more likely to report intent to discriminate may reflect their perception that they have more control over their job duties than their female counterparts. This needs to be explored further to determine how to best address this gender difference in a stigma reduction intervention. It was encouraging that physicians and nurses were significantly less likely to state that they intended to discriminate in low-risk situations; however, health care professionals who use universal precautions do not need to use double gloves or avoid HIV-infected patients in order to be safe. In addition to stigma, these high rates might also be indicative of lack of confidence in standard universal measures to prevent infection.

Intent to discriminate was only slightly less in non-professional situations. The majority of all groups stated that they did not want to be treated in the same clinics as PLHIV and more than half of the nurses and ward staff reported that they would be unwilling to eat from the same plate as an infected individual. This item was endorsed by 42% of the doctors also.

Although there are minor variations, the drivers of stigma and discrimination appear to be fairly consistent across the different groups. Transmission-related fears and misconceptions, as well as limited experience working with PLHIV, blame and negative feelings towards PLHIV seem to be driving both endorsement of coercive measures and intent to discriminate against PLHIV in personal and professional contexts, regardless of whether the latter situations actually involve risk of fluid exposure. This is consistent with findings from previous studies [30, 31, 33, 34], and our previous paper on stigma among outpatients in Mumbai and Bengaluru [38], suggesting that misconceptions are a consistent driver of HIV stigma in India. The findings from this study thus indicate that stigma reduction interventions need to target common misconceptions, even among highly educated and already trained healthcare providers. Since younger and less experienced nurses and ward staff were more likely to discriminate, there may also be a need to ensure that they are thoroughly trained in universal precautions until they are comfortable and confident in their ability to prevent transmission.

The fact that more experience with PLHIV was associated with lower rates of stigma and discrimination in all three groups suggests that interventions may be more effective if PLHIV are involved at all stages of intervention development and implementation to ensure sufficient and meaningful interactions. It might also be helpful to involve experienced healthcare providers, who have extensive experience treating PLHIV as role models for their junior colleagues to provide opportunities for observational learning, help change norms in the workplace and to increase the likelihood of intervention sustainability. Doctors treating PLHIV respectfully are also likely to make an impression and set a standard for both nurses and ward staff in their institutions, given the hierarchical nature of relationships in these settings.

Both female doctors and female ward staff reported a greater number of transmission misconceptions than did their male counterparts, in spite of their very different levels of education. This suggests that there may be differences in HIV-related education received by male and female students in Indian schools. It is thus important for future HIV prevention and stigma interventions to address basic transmission facts when targeting female participants, regardless of their level of education.

Similar to every study, ours has a number of limitations that need to be considered when interpreting its results. Since this study used a cross-sectional design, we are unable to draw conclusions about causality and can only state which variables are associated. Future research is needed to examine these relationships in a longitudinal fashion to clarify the nature of these associations. In addition, the generalizability of these findings is limited to the types of healthcare settings that collaborated with us in these two large urban areas. We made every effort to recruit healthcare providers from a wide range of clinics and hospitals, in order to be as representative as possible of healthcare settings that are accessible to patients of all socioeconomic backgrounds. However, our sample did not include healthcare providers in non-allopathic institutions. We are also limited by our reliance on self-reported measures, which may be subject to social desirability biases. Additional studies using behavioural observations are needed to provide data on enacted stigma in these settings.

## Conclusions

The high rates of stigma and discrimination among healthcare providers in these urban Indian healthcare settings appear to be driven primarily by negative feelings towards PLHIV, lack of experience as well as misconceptions and fear of casual transmission. Stigma reduction interventions are thus urgently needed to target transmission misconceptions and to increase interactions with PLHIV. Such programmes need to be designed and implemented in collaboration with PLHIV networks and use a rights-based and gender-sensitive approach. In order to be both effective and sustainable, interventions should ideally make use of professional role models and be integrated into existing training structures in hospital clinics and the curricula in nursing and medical schools.

## References

[1] Krishna V, Bhatti R, Chandra P, Juvva S (2005). Unheard voices: experiences of families living with HIV/AIDS in India*. Contemp Fam Ther.

[2] Ma W, Detels R, Feng Y, Wu Z, Shen L, Li Y (2007). Acceptance of and barriers to voluntary HIV counselling and testing among adults in Guizhou province, China. AIDS.

[3] Obermeyer CM, Osborn M (2007). The utilization of testing and counseling for HIV: a review of the social and behavioral evidence. Am J Public Health.

[4] Nguyen TA, Oosterhoff P, Pham YN, Hardon A, Wright P (2009). Health workers’ views on quality of prevention of mother-to-child transmission and postnatal care for HIV-infected women and their children. Hum Resour Health.

[5] Varga CA, Sherman GG, Jones SA (2006). HIV-disclosure in the context of vertical transmission: HIV-positive mothers in Johannesburg, South Africa. AIDS Care.

[6] Eide M, Myhre M, Lindbaek M, Sundby J, Arimi P, Thior I (2006). Social consequences of HIV-positive women's participation in prevention of mother-to-child transmission programmes. Patient Educ Couns.

[7] Bond V, Chase E, Aggleton P (2002). Stigma, HIV/AIDS and prevention of mother-to-child transmission in Zambia. Eval Program Plann.

[8] Nyblade L, Field ML (2000). Community involvement in prevention of mother-to-child transmission (PMTCT) initiative. Women, communities and the prevention of mother-to-child transmission of HIV: issues and findings from community research in Botswana and Zambia.

[9] Madiba S (2013). The impact of fear, secrecy, and stigma on parental disclosure of HIV status to children: a qualitative exploration with HIV positive parents attending an ART clinic in South Africa. Glob J Health Sci.

[10] Calin T, Green J, Hetherton J, Brook G (2007). Disclosure of HIV among black African men and women attending a London HIV clinic. AIDS Care.

[11] Chandra PS, Deepthivarma S, Manjula V (2003). Disclosure of HIV infection in south India: patterns, reasons and reactions. AIDS Care.

[12] Kumarasamy N, Safren SA, Raminani SR, Pickard R, James R, Krishnan AK (2005). Barriers and facilitators to antiretroviral medication adherence among patients with HIV in Chennai, India: a qualitative study. AIDS Patient Care STDS.

[13] Steward WT, Herek GM, Ramakrishna J, Bharat S, Chandy S, Wrubel J (2008). HIV-related stigma: adapting a theoretical framework for use in India. Soc Sci Med.

[14] Nyblade L, Singh S, Ashburn K, Brady L, Olenja J (2011). “Once I begin to participate, people will run away from me”: understanding stigma as a barrier to HIV vaccine research participation in Kenya. Vaccine.

[15] Bharat S, Aggleton P, Tyrer P (2001). India: HIV and AIDS-related discrimination, stigma and denial.

[16] Kinsler JJ, Wong MD, Sayles JN, Davis C, Cunningham WE (2007). The effect of perceived stigma from a health care provider on access to care among a low-income HIV-positive population. AIDS Patient Care STDS.

[17] Morrison SD, Banushi VH, Sarnquist C, Gashi VH, Osterberg L, Maldonado Y (2011). Barriers to care and current medical and social needs of HIV-positive patients in Albania. Cent Eur J Public Health.

[18] Paterson DL, Swindells S, Mohr J, Brester M, Vergis EN, Squier C (2000). Adherence to protease inhibitor therapy and outcomes in patients with HIV infection. Ann Intern Med.

[19] Bangsberg DR, Hecht FM, Charlebois ED, Zolopa AR, Holodniy M, Sheiner L (2000). Adherence to protease inhibitors, HIV-1 viral load, and development of drug resistance in an indigent population. AIDS.

[20] Arnsten JH, Demas PA, Farzadegan H, Grant RW, Gourevitch MN, Chang CJ (2001). Antiretroviral therapy adherence and viral suppression in HIV-infected drug users: comparison of self-report and electronic monitoring. Clin Infect Dis.

[21] Bangsberg DR, Perry S, Charlebois ED, Clark RA, Roberston M, Zolopa AR (2001). Non-adherence to highly active antiretroviral therapy predicts progression to AIDS. AIDS.

[22] Ford K, Wirawan DN, Sumantera GM, Sawitri AA, Stahre M (2004). Voluntary HIV testing, disclosure, and stigma among injection drug users in Bali, Indonesia. AIDS Educ Prev.

[23] Niang CI, Tapsoba P, Weiss E, Diagne M, Niang Y, Moreau AM (2003). It's raining stones’: stigma, violence and HIV vulnerability among men who have sex with men in Dakar, Senegal. Cult Health Sex.

[24] Wolfe WR, Weiser SD, Leiter K, Steward WT, Percy-De Korte F, Phaladze N (2008). The impact of universal access to antiretroviral therapy on HIV stigma in Botswana. Am J Public Health.

[25] White RC, Carr R (2005). Homosexuality and HIV/AIDS stigma in Jamaica. Cult Health Sex.

[26] Feyissa GT, Abebe L, Girma E, Woldie M (2012). Stigma and discrimination against people living with HIV by healthcare providers, Southwest Ethiopia. BMC Public Health.

[27] Banteyerga H, Kidanu A, Nyblade L, MacQuarrie K, Pande R (2004). Yicha-laliko! Exploring HIV and AIDS stigma and related discrimination in Ethiopia: causes, manifestations, consequences, and coping mechanisms.

[28] (2005). Working report measuring HIV stigma: results of a field test in Tanzania. http://www.icrw.org/files/publications/Working-Report-Measuring-HIV-Stigma-Results-of-a-Field-Test-in-Tanzania.pdf.

[29] Hassan ZM, Wahsheh MA (2011). Knowledge and attitudes of Jordanian nurses towards patients with HIV/AIDS: findings from a nationwide survey. Issues Ment Health Nurs.

[30] Vyas KJ, Patel GR, Shukla D, Mathews WC (2010). A comparison in HIV-associated stigma among healthcare workers in urban and rural Gujarat. SAHARA J.

[31] Mahendra VS, Gilborn L, Bharat S, Mudoi R, Gupta I, George B (2007). Understanding and measuring AIDS-related stigma in health care settings: a developing country perspective. SAHARA J.

[32] Bharat S (1998). Facing the HIV/AIDS challenge: a study on household and community responses. Health Millions.

[33] Kurien M, Thomas K, Ahuja RC, Patel A, Shyla PR, Wig N (2007). Screening for HIV infection by health professionals in India. Natl Med J India.

[34] Kermode M, Holmes W, Langkham B, Thomas MS, Gifford S (2005). HIV-related knowledge, attitudes and risk perception amongst nurses, doctors and other healthcare workers in rural India. Indian J Med Res.

[35] Kumar R, Mohan N, Seenu V, Kumar A, Nandi M, Sarma RK (2002). Knowledge, attitude and practices towards HIV among nurses in a tertiary care teaching hospital: two decades after the discovery. J Commun Dis.

[36] Bharat S (2011). A systematic review of HIV/AIDS-related stigma and discrimination in India: current understanding and future needs. SAHARA J.

[37] Mahendra VS, Gilborn L, George B, Samson L, Mudoi R, Jadav S (2006). Reducing AIDS-related stigma and discrimination in Indian hospitals.

[38] Ekstrand ML, Bharat S, Ramakrishna J, Heylen E (2012). Blame, symbolic stigma and HIV misconceptions are associated with support for coercive measures in urban India. AIDS Behav.

[39] Singh D, Chaudoir SR, Escobar MC, Kalichman S (2011). Stigma, burden, social support, and willingness to care among caregivers of PLWHA in home-based care in South Africa. AIDS Care.

[40] Messer LC, Pence BW, Whetten K, Whetten R, Thielman N, O'Donnell K (2010). Prevalence and predictors of HIV-related stigma among institutional- and community-based caregivers of orphans and vulnerable children living in five less-wealthy countries. BMC Public Health.

[41] Weiss MG, Ramakrishna J, Somma D (2006). Health-related stigma: rethinking concepts and interventions. Psychol Health Med.

[42] Steward WT, Bharat S, Ramakrishna J, Heylen E, Ekstrand ML (2013). Stigma is associated with delays in seeking care among HIV-infected people in India. J Int Assoc Provid AIDS Care.

[43] Steward WT, Chandy S, Singh G, Panicker ST, Osmand TA, Heylen E (2011). Depression is not an inevitable outcome of disclosure avoidance: HIV stigma and mental health in a cohort of HIV-infected individuals from Southern India. Psychol Health Med.

[44] National AIDS Control Organization http://naco.gov.in/upload/Surveillance/Reports%20&%20Publication/HIV%20Sentinel%20Surveillance%20India%20Country%20Report,%202008-09.pdf.

[45] Herek GM (2002). Thinking about AIDS and stigma: a psychologist's perspective. J Law Med Ethics.

[46] Herek GM (1997). AIDS and stigma. Am Behav Sci.

[47] Herek GM (2007). Confronting sexual stigma and prejudice: theory and practice. J Soc Issues.

[48] Herek GM, Capitanio JP (1993). Public reactions to AIDS in the United States: a second decade of stigma. Am J Public Health.

[49] Herek GM, Mitnick L, Burris S, Chesney M, Devine P, Fullilove MT (1998). Workshop report: AIDS and stigma: a conceptual framework and research agenda. AIDS Public Policy J.

[50] Hosmer D, Lemeshow S (2000). Applied logistic regression.

# Sub article: Assessment of HIV-related stigma in a US faith-based HIV education and testing intervention

**DOI:** 10.7448/IAS.16.3.18644

**Published:** 2013-11-13

**Authors:** Jannette Y Berkley-Patton, Erin Moore, Marcie Berman, Stephen D Simon, Carole Bowe Thompson, Thomas Schleicher, Starlyn M Hawes

**Affiliations:** 1 Department of Psychology, University of Missouri – Kansas City, Kansas City, MO, USA; 2 Department of Psychology, Stetson University, DeLand, FL, USA; 3 Department of Biomedical and Health Informatics, University of Missouri – Kansas City, Kansas City, MO, USA; 4 Department of Psychiatry and Behavioral Sciences, University of Washington School of Medicine, Seattle, WA, USA

**Keywords:** faith-based settings, faith organizations, HIV-related stigma, African American church, church members, community members, HIV intervention, HIV testing, people living with HIV

## Abstract

**Introduction:**

The African American church is a highly influential institution with the potential to greatly increase the reach of HIV prevention interventions and address HIV-related stigma in US African American communities. However, there are few studies on HIV-related stigma and African American church populations. This study explored HIV-related stigma among church and community members participating in an HIV education and testing intervention pilot study in African American churches, named Taking It to the Pews.

**Methods:**

Four African American churches located in Kansas City, MO and KS, were randomized to either intervention or comparison groups. Churches assigned to the intervention group received religiously tailored HIV education, testing and compassion messages/activities (e.g., sermons, brochures/church bulletins, testimonials) via the Taking It to the Pews HIV Tool Kit. Comparison churches received non-religiously tailored HIV information. HIV-related stigma was assessed with 543 church members and with community members served through church outreach services (e.g., food/clothing pantries, social services) in the four churches. Participants completed surveys at baseline, 6 months and 12 months to assess their HIV-related stigma beliefs, exposure to intervention components and satisfaction with the study.

**Results:**

At baseline, HIV-related stigma beliefs were similar across experimental groups and were quite low. Mean HIV-related stigma scores were not significantly different between experimental groups at 6 months (*p*=0.92) or at 12 months (*p*=0.70). However, mean HIV-related stigma scores within both groups showed decreasing trends at six months, which approached significance. Analysis of previously studied HIV-related stigma factors (e.g., age, gender, income, HIV knowledge, religiosity) did not yield changes in the null findings. Intervention group participants were highly exposed to several intervention components (sermons, HIV resource tables, posters, brochures/church bulletins). Overall, participants were highly satisfied with the intervention pilot study.

**Conclusions:**

African American churches may be well positioned to increase the reach of HIV prevention interventions to church and community members and could serve an important role in addressing HIV-related stigma in their church communities. Future research is needed on measuring HIV-related stigma beliefs and on testing intensive, scalable, religiously tailored HIV interventions to impact HIV-related stigma in African American churches.

## Introduction

HIV continues to disproportionately impact African American (AA) communities in the United States [1–5]. A primary barrier that impedes efforts to develop, implement and test HIV education, testing and linkage-to-care programmes is HIV-related stigma (hereafter referred to as “HIV stigma”) [6–11], which can hamper efforts to reduce the HIV burden in AA communities. Studies have shown that fear of HIV stigma has been associated with reduced rates of HIV testing and engagement in treatment among AAs [12, 13], and poor disease management and quality of life for AA people living with HIV (PLHIV) [12–15].

Traditionally, stigma has been defined as negative attitudes towards preventable or controllable illnesses with causes identified as undesirable/immoral behaviours (e.g., having sex outside of marriage) and associated with certain groups (e.g., men who have sex with men) who are blamed for their illness [16]. Goffman's early work on stigma suggested that negative attitudes towards undesirable behaviours arise from perceptions that “out-groups” exhibiting these unacceptable behaviours have violated a community's set of values or community norms [17]. Regarding HIV stigma, PLHIV may be prone to experience HIV stigma based on others’ perceptions of these behaviours, which can lead to perceived and actual disadvantage or discrimination. Parker and Aggelton [18] argue for the need to go beyond the individual focus on HIV stigma and to instead examine stigma as an evolving social process with consideration given to changing the structural and changing beliefs of stigma-generating groups through community mobilization efforts. However, important questions remain on how community mobilization in settings that traditionally may have promoted HIV stigmatizing beliefs can now be engaged in positively influencing their constituents to extend compassion and support for PLHIV, join PLHIV in speaking against HIV stigma, and ultimately encourage their community members to assist in advocacy efforts to eliminate injustices and discrimination against PLHIV.

The AA church is a long-standing, powerful institution with a tradition of mobilizing AA communities for social and political change and could play an important role in leading mobilization efforts to reduce HIV stigma beliefs among AAs. Nationwide studies indicate that most AAs in the United States attend church weekly [19–21] and believe that church leaders are highly influential [22, 23]. Also, most AA churches have similar characteristics, including similar modes of worship (e.g., sermons, testimonials) [23, 24], and community outreach ministries [23–25] that could facilitate the implementation of HIV prevention interventions to educate about HIV risks, promote HIV testing and impact HIV stigma in their church communities. Although the AA church has been criticized for its lack of involvement in the early years of the HIV epidemic [7], a shift in churches willing to address HIV and participate in HIV-related research studies seems to be emerging [24, 26–30]. Still, several of the controversial issues (e.g., homosexuality, premarital sex, multiple sex partners, drug use) associated with HIV stigma in AA communities are not discussed or are denounced in many AA churches [31–33]. Studies with AA faith leaders suggest they are interested in participating in church-based HIV prevention interventions [24, 31, 32], but also identify HIV stigma as a key barrier that can slow adoption of such interventions [33–35]. Recent studies have found that with religiously tailored HIV education and motivational supportive strategies, the AA church can serve as a potentially influential venue to address HIV and related stigma beliefs among their church/community members [24, 26–30].

Few studies have examined HIV stigma beliefs with AA church populations [35, 36]. Still, these studies with AA church populations found low levels of HIV stigma beliefs and related personal factors, such as age, education, religiosity and HIV knowledge [35, 36]. Yet, to our knowledge, no studies have examined HIV prevention intervention effects on HIV stigma in AA churches. The primary aim of this study was to pilot test feasibility of the Taking It to the Pews (TIPS) project (an HIV education and testing intervention in AA churches) and determine its effect size on HIV-testing rates to plan a future, clustered randomized trial. During this pilot study, we also assessed HIV stigma beliefs as an intervention outcome. The current study reports on HIV stigma beliefs assessed at baseline, 6 months and 12 months in the pilot study.

## Methods

### Contextual background

The TIPS project used a community-based participatory research (CBPR) approach to mobilize AA churches to address HIV prevention through education and testing [37]. AA church leaders chose this TIPS research focus with the aim of increasing HIV testing (instead of focusing on condom use as an HIV prevention strategy) and reducing HIV stigma. Church leaders along with church and community members (inclusive of PLHIV) participated in TIPS intervention development (including creation of the TIPS HIV Tool Kit), implementation and evaluation [24, 27, 38]. Using the CBPR approach, trained church leaders delivered religiously appropriate TIPS HIV Tool Kit materials/activities through multiple church outlets (community outreach ministries, church services, group ministries, peer-to-peer). Tool kit materials/activities (e.g., sermon guides, posters, resource tables, video/printed/in-person testimonials, church bulletin inserts, brochures) were designed to: (a) “fit” within existing church activities for ease in mobilizing AA churches, (b) provide HIV education and enhance compassion/respect for PLHIV and (c) engage pastors to promote HIV testing and stigma reduction [24]. Based on past AA church population studies [35, 36], HIV stigma was hypothesized to be related to age and religiosity; and inversely related to income, HIV knowledge and intervention exposure.

### Study design

Pastors of five AA churches were approached for recruitment; four agreed to have their churches participate in the study, and one declined due to commitments to other new projects. Churches were matched on size of membership and type of outreach services and were randomly assigned to intervention (*n*=2 churches) and comparison groups (*n*=2 churches). Intervention churches received one to two TIPS intervention religiously tailored materials/activities, and comparison churches received one to two non-religiously tailored HIV informational brochures/church bulletins, monthly. Participants were assessed at baseline, 6 months and 12 months.

### Setting and participants

The four participating churches were located in Kansas City, MO and KS, USA. Churches were recruited to participate based on four criteria: (a) minimum of 150 AA adult church members; (b) a willing pastor and two church members serving as church liaisons to assist in delivery of TIPS study activities; (c) outreach services (e.g., food/clothing programmes, recovery programmes) available to a minimum of 50 community members per month; and (d) never having hosted an HIV-related event. Participating churches were compensated $2500 for recruitment, retention, implementation of TIPS interventions and reporting data through an online system. Church liaisons were provided with $500 for their assistance in intervention delivery. Additionally, churches were supplied with technology support to assist in delivering the intervention (e.g., digital projector, telephone messaging system). Participating church members and community members (who regularly used church outreach services) were aged 18–64 and were consented to participate in the study, and received $10 for completing baseline, 6-month and 12-month surveys ($30 total). Surveys were administered to church members after their church services and to community members after church community outreach activities. The University of Missouri – Kansas City IRB approved the study.

### Intervention overview

Over a 12-month period, church liaisons delivered the TIPS HIV Tool Kit materials/activities through various church activities (e.g., community outreach, church services, ministry groups, interpersonal interactions). The original TIPS case study and all HIV Tool Kit materials/activities have been fully described elsewhere [24, 27] and are briefly described here.

#### Community outreach level activities

Church liaisons delivered printed materials (e.g., brochures, printed testimonials) to community members through church outreach services (e.g., food/clothing pantry, social services). At opportune times, pastors delivered brief messages about HIV topics and promoted HIV testing and PLHIV compassion with community members where/when appropriate (e.g., parents meetings, before prayer at a free meal event). In collaboration with local health agencies, liaisons coordinated three HIV-testing events for church/community members. At least one of these testing events was scheduled during a highly attended community outreach activity (e.g., food/clothing programme, social services).

#### Church-wide service level

Pastors delivered sermons on HIV topics, promoted HIV testing and encouraged the reduction of HIV risks and stigma. During HIV-testing events, pastors modelled receipt of HIV testing for church/community members. Also, HIV information was delivered through church bulletins/brochures, announcements, responsive readings, and through in-person and printed testimonials from PLHIV and from members who had been tested for HIV.

#### Ministry group level

Printed/video testimonials of members who had been tested for HIV with accompanying discussion guides, HIV education games (HIV Basics Jeopardy, Wheel of Awareness, HIV Testing Jeopardy) with facilitator instruction guides and printed Tool Kit materials were delivered through women's, men's and singles ministry group meetings.

#### Interpersonal/individual-level activities

Church/community members received brochures tailored by gender with information on HIV risks, prevention and testing. They also received scripted phone voice/text messages read by pastors and church liaisons to remind them about upcoming HIV-testing events and to increase intentions to seek HIV testing.

### Measures

#### Participant characteristics

Demographics (e.g., age, gender, income) and HIV testing (ever; yes/no) were assessed. Religiosity was measured with a summation of the seven-item version of the Religious Background and Behavior Scale [39] with six items on participants’ past year engagement in religious activities (e.g., prayed, attended a church service) using an eight-point Likert scale (0=never to 7=always) and one item on description of their religiosity (atheist=0 to religious=4) (*α*=0.77). HIV knowledge consisted of the summation of correct scores for 10 items (e.g., “You can get HIV if you share a drink, sink, shower, or toilet seat with someone who has AIDS”; *α*=0.56) from the HIV Knowledge Questionnaire [40].

#### HIV stigma beliefs

Similar to other stigma studies with church populations [35, 36], HIV stigma items were selected from national studies on HIV stigma [6, 41] and based on pre-intervention focus group findings [42]. The following five items assessed HIV stigma: (a) “How *comfortable* would you be sharing a pew with an HIV-positive person?” (symbolic contact); (b) “How strongly would you agree or disagree that scientists and doctors can be *trusted* to tell the truth about HIV?” (trust of authorities); (c) “How *afraid* are you of people who are infected with HIV?” (fear); (d) “If you were going to be tested for HIV, how *concerned* would you be that you might be treated differently or discriminated against if your test results were positive for HIV?” (discrimination); and (e) “How strongly would you agree or disagree that HIV-positive people are *responsible* for their illness?” (attitudes towards PLHIV) using a four-point Likert scale (e.g., 1=not at all afraid to 4=very afraid). A mean HIV stigma score was computed from items 1–4. The fifth stigma item (*responsible*) was not included in the final analyses of mean HIV stigma scores after preliminary analyses indicating poor reliability (*α*=0.37). After removing the *responsible* item from the mean HIV stigma scores, the *α*'s ranged from 0.50 to 0.55 across assessments.

#### Intervention exposure and satisfaction

Intervention exposure was assessed on participants’ exposure to 11 TIPS materials/activities (e.g., pastoral sermons, brochures/church bulletins, PLHIV/others’ testimonials, resource tables, health educator presentations) (1=yes; 0=no). Intervention participants’ satisfaction was assessed (e.g., how clearly HIV information on HIV delivered, how compassionately their pastor spoke about HIV) on a seven-point scale (1=not at all satisfied to 7=very satisfied).

### Data analysis

Frequencies and means were computed to describe participant characteristics, individual HIV stigma items, and intervention exposure and satisfaction. Analyses of mean HIV stigma scores (study outcome variable) were based on randomized churches, instead of individuals. Mean HIV stigma scores were examined using a mixed linear regression model (IBM SPSS version 20 and R version 2.12.1), which accounted for subject non-independence within church. The model included experimental condition and covariates (age, gender, income, religiosity, knowledge) as fixed-effect terms; churches nested in experimental condition were included as random effects terms. The mixed linear regression model found a 0 intracluster correlation within churches at baseline, 6 and 12 months; therefore, a simple linear regression model without clustered churches was used. Linear regression analysis was also conducted to determine if increased intervention exposure (dosage) was related to an increase in mean HIV stigma scores in the intervention group. Due to the significant attrition of participants across groups at 6 and 12 months, results were examined using simple mean imputation. Imputation yielded results similar to using complete case analysis; therefore, results on mean HIV stigma scores are reported from complete case analysis with individuals who completed questionnaires at baseline and in each subsequent time point.

## Results

### Participant characteristics

Overall, 543 church/community members were recruited from the four churches at baseline (*n*=235 intervention participants; *n*=308 comparison participants), as shown in Figure 1. Six-month and 12-month retention rates were 54% (*n*=127) and 40% (*n*=93), respectively, for the intervention group, and 62% (*n*=190) and 58% (*n*=178), respectively, for the comparison group. Participants were primarily Baptist (36%, *n*=196) and Church of God in Christ (32%, *n*=175) and had a mean age of 42.3 (SD=13.5). Also, most participants were female, single, highly religious (85% prayed daily; 79% attended church weekly) and had been tested for HIV, as shown in Table 1. The overall average HIV knowledge score at baseline was 7.49. Most frequently incorrect HIV knowledge items were: “A condom should be completely unrolled before it is placed on the penis” and “A person can get HIV by giving blood.” Except for sexual identity, intervention and comparison arm characteristics were similar. For HIV stigma and exposure measures, there were no differences between 6- and 12-month completers and non-completers on baseline data. Differential attrition occurred at 6 and 12 months on demographic measures; non-completers tended to be younger, male, have less education and income, and be community members.

**Figure 1:** Flow of churches and participants through completion of 12-month assessment: Taking It to the Pews Pilot Study: Kansas City Metropolitan Area, USA.

**Table 1:** Baseline participant characteristics

	Intervention group			Comparison group			
			
Survey measures	Church members	Community members	Overall	Church members	Community members	Overall	*p*
Age							0.27
18–29	25.1% (46)	23.1% (12)	24.7% (58)	22.6% (54)	17.3% (13)	21.3% (67)	
30–49	39.3% (72)	36.5% (19)	38.7% (91)	37.7% (90)	41.3% (31)	38.5% (121)	
50–64	34.4% (63)	40.4% (21)	35.7% (84)	36.8% (88)	41.3% (31)	37.9% (119)	
Gender							0.78
Male	27.3% (50)	63.5% (33)	35.3% (83)	29.3% (70)	57.3% (43)	36.0% (113)	
Female	72.7% (133)	36.5% (19)	64.7% (152)	68.2% (163)	42.7% (32)	62.1% (195)	
Sexual identity							0.01
Heterosexual	84.7% (155)	65.4% (34)	80.4% (189)	89.5% (214)	80% (60)	87.3% (274)	
Homosexual	1.1% (2)	–	0.9% (2)	2.1% (5)	4% (3)	2.5% (8)	
Bisexual	–	7.7% (4)	1.7% (4)	0.4% (1)	2.7% (2)	1.0% (3)	
Other/choose not to Answer	11.4% (21)	25.0% (13)	14.5% (34)	3.7% (9)	12% (9)	5.7% (18)	
Marital status							0.73
Single/separated/divorced/widowed	56.8% (104)	82.7% (43)	62.6% (147)	52.7% (126)	81.3% (61)	59.6% (187)	
Co-habiting/married	42.7% (78)	17.3% (9)	37.0% (87)	44.8% (107)	17.4% (13)	38.3% (120)	
Monthly income							0.10
$0–$1000	9.8% (18)	50.0% (26)	18.7% (44)	8.8% (21)	46.7% (35)	17.8% (56)	
$1001–$2000	14.2% (26)	5.8% (30)	12.3% (29)	18.4% (44)	18.7% (14)	18.5% (58)	
$2001–$2500	7.7% (14)	1.9% (1)	6.4% (15)	13.0% (31)	5.3% (4)	11.1% (35)	
$2501–$3000	14.8% (27)	7.7% (4)	13.2% (31)	11.7% (28)	4.0% (3)	9.9% (31)	
More than $3000	42.6% (78)	13.5% (7)	36.2% (85)	37.7% (90)	8.0% (6)	30.6% (96)	
Don't know	9.8% (18)	21.2% (11)	12.3% (29)	7.9% (19)	14.7% (11)	9.6% (30)	
Ever tested for HIV							0.12
Yes	71.2% (131)	74.5% (38)	71.9% (169)	72.8% (174)	86.7% (65)	76.1% (239)	
No	27.7% (51)	23.5% (12)	26.8% (63)	24.7% (59)	9.3% (7)	21% (66)	
Religiosity (M, SD) (possible range 0 to 46)	36.9 (7.3)	29.0 (10.8)	35.4 (8.6)	34.5 (7.4)	32.2 (10)	33.9 (8.2)	0.23
HIV knowledge (M, SD) (possible range 1 to 10)	7.4 (1.8)	7.2 (1.6)	7.4 (1.8)	7.7 (1.6)	7.2 (1.8)	7.6 (1.7)	0.36

Note: Percentages are based on actual rather than valid percent. Many of the variables reported in this table had missing data (ranging from 0 to 55), including those who did not respond because the question(s) were not applicable to them (ranging from 0 to 78).

### HIV stigma belief items and scores

Three out of the five HIV stigma items (sharing pew, trust doctors, afraid of PLHIV) were low at baseline (range: 1.59–2.04), as shown in Table 2. Although most of the stigma items showed reductions over time, none were significantly lower at 6 and 12 months. As shown in Table 3, HIV stigma mean did not differ at baseline between experimental groups (*p=*0.24). At 6 months, the difference in mean HIV stigma scores between experimental groups was not significant (*p*=0.92). However, the difference in mean HIV stigma scores within both groups at six months approached significance. At 12 months, the difference in mean HIV stigma scores between groups was not significant (*p*=0.70). Inclusion of age, gender, income, religiosity and HIV knowledge did not change non-significant differences between intervention and comparison group HIV stigma change scores at 6 and 12 months. However, linear regression with the baseline mean HIV stigma score as the outcome identified two predictors: HIV knowledge (*β*=−0.09, *p*=0.00) and income level (*β*=−0.19, *p*=0.04), meaning that increased HIV knowledge and higher levels of income were predictive of lower HIV stigma at baseline.

**Table 2:** HIV stigma items

	Intervention, M (SE)	Comparison, M (SE)	*P*
Comfortable sharing pew			
Baseline	1.59 (1.01)	1.78 (1.08)	0.10
6 months	1.45 (0.89)	1.63 (1.03)	0.91
12 months	1.68 (1.13)	1.69 (1.01)	0.37
Trust doctors are telling truth			
Baseline	2.01 (0.88)	2.04 (0.89)	0.78
6 months	1.95 (0.79)	2.01 (0.80)	0.82
12 month	1.97 (0.83)	2.00 (0.85)	0.92
Afraid of PLHIV			
Baseline	1.60 (0.84)	1.66 (0.90)	0.48
6 months	1.51 (0.80)	1.60 (0.77)	0.93
12 months	1.43 (0.77)	1.58 (0.80)	0.95
Concern discrimination			
Baseline	2.45 (1.03)	2.48 (0.99)	0.85
6 months	2.33 (0.96)	2.37 (1.01)	0.75
Post-test	2.31 (0.96)	2.41 (1.03)	0.87
People living with HIV responsible for illness			
Baseline	2.42 (0.87)	2.42 (0.85)	0.62
6 months	2.30 (0.88)	2.42 (0.83)	0.65
12 months	2.23 (0.88)	2.37 (0.80)	0.94

**Table 3:** Mean HIV stigma scores

	Intervention group			Comparison group			Between group change	
	
Outcome	Baseline (*n*=228), mean (SE)	6 Months (*n*=124), mean (SE)	12 Months (*n*=92), mean (SE)	Baseline (*n*=297), mean (SE)	6 months (*n*=181), mean (SE)	12 Months (*n*=172), mean (SE)	6 Months, *p*	12 Months, *p*
HIV stigma scores	1.92 (0.61)^a^	1.84 (0.55)^b^	1.91 (0.62)^c^	1.98 (0.59)	1.90 (0.58)^d^	1.93 (0.62)^e^	0.92	0.70

a Differences between groups at baseline: *p =*0.24.

b Differences within groups: *p=*0.09

c *p=*0.83

d *p=*0.08

d *p=*0.32.

### Intervention exposure

At 12 months, intervention group participants reported exposure to 72% (8 out of 11) of TIPS tools. Highly reported exposure to TIPS materials/activities included sermons (93%), posters (91%), resource tables (90%) and brochures/church bulletins (85%). Several intervention materials/activities were significantly related to (or trending towards) lower HIV stigma items at 12 months. Exposure to health professionals and PLHIV sharing HIV information and HIV-testing events were related to increased comfort in sharing pews with PLHIV (*p*=0.06 and *p*=0.07, respectively). Brochures/church bulletins and pastor-delivered sermons were related to decreased fear of PLHIV (*p=*0.078 and 0.01, respectively). Increased exposure to the intervention was not significantly related to reductions in mean HIV stigma scores at 6 and 12 months (*p=*0.21 and *p=*0.20, respectively).

### Participant satisfaction

Overall, intervention participants reported being highly satisfied with how clear HIV information was delivered (90%), how compassionately their pastor discussed HIV (81%) and how often HIV information and events were offered (80%).

## Discussion

This study examined HIV stigma as an outcome in an HIV education and testing pilot intervention implemented in AA churches. To our knowledge, this the first HIV prevention intervention study to assess HIV stigma outcomes among an AA church population, inclusive of church and community members. Overall, participants were highly religious (e.g., 79% attended church weekly), thus highlighting the potential reach and influence of churches to deliver ongoing HIV stigma reduction messages/activities on compassion, support and advocacy for PLHIV with their church communities [24, 27, 35]. Participants were also quite knowledgeable about HIV. However, among these and other factors hypothesized to be related to HIV stigma, only greater HIV knowledge and income were predictive of a lower HIV stigma score at baseline. Other church-population studies have also found a relationship between HIV knowledge and stigma [35, 36], and they have emphasized the role AA churches can serve in correcting HIV myths and sharing facts about HIV transmission/prevention to address HIV stigma.

No significant differences were found between intervention and comparison groups for the individual HIV items or composite scores assessed at baseline, 6-month and 12-month assessments. Also, subgroup analysis found no significant effects on HIV stigma score outcomes based on hypothesized factors. Several aspects of the study may have contributed to the null effects. Significant reductions in composite HIV stigma scores may have been difficult to achieve due to the low levels of HIV stigma measured at baseline; most of the stigma items ranged from 1.59 to 2.04 (possible range 1 to 4). However, higher levels were found for two stigma items: concern about discrimination if tested HIV-positive and PLHIV being responsible for illness. Yet, small reductions in HIV stigma scores, which trended towards significance within experimental groups at six months, and HIV stigma items occurred over time, possibly suggesting that the intervention could bring about small shifts in HIV stigma.

The process evaluation revealed that participants were highly satisfied with the TIPS intervention. Also, some of the TIPS materials/activities, particularly those (e.g., sermons, printed and video testimonials, brochures/church bulletins) delivered in church services and group ministries, were significantly related to lower HIV stigma beliefs. However, with the near-floor-level HIV stigma beliefs at baseline, an intervention with increased strength and dosage of these components and inclusion of more HIV stigma reduction strategies may be needed to shift stigma beliefs. For example, studies have shown that altruistic intervention strategies [43, 44] may contribute to reductions in HIV stigma; yet, most of these studies were individually or group delivered and have not been examined with communities of ethnic minority participants. There is more to learn about mobilizing church communities to address HIV stigma, particularly in using a CBPR-guided approach, through various church ministries to increase church reach of HIV stigma reduction strategies. These church ministry strategies could include the use of: (a) community outreach ministries (support groups, food/clothing pantry services, prayer circles) for those affected by and living with HIV; (b) church services with pastors/church leaders role modelling and promoting HIV compassion through brief plays and liturgical readings; (c) ministry group discussions on HIV stigma; and (d) self-assessments on personal HIV stigma beliefs and strategies to address one's stigmatizing beliefs. Also, future research is needed on how church/community members can be trained to serve as health advocates assisting PLHIV with linkage to and maintenance of health and social services.

This study had limitations, particularly related to methodological issues. Given that it was a pilot study, the null effects could possibly be attributed to insufficient power. Although each of the four participating churches on average had 136 participants at baseline, more churches are probably needed to detect a significant difference in HIV stigma. Further HIV stigma research with church-based interventions is needed using an appropriately powered research designs. This study also incurred significant attrition at 6 and 12 months, especially among participants who tended to be younger, male and less educated/lower income. Most of these characteristics (male, less educated/income) were highly representative of our community participants, who at times were difficult to contact due their transience and irregular contact with participating churches. Yet even with participant attrition, differential stigma beliefs between experimental groups were not detected. Also, the reliability of the HIV stigma composite measure was moderate at best in magnitude even after dropping one of the HIV stigma items from the HIV stigma composite variable. Possibly increasing the number of questions and dimensionality of the HIV stigma composite scale may enhance the reliability of this variable. Additionally, to increase understanding of HIV stigma in church settings, inclusion of religion-attributed HIV stigma measurements [35] and relevant behavioural outcomes (e.g., supportive acts similarly extended to persons with other chronic diseases) may be important to consider. For example, measures inclusive of hypothetical situations in which HIV stigma (and related compassionate acts) could occur may be more appropriate for measuring stigma in church populations [45]. Furthermore, it is possible that participants socially responded to stigma questions, especially considering surveys were completed at participating churches. Yet, baseline HIV stigma findings (and non-existent ICCs) suggest that if social responding occurred, it was similar between the randomized groups. Still, use of measurements to detect social responding among church populations may be necessary [46]. Finally, since this community-engaged study was tailored for a specific AA population and included pastors willing to participate in addressing HIV, findings may not generalize to other faith-based settings.

## Conclusions

With their reach and influence, AA churches can play an important role in changing HIV stigma beliefs in their church communities, particularly by promoting compassion and providing support for PLHIV, while advocating for elimination of injustices and discrimination against PLHIV. Rigorous AA church-based studies are needed that: (a) focus on measurement and retention issues in evaluating HIV stigma beliefs in church populations and (b) test AA church-based interventions that equip faith leaders with religiously tailored stigma reduction tools and strategies.

## References

[1] Centers for Disease Control and Prevention (2011). Disparities in diagnosis of HIV infection between Blacks/African Americans and other Racial/Ethnic populations – 37 States, 2005–2008. Morb Mortal Wkly Rep.

[2] Centers for Disease Control and Prevention (2010). Vital signs: HIV testing and diagnosis among adults – United States, 2001–2009. MMWR.

[3] Crum NF, Riffenburgh RH, Wegner S, Agan BK, Tasker SA, Spooner KM (2006). Comparisons of causes of death and mortality rates among HIV-Infected persons: analysis of the pre-, early, and late HAART (Highly Active Antiretroviral Therapy) eras. JAIDS.

[4] Hammer SM, Saag MS, Schechter M, Montaner JS, Schooley RT, Jacobsen DM (2006). Treatment for adult HIV infections: 2006 recommendations of the International AIDS Society – USA panel. JAMA.

[5] Panos G, Samonis G, Alexiou VG, Kavarnou GA, Charatsis G, Falagas ME (2008). Mortality and morbidity of HIV infected patients receiving HAART: a cohort study. Curr HIV Res.

[6] Herek GM, Capitanio JP, Widaman KF (2002). HIV-related stigma and knowledge in the United States: prevalence and trends, 1991–1999. Am J Public Health.

[7] Fullilove MT, Fullilove RE (1999). Stigma as an obstacle to AIDS action: the case of the African American community. Am Behav Scientist.

[8] Kinsler JJ, Wong MD, Sayles JN, Davis C, Cunningham WE (2007). The effect of perceived stigma from a health care provider on access to care among a low-income HIV-positive population. AIDS Patient Care STDs.

[9] Sengupta S, Strauss RP, Miles MS, Roman-Isler M, Banks B, Corbie-Smith G (2010). A conceptual model exploring the relationship between HIV stigma and implementing HIV clinical trials in rural communities of North Carolina. N C Med J.

[10] Mahajan AP, Sayles JN, Patel VA, Remien RH, Ortiz D, Szekeres G (2008). Stigma in the HIV/AIDS epidemic: a review of the literature and recommendations for the way forward. AIDS.

[11] Widaman K, Capatino J (2005). When sex equals AIDS: symbolic stigma and heterosexual adults’ inaccurate beliefs about sexual transmission of AIDS. Soc Probl.

[12] Clark HJ, Lindner G, Armistead L, Austin BJ (2004). Stigma, disclosure, and psychological functioning among HIV-infected and non-infected African-American women. Wom Health.

[13] Sengupta S, Banks B, Jonas D, Miles M, Smith GC (2011). HIV interventions to reduce HIV/AIDS stigma: a systematic review. AIDS Behav.

[14] Prachakul W, Grant JS, Keltner NL (2007). Relationships among functional social support, HIV-related stigma, social problem solving, and depressive symptoms in people living with HIV: a pilot study. J Assoc Nurses AIDS Care.

[15] Galvan FH, Davis EM, Banks D, Bing EG (2008). HIV stigma and social support among African Americans. AIDS patient care and STDs.

[16] Deacon H (2006). Towards a sustainable theory of health-related stigma: lessons from the HIV/AIDS literature. J Community Appl Soc Psychol.

[17] Goffman E (1963). Stigma: notes on the management of spoiled identity.

[18] Parker R, Aggelton P (2003). HIV and AIDS-related stigma and discrimination: a conceptual framework and implications for action. Soc Sci Med.

[19] Pew Forum on Religion and Public Life (2009). A religious portrait of African Americans, U.S. Religious Landscape Survey.

[20] Barna Group Church attendance. http://www.barna.org/barna-update/article/13-culture/286-how-the-faith-of-african-americans-haschanged?q=church+attendance+2007+african+americans.

[21] Taylor RJ, Chatters LM, Levin J (2004). Religion in the lives of African Americans: social, psychological, and health perspectives.

[22] Davis DT, Bustamante A, Brown CP, Wolde-Tsadik G, Savage EW, Cheng X (1994). The urban church and cancer control: a source of social influence in minority communities. Public Health Rep.

[23] Lincoln CE, Mamiya LH (1990). The Black Church and the African American experience.

[24] Berkley-Patton J, Martinez D, Bowe-Thompson C, Hawes S, Moore E, Williams E (2013). Examining church capacity to develop and disseminate a religiously-appropriate HIV tool kit with African American churches. J Urban Health.

[25] Derose KP, Mendel PJ, Palar K, Kanouse DE, Bluthenal RN, Castaneda LW (2011). Religious congregations’ involvement in HIV: a case study approach. AIDS Behav.

[26] Agate L, Cato-Watson D, Mullins JM, Scott GS, Rolle V, Markland D (2005). Churches United to stop HIV (CUSH): a faith-based HIV prevention initiative. J Natl Med Assoc.

[27] Berkley-Patton J, Bowe-Thompson C, Bradley-Ewing A, Hawes S, Moore E, Williams E (2010). Taking It to the Pews: a CBPR-guided HIV awareness and screening project with black churches. AIDS Educ Prev.

[28] Griffith DM, Campbell B, Allen JO, Robinson K, Stewart SK (2010). Your blessed health: an HIV prevention program bridging faith and public health communities. Public Health Rep.

[29] MacMaster SA, Jones JL, Rasch R, Crawford SL, Thompson S, Sanders E (2007). Evaluation of a faith-based culturally relevant program for African American substance users at risk for HIV in the Southern United States. Res Soc Work Pract.

[30] Wingood GM, Simpson-Robinson L, Braxton ND, Raiford JL (2011). Design of a faith-based HIV intervention: successful collaboration between a university and a church. Health Promot Pract.

[31] Coyne-Beasley T, Schoenbach VJ (2000). The African-American church: a potential forum for adolescent comprehensive sexuality education. J Adolesc Health.

[32] Smith J, Simmons E, Mayer KH (2005). HIV/AIDS and the Black church: what are the barriers to prevention services?. J Natl Med Assoc.

[33] Ayers JR (1995). The quagmire of HIV/AIDS related issues which haunt the church. J Pastoral Care.

[34] Khosrovani M, Poudeh R, Parks-Yancy R (2008). How African-American ministers communicate HIV/AIDS-related health information to their congregants: a survey of selected Black churches in Houston, TX. Ment Health Relig Cult.

[35] Muturi N, Soontae A (2010). HIV/AIDS stigma and religiosity among African American women. J Health Comm.

[36] Lindley LL, Coleman JD, Gaddist BW, White J (2010). Informing faith-based HIV/AIDS interventions: HIV-related knowledge and stigmatizing attitudes at Project F.A.I.T.H. churches in South Carolina. Public Health Rep.

[37] Israel BA, Eng E, Schulz AJ, Parker EA, Israel BA, Eng E, Schulz AJ, Parker EA (2005). Introduction to methods in community-based participatory research for health. Methods in community-based participatory research for health.

[38] Berkley-Patton J, Bowe Thompson C, Williams ED, Hawes S, Moore E, Wainwright S (2012). Engaging African American faith leaders in developing, implementing, evaluating, and disseminating church-based HIV interventions for youth and adults. https://apha.confex.com/apha/140am/webprogram/Paper270882.html.

[39] Connors GJ, Tonigan JS, Miller WR (1996). A measure of religious background and behavior for use in behavior change research. Psychol Addict Behav.

[40] Carey MP, Morrison-Beedy D, Johnson BT (1997). HIV knowledge questionnaire: development and evaluation of a reliable, valid, and practical self-administered questionnaire. AIDS Behav.

[41] Herek GM (1999). AIDS and stigma in the United States. Am Behav Scientist.

[42] Berkley-Patton J, Hawes SM, Moore E, Bowe-Thompson C, Williams E, Martinez D (2012). Examining facilitators and barriers to HIV testing in African American churches using a community-based participatory research approach. Ann Behav Med.

[43] Ashworth CS, DuRant RH, Gaillard G, Rountree J (1994). An experimental evaluation of an AIDS education intervention for WIC mothers. AIDS Educ Prev.

[44] Moore D, Onsomu EO, Abuya BA (2011). Entertainment-education for starting HIV/AIDS discussions and reducing stigma: African American college students’ reactions to the film Yesterday. Res Educ Policy.

[45] Heredia D, Martinez DA, Gutierrez A, Champassak SL, Berkley-Patton J, Goggin K (2013). HIV-related stigma as a function of age and HIV-knowledge in a Latino church sample. Ann Behav Med.

[46] Strahan R, Gerbasi KC (1972). Short, homogeneous versions of the Marlowe–Crowne social desirability scale. J Clin Psychol.

# Sub article: Reducing shame in a game that predicts HIV risk reduction for young adult men who have sex with men: a randomized trial delivered nationally over the web

**DOI:** 10.7448/IAS.16.3.18716

**Published:** 2013-11-13

**Authors:** John L Christensen, Lynn Carol Miller, Paul Robert Appleby, Charisse Corsbie-Massay, Carlos Gustavo Godoy, Stacy C Marsella, Stephen J Read

**Affiliations:** 1 Department of Communication and Center for Health, Intervention, & Prevention, University of Connecticut, Storrs, CT; 2 Annenberg School for Communication and Journalism, University of Southern California, Los Angeles, CA; 3 Department of Paediatrics (CHLA), Keck School of Medicine, University of Southern California, Los Angeles, CA; 4 S. I. Newhouse School of Public Communication, Syracuse University, Syracuse, NY; 5 Institute for Creative Technologies, University of Southern California, Playa Vista, CA; 6 Department of Psychology, University of Southern California, Los Angeles, CA

**Keywords:** stigma, shame, intervention, serious games, SOLVE, HIV, AIDS, sexual risk-taking, men who have sex with men (MSM)

## Abstract

**Introduction:**

Men who have sex with men (MSM) often face socially sanctioned disapproval of sexual deviance from the heterosexual “normal.” Such *sexual stigma* can be internalized producing a painful affective state (i.e., shame). Although shame (e.g., addiction) can predict risk-taking (e.g., alcohol abuse), sexual shame's link to sexual risk-taking is unclear. Socially Optimized Learning in Virtual Environments (SOLVE) was designed to reduce MSM's sexual shame, but whether it does so, and if that reduction predicts HIV risk reduction, is unclear. To test if at baseline, MSM's reported past unprotected anal intercourse (UAI) is related to shame; MSM's exposure to SOLVE compared to a wait-list control (WLC) condition reduces MSM's shame; and shame-reduction mediates the link between WLC condition and UAI risk reduction.

**Methods:**

HIV-negative, self-identified African American, Latino or White MSM, aged 18–24 years, who had had UAI with a non-primary/casual partner in the past three months were recruited for a national online study. Eligible MSM were computer randomized to either WLC or a web-delivered SOLVE. Retained MSM completed baseline measures (e.g., UAI in the past three months; current level of shame) and, in the SOLVE group, viewed at least one level of the game. At the end of the first session, shame was measured again. MSM completed follow-up UAI measures three months later. All data from 921 retained MSM (WLC condition, 484; SOLVE condition, 437) were analyzed, with missing data multiply imputed.

**Results:**

At baseline, MSM reporting more risky sexual behaviour reported more shame (*r*_s_=0.21; *p*<0.001). MSM in the SOLVE intervention reported more shame reduction (*M=*−0.08) than MSM in the control condition (*M*=0.07; *t*(919)=4.24; *p*<0.001). As predicted, the indirect effect was significant (point estimate −0.10, 95% bias-corrected CI [−0.01 to −0.23] such that participants in the SOLVE treatment condition reported greater reductions in shame, which in turn predicted reductions in risky sexual behaviour at follow-up. The direct effect, however, was not significant.

**Conclusions:**

SOLVE is the first intervention to: (1) significantly reduce shame for MSM; and (2) demonstrate that shame-reduction, due to an intervention, is predictive of risk (UAI) reduction over time.

## Introduction

From 2006 to 2009, there was a 21% increase in HIV incidence among those aged 13–29, largely due to a 34% increase in young men who have sex with men (MSM) [1]. Despite successes [2], HIV prevention for MSM falls short, perhaps because interventions to reduce unprotected anal intercourse (UAI) do not address the “discrimination and homophobia,” – and stigma – that “fuel the HIV epidemic in gay and bisexual men” [3]. S*exual stigma* has been defined as “the negative regard, inferior status, and relative powerlessness that society collectively accords to any non-heterosexual behaviour, identity, relationship, or community” [4].

MSM can internalize sexual stigma [4], producing shame [5–7]. Shame may operate quite differently from other painful affective states, such as fear, that can impact risk-taking [8]. Shame provides immediate feedback (i.e., punishment) regarding whether one's past, current or anticipated future (sexual) behaviour is in line with one's moral standards [9]. Shame has been differentiated from guilt empirically in that shame results from seeing one's situation as unchangeable (stable) and attributable to the individual as a whole (global) whereas guilt is caused by unstable, non-global attributions [10, 11]. When stable desires (e.g., for other men) and moral standards (e.g., one should not desire sex with a man) conflict and neither seems changeable, one may perceive the self as responsible for uncontrollable outcomes, and the global self may be devalued [5], resulting in a state of shame [9, 11, 12]. Shame and guilt can each be experimentally activated (e.g., imagining a given situation) and produce differential brain patterns as assessed by functional magnetic resonance imaging [13].

States of shame, but not guilt, have been linked to a variety of negative health outcomes [9]. For example, recovering alcoholics’ displays of shame (e.g., humped shoulders) when describing their last drink predicted subsequent relapse severity [14]. States of shame differ from trait-like constructs (e.g., internalized homophobia that inconsistently predicts sexual risk-taking [15]) in that they involve *specific situational contexts* (e.g., before an attractive partner). For example, participants given misleading feedback – suggesting their responses conflicted with their self-standards – felt shame [16]. If conflicting standards and responses produce shame, perhaps reducing perceived conflict might reduce shame. Conflict between some MSM's self-standards (e.g., having sex with men is wrong) and their actual reactions (e.g., desiring men) is one unresolvable shame-producing conflict involving potentially changeable self-standards (e.g., desiring another man is normal/acceptable for me). Changing self-standards might entail changing beliefs about others’ beliefs (e.g., others share and/or accept – rather than reject – me/my desires). Reducing UAI in line with standards (e.g., having sex with another man is normal; I should not risk HIV) may then be easier.

Sex-positive interventions (e.g., accepting, sharing men's same-sex desires as normal) might help MSM differentiate desires and behaviour that need not change (e.g., sex) from self-harmful behaviour that can and must change (e.g., risky sex). To reduce UAI via shame reduction, interventions might: (1) simulate typical sexually and emotionally charged (potentially shame-activating) situations where participants could choose sexual risks (or not), (2) interrupt and unpack affect-based, and as neuroscience models of decision-making suggest [17], automatic processes guiding risky decision-making [18] and (3) remain sex-positive (e.g., shared sexual desires as differentiated from self-harmful choices such as UAI).

Such interventions would be challenging using traditional one-on-one or group interventions. Thus, using an approach called Socially Optimized Learning in Virtual Environments (SOLVE), Miller and her colleagues [19–24] developed and tested interactive, media-based interventions designed to simulate and immerse high-risk young adult MSM in affectively charged risky situations (e.g., an attractive man desiring sex but refusing to use a condom) typically confronted on first dates or “hook-ups.” The player's decisions for his character affect the narrative while learning from virtual mentors/guides and sex partners who accept and share participants’ desires. Throughout the narrative, guide characters (e.g., peers, one's virtual future self) use an ICAP process that involves *(I) interrupting automatic* risky choices, *(C) challenging* those choices with persuasive messages, *(A) acknowledging, accepting and sharing* MSM's emotions/motives (e.g., desires for men) and *(P) providing* a way and skills for MSM to be safe [19–24]. Two prior SOLVE randomized controlled trials (RCTs) demonstrated UAI risk reduction over time. In one trial, at an HIV testing site, HIV-negative MSM exposed to SOLVE (human actors with CD-ROM technology) versus controls (only receiving post HIV-negative counselling) had lower levels of UAI after 10 weeks [21]. In a second, younger (aged 18–24) MSM exposed to SOLVE (interactive DVD with human actors) versus a wait-list control (WLC) condition had lower levels of UAI after three months [19, 20, 24].

Left unclear, however, was whether SOLVE interventions actually reduce shame as intended. To test this, our team, funded by a grant from the National Institute of Mental Health (NIMH), developed an intervention with virtual intelligent agents that incorporated this SOLVE approach into a 3-D animated serious game. Our first hypothesis is that MSM reporting more shame at baseline will report more UAI over the past three months. We also hypothesize that MSM exposed to SOLVE will report immediate shame reductions compared to a WLC condition, and that this shame reduction will predict change in UAI over three months. Finally, shame will mediate the link between condition and UAI change.

## Methods

### Trial design

This online RCT tested the effectiveness of SOLVE, a downloadable simulation video game, compared to a WLC condition in reducing shame and directly or indirectly (via shame reduction) reducing UAI over three months. Randomization was imbalanced [2:1] to compensate for differential loss of participants in the SOLVE treatment condition due to unaddressable technical issues identified in pre-trial piloting (see limitations for details). Online data collection software (Qualtrics) automatically generated the random allocation sequence and assigned participants to condition. Researchers and staff were blind to condition assignment at enrolment, but some were subsequently unblinded to prohibit participant re-enrolment. A data analysis plan was consistently used within and across conditions (addressing out-of-range, missing values; data reduction procedures; outliers, missingness; statistical assumption checks).

### Participants

These data come from the 935 MSM who enrolled between February and November 2012. In this primary prevention intervention, participants were eligible only if they self-reported that they: (1) had a prior HIV-negative test result; (2) lived in the United States; (3) were between 18 and 24 years of age; and (4) engaged in UAI with a non-primary male partner during the three-month period prior to enrolment. We defined a non-primary partner as a man with whom the participant was not currently in a romantic relationship. We targeted young adult MSM because of this group's considerable impact on the epidemiology of HIV in the United States; from 2006 to 2009, estimated HIV incidence increased significantly among MSM only [1]. Because of budget limitations, characters of only three racial groups could be developed for the game. Since the groups most at risk for HIV were Black/African American, Hispanic/Latino or White/Caucasian [1], participants needed to self-identify as belonging to one of these groups. Exclusion criteria included participation in prior SOLVE studies, non-corrected vision/hearing impairment and a history of injecting non-prescribed drugs. Additional exclusion criteria after allocation (resulting in disenrollment and study discontinuation) included not completing baseline measures (since data would have been unanalyzable) and, in the SOLVE treatment condition, being unwilling or unable to download the game and/or being unable to play at least one of two game levels. To recruit, we posted clickable banner ads on websites frequented by the target population. Participants could enter a lottery drawing at baseline with a 1:40 chance of receiving a $100 gift card. At three-month follow-up, participants were offered a $25 gift card.

### Description of SOLVE intervention and control condition

The SOLVE intervention immerses MSM in a virtual world simulating many common obstacles to safer sex. The intervention is guided by cognition-based approaches such as the Theory of Planned Behaviour [25] and Social Cognitive Theory [26] while also capitalizing upon recent advances in neuroscience that suggest emotions are critical during decision-making [17, 18]. The interactive narrative begins after the player customizes his avatar's hair colour, skin tone and clothing style. On the first level, the player can flirt with potential sex partners at a virtual house party (see Figure 1). As the drama unfolds, the player encounters a series of choice points where he must make self-regulatory decisions (e.g., accept/decline multiple offers of alcohol and casual sex). Next, he is at a potential sex partner's apartment. Here, the player gains experience initiating a conversation about safe sex, negotiating condom use and refusing sex if a condom is unavailable. When the player makes a risky choice, he is immediately exposed to a contextualized ICAP intervention. After choosing to engage (or not) in virtual sex, there is a tailored recap sequence where the player's virtual behaviour is evaluated and linked to real-life consequences. Players then move to level two – a virtual nightclub – where the artificially intelligent characters and decision points are more challenging. A primary goal of the intervention is to reduce shame associated with sexual stigma by enabling MSM to more consciously acknowledge their desires and to recognize that their desires are normal. This is achieved through careful design of the characters, dialogue and storylines. For example, the player's avatar consistently models positive self-appraisals and comfort with his sexuality/desires. Through conversations with other characters, he is exposed to dialogue designed to decrease feelings of isolation and inferiority while increasing self-worth. In addition, messages providing HIV knowledge and risk-reduction skills are written/delivered in a non-judgmental, gay-positive manner. Negative feelings associated with religious, societal and familial rejection are also addressed. Participants in the control condition completed the same baseline and immediate post-test measures as those in the SOLVE treatment condition but did not play the game at this time.

**Figure 1:** Agents at a virtual house party.

### Measures

Consistent with the trial registry, our primary outcome was change in counts of risky sexual behaviour over three months and our secondary outcome was change in shame from baseline to immediate post-test.

#### Risky sexual behaviour

At baseline, participants reported the number of times they engaged in UAI with non-primary partners during the past three-months (i.e., receptive and insertive anal sex without a condom). UAI was reassessed at three-month follow-up.

#### Shame

We measured shame immediately before and immediately after the intervention (or WLC waiting period) using five items from an existing subscale of Watson and Clark's (1994) Positive and Negative Affect Schedule – Expanded Form designed to assess state shame [27]: *ashamed, blameworthy, angry at self, disgusted with self and dissatisfied with self*. Participants indicated how they felt at the present moment using a 1 (*very slightly or not at all*) to 5 (*extremely*) scale. Responses were averaged; internal consistency was high (baseline *α*=0.86; immediate post-test *α*=0.90).

### Ethical considerations

This RCT was approved by the University of Southern California's institutional review board (IRB). To ensure confidentiality, participants were only identified via email address, which was deleted upon study completion.

### Data analysis

Of the 935 participants, 14 (seven per condition) “completed” their baseline measures but responded to each shame item with “refuse to answer” and therefore could not be included in the main analyses. Data from 921 MSM (control condition, 484; SOLVE treatment condition, 437) were analyzed.

Using the mean function in SPSS-20, we replaced missing values on the five baseline shame items with the mean of values present for that participant. We then calculated a shame change score. Simple difference scores are typically correlated with baseline values and so we used residualized change scores, which eliminates this dependency [28, 29]. Residuals for shame were calculated by regressing immediate post-test values (Y) on baseline values (X), affording estimates of predicted Y values (Y’) that are then subtracted from Y (and saved as unstandardized residuals). Positive scores indicate an increase; negative scores a decrease. Where a residualized score could not be calculated due to completely missing immediate post-test data, we used the average residual of participants with matching baseline shame scores. Change in UAI was computed by regressing three-month follow-up values on baseline values. UAI change scores of those lost to follow-up were estimated in M*Plus* 7 using a full-information maximum likelihood (FIML) procedure [30].

To test whether prior UAI predicts baseline shame, we used the Spearman correlation coefficient because of the positive skew that is typical of count variables such as UAI (*z*=30.0; *p*<0.001). An independent-samples *t*-test examined whether shame was reduced in the SOLVE treatment condition versus control condition. Tests are reported as two-tailed and a *p*-value of 0.05 indicates statistical significance. To test the proposed mediation model, we used the “Model Indirect” command within M*Plus* 7 [30]. Bias-corrected 95% confidence intervals were generated using 5000 bootstrap samples. An indirect effect is observed if the confidence interval is entirely above or below zero. Using a Mahalanobis distance critical value of 13.8, we detected and removed three multivariate outliers in the WLC and five in the SOLVE treatment condition.

## Results

The CONSORT [31, 32] diagram of participant flow presented in Figure 2 provides information regarding the number of participants assessed for eligibility, randomized to condition, lost to follow-up and included in the main analyses. Of those receiving the allocated intervention or control, 73% were White/Caucasian, 14% were Latino/Hispanic and 13% were Black/African American. The majority identified as gay or homosexual (76%) and had at least some post-secondary training (82%). Approximately 13% reported living in a rural geographic area. Participants were 21 years old on average, had engaged in UAI about 12 times in the past three months and reported relatively low levels of baseline shame (1.7 on a five-point scale). See Table 1 for measures split by condition. A randomization check confirmed that the conditions did not significantly differ on any of the baseline measures. Consistent with past work [19–21, 24], no ethnic differences for any analyses were found.

**Figure 2:** Participant flow diagram comparing the SOLVE treatment condition and control condition.

**Table 1:** Baseline characteristics by condition

	Control (*N=*491)	Treatment (*N=*444)	Difference by condition
Race/ethnicity			*p*=0.77
White/Caucasian	349 (71.1%)	338 (76.1%)	
Latino/Hispanic	76 (15.5%)	55 (12.4%)	
Black/African American	66 (13.4%)	51 (11.5%)	
Sexual orientation			*p*=0.49
Gay/homosexual	376 (76.6%)	331 (74.5%)	
Bisexual	56 (11.4%)	65 (14.7%)	
Other	57 (11.6%)	46 (10.4%)	
Postsecondary education	394 (80.2%)	371 (83.6%)	*p*=0.48
Rural geographic area	66 (13.4%)	57 (12.8%)	*p*=0.74
Age (mean, SD)	21.3 (1.8)	21.3 (1.7)	*p*=0.73
UAI (mean, SD)	12.5 (11.8)	11.7 (12.5)	*p*=0.32
Shame (mean, SD)	1.7 (0.82)	1.7 (0.77)	*p*=0.54

### Main analyses

Risky sexual behaviour and shame. As predicted, we found that prior sexual risk-taking was positively correlated with baseline shame, *r*_s_=0.21, *p<*0.001, 95% CI [0.15–0.27].

#### Change in shame due to the SOLVE treatment versus control condition

Exposure to the intervention led to immediate mean shame reduction for those in the SOLVE treatment condition (*M*=−0.08, *SD*=0.51, *n*=437) while it unexpectedly led to an increase (*M*=0.07, *SD*=0.54, *n=*484) in the control condition (Table 2). The difference was statistically significant, *t*(919)=4.24, *p<*0.001. Cohen's *d*=0.29.

#### Mediation analysis

We used a bootstrapping approach to assess the effect of condition on UAI change indirectly through shame change. As expected, condition predicted shame change (path a), *B=*−0.14, SE=0.03, 95% bias-corrected CI [−0.07 to −0.20], and shame change predicted UAI change (path b), *B=*0.73, SE=0.36, 95% bias-corrected CI [0.03–1.45]. The direct effect of the intervention on UAI change was not significant but, as hypothesized, the indirect effect was negative and statistically different from zero; point estimate=−0.10, 95% bias-corrected CI [−0.01 to −0.23]. Participants in the SOLVE treatment condition reported greater reductions in shame, which in turn influenced reductions in risky sexual behaviour at follow-up.

**Table 2:** Means for shame items

	Control		Treatment	
		
	Pre-test	Post-test	Pre-test	Post-test
Ashamed	1.70	1.73	1.66	1.55
Blameworthy	1.69	1.71	1.60	1.60
Angry at self	1.68	1.70	1.70	1.58
Disgusted with self	1.73	1.63	1.57	1.49
Dissatisfied with self	1.84	1.91	1.93	1.70
Scale total	1.73	1.74	1.69	1.58

## Discussion

Our *a priori* hypothesis that a web-based simulation game would reduce sexual shame was supported. This reduction, in turn, indirectly reduced UAI following the intervention. These findings are exciting because they suggest that, for some MSM, shame reduction may be an important intervention component resulting in UAI change. Condition, however, did not have a direct effect on UAI in this study, as it had in two prior SOLVE studies. This SOLVE intervention differed from earlier versions in a number of ways (e.g., animated versus life actors; national sample versus Los Angeles; participation over the web under “real-life” and less controlled conditions). We are currently exploring potential suppressors of the link between condition and UAI that might have resulted in an overall insignificant direct effect [33]. Such mediational analyses are critical in identifying what works (and does not) for whom, to better optimize and cumulatively advance our risk-reduction interventions. Additional analyses with this sample indicated that measured fear was not predictive of risk-reduction, indicating that shame specifically matters, and not negative emotions generally.

To our knowledge, this is the first HIV prevention intervention to demonstrate shame reduction. Although we did not expect shame to change in the control condition, a slight increase was observed. Might increased shame be a by-product of recently reporting and ruminating about one's prior risky behaviour? Future research should examine how survey items may negatively affect participants.

SOLVE is one of a family of recent theory-based interventions to reduce shame among high-risk individuals [34]. These interventions share a focus on greater self-awareness of emotions, goals, behaviours and associated barriers while fostering acceptance of parts of the self that cannot change. SOLVE's focus on counselling and social support can be compared to several intervention efforts aimed at reducing HIV stigma. A recent systematic review by Sengupta and colleagues identified four interventions that attempted to reduce HIV stigma using this approach [35]. The duration of these interventions ranged from six hours to one year. SOLVE, in comparison, is brief (e.g., 30 minutes).

SOLVE, unlike most HIV prevention interventions, is completely deliverable over the Internet. In this regard, SOLVE is similar to at least two other recent interventions designed to improve the wellbeing of MSM. In one study, a web-based expressive writing intervention for gay-related stress led to improvements in psychosocial functioning, including increased sexual orientation openness [36]. Another web-based HIV prevention intervention called *Keep it Up!* successfully used videos, animation and games to reduce rates of UAI [37]. Collectively, these interventions demonstrate the plausibility of rapid dissemination and broader reach – with potentially greater cost effectiveness.

### Limitations

Nationwide web-based testing of an intervention is challenging. Glitches internal to the game itself were remedied pre-trial; however, some participants would not download an executable file. Others could not play the game given hardware (e.g., CPU, Internet speed, memory, disk space, graphics card) and software configurations (e.g., operating system age and version; conflicts with other software). Given rapid computer configuration changes, this remains a “moving target.” Although “dummying down” the technology is tempting, two considerations argued against that: (1) prior work indicates that immersion/presence predicts behaviour change [38]; and (2) if effective, the intervention might then have a shorter “shelf-life” after trials conclude. We attempted to use meta-data to identify participants with technical issues but found no discernable predictor pattern. A lesson learned is that a mini-game in the screener might reliably discern if players, randomized to a game condition, could (or would) download/play it. Although it is possible to play the game at a local intervention site or cyber-café, we recommend privacy since the player's virtual choices may be subject to social desirability bias if others are present.

A second limitation was our retention rate (69%). At three-months, this was under the desired cut-off of 70% for “best evidence,” specified by the Centres for Disease Control and Prevention [39]. Although on-line studies are still rare, some researchers have assessed the retention of MSM in RCTs and have found it can be surprisingly low over three months. Across four other studies, the rates were 15% [40], 25% [41], 53% [42] and 95% [43]. Thus, the current study had one of the highest retention rates for online studies over three months with MSM to date.

Third, financial constraints precluded developing characters other than Black, White or Latino, making the game potentially less suitable for other MSM. Even for Black and Latino MSM, finances constrained our ability to include culturally targeted dialogue, pop-culture references and behavioural choices as we had in prior interventions. Nevertheless, no ethnic differences were found in the current work. Financial constraints also limited our ability to develop storylines to address unique issues faced by people living with HIV (PLHIV). In addition to shame as a manifestation of sexual stigma, it is likely that some MSM living with HIV would also be experiencing shame associated with HIV-related stigma and discrimination. Future research should explore how intersecting stigmas might be best addressed in serious games and other technology-enabled interventions for PLHIV. The framework described by Stangl and colleagues may provide a useful starting point [44].

In this national online study, financial, ethical and practical considerations made the collection of bio-markers of risky sexual behaviour infeasible, forcing a reliance on self-report measures alone. Nevertheless, if a game is widely used nationally, other local measures (e.g., of condom sales; STI rates) tied to participant zip codes might provide alternative, inexpensive methods for assessing condom use.

The study is also limited in that participants may have been reluctant to self-report feelings of shame [9], reducing our ability to detect shame reduction. It should be noted that the degree of shame reported by participants was relatively low, corresponding to the anchor labelled “a little.” We are currently investigating possible non self-report methods of shame that could be gathered unobtrusively during a game promising better predictability [14].

### Generalizability

SOLVE's process of game development is designed to enhance generalizability. First, the content was based on several qualitative and quantitative pilot studies that allowed us to understand and map common story arcs, obstacles to safe sex and personal preferences. We sampled MSM across the United States, allowing urban and rural geographic representation. Input from several population-matched community advisory boards throughout informed the design process. Despite successfully recruiting and retaining Black and Latino MSM online, larger sample sizes would afford more granular within-group analyses. Finally, this trial was conducted exclusively online, supporting the feasibility of rapid dissemination and evaluation of serious games (and more traditional interventions) targeting diverse, hard-to-reach high-risk populations.

## Conclusions

Overall, these RCT findings indicate that a game intervention can reduce shame and suggest that such reductions are diagnostic of future reductions in sexual risk-taking for young adult MSM. In ongoing research, we are addressing whether reduction in shame predicts reduction in UAI at longer time intervals (i.e., six months). We also plan to examine which of these components alone or in combination might reduce shame for MSM using more sensitive measures (e.g., neural signals). Another goal for future research is to examine whether a SOLVE game approach can be effectively generalized to other target groups and other risk-reduction efforts. Although technology-based interventions tested and disseminated over the web are promising, understanding how to better adapt these tools for limited resource settings and better overcome the technical challenges posed is critical.

## References

[1] Prejean J, Song R, Hernandez A, Ziebell R, Green T, Walker F (2011). Estimated HIV incidence in the United States, 2006–2009. PLoS One.

[2] Sullivan PS, Carballo-Diéguez A, Coates T, Goodreau SM, McGowan I, Sanders EJ (2012). Successes and challenges of HIV prevention in men who have sex with men. Lancet.

[3] Halkitis PN (2012). Discrimination and homophobia fuel the HIV epidemic in gay and bisexual men. Psychology and AIDS Exchange Newsletter.

[4] Herek G (2007). Confronting sexual stigma and prejudice: theory and practice. J Soc Issues.

[5] Lewis M, Gilbert P, Andrews B (1999). Shame and stigma. Shame: interpersonal behavior, psychopathology, and culture.

[6] Herek GM (2004). Beyond “homophobia”: thinking about sexual prejudice and stigma in the twenty-first century. Sex Res Soc Policy.

[7] Fortenberry JD, McFarlane M, Bleakley A, Bull S, Fishbein M, Grimley DM (2002). Relationships of stigma and shame to gonorrhea and HIV screening. Am J Public Health.

[8] Witte K (1992). Putting the fear back into fear appeals: the extended parallel process model. Commun Monogr.

[9] Tangney JP, Stuewig J, Mashek DJ (2007). Moral emotions and moral behavior. Ann Rev Psychol.

[10] Tracy JL, Robins RW (2004). Putting the self into self-conscious emotions: a theoretical model. Psychol Inq.

[11] Tracy JL, Robins RW (2006). Appraisal antecedents of shame and guilt: support for a theoretical model. Pers Soc Psychol Bull.

[12] Niedenthal PM, Tangney JP, Gavansky I (1994). “If only I weren't” versus “If only I hadn't”: distinguishing shame and guilt in counterfactual thinking. J Pers Soc Psychol.

[13] Wagner U, N'Diaye K, Ethofer T, Vuilleumier P (2011). Guilt-specific processing in the prefrontal cortex. Cereb Cortex.

[14] Randles D, Tracy JL (2013). Nonverbal displays of shame predict relapse and declining health in recovering alcoholics. Clin Psychol Sci.

[15] Newcomb ME, Mustanski B (2011). Moderators of the relationship between internalized homophobia and risky sexual behavior in men who have sex with men: a meta-analysis. Arch Sex Behav.

[16] Fourie MM, Rauch HGL, Morgan BE, Ellis GFR, Jordaan ER, Thomas KGF (2011). Guilt and pride are heartfelt, but not equally so. Psychophysiology.

[17] Damasio AR (1993). Descartes’ error: emotion, reason, and the human brain.

[18] Bechara A, Damasio H, Tranel D, Damasio AR (1997). Deciding advantageously before knowing the advantageous strategy. Science.

[19] Appleby PR, Godoy C, Miller LC, Read SJ, Edgar T, Noar SM, Freimuth VS (2008). Reducing risky sex through the use of interactive video technology. Communication perspectives on HIV/AIDS for the 21st century.

[20] Miller LC, Read SJ (2005). Virtual sex: creating environments for reducing risky sex.

[21] Read SJ, Miller LC, Appleby PR, Nwosu ME, Reynaldo S, Lauren A (2006). Socially optimized learning in a virtual environment: reducing risky sexual behavior among men who have sex with men. Hum Commun Res.

[22] Miller LC, Appleby PR, Christensen JL, Godoy CG, Si M, Corsbie-Massay C, Noar SM, Harrington NG (2012). Virtual interactive interventions for reducing risky sex: adaptations, integrations, and innovations. eHealth applications: promising strategies for health behavior change.

[23] Miller LC, Marsella S, Dey T, Appleby PR, Christensen JL, Klatt J (2011). Socially optimized learning in virtual environments (SOLVE). Interactive Storytelling. Lecture Notes in Computer Science.

[24] Miller LC, Christensen JL, Appleby PR, Read SJ, Godoy CG, Corsbie-Massay C (2012). Socially optimized learning in virtual environments: RCT evaluating MSM HIV risk-reduction.

[25] Ajzen I, Lange PAM, Kruglanski AW, Higgins ET (2012). The theory of planned behavior. Handbook of theories of social psychology.

[26] Bandura A, DiClemente RJ, Peterson JL (1994). Social cognitive theory and the exercise of control over HIV infection. Preventing AIDS: theories and methods of behavioral interventions.

[27] Watson D, Clark LA (1994). The PANAS-X manual for the positive and negative affect schedule – expanded eorm.

[28] Cronbach LJ, Furby L (1970). How we should measure “change”: or should we?. Psychol Bull.

[29] MacKinnon DP (2012). Introduction to statistical mediation analysis.

[30] Muthén LK, Muthén BO (1998–2012). Mplus user's guide.

[31] Schulz KF, Altman DG, Moher D, for the CONSORT Group (2010). CONSORT 2010 statement: updated guidelines for reporting parallel group randomised trials. Ann Int Med.

[32] Moher D, Hopewell S, Schulz KF, Montori V, Gøtzsche PC, Devereaux PJ (2010). CONSORT 2010 Explanation and Elaboration: updated guidelines for reporting parallel group randomised trial. BMJ.

[33] Hayes AF (2009). Beyond Baron and Kenny: statistical mediation analysis in the new millennium. Commun Monogr.

[34] Luoma JB, Kohlenberg BS, Hayes SC, Fletcher L (2012). Slow and steady wins the race: a randomized clinical trial of acceptance and commitment therapy targeting shame in substance use disorders. J Consult Clin Psychol.

[35] Sengupta S, Banks B, Jonas D, Miles MS, Smith GC (2011). HIV interventions to reduce HIV/AIDS stigma: a systematic review. AIDS Behav.

[36] Pachankis JE, Goldfried MR (2010). Expressive writing for gay-related stress: psychosocial benefits and mechanisms underlying improvement. J Consult Clin Psychol.

[37] Mustanski B, Garofalo R, Monahan C, Gratzer B, Andrews R (2013). Feasibility, acceptability, and preliminary efficacy of an online HIV prevention program for diverse young men who have sex with men: the keep it up! Intervention. AIDS and Behavior.

[38] Fox J, Bailenson J, Binney J (2009). Virtual experiences, physical behaviors: the effect of presence on imitation of an eating avatar. Presence.

[39] Centers for Disease Control and Prevention (2010). HIV risk reduction efficacy review: efficacy criteria. http://www.cdc.gov/hiv/dhap/prb/prs/efficacy/rr/criteria/index.html.

[40] Bull SS, Lloyd L, Rietmeijer C, McFarlane M (2004). Recruitment and retention of an online sample for an HIV prevention intervention targeting men who have sex with men: the smart sex quest project. AIDS Care.

[41] Khosropour CM, Sullivan PS (2011). Predictors of retention in an online follow-up study of men who have sex with men. J Med Internet Res.

[42] Chiasson MA, Shaw FS, Humberstone M, Hirshfield S, Hartel D (2009). Increased HIV disclosure three months after an online video intervention for men who have sex with men (MSM). AIDS Care.

[43] Rosser B, Oakes J, Konstan J, Hooper H, Horvath KJ, Danilenko GP (2010). Reducing HIV risk behavior of MSM through persuasive computing: results of the men's INTernet study (MINTS-II). AIDS.

[44] Stangl A, Go V, Zelaya C, Brady L, Nyblade L, Stackpool-Moore L (2010). Enabling the scale-up of efforts to reduce HIV stigma and discrimination: a new framework to inform program implementation and measurement. Poster presented at: XVIII International AIDS Conference.

# Sub article: Sexual stigma and discrimination as barriers to seeking appropriate healthcare among men who have sex with men in Swaziland

**DOI:** 10.7448/IAS.16.3.18715

**Published:** 2013-11-13

**Authors:** Kathryn Risher, Darrin Adams, Bhekie Sithole, Sosthenes Ketende, Caitlin Kennedy, Zandile Mnisi, Xolile Mabusa, Stefan D Baral

**Affiliations:** 1 Department of Epidemiology, Johns Hopkins Bloomberg School of Public Health, Baltimore, MD, USA; 2 Rock of Hope, Mbabane, Swaziland; 3 Department of International Health, Johns Hopkins Bloomberg School of Public Health, Baltimore, MD, USA; 4 Swaziland National AIDS Program, Mbabane, Swaziland

**Keywords:** sexual stigma, MSM, disclosure, structural HIV prevention, combination HIV prevention

## Abstract

**Introduction:**

Same-sex practices and orientation are both stigmatized and criminalized in many countries across sub-Saharan Africa. This study aimed to assess the relationship of fear of seeking healthcare and disclosure of same-sex practices among a sample of men who have sex with men (MSM) in Swaziland with demographic, socio-economic and behavioural determinants.

**Methods:**

Three hundred and twenty-three men who reported having had anal sex with a man in the past year were recruited using respondent-driven sampling and administered a structured survey instrument. Asymptotically unbiased estimates of prevalence of stigma and human rights abuses generated using the RDSII estimator are reported with bootstrapped confidence intervals (CIs). Weighted simple and multiple logistic regressions of fear of seeking healthcare and disclosure of same-sex practices to a healthcare provider with demographic, social and behavioural variables are reported.

**Results:**

Stigma was common, including 61.7% (95% CI=54.0–69.0%) reporting fear of seeking healthcare, 44.1% (95% CI=36.2–51.3%) any enacted stigma and 73.9% (95% CI=67.7–80.1%) any perceived social stigma (family, friends). Ever disclosing sexual practices with other men to healthcare providers was low (25.6%, 95% CI=19.2–32.1%). In multiple logistic regression, fear of seeking healthcare was significantly associated with: having experienced legal discrimination as a result of sexual orientation or practice (aOR=1.9, 95% CI=1.1–3.4), having felt like you wanted to end your life (aOR=2.0, 95% CI=1.2–3.4), having been raped (aOR=11.0, 95% CI=1.4–84.4), finding it very difficult to insist on condom use when a male partner does not want to use a condom (aOR=2.1, 95% CI=1.0–4.1) and having a non-Swazi nationality at birth (aOR=0.18, 95% CI=0.05–0.68). In multiple logistic regression, disclosure of same-sex practices to a healthcare provider was significantly associated with: having completed secondary education or more (aOR=5.1, 95% CI=2.5–10.3), having used a condom with last casual male sexual partner (aOR=2.4, 95% CI=1.0–5.7) and having felt like you wanted to end your life (aOR=2.1, 95% CI=1.2–3.8).

**Conclusions:**

MSM in Swaziland report high levels of stigma and discrimination. The observed associations can inform structural interventions to increase healthcare seeking and disclosure of sexual practices to healthcare workers, facilitating enhanced behavioural and biomedical HIV-prevention approaches among MSM in Swaziland.

## Introduction

Consistent data highlight the central role of stigma in limiting uptake of HIV prevention, treatment and care services [1–3]. This is especially true among men who have sex with men (MSM), who are at elevated risk of HIV acquisition and transmission, live outside of broad social expectations for gender roles, and therefore often experience homoprejudice [4]. The institutionalization of heterosexual norms, or heteronormativity, results in MSM being ignored or discriminated against by laws, individuals and societies [5–7]. Stigma has been defined as the social devaluation of a person based on an attribute [8], and discrimination, as behaviour resulting from prejudice [9]. Sexual stigma, commonly defined as a shared belief system that denigrates and discredits homosexuality with respect to heterosexuality [10], affects the lives of gay men and other MSM. Researchers have traditionally divided stigma into enacted and perceived, or felt, stigma [1]. Enacted stigma refers to a discrimination event based on the attribute that is ascribed to the stigmatized group [9, 11]. Perceived stigma, conversely, has been described as the shame associated with the stigmatized attribute and the “*fear* of enacted stigma,” including awareness that the attribute is stigmatized [9, 11, 12].

In 38 countries across sub-Saharan Africa, MSM not only experience stigma but also same-sex practices are criminalized [13]. In Swaziland, sodomy, defined as male–male anal sex, is illegal [13]. Many leaders in sub-Saharan Africa have made public claims that homosexuality is “un-African” [14], though researchers have found evidence of a long history of homosexual acts in sub-Saharan Africa suggesting that anti-sodomy laws have colonial origins [15–18]. Respondents in quantitative studies of stigma among MSM in Southern Africa report high levels of stigma regardless of country of residence. Among MSM in Malawi, Botswana, and Namibia, 23.5% of participants reported experiencing some form of discrimination [19]. Among MSM in Lesotho and South Africa, 76.2% [20] and 24.5% [21], respectively, reported at least one human rights abuse due to their sexual practices.

Broadly, stigma has been associated with the physical and mental health of MSM across contexts. Studies from Malawi, Botswana and Namibia have demonstrated that MSM who had any interaction with healthcare had over two times greater odds of experiencing fear of seeking healthcare and over six times greater odds of having been denied healthcare due to sexual orientation [19]. Moreover, studies in other sub-Saharan African countries have found HIV status associated with four times increased odds of blackmail due to same-sex practices [21], and disclosure to healthcare workers associated with nearly four times or family members with nearly three times increased odds of blackmail [20]. In a respondent-driven sample of MSM from Uganda, ever reporting homophobic abuse was associated with over five times greater odds of HIV infection [22]. In qualitative research, MSM in South Africa reported verbal discrimination by healthcare workers, non-disclosure by bisexually identified MSM and travelling long distances to seek appropriate care [23, 24]. Similar findings have been reported across sub-Saharan Africa [25, 26].

These studies highlight that experiencing stigma often results in stigma management, including modified behaviours and coping mechanisms to avoid enactments of stigma, which can often be disruptive and lead to distress [10]. In addition, when the stigmatized attribute is concealable (as same-sex orientation and practices are), non-disclosure of same-sex orientation or practices is a potential stigma management technique with associated stress of concealment [8]. Disclosure of same-sex orientation and practices may result in negative outcomes ranging from social isolation to physical attack [27], and therefore an individual chooses how to manage the information on their sexual orientation or practices [28]. Additionally, the minority stress model proposes that stress experienced by minority groups is greater than stress experienced by the general population and is therefore unique, chronic and based on social processes outside of the individual [29, 30]. The minority stress model provides a clear link between stigma and mental health for sexual minorities, including MSM [29, 30].

HIV prevalence among adults aged 15–49 in Swaziland is estimated to be 25.9%, among the highest worldwide [31]. Given that Swaziland's highly generalized epidemic is known to disproportionately affect women [31], there has been limited evaluation of the HIV burden and determinants of HIV infection among MSM. However, in other settings in Southern Africa, HIV has been shown to be concentrated among MSM, particularly when compared to other men, given the region's primarily female-predominant HIV epidemics [20, 21, 32–36].

Given that MSM in Swaziland are understudied and live in a setting of legal discrimination, we aimed to assess the prevalence of sexual stigma and discrimination among MSM in Swaziland in late 2011. We also sought to examine the associations of demographic, social and behavioural variables with fear of seeking healthcare and disclosure of same-sex practices to a healthcare provider. Enhancing the understanding of these associations will support the development of targeted and effective combination HIV-prevention strategies that include mitigating stigma as well as novel biomedical approaches and established behavioural interventions for MSM in Swaziland [37].

## Methods

### Study population

Participants eligible for this study were men at least 18 years of age who were able to provide informed consent in either English or siSwati, reported receptive or insertive anal intercourse with another man in the past 12 months, and presented a valid recruitment coupon or were selected as a seed as part of respondent-driven sampling (RDS) (methodology described below). Exclusion criteria included having been born biologically female or previous participation in the current survey.

### Sampling and recruitment

RDS was used to recruit study participants from July to December 2011. RDS is a form of chain-referral sampling developed to recruit participants from hidden populations for whom it is infeasible to generate a sampling frame [38]. RDS starts with an initial sample from the population, referred to as seeds, which are selected in a non-random manner. Seeds are given a set number of coupons with which to recruit peers, and are given small financial reimbursement for participation and recruitment. Additional waves of recruits are offered the same incentives and asked to recruit with a set number of coupons. RDS generates asymptotically unbiased estimates independent of the initial seeds [39]. By asking a participant to identify his/her network size and giving a set number of coupons to each participant, RDS allows calculation of population proportions. MSM in Swaziland are hard-to-reach and legally discriminated against, making RDS an appropriate method for recruitment.

Three seeds were chosen at study onset to begin recruitment. Seeds were chosen based on social connection and status within the MSM community, ability to articulate study goals, motivation and inclusion criteria. Seeds were intended to be diverse in socio-demographic and behavioural characteristics, sub-group membership and sexual practices. Seeds and subsequent respondents were given three coupons that expired four weeks from the date of study visit. An additional eight seeds were added when recruitment slowed.

### Sample size calculation

Sample size was calculated based on national estimated HIV prevalence among reproductive-age men in Swaziland in 2007 [31], because there was no previous estimate of HIV prevalence among MSM in Swaziland. Based on this prevalence, a sample size of 324 was required to detect significant differences (OR=2.0) in HIV prevalence based on condom use during sex with men (always use compared to less than always) with 95% confidence, 80% power and a design effect of 1.5. This method allowed for testing for differences between groups based on social factors such as experienced stigma and discrimination.

### Study procedure and survey instrument

Each participant completed an in-person interview with a trained local research staff member in a private office setting lasting approximately one hour. The instrument included modules on socio-demographics, sexual orientation, behavioural HIV-related risk factors (HIV-related knowledge, attitudes, and risk behaviours, including condom negotiation), stigma and discrimination, and social cohesion. Questions on sexual stigma were dichotomous and included perceived stigma and enacted stigma all in relation to sexual orientation or practice. We report “any enacted stigma” (lost employment, denied education, arrested on false charges, or beaten up) and “any perceived social stigma” (having felt exclusion from family gatherings, felt family members made discriminatory remarks or felt rejection by friends) as responding “yes” to any of the dichotomous questions in each respective category. Testing and counselling for HIV and syphilis were also conducted, and results and procedures are reported elsewhere [40].

Verbal informed consent was obtained for this anonymous study. No names or identifying information were collected to ensure anonymity and safety of participants. Individuals received primary reimbursement for travel costs and a meal and secondary reimbursement for travel and a set amount per eligible participant accrued with their coupons.

### Statistical analysis

To estimate asymptotically unbiased prevalence of demographic, social and stigma variables, the RDSII estimator was used to assess a sampling weight for each variable using collected non-missing data [41]. Asymptotically unbiased estimates were generated using these weights, which adjusted for an individual's level of homophily (the extent to which participants recruit individuals who are similar to themselves) and degree (personal network size) [42]. Bootstrapping was utilized to calculate all population prevalence confidence intervals (CIs) using 1000 replicates [43]. Non-seed individuals with network size zero were excluded from all analyses as these individuals violate the RDS assumption of reciprocal relationships [44]. Network size for weighting was characterized by the number of MSM the participant knew and had seen or spoken with in the past year. Denominators for individual questions differ because participants were free to refuse response to any question. Crude results and weighted percentages with CIs are presented.

There is currently limited consensus in RDS literature regarding how to handle regression analyses of RDS data [41, 45, 46]. Here, sensitivity analyses were completed with and without sampling weights for dependent variables [41, 46]. Specifically, to assess associations between stigma outcome variables (fear of seeking healthcare due to sexual orientation or practice, and disclosure of sexual practice to a healthcare provider) and social, demographic and behavioural variables, simple and multiple logistic regressions were conducted with and without the outcome variable's population weight. Potential predictors were chosen for assessment based on associations with sexual stigma found in previous literature and guided by the modified social ecological model [47]. After controlling for potential confounders (age, education and sexual orientation), independent variables were chosen for inclusion in multiple logistic regressions based on simple logistic regression coefficients with a *p*-value less than 0.05. Weighted results are reported for simple (odds ratios, ORs) and multiple logistic regressions (adjusted odds ratios, aORs). Furthermore, sensitivity analyses were completed including and excluding the seeds used for recruitment initiation and propagation for multiple logistic regression models [48]. Results including seeds are reported due to negligible difference between models. Missing data were assessed to be less than 5% for each variable, and therefore ignorable.

All statistical analyses were conducted using STATA 12.0 (College Station, TX).

### Ethical review

The National Ethics Committee of Swaziland and the Institutional Review Board of the Johns Hopkins Bloomberg School of Public Health approved this study for human subjects research.

## Results

Overall, 323 men were recruited and consented to participate in the study. Table 1 shows respondents’ socio-demographic characteristics. Table 2 shows the prevalence of stigma and discrimination. A large proportion of respondents reported fear of seeking healthcare as a result of sexual orientation or practice (61.7%, 95% CI=54.0–69.0%, *n*=179/320). A minority of participants reported having disclosed sexual practices with other men to a healthcare provider (25.6%, 95% CI=19.2–32.1%, 101/323). Almost three-quarters of participants had experienced any perceived social stigma, and 44.1% (95% CI=36.2–51.3%, 149/323) of participants reported any enacted stigma.

**Table 1:** Socio-demographic characteristics of MSM in Swaziland

	Crude %	Population adjusted %	
		
Characteristic	(*n*/*N*)	(95% bootstrapped confidence interval)	Homophily
Age			
Mean/median	23.1/22	–	–
<21	30.0 (97/323)	35.1 (26.9–43.9)	0.23
21–29	60.7 (196/323)	57.7 (49.0–66.0)	0.28
>=30	9.3 (30/323)	7.2 (3.9–11.3)	0.06
Education			
Less than secondary completion	34.7 (112/323)	44.2 (35.5–53.5)	0.13
Completed secondary or more	65.3 (211/323)	55.9 (46.5–64.5)	0.37
Employment			
Unemployed	31.9 (99/310)	30.9 (23.9–39.3)	0.18
Employed	34.2 (106/310)	27.0 (20.0–34.2)	0.20
Student	33.9 (105/310)	42.1 (34.1–50.4)	−0.01
Area grew up in:			
Urban	63.5 (198/312)	63.3 (54.8–71.0)	0.15
Rural	36.5 (114/312)	36.7 (29.0–45.2)	0.19
Country of origin			
Swazi	96.0 (308/321)	97.7 (95.8–99.1)	−0.77
Not Swazi	4.1 (13/321)	2.3 (0.90–4.2)	0.07
Income (in SZL, last month)			
Mean/median	2930/780	–	–
No income	31.2 (100/321)	36.8 (28.5–44.8)	−0.05
Any income	68.9 (221/321)	63.2 (55.2–71.5)	0.21
Sexual orientation			
Gay or homosexual	63.4 (204/322)	57.2 (48.8–65.1)	0.24
Bisexual	34.8 (112/322)	39.7 (31.5–48.0)	0.06
Straight or heterosexual	1.6 (5/322)	3.1 (0.29–7.5)	0.08
Region where currently stay			
Hhohho	14.2 (46/323)	12.3 (6.8–19.1)	0.15
Manzini	61.0 (197/323)	57.0 (45.5–67.5)	0.39
Shiselweni	6.2 (20/323)	6.5 (2.9–12.0)	0.08
Lubombo	18.3 (59/323)	24.3 (15.6–33.4)	0.29
Number of MSM seen or talked to in the past 6 months, mean/median (range), of known MSM	13.8/7 (1–400)	–	–
Married/cohabitating	3.4 (11/321)	1.7 (0.47–3.6)	−0.02
Have children	12.1 (39/322) Range (0–5)	10.2 (6.4–14.8)	0.10

MSM, men who have sex with men.

**Table 2:** Prevalence of stigma and discrimination among MSM in Swaziland

	Crude %	Population adjusted %
	
Characteristic	(*n*/*N*)	(95% bootstrapped confidence interval)
Fear of seeking healthcare as a result of sexual orientation or practice	55.9 (179/320)	61.7 (54.0–69.0)
Felt afraid to walk around in public places as a result of your sexual orientation or practice	45.7 (147/322)	44.4 (37.1–51.4)
Any perceived social stigma (family, friends)	76.2 (246/323)	73.9 (67.7–80.1)
Felt that you received lower quality healthcare services as a result of your sexual orientation or practice	16.7 (54/323)	19.0 (13.3–25.6)
Denied health services as a result of sexual orientation or practice	3.7 (12/322)	3.0 (1.1–5.4)
Ever been beaten up as a result of sexual orientation or practice	9.0 (29/323)	8.6 (4.5–13.6)
Lost employment as a result of your sexual orientation or practice	2.8 (9/322)	3.7 (1.1–6.7)
Denied educational opportunities as a result of sexual orientation or practice	5.3 (17/323)	3.4 (1.7–5.7)
Arrested on false charges because of your sexual orientation or practice	4.6 (15/323)	3.2 (1.5–5.5)
Any enacted stigma	46.1 (149/323)	44.1 (36.2–51.3)

MSM, men who have sex with men.

There was a high prevalence of depressive symptoms and self-reported suicidal ideation, with 58.3% (95% CI=51.2–65.4%, *n*=207/323) reporting feeling sad or depressed for over two weeks in the past three years and 36.8% (95% CI=29.3–44.0%, *n*=140/322) reporting having ever felt like they wanted to end their lives. Nineteen participants, 6.0% of the sample (95% CI=2.9–9.6%, *n*=19/314), reported having ever been raped. Forty participants (13.0%; 95% CI=8.4–18.2%, *n*=40/323, homophily=0.156) had been to jail or prison.

### Associations with fear of seeking healthcare

Table 3 shows simple and multiple logistic regressions of fear to seek healthcare due to sexual orientation or practice on independent variables. Significant bivariate associations with fear to seek healthcare included: having experienced legal discrimination as a result of sexual orientation or practice (OR=2.2, 95% CI=1.3–3.6), having felt like you wanted to end your life (OR=2.4, 95% CI=1.5–3.8), having ever been raped (OR=7.3, 95% CI=1.7–32.5), finding it very difficult to insist on condom use when a male partner does not want to use a condom (OR=2.8, 95% CI=1.6–4.9), any unprotected anal sex in the past 12 months (OR=2.0, 95% CI=1.2–3.1), having been denied healthcare (OR=8.3, 95% CI=1.0–66.6), and lower odds associated with having a non-Swazi nationality at birth (OR=0.23, 95% CI=0.06–0.84).

**Table 3:** Associations with fear of seeking healthcare among MSM in Swaziland

	Fear to seek healthcare (*N*=320)					
					
Variable	Yes *n* (%)	No *n* (%)	OR (95% confidence interval)	*p*	aOR* (95% confidence interval)	*p*
Disclosure to a healthcare worker	61 (60.4)	40 (39.6)	1.3 (0.81–2.1)	0.277	–	–
Having experienced legal discrimination as a result of sexual orientation or practice	69 (68.3)	32 (31.7)	2.2 (1.3–3.6)	0.003	1.9 (1.1–3.4)	0.026
Having felt like you wanted to end your life	93 (67.9)	44 (32.1)	2.4 (1.5–3.8)	<0.001	2.0 (1.2–3.4)	0.013
Having been raped	17 (89.5)	2 (10.5)	7.3 (1.7–32.5)	0.009	11.0 (1.4–84.4)	0.022
Finding it very difficult to insist on condom use when male partner does no't want to use	60 (73.2)	22 (26.8)	2.8 (1.6–4.9)	<0.001	2.1 (1.0–4.1)	0.039
Any unprotected anal sex in the past 12 months	101 (63.5)	58 (36.5)	2.0 (1.2–3.1)	0.004	0.97 (0.54–1.7)	0.929
HIV counselling and testing						
Not tested for HIV in the past 12 months	86 (58.9)	60 (41.1)	REF	REF	–	–
Tested for HIV one time in the past 12 months	58 (59.2)	40 (40.8)	1.0 (0.60–1.7)	0.965	–	–
Tested for HIV two or more times in the past 12 months	35 (46.1)	41 (54.0)	0.60 (0.34–1.0)	0.07	–	–
Non-Swazi nationality at birth	3 (23.1)	10 (76.9)	0.23 (0.06–0.84)	0.027	0.18 (0.05–0.68)	0.012
Self-reported HIV-positive test	12 (66.7)	6 (33.3)	1.6 (0.60–4.5)	0.338	–	–
HIV seropositive (test on interview date)	28 (51.9)	26 (48.2)	0.83 (0.46–1.5)	0.537	–	–
Denied healthcare	10 (90.9)	1 (9.1)	8.3 (1.0–66.6)	0.046	4.4 (0.53–36.5)	0.170

* The final model also included categorical variables for age, education and sexual orientation.

MSM, men who have sex with men.

In multiple logistic regression, having experienced legal discrimination as a result of sexual orientation or practice (aOR=1.9, 95% CI=1.1–3.4), having felt like you wanted to end your life (aOR=2.0, 95% CI=1.2–3.4), having been raped (aOR=11.0, 95% CI=1.4–84.4), finding it very difficult to insist on condom use when a male partner doesn't want to use a condom (aOR=2.1, 95% CI=1.0–4.1), and having a non-Swazi nationality at birth (aOR=0.18, 95% CI=0.05–0.68), were statistically significantly associated with fear of seeking healthcare as a result of sexual orientation or practice.

### Associations with disclosure of sexual practices to healthcare provider

Significant bivariate associations with disclosing same-sex practices to a healthcare worker included: being 25 or older (OR=1.7, 95% CI=1.0–2.8), having completed secondary education or more (OR=3.7, 95% CI=2.1–6.7), being employed (OR=1.9, 95% CI=1.0–3.4), having been tested for a sexually transmitted infection (STI) in the past 12 months (OR=2.6, 95% CI=1.3–5.1), having used a condom with last casual male partner (OR=2.3, 95% CI=1.1–4.7), having felt like you wanted to end your life (OR=2.1, 95% CI=1.3–3.4), having disclosed to a family member (OR=2.1, 95% CI=1.3–3.5), having participated in any talks or meetings related to HIV and AIDS with other MSM (OR=1.8, 95% CI=1.1–3.2), and feeling that there is a place for MSM to socialize (OR=1.7, 95% CI=1.0–2.7). Having been denied healthcare services as a result of sexual orientation or practices was close to being statistically significantly associated with disclosure to a healthcare worker (OR=3.2, 95% CI=0.99–10.5).

In multiple logistic regression, having completed secondary education or more (aOR=5.1, 95% CI=2.5–10.3), having used a condom with last casual male sexual partner (aOR=2.4, 95% CI=1.0–5.7) and having felt like you wanted to end your life (aOR=2.1, 95% CI=1.2–3.8) were statistically significantly associated with having disclosed sexual orientation or practice to a healthcare provider. All other variables from significant bivariate associations were included in the model but were not statistically significant after adjustment.

## Discussion

This study is the first assessment of sexual stigma among MSM in Swaziland. We identified adjusted associations with fear of seeking healthcare as a result of same-sex orientation or practice and with disclosure of same-sex practices to a healthcare provider. This study also described the prevalence of stigma and discrimination among MSM in Swaziland.

The high level of fear of seeking healthcare in this sample, reported by over half of the respondents, suggests that MSM in Swaziland may not be seeking care that is important to their health and wellbeing. Fear of seeking healthcare due to same-sex practices or orientation is an example of perceived stigma and choosing not to seek healthcare may be a coping mechanism to avoid enacted stigma including the denial of care [49]. Ultimately, reduced healthcare seeking practices impede the provision of appropriate care.

Disclosure to healthcare providers was low in this sample, only reported by a quarter of respondents, which suggests that MSM in Swaziland who do seek care are not receiving appropriate services. Disclosure of sexual orientation or practices to a healthcare provider is an important step in the provision of appropriate healthcare for MSM. For example, evidence-based healthcare for MSM includes anal pap smears to detect rectal cancers and testing for anal STIs [50, 51]. MSM should also receive targeted safe sex counselling, particularly on the use of water-based lubricant with condoms [52], and same-sex couple-based HIV counselling and testing if desired.

These data also emphasize the need for availability of referrals to mental healthcare when MSM seek care [51]. The prevalence of depressive symptoms and suicidal ideation were high in this sample, with over a half and a third reporting each, respectively. Moreover, suicidal ideation was a strong predictor both of fear of seeking healthcare and of having disclosed same-sex practices to a healthcare provider. Poor mental health has been associated with sexual stigma and stress elsewhere [49], and this study supports these findings in Swaziland. Given that MSM experiencing suicidal ideation may be seeking treatment and disclosing sexual practices at that time, healthcare sensitization to guide an appropriate response at time of crisis may be part of larger combination interventions to decrease fear of seeking healthcare.

Fear of seeking healthcare was positively associated with having experienced legal discrimination as a result of sexual orientation or practice and having been raped, two forms of rights abuses. Thus, individuals who have been disempowered in other contexts appear to experience greater perceived stigma in healthcare settings. Additionally, fear of seeking healthcare was positively associated with finding it very difficult to insist on condom use with partners who do not want to use them. Individuals who reported feeling less power in sexual negotiation also reported greater perceived healthcare stigma. In accordance with perceived stigma resulting in coping mechanisms [10], these findings suggest that those individuals who have less social capital are also less likely to seek healthcare. This association can inform combination HIV-prevention approaches among Swazi MSM by emphasizing the need for structural interventions that empower MSM. In addition, fear of seeking healthcare was negatively associated with non-Swazi nationality at birth, despite small sample size. Potentially, individuals born in surrounding South Africa, where sexual minorities have constitutional protection, may seek care in more tolerant facilities in South Africa where denial of care reported by MSM has been low [21]. This study, however, did not assess where individuals were seeking care and further research is necessary to better characterize this association.

Disclosure of same-sex practices to a healthcare provider was strongly associated with increased education, and indicates that MSM in Swaziland with the most education are potentially receiving more competent care. This finding suggests that beyond health inequity between the general population and MSM established elsewhere [53, 54], there is additional inequity between more and less empowered MSM. These results indicate the need for healthcare provider sensitization and training as part of structural HIV-prevention strategies, which have been implemented in other settings where MSM are highly stigmatized [55–58]. In addition, MSM who reported using a condom with last casual male partner were more likely to have disclosed information to a healthcare provider. This may indicate that MSM who disclose are receiving appropriate care including counselling on condom use, similar to studies in other contexts which have found disclosure to be associated with HIV protective behaviours [59, 60]. Conversely, it may indicate that individuals at the greatest risk for acquiring anal STIs are not comfortable disclosing and therefore are not receiving appropriate care. In either scenario, these data highlight the need for health sector interventions to include training on taking sexual histories including non-heteronormative questions about sexuality as well as preparing the provider to respond sensitively to a person's disclosure.

There were a number of limitations to the scope of this study. This study uses cross-sectional data, which precludes any statements about causality, temporality or directionality of associations. The behavioural data collected by interviewer-administered surveys were likely skewed by social desirability bias, despite efforts to ensure strict confidentiality and interviewer training. The study was powered based on HIV prevalence, not stigma outcomes, which may have resulted in type II error. Additionally, the study was not powered to identify differences in very rare outcomes, such as rape and having been denied healthcare (each with a prevalence in this sample of below 7%). Thus, conclusions about these variables have a high level of uncertainty. This study only evaluated reported depressive symptoms rather than a validated depression screen such as the CES-D [61], Hopkins Symptom Checklist [62] and Beck Depression Inventory [63]. Future studies should utilize a validated scale to facilitate a better understanding of the burden and associations of mental health among MSM in Swaziland. Finally, RDS makes a number of assumptions [39] about network structure, which may be violated in the network of MSM in Swaziland. If these assumptions were violated, our results are not generalizable to the wider MSM network of Swaziland, and even if these assumptions were met, generalizability to populations outside of Swaziland is limited. Despite these limitations, this is the first study of MSM in Swaziland and it builds a strong foundation for further research, and intervention development and testing with MSM in the country.

## Conclusions

This study suggests the importance of incorporating structural stigma-reduction intervention strategies into combination HIV prevention among MSM in Swaziland. MSM are not currently included in HIV-prevention programming in Swaziland, though the National HIV and AIDS Strategic Framework identifies MSM as a group for whom insufficient data have been collected [64]. While the provision of biomedical and behavioural interventions to reduce HIV transmission is necessary, it is insufficient to increase coverage of services. Interventions focused on increasing uptake of targeted interventions by increasing healthcare seeking and disclosures of same-sex practices are equally crucial to increase the coverage of prevention programmes. These results emphasize the importance of structural interventions to reduce HIV and sexual stigma and discrimination, such as healthcare provider sensitization, the inclusion of MSM in national HIV strategies, increased provision of appropriate care, improved social capital and community capacity building [65]. Comprehensive anti-stigma approaches which engage communities, healthcare providers, governments, researchers and more are needed to generate a space in which it is safe to access healthcare and disclose to a healthcare provider for MSM in Swaziland [66].

## References

[1] Logie C, Gadalla TM (2009). Meta-analysis of health and demographic correlates of stigma towards people living with HIV. AIDS Care.

[2] Mahajan AP, Sayles JN, Patel VA, Remien RH, Sawires SR, Ortiz DJ (2008). Stigma in the HIV/AIDS epidemic: a review of the literature and recommendations for the way forward. AIDS.

[3] Nyblade L, Stangl A, Weiss E, Ashburn K (2009). Combating HIV stigma in health care settings: what works?. J Int AIDS Soc.

[4] Altman D, Aggleton P, Williams M, Kong T, Reddy V, Harrad D (2012). Men who have sex with men: stigma and discrimination. Lancet.

[5] Seale A (2009). Heteronormativity and HIV in sub-Saharan Africa. Development.

[6] Warner M (1993). Fear of a queer planet: queer politics and social theory.

[7] Link BG, Phelan JC (2001). Conceptualizing stigma. Annu Rev Sociol.

[8] Goffman E (1963). Stigma; notes on the management of spoiled identity.

[9] Earnshaw VA, Chaudoir SR (2009). From conceptualizing to measuring HIV stigma: a review of HIV stigma mechanism measures. AIDS Behav.

[10] Herek GM, Chopp R, Strohl D, Meyer IH, Northridge ME (2007). Sexual stigma: putting sexual minority health issues in context. The health of sexual minorities: public health perspectives on lesbian, gay, bisexual and transgender population.

[11] Jacoby A (1994). Felt versus enacted stigma: a concept revisited. Evidence from a study of people with epilepsy in remission. Soc Sci Med.

[12] Herek GM (2004). Beyond “Homophobia”: thinking about sexual prejudice and stigma in the twenty-first century. Sex Res Soc Pol.

[13] Itaborahy LP (2012). The International Lesbian, Gay, Bisexual, Trans and Intersex Association (ILGA). State-sponsored homophobia: a world survey of laws criminalising same-sex sexual acts between consenting adults.

[14] Dunton C, Palmberg M (1996). Human rights and homosexuality in Southern Africa.

[15] Hoad NW (2007). African intimacies: race, homosexuality, and globalization.

[16] Murray SO, Roscoe W (1998). Boy-wives and female husbands: studies in African homosexualities.

[17] Epprecht M (2004). Hungochani: the history of a dissident sexuality in southern Africa.

[18] Semugoma P, Nemande S, Baral SD (2012). The irony of homophobia in Africa. Lancet.

[19] Fay H, Baral SD, Trapence G, Motimedi F, Umar E, Iipinge S (2011). Stigma, health care access, and HIV knowledge among men who have sex with men in Malawi, Namibia, and Botswana. AIDS Behav.

[20] Baral S, Adams D, Lebona J, Kaibe B, Letsie P, Tshehlo R (2011). A cross-sectional assessment of population demographics, HIV risks and human rights contexts among men who have sex with men in Lesotho. J Int AIDS Soc.

[21] Baral S, Burrell E, Scheibe A, Brown B, Beyrer C, Bekker LG (2011). HIV risk and associations of HIV infection among men who have sex with men in peri-urban Cape Town, South Africa. BMC Public Health.

[22] Hladik W, Barker J, Ssenkusu JM, Opio A, Tappero JW, Hakim A (2012). HIV infection among men who have sex with men in Kampala, Uganda-a respondent driven sampling survey. PLoS One.

[23] Lane T, Mogale T, Struthers H, McIntyre J, Kegeles SM (2008). “They see you as a different thing”: the experiences of men who have sex with men with healthcare workers in South African township communities. Sex Transm Infect.

[24] Rispel LC, Metcalf CA, Cloete A, Moorman J, Reddy V (2011). You become afraid to tell them that you are gay: health service utilization by men who have sex with men in South African cities. J Public Health Pol.

[25] Sharma A, Bukusi E, Gorbach P, Cohen CR, Muga C, Kwena Z (2008). Sexual identity and risk of HIV/STI among men who have sex with men in Nairobi. Sex Transm Dis.

[26] Poteat T, Diouf D, Drame FM, Ndaw M, Traore C, Dhaliwal M (2011). HIV risk among MSM in Senegal: a qualitative rapid assessment of the impact of enforcing laws that criminalize same sex practices. PLoS One.

[27] Herek GM, Herek GM, Jobe JB, Carney RM (1996). Why tell if you are not asked? Self-disclosure, intergroup contact, and heterosexuals’ attitudes toward lesbians and gay men. Out in force: sexual orientation and the military.

[28] Chrobot-Mason D, Button SB, DiClementi JD (2001). Sexual identity management strategies: an exploration of antecedents and consequences. Sex Roles.

[29] Meyer IH, Meyer IH, Northridge ME (2007). Prejudice and discrimination as social stressors. The health of sexual minorities: public health perspectives on lesbian, gay, bisexual, and transgender populations.

[30] Meyer IH (1995). Minority stress and mental health in gay men. J Health Soc Behav.

[31] Swaziland Central Statistical Office, Macro International Inc (2008). Swaziland demographic and health survey, 2006–07.

[32] Smith AD, Tapsoba P, Peshu N, Sanders EJ, Jaffe HW (2009). Men who have sex with men and HIV/AIDS in sub-Saharan Africa. Lancet.

[33] Rispel LC, Metcalf CA, Cloete A, Reddy V, Lombard C (2011). HIV prevalence and risk practices among men who have sex with men in two South African cities. J Acquir Immune Defic Syndr.

[34] Lane T, Raymond HF, Dladla S, Rasethe J, Struthers H, McFarland W (2011). High HIV prevalence among men who have sex with men in Soweto, South Africa: results from the Soweto Men's Study. AIDS Behav.

[35] Burrell E, Mark D, Grant R, Wood R, Bekker LG (2010). Sexual risk behaviours and HIV-1 prevalence among urban men who have sex with men in Cape Town, South Africa. Sex Health.

[36] Muraguri N, Temmerman M, Geibel S (2012). A decade of research involving men who have sex with men in sub-Saharan Africa: current knowledge and future directions. SAHARA J.

[37] Sullivan PS, Carballo-Dieguez A, Coates T, Goodreau SM, McGowan I, Sanders EJ (2012). Successes and challenges of HIV prevention in men who have sex with men. Lancet.

[38] Heckathorn DD (1997). Respondent-driven sampling: a new approach to the study of hidden populations. Soc Probl.

[39] Salganik MJ, Heckathorn DD (2004). Sampling and estimation in hidden populations using respondent-driven sampling. Socio Meth.

[40] Baral SD, Ketende S, Mnisi Z, Mabuza X, Grosso A, Sithole B (2013). A cross-sectional assessment of the burden of HIV and associated individual and structural level characteristics among men who have sex with men in Swaziland. J Int AIDS Soc.

[41] Schonlau M, Liebau E (2012). Respondent-driven sampling. Stata J.

[42] Heckathorn DD (2002). Respondent-driven sampling II: deriving valid population estimates from chain-referral samples of hidden populations. Soc Probl.

[43] Salganik MJ (2006). Variance estimation, design effects, and sample size calculations for respondent-driven sampling. J Urban Health.

[44] Wejnert C (2009). An empirical test of respondent-driven sampling: point estimates, variance, degree measures, and out-of-equilibrium data. Socio Meth.

[45] Heckathorn DD (2007). Extensions of respondent-driven sampling: analyzing continuous variables and controlling for differential recruitment. Socio Meth.

[46] Johnston L, O'Bra H, Chopra M, Mathews C, Townsend L, Sabin K (2010). The associations of voluntary counseling and testing acceptance and the perceived likelihood of being HIV-infected among men with multiple sex partners in a South African Township. AIDS Behav.

[47] Baral S, Logie CH, Grosso A, Wirtz AL, Beyrer C (2013). Modified social ecological model: a tool to guide the assessment of the risks and risk contexts of HIV epidemics. BMC Public Health.

[48] Gile KJ, Handcock MS (2010). Respondent-driven sampling: an assessment of current methodology. Socio Meth.

[49] Meyer IH (2003). Prejudice, social stress, and mental health in lesbian, gay, and bisexual populations: conceptual issues and research evidence. Psychol Bull.

[50] Ferri RS (2004). Issues in gay men's health. Nurs Clin North Am.

[51] Mayer KH, Bekker LG, Stall R, Grulich AE, Colfax G, Lama JR (2012). Comprehensive clinical care for men who have sex with men: an integrated approach. Lancet.

[52] Beyrer C, Wirtz A, Walker D, Johns B, Sifakis F, Baral S (2011). The global HIV epidemics among men who have sex with men.

[53] Beyrer C, Baral SD, van Griensven F, Goodreau SM, Chariyalertsak S, Wirtz AL (2012). Global epidemiology of HIV infection in men who have sex with men. Lancet.

[54] Institute of Medicine (US) (2011). Committee on Lesbian Gay Bisexual and Transgender Health Issues and Research Gaps and Opportunities. The health of lesbian, gay, bisexual, and transgender people: building a foundation for better understanding.

[55] Brown B, Duby Z, Scheibe A, Sanders E (2011). Men who have sex with men: an workers in Africa. http://www.desmondtutuhivfoundation.org.za/documents/MSM-Manual.pdf.

[56] Minichiello SN, Casey K, Khienvichit J, Carl G, Phanuphak P, Smutraprapoot P (2010). Increasing uptake of HIV counseling and testing (HCT) among men who have sex with men in Bangkok through service quality improvement. 18th International AIDS Conference.

[57] Scheibe A, Duby Z, Brown B, Sanders E, Bekker L-G (2011). Evaluation of a health care worker training program around sensitization around men who have sex with men (MSM) in Cape Town, South Africa. 6th IAS Conference on HIV Pathogenesis and Treatment.

[58] Barbo C, Jankee R (2012). Strengthening the organizational and advocacy capabilities of Jamaican NGOs in support of MARPs. 19th International AIDS Conference.

[59] Bernstein KT, Liu KL, Begier EM, Koblin B, Karpati A, Murrill C (2008). Same-sex attraction disclosure to health care providers among New York City men who have sex with men: implications for HIV testing approaches. Arch Intern Med.

[60] Yasin F, Delegchoimbol A, Jamiyanjamts N, Sovd T, Mason K, Baral S (2013). A cross-sectional evaluation of correlates of HIV testing practices among men who have sex with men (MSM) in Mongolia. AIDS Behav.

[61] Radloff LS (1977). The CES-D scale: a self-report depression scale for research in the general population. Appl Psychol Meas.

[62] Derogatis LR, Lipman RS, Covi L (1973). SCL-90: an outpatient psychiatric rating scale--preliminary report. Psychopharmacol Bull.

[63] Beck AT, Ward CH, Mendelson M, Mock J, Erbaugh J (1961). An inventory for measuring depression. Arch Gen Psychiatry.

[64] The Government of Swaziland, National Emergency Response Council on HIV/AIDS (2009). The national multi-sectoral strategic framework for HIV and AIDS 2009 – 2014.

[65] Baral S, Scheibe A, Sullivan P, Trapence G, Lambert A, Bekker LG (2012). Assessing priorities for combination HIV prevention research for men who have sex with men (MSM) in Africa. AIDS Behav.

[66] Beyrer C, Sullivan PS, Sanchez J, Dowdy D, Altman D, Trapence G (2012). A call to action for comprehensive HIV services for men who have sex with men. Lancet.

# Sub article: Individual-level socioeconomic status and community-level inequality as determinants of stigma towards persons living with HIV who inject drugs in Thai Nguyen, Vietnam

**DOI:** 10.7448/IAS.16.3.18637

**Published:** 2013-11-13

**Authors:** Travis Lim, Carla Zelaya, Carl Latkin, Vu Minh Quan, Constantine Frangakis, Tran Viet Ha, Nguyen Le Minh, Vivian Go

**Affiliations:** 1 Department of International Health, Johns Hopkins Bloomberg School of Public Health Baltimore, MD, USA; 2 Department of Epidemiology, Johns Hopkins Bloomberg School of Public Health Baltimore, MD, USA; 3 Department of Health, Behavior and Society, Johns Hopkins Bloomberg School of Public Health Baltimore, MD, USA; 4 Department of Biostatistics, Johns Hopkins Bloomberg School of Public Health Baltimore, MD, USA; 5 Thai Nguyen Center for Preventive Medicine, Thai Nguyen, Vietnam

**Keywords:** stigma, injection drug users, persons who inject drugs, HIV, income inequality, socioeconomic status, GINI coefficient, multi-level model, social determinants of health

## Abstract

**Introduction:**

HIV infection may be affected by multiple complex socioeconomic status (SES) factors, especially individual socioeconomic disadvantage and community-level inequality. At the same time, stigma towards HIV and marginalized groups has exacerbated persistent concentrated epidemics among key populations, such as persons who inject drugs (PWID) in Vietnam. Stigma researchers argue that stigma fundamentally depends on the existence of economic power differences in a community. In rapidly growing economies like Vietnam, the increasing gap in income and education levels, as well as an individual's absolute income and education, may create social conditions that facilitate stigma related to injecting drug use and HIV.

**Methods:**

A cross-sectional baseline survey assessing different types of stigma and key socioeconomic characteristics was administered to 1674 PWID and 1349 community members living in physical proximity throughout the 32 communes in Thai Nguyen province, Vietnam. We created four stigma scales, including HIV-related and drug-related stigma reported by both PWID and community members. We then used ecologic Spearman's correlation, ordinary least-squares regression and multi-level generalized estimating equations to examine community-level inequality associations, individual-level SES associations and multi-level SES associations with different types of stigma, respectively.

**Results:**

There was little urban–rural difference in stigma among communes. Higher income inequality was marginally associated with drug-related stigma reported by community members (*p*=0.087), and higher education inequality was significantly associated with higher HIV-related stigma reported by both PWID and community members (*p*<0.05). For individuals, higher education was significantly associated with lower stigma (HIV and drug related) reported by both PWID and community members. Part-time employed PWID reported more experiences and perceptions of drug-related stigma, while conversely unemployed community members reported enacting lower drug-related stigma. Multi-level analysis revealed that the relationship between education inequality and HIV-related stigma is superseded by the effect of individual-level education.

**Conclusions:**

The results of the study confirm that socioeconomic factors at both the individual level and community level affect different types of stigma in different ways. Attention should be paid to these differences when planning structural or educational interventions to reduce stigma, and additional research should investigate the mechanisms with which SES and inequality affect social relationships and, in turn, stigma.

## Introduction

Stigma towards persons living with HIV (PLHIV) and key populations at higher risk of HIV infection is a major barrier to curbing the HIV epidemic [1–4]. Research has shown that HIV-related and drug-related stigma can undermine HIV prevention efforts [5, 6] by negatively affecting HIV test-seeking behaviour [7–9], willingness to disclose HIV status, health-seeking behaviour [10, 11] and quality of healthcare received [12, 13]. Parker and Aggleton [14] described a conceptual framework of stigma, which was pivotal in highlighting the socioeconomic differences between groups as central conditions that may facilitate stigma through the reinforcement of differences, imbalance of power, and loss of social status [14]. This, and other related work [15, 16], emphasizes the need to need to intervene on stigma at a social level rather than at the level of individual emotional responses and beliefs concerning HIV and AIDS. However, there have been few studies to explore the hypothesized structural processes with quantitative statistical methods.

Because stigma is an inherently social phenomenon composed of the interactions between people, it follows that phenomena which disrupt interpersonal interaction could also reinforce stigma; socioeconomic inequalities are believed to have detrimental effects on social interactions, especially by reducing social capital and social trust [17–21]. The consequences of low social capital resulting from socioeconomic inequalities have characteristics remarkably similar to those of stigma, including social differentiation, prejudice and social exclusion. Indeed, several papers have in turn demonstrated that decreased social capital is associated with more expression and perception of HIV-related stigmatizing attitudes in the community [22, 23]. This suggests at least one pathway in which socioeconomic inequality (e.g., income or education inequality) perpetuates stigma indirectly through reduced social capital.

However, there is also evidence to support the idea that individuals’ social interactions are shaped by their own personal circumstances [24, 25]. At the individual level, expression of HIV-related stigma and discrimination among community members has been shown to be greater among those with less education in several low-income settings [26–29], partially due to a lack of understanding of modes of HIV transmission compared to the educated (i.e., who have less fear of infection through casual contact) [29]. Reported experience of stigma by PLHIV was more also pronounced among those who are poor [28, 30, 31], possibly because they have fewer resources available to conceal their HIV status and/or mitigate negative responses from society. Additionally, HIV-related stigma and discrimination in employment and housing reduce the stability and therefore the socioeconomic status (SES) of individuals living with HIV [32, 33], and these consequences may potentially be extended to family members who are stigmatized by association [34]. However, few studies have compared the association of both SES inequalities and individual SES and stigma in the same setting.

Research on the separate contributions of community-level and individual-level factors, such as social determinants of health within neighbourhoods, has received considerable attention [35–37] and may be applied to stigma research. Social determinants of health at both the community and individual levels are also known to be determinants of HIV infection, and therefore are candidate factors that might relate HIV infection and HIV stigma. For example, the socioeconomic status of an individual may affect HIV infection risk [38–41] and affect HIV disease prognosis [42, 43]. In the reverse causal direction, HIV and AIDS place a significant economic burden on infected individuals as well as their families, as caregiving imposes a significant opportunity cost in lost wages to households caring for PLHIV [44, 45], a consequence that might be alleviated, but not eliminated, by free antiretroviral therapy (ART) [46]. Moreover, inequality within a country or a community is significantly associated with HIV burden, even more so than the average wealth of that community [47–51].

Vietnam may be an instructive setting for its confluence of stigma and social inequality. The HIV epidemic in Vietnam has been concentrated among persons who inject drugs (PWID), who currently comprise between 53 and 65% of HIV infections in the country [52]. Stigmatization of PLHIV in Vietnam has resulted, in part, from state-initiated propaganda campaigns against “social evils” which encouraged the identification of drug users to the authorities [53], and implied that drug use and sex work were to blame for the HIV epidemic [54]. The result of such programs may lead to what has been called “layered stigma” or “double stigma” in the literature [55], a combination of both drug-related stigma and HIV-related stigma, which is potentially more detrimental than either alone [56].

There have also been great economic changes in Vietnam over the past 25 years. The relative levels of income and education in Vietnam have diverged in response to “Doi Moi” economic policies which started in 1986 to gradually encourage more private enterprise, opening of markets, and increased industrialization, trade and investment [57]. This period of rapid economic growth has contributed to a shift from formerly socialist agricultural collectives towards more unequal wealth distribution, and the rise in non-agriculture wages is attributed to widening income gaps [58, 59] and a parallel increase in private schooling and tutoring.

Using the conceptual framework of Parker and Aggleton [14], and utilizing the measurement framework outlined by Stangl *et al*. [60], we will test the association between intersecting HIV- and drug-related stigma, and the socioeconomic inequalities that may drive, and be reinforced by, stigma. This will be done on two levels: firstly, we aim to determine what types of stigma may be associated with unequal distribution of socioeconomic resources at the community level; and, secondly, we aim to determine what types of stigma are associated with individual-level socioeconomic characteristics irrespective of the level of others. Given the literature on inequality, SES and HIV stigma, we hypothesize that in communities with higher socioeconomic inequalities the majority of community members will express more stigmatizing attitudes, while better educated individuals will hold fewer stigmatizing attitudes. We also hypothesize that PLHIV in communities with higher inequalities, and poorer and less educated PLHIV, will on average perceive higher levels of stigma. Finally, we will use multi-level regression analysis to determine if community-level income inequality mediates or modifies the associations of individual-level SES characteristics with stigma.

## Methods

### Study design and population

Cross-sectional data for this study were collected in Vietnam from the baseline visit of our study entitled “Prevention with positives: a randomized controlled trial among HIV-infected IDU,” a four-arm factorial design intervention that included both individual-level and community-level stigma reduction components. Briefly, 1674 male PWID, of whom 31% were living with HIV, and 1349 community members (40% male) were recruited from Thai Nguyen province for enrolment. PWID were recruited by active recruiters and peer referral; community members (who were not known to be injection drug users) were systematically sampled from the first consenting eligible adult living at the fifth house on the right from the PWID household. The community members and PWID were not revealed to one another at any point during the study. Additionally, of the PWID living with HIV (31%), the majority did not know their HIV status at the time they completed the baseline assessment (73%). After completion of the baseline assessment, all PWID were offered pre- and post-test counselling and two parallel rapid HIV tests (Determine: Abbott Laboratories, Abbott Park, IL; and Bioline: SD, Toronto, Canada), with same-day result return.

### Stigma measures

Both PWID and community members were asked to self-report on the following types of stigma:

1. *HIV-related stigma*. Both PWID and community members were asked to report on their (a) expression of shame, blame and social isolation towards PLHIV, (b) perceptions of HIV-related stigma and discrimination in the community and (c) support for equitable policies (i.e., two scales, with three domains each).
2. *Drug-related stigma*. In one scale, PWID were asked to report on their (a) experiences of stigma and discrimination, (b) internalized or self-stigma and (c) perceived stigma in the community (three domains). Conversely, in a separate scale, community members were asked to report on their perceptions of devaluation of IDU in their community (one domain).

We chose to divide HIV-related stigma items and drug-related stigma items *a priori*. All HIV-related stigma questions were adapted from stigma scales previously used and validated in other settings [54, 61–63]. Previously validated drug-related stigma questions did not exist from previous studies and were newly developed for this study.

The individual stigma scales (a set of four) used in regression analysis were calculated as the sum of scores ranging from one to four on a Likert scale of participants’ responses to statements assessing their opinions and attitudes towards HIV and drug use. A higher value is associated with more stigma, as positively phrased items were reverse coded. In addition, the means of individual item scores for each type of stigma were also calculated to facilitate comparisons between scales. We conducted an exploratory factor analysis (EFA) on each scale, removing items with uniqueness greater than 0.75. Scale reliability before and after item reduction was measured by calculating Cronbach's alpha (α). The highest reliability was sought by calculating the α if individual items were deleted (α_d_), thereby assessing the contribution of each item to the scale's reliability. Items that lowered a scale's overall reliability were removed, and final items are in the Supplementary file.

### Socioeconomic and inequality measures

Community members were asked about their average monthly incomes from all jobs and businesses, and also their incomes from supplemental sources such as government assistance and pensions. The amounts were summed to obtain total average monthly income. The study catchment province of Thai Nguyen was divided by its 32 administrative communes, which we used to define the unit of “community” in our study as they typically contained their own health centre and economic centre, and they were identifiable as either predominantly urban or rural. For each commune, Lorenz curves were plotted from the self-reported total incomes (employment and non-employment income) of community study participants. PWID incomes were not included, as they may not have been representative of the broader community. The GINI coefficient, a standard index for measuring inequality that falls between 0 and 1, was calculated for each commune from the Lorenz curves as described here [64]. We also created a GINI index for education by calculating the inequality in total years of education by commune. To account for possible GINI coefficient bias due to varying sample sizes from the different communes, GINI coefficients were normalized using a first-order correction factor of N/N-1 [65].

### Community-level analysis

For each commune, the mean stigma score for each of the four types of stigma measured was calculated. Correlation (both Spearman's correlation for sparse data and a sample-size weighted Pearson's correlation) was calculated between mean commune stigma and commune inequality. Due to the sample size of 32 communes within Thai Nguyen province for this analysis, as limited by the design of the parent study, we set a significance level of *p*<0.1 as our threshold of interest.

### Individual-level analysis

We examined total monthly income, level of education and employment status as self-reported in the questionnaire as predictors of stigma. For mean HIV-related and drug-related stigma reported by PWID, we used the PWID income, education and employment as the individual-level predictors. For HIV-related and drug-related stigma reported by community members, we used their income, education and employment.

### Ethics approval

The study was approved by the Johns Hopkins Bloomberg School of Public Health Institutional Review Board and the Thai Nguyen Center for Preventive Medicine Institutional Review Board.

### Regression and multi-level regressions

For both ordinary least squares (OLS) regression and multi-level generalized estimating equation (GEE) models, the outcome of stigma was modelled as a continuous scale variable composed of either drug-related stigma factors or HIV-related stigma factors. For the multi-level model of PWID, we treated PWID as clustered in networks nested in communes. For community members, participants were clustered in communes. Independent variables were socioeconomic factors at the individual and/or community level.

## Results

The socioeconomic indicators for the entire province of Thai Nguyen are summarized in Table 1. There was a wide range of monthly incomes (coefficient of variation = 0.847) across the study population, which translates into a GINI coefficient of 0.42, considerably higher than the average provincial estimate of 0.32 [66] and slightly above the national estimate of 0.38 [67]. Years of education had less variability because it has a finite range of values, and the overall GINI for education was 0.19. Education in Vietnam is relatively high: more than 93% of community members had higher than primary school education, including more than 90% of PWID.

**Table 1:** Province-wide estimates of commune characteristics and average stigma, not accounting for commune or other network clustering

	Overall (SD) or [SE]	Range	Urban (SD) or [SE]	Rural (SD) or [SE]
GINI coefficient income inequality	0.420 [0.009]	0.278–0.499 (communes)	0.407 [0.013]	0.431 [0.012]
GINI coefficient educational attainment	0.194 [0.003]	0.125–0.276 (communes)	0.173 [0.004]	0.189 [0.005]
Median income, USD	$92.59 (78.45)	$0.5–$588.24	97.93 (81.53)	87.45 (75.06)
Median years of education	9.67 (3.37)	0–18	10.84 (3.33)	8.57 (3.00)
Summary of four stigma scales – main outcomes of interest				
Total HIV-related stigma reported by PWID	40.35 (3.32)	21–52	40.16 (3.44)	40.61 (3.14)
Average response for HIV-related stigma items (PWID)	2.37	1–4	2.36	2.39
Total drug-related stigma reported by PWID	15.88 (2.64)	6–24	15.93 (2.60)	15.82 (2.69)
Average response for drug-related stigma items (PWID)	2.65	1–4	2.66	2.64
Total HIV-related stigma, reported by community (non-PWID)	38.09 (5.10)	18–56	37.50 (5.04)	38.67 (5.09)
Average response for HIV-related stigma items (non-PWID)	2.12	1–4	1.97	2.04
Total drug-related stigma reported by community (non-PWID)	10.88 (1.48)	7–16	10.83 (1.57)	10.92 (1.39)
Average response for drug-related stigma items (non-PWID)	2.72	1–4	2.71	2.73

The drug-related stigma domain had fewer valid unique items, and therefore its scales were generally shorter compared to the HIV-related stigma scales.

### HIV-related stigma measures

For PWID, 17 items comprised the final HIV-related stigma scale (Cronbach's alpha = 0.85). If PWID had all “agreed” about each HIV-related Likert scale stigma item, the mean score would be 3 on a 4-point scale, and if they had all “disagreed” about the HIV stigma items, the mean score would be 2. In our sample of PWID, the mean score for HIV-related stigma, assuming equal weight for each item, was 2.37 (Table 1). For community members, 19 survey items comprised the final HIV-related stigma scale (Cronbach's alpha = 0.89). The mean score was 2.12, suggesting low expression and perception of HIV-related stigma reported by community members. Among both PWID and community members, all items loaded onto their three respective domains outlined *a priori*.

### Drug-related stigma measures

For PWID, six survey items were sufficiently unique to contribute to the total drug-related stigma scale (Cronbach's alpha = 0.81). The mean score for drug-related stigma was 2.65 on the 4-point scale. These items correctly loaded on two of the *a priori* domains of experienced and perceived stigma. All items belonging to the domain of internalized or self-stigma were dropped, as they did not load sufficiently in the EFA. For community members, four of the original five items were retained and comprised the final drug-related stigma scale (Cronbach's alpha = 0.72), with a mean score of 2.72.

Participants were recruited equally from urban and rural communes, with 57.8% of PWID and 48.7% of community participants living in predominantly urban communes of Thai Nguyen. Urban–rural differences encompass a subset of related socioeconomic and demographic factors; thus, we stratified communes by urban or rural based on their administrative designation (Table 1). As expected, both individual income and years of education were higher in urban communities. Surprisingly, income inequality and educational inequality were slightly higher in rural settings. However, none of the stigma scales were appreciably different when comparing urban to rural communes.

### Community-level socioeconomic inequality

We used adjusted GINI indices to look at the correlation between the four stigma scales and community-level distribution of income and years of education. Table 2 shows that income inequality is not significantly correlated with total HIV stigma reported by either PWID or community members at the community level, although communes with higher income inequality were correlated with higher drug-related stigma towards PWID (weighted correlation coefficient 0.33) with marginal statistical significance (*p*<0.1 level). Education inequality, estimated using the adjusted GINI coefficient for the community-level distribution of total years of education, was significantly correlated with both total HIV-related stigma reported by PWID and total HIV-related stigma reported by community members (*p*<0.05 level), but not with drug-related stigma scales (Table 3).

**Table 2:** Ecologic correlations between mean stigma scale and inequality, comparing four types of inequality by both income inequality (adjusted income GINI coefficient, top) and educational inequality (adjusted years of education GINI coefficient, bottom); *n*=32 communes

	Spearman's correlation coefficient	Pearson's
Income inequality and stigma		
Effect on total stigma reported by PWID		
HIV-related stigma	−0.1393	−0.0776
Drug-related stigma	0.1375	0.1620
Effect on total stigma reported by community		
HIV-related stigma	−0.1910	−0.1223
Drug-related stigma	0.2398	0.3316*
Commune-level educational inequality and stigma		
Effect on total stigma reported by PWID		
HIV-related stigma	0.3893**	0.3284*
Drug-related stigma	0.0183	0.0676
Effect on total stigma reported by community		
HIV-related stigma	0.4117**	0.4291**
Drug-related stigma	0.1617	0.0920

* Significant at the *p*<0.1 level.

** Significant at the *p*<0.05 level.

**Table 3:** Bivariate (unadjusted) associations between individual-level SES and individual-level stigma

	Effect on total stigma reported by PWID		Effect on total stigma reported by community (non-PWID)	
		
	HIV-related stigma	Drug-related stigma	HIV-related stigma	Drug-related stigma
Total average monthly income, USD	−0.000286 (0.00111)	−0.000676 (0.000885)	0.000241 (0.00183)	−0.000108 (0.000527)
Highest level of education completed				
Primary (reference)	–	–	–	–
Some secondary	−0.572 (0.280)**	−0.595 (0.223)***	−1.982 (0.568)***	−0.304 (0.163)*
Graduated high school	−1.328 (0.289)***	−0.392 (0.230)*	−3.662 (0.599)***	−0.515 (0.172)***
College or higher	−1.650 (0.396)***	−0.860 (0.316)***	−4.696 (0.614)***	−0.515 (0.177)***
Employment status				
Full-time (reference)	–	–		
Part-time	−0.376 (0.224)*	0.481 (0.176)***	−0.677 (0.544)	0.0865 (0.153)
Unemployed/retired/student	−0.316 (0.278)	0.0676 (0.220)	−0.341 (0.371)	−0.216 (0.106)**

Independent variables are characteristics of PWID (Column 2) or of non-PWID community members (Column 3). Each stigma coefficient is a separate simple OLS linear regression with a single predictor from the same individual reporting the stigma.

* Significant at the *p*<0.1 level.

** Significant at the *p*<0.05 level.

*** Significant at the *p*<0.01 level.

### Individual-level SES

We next examined the associations between our stigma scales and individual-level SES variables. Using bivariate OLS regression, we modelled the four stigma scales on monthly income, education level and employment status of the individual respondent who was reporting the stigma (Table 3). For bivariate associations, we wanted to ignore group-level effects, and thus we did not account for clustering at the commune level or the network level.

Compared to primary school education, having any high school or higher education was significantly associated (*p*<0.001) with lower stigma scores, in stigma of all types, and the effect was approximately dose dependent. Most PWID were employed either full-time (73.9%) or part-time (16.4%). Compared to PWID with full-time jobs, PWID with part-time jobs experienced significantly higher drug-related stigma (*p*=0.006). Unexpectedly, they also reported experiencing lower HIV-related stigma with marginal statistical significance (*p*=0.093).

To simultaneously account for the effects of individual-level SES and community-level inequality in SES, we created a full multi-level multivariate regression model using GEE to adjust for clustering at the commune level and the PWID network level. Four types of stigma (HIV-related reported by PWID, drug-related reported by PWID, HIV-related reported by the community and drug-related reported by the community) were modelled as outcomes as a function of both individual-level SES predictors and community-level inequality predictors used in the previous bivariate models.

The inclusion of both levels of SES in the model generally rendered community-level predictors statistically insignificant. The exception is that PWID in communes with higher median income reported perceiving significantly higher levels of drug-related stigma (*p*<0.05), and higher income inequality was associated with higher drug-related stigma enacted by community members, with marginal statistical significance (*p*<0.1); however, this finding was not found to be highly robust to different covariate combinations.

In the multi-level model, individual-level educational attainment remained associated with reduced stigma of all four categories, with high statistical significance. This individual-level education effect appears to negate the community-level effect of inequality in education, which had no statistically significant relationship in the full multi-level model. As with the bivariate case, PWID employed part-time reported higher total drug-related stigma compared to PWID employed full-time (Table 4).

**Table 4:** Full adjusted multi-level GEE model of stigma on individual-level and community-level covariates, accounting for clustering by district for non-PWID community members; coefficients are population average estimates

	Model	[1] Total HIV-related stigma reported by PWID (SE)	[2] Total drug-related stigma reported by PWID (SE)	[3] Total HIV-related stigma reported by community (SE)	[4] Total drug-related stigma reported by community (SE)
Individual-level factors	Highest level of education completed				
	Primary (reference)				
	Some secondary	−0.527 (0.282)*	−0.652 (0.224)***	−1.972 (0.559)***	−0.290 (0.165)*
	Graduated high school	−1.151 (0.296)***	−0.415 (0.235)*	−3.433 (0.608)***	−0.427 (0.180)**
	College or higher	−1.513 (0.405)***	−0.932 (0.324)***	−4.182 (0.651)***	−0.361 (0.194)*
	Employment status				
	Full-time (reference)	–	–	–	–
	Part-time	−0.315 (0.228)	0.494 (0.181)***	−0.326 (0.529)	0.185 (0.157)
	Unemployed/retired/ student	−0.0332 (0.280)	0.105 (0.222)	0.488 (0.381)	−0.223 (0.114)*
	Average total monthly income (USD)	0.000721 (0.00113)	−0.000225 (0.0009)	0.00295 (0.00188)	−0.000387 (0.000565)
	Age (in years)	0.00360 (0.0111)	0.0144 (0.00885)	0.0719 (0.0124)***	0.0130 (0.00371)***
Community	GINI coefficient, income	−1.270 (1.842)	0.177 (1.864)	−0.555 (2.843)	1.443 (0.842)*
	GINI coefficient, education	3.536 (4.156)	2.905 (4.143)	6.036 (6.576)	−0.577 (1.934)
	Urban (vs. rural)	−0.135 (0.250)	0.299 (0.261)	−0.540 (0.304)*	−0.0598 (0.0900)
	HIV prevalence	0.424 (1.101)	1.803 (1.124)	−2.538 (1.54)*	−0.541 (0.459)
	Median commune income	−0.00552 (0.00520)	0.00982 (0.00530)**	0.0001 (0.0090)	0.00200 (0.00265)

For PWID, a mixed-effects model accounts for clustering by injection networks nested within the district.

* Significant at the *p*<0.1 level.

** Significant at the *p*<0.05 level.

*** Significant at the *p*<0.01 level.

Finally, we added cross-level interaction terms between community-level and individual-level predictors into the multi-level GEE model. The previous models 1–4 in Table 3 assume that the effect of community-level inequality is the same regardless of individual-level SES. Adding cross-level interaction terms to each model allows for the effect of community-level inequality to vary depending on the individual's SES. To select the interaction terms, we checked significant or marginally significant predictors of stigma from other tables, especially Table 3. The effect of income inequality on enacted drug-related stigma appears to primarily effect community members who are employed part-time, rather than employed full-time or unemployed (*p*=0.087). However, the other cross-level effects were not statistically significant, indicating that the effect of SES inequality in the community did not vary by individual-level SES.

## Discussion

We have modelled the association between socioeconomic factors and four types of stigma: HIV-related stigma reported by PWID, drug-related stigma reported by PWID, HIV-related stigma reported by community members and drug-related stigma reported by community members. In this setting, reports of drug-related stigma were slightly higher than those of HIV-related stigma, according to both PWID and community members, who on average had higher endorsement of drug-related stigma items than for HIV-related stigma items. The findings in this study suggest that there is not a single dimension to stigma in Vietnam, but rather that each type of stigma has unique associations with individual-level SES and/or community-level SES inequality. Consequently, addressing socioeconomic factors may not uniformly lead to a reduction in each type of stigma. Public health interventions should take these differences into account to use appropriate strategies depending on the target population, type of stigma and community context.

Although urban and rural participants in our study differed significantly by socioeconomic characteristics, we found no urban–rural differences by any type of drug-related or HIV-related stigma. In the literature, urban–rural differences are significant predictors of HIV-related stigma in high-income countries [68, 69], with one study showing no urban–rural differences in low- and middle-income countries [70]. However, the distinction between urban and rural in Thai Nguyen may not have been as sharp or as updated as the administrative commune boundaries indicated.

At the community level, education inequality was correlated with HIV-related stigma reported by both PWID and community members. Income inequality is positively correlated with drug-related stigma reported by community members, but the statistical significance was marginal; the results may have been limited by commune sample size, or the effect of inequality may be distal to our observation of stigma, and obscured by proximal factors. In the context of Vietnam, income inequality may lead to a more judgmental attitude towards injecting drug users, who are perceived as not meeting expectations as providers of families [2, 71]. However, our results do not unequivocally confirm the prevailing stigma frameworks, which emphasize the central role of economic inequality [14–16].

At the individual level, drug-related stigma reported by PWID was associated with employment. PWID employed part-time reported higher drug-related stigma compared to those with full-time employment. Part-time employment was often reported as odd jobs, and the transient nature of this type of work may have reduced the social connections of these PWID with their community. Since underemployed PWID may not be able to fulfil their responsibility to provide for the family, a central tenet of Vietnamese society, PWID may have higher perceived stigma if they feel shame and pressure from failure to do so [72]. Our results suggest that employment interventions may help to counter drug-related stigma, possibly including combinations of community-level efforts like non-discrimination or privacy policies, plus individual-level efforts to educate employers about stigma, develop employable skills for PWID and/or re-integrate PWID into full-time employment. In other contexts, employment is a critical facilitator for re-integration after rehabilitation or detainment [73, 74]. We also found a complementary result: that individual-level unemployment among community members who were not PWID was associated with lower drug-related stigma, compared to employed community members. This may be, in part, because unemployed community members are less judgmental of PWID who are struggling like themselves.

As proposed *a priori*, higher education at the individual level was significantly associated with a reduction in all forms of stigma across all study participants. General education may be a proxy for a variety of factors, such as greater life experience, greater exposure to diversity or a higher level of HIV-specific knowledge. Knowledge about HIV has been shown to be associated with lower stigma due to greater understanding about transmission and risk (reviewed in Refs. [30, 75]). Given the association between education inequality and HIV-related stigma, it will be important to ensure that community members and PWID across various levels of education are reached with anti-stigma messaging tailored to the appropriate educational level.

Multi-level analysis, controlling for both individual-level and community-level factors together, did not markedly change the findings of the previous models. The notable exception was community-level education inequality, which lost its ecologic association with HIV-related stigma, a result which emphasizes that improving individual education may supersede the challenges of community inequality in education. Taken together, these findings suggest that interventions to reduce stigma would benefit most if they contain both individual-level and community-level components. Socioeconomic characteristics of communities could also give some strong indications on which areas would have the greatest need of such structural interventions (short of an actual stigma survey in each community).

### Limitations

It is possible that the relatively small number of communes within our sample made our analysis underpowered to detect the relationship between community-level income inequality and increased stigma. The results should be confirmed in other contexts using larger numbers of communities. In addition, it would be informative to study stigma among specific types of community members who may interact with PLHIV or PWID, such as employers or healthcare providers, who often influence social inequalities in the community broadly and towards PWID specifically. Future studies may collect more detailed income information or look at other measures of wealth such as expenditures or household assets, and could also collect primary data on social cohesion or social capital.

Previously validated drug-related stigma questionnaire items did not exist and were developed *de novo* for the parent study; furthermore, although the HIV-related stigma items were validated in other settings, they were not necessarily intended to be collapsed or combined. However, we found the newly compiled scales to be valid (adhering to *a priori* domains) and reliable in this study population. Responses to the stigma scale from community members may suffer from social desirability bias. If government mass communications to reduce stigma have been successfully disseminated in this area, respondents may have felt that it was important to respond in concordance with this government message, which would flatten the differences between reported stigma both within and between communities.

Finally, since this was a cross-sectional baseline survey, the directionality of the relationship between individual-level SES and stigma cannot be ascertained, especially for PWID whose SES may be directly affected by discrimination which in turn may affect their outlook, attitudes and coping mechanisms.

### Strengths

By studying both PWID and community members (not known to be PWID), we were able to examine the effect of community context and derive measures of inequality and wealth from one source – the broader community – and examine the effect on another, PWID. We were also able to examine two types of stigma (HIV and drug related), from the perspectives of both the source and the target of potential stigma. To our knowledge, this is also the first study that examines the socioeconomic determinants of stigma on multiple levels and their cross-level interactions. The method of sampling community members from their proximity to PWID households was a strength in that we intended to capture and measure a community microenvironment to increase the likelihood that PWID and community members are aware of one another and are affected by the same community-level context. However, our results are less generalizable to larger geographic settings, where PWID and other community members are less likely to encounter one another.

## Conclusions

Prevailing conceptual frameworks about the drivers of stigma posit that it causes, and is potentially facilitated by, inequalities between groups. Our findings on the relationship between stigma and inequality indicate that while inequalities are associated with stigma, individual-level factors such as education and employment can supersede the effects of inequality. Thus, even if broader social inequalities are complex and challenging to eliminate overall, specific interventions and policies that facilitate PWID employment and fill gaps in education and knowledge should make a tangible impact on stigma, and should be pursued by policy makers and practitioners. Given the rapid pace of economic development in Vietnam, it is important to detect negative social consequences such as increased stigma, and to ensure that neither HIV burden nor stigma is disproportionately affecting persons in lower social or economic strata.

## References

[1] American Foundation for AIDS Research (2010). Lessons from the front lines: effective community-led responses to HIV and AIDS among MSM and transgender populations.

[2] International Center for Research on Women (2004). Because this is the disease of the century: understanding HIV and AIDS-related stigma and discrimination in Vietnam.

[3] PEPFAR (2012). PEPFAR blueprint: creating an AIDS-free generation.

[4] UNAIDS (2010). Getting to zero: 2011–2015 strategy.

[5] Malcolm A, Aggleton P, Bronfman M, Galvão J, Mane P, Verrall J (1998). HIV-related stigmatization and discrimination: its forms and contexts. Crit Public Health.

[6] UNAIDS (2002). Report on the global HIV/AIDS epidemic.

[7] Kalichman SC, Simbayi LC (2003). HIV testing attitudes, AIDS stigma, and voluntary HIV counselling and testing in a black township in Cape Town, South Africa. Sex Transm Infect.

[8] Khumalo-Sakutukwa G, Morin SF, Fritz K, Charlebois ED, van Rooyen H, Chingono A (2008). Project accept (HPTN 043): a community-based intervention to reduce HIV incidence in populations at risk for HIV in sub-Saharan Africa and Thailand. J Acquir Immune Defic Syndr.

[9] Pulerwitz J, Michaelis AP, Lippman SA, Chinaglia M, Diaz J (2008). HIV-related stigma, service utilization, and status disclosure among truck drivers crossing the Southern borders in Brazil. AIDS Care.

[10] Link BG, Struening EL, Rahav M, Phelan JC, Nuttbrock L (1997). On stigma and its consequences: evidence from a longitudinal study of men with dual diagnoses of mental illness and substance abuse. J Health Soc Behav.

[11] Semple SJ, Grant I, Patterson TL (2005). Utilization of drug treatment programs by methamphetamine users: the role of social stigma. Am J Addict.

[12] Khoat DV, Hong LD, An CQ, Ngu D, Reidpath DD (2005). A situational analysis of HIV/AIDS-related discrimination in Hanoi, Vietnam. AIDS Care.

[13] UNAIDS (2000). HIV and AIDS-related stigmatization, discrimination and denial: forms, contexts and determinants.

[14] Parker R, Aggleton P (2003). HIV and AIDS-related stigma and discrimination: a conceptual framework and implications for action. Soc Sci Med.

[15] Deacon H (2006). Towards a sustainable theory of health-related stigma: lessons from the HIV/AIDS literature. J Community Appl Soc Psychol.

[16] Link BG, Phelan JC (2001). Conceptualizing stigma. Ann Rev Sociol.

[17] Kawachi I, Kennedy BP, Lochner K, Prothrow-Stith D (1997). Social capital, income inequality, and mortality. Am J Public Health.

[18] Wilkinson RG (1997). Comment: income, inequality, and social cohesion. Am J Public Health.

[19] Wilkinson RG, Pickett KE (2006). Income inequality and population health: a review and explanation of the evidence. Soc Sci Med.

[20] Jordahl H (2007). Inequality and trust.

[21] Mansyur C, Amick BC, Harrist RB, Franzini L (2008). Social capital, income inequality, and self-rated health in 45 countries. Soc Sci Med.

[22] Chiu J, Grobbelaar J, Sikkema K, Vandormoel A, Bomela N, Kershaw T (2008). HIV-related stigma and social capital in South Africa. AIDS Educ Prev.

[23] Sivaram S, Zelaya C, Srikrishnan AK, Latkin C, Go VF, Solomon S (2009). Associations between social capital and HIV stigma in Chennai, India: considerations for prevention intervention design. AIDS Educ Prev.

[24] Pinquart M, Sorensen S (2000). Influences of socioeconomic status, social network, and competence on subjective well-being in later life: a meta-analysis. Psychol Aging.

[25] Vonneilich N, Jockel KH, Erbel R, Klein J, Dragano N, Siegrist J (2012). The mediating effect of social relationships on the association between socioeconomic status and subjective health – results from the Heinz Nixdorf Recall cohort study. BMC Public Health.

[26] Aggleton P, Yankah E, Crewe M (2011). Education and HIV/AIDS – 30 years on. AIDS Educ Prev.

[27] de Araujo P (2008). Socio-economic status, HIV/AIDS knowledge and stigma, and sexual behavior in India.

[28] de Araujo P (2008). Socio-economic status, HIV/AIDS knowledge and stigma, and sexual behavior in India.

[29] Letamo G (2003). Prevalence of, and factors associated with, HIV/AIDS-related stigma and discriminatory attitudes in Botswana. J Health Popul Nutr.

[30] Logie C, Gadalla TM (2009). Meta-analysis of health and demographic correlates of stigma towards people living with HIV. AIDS Care.

[31] Nyblade L, Pande R, Mathur S, MacQuarrie K, Kidd R, Banteyerga H (2003). Disentangling HIV and AIDS stigma in Ethiopia, Tanzania and Zambia.

[32] Hatzenbuehler ML, Phelan JC, Link BG (2013). Stigma as a fundamental cause of population health inequalities. Am J Public Health.

[33] Thi M, Brickley D, Vinh D, Colby D, Sohn A, Trung N (2008). A qualitative study of stigma and discrimination against people living with HIV in Ho Chi Minh City, Vietnam. AIDS Behav.

[34] Salter ML, Go VF, Minh NL, Gregowski A, Ha TV, Rudolph A (2010). Influence of perceived secondary stigma and family on the response to HIV infection among injection drug users in Vietnam. AIDS Educ Prev.

[35] Diez Roux AV (2001). Investigating neighborhood and area effects on health. Am J Public Health.

[36] Diez Roux AV, Merkin SS, Hannan P, Jacobs DR, Kiefe CI (2003). Area characteristics, individual-level socioeconomic indicators, and smoking in young adults: the coronary artery disease risk development in young adults study. Am J Epidemiol.

[37] Pickett KE, Pearl M (2001). Multilevel analyses of neighbourhood socioeconomic context and health outcomes: a critical review. J Epidemiol Community Health.

[38] Hargreaves JR, Glynn JR (2002). Educational attainment and HIV-1 infection in developing countries: a systematic review. Trop Med Int Health.

[39] Hargreaves JR, Morison LA, Chege J, Rutenburg N, Kahindo M, Weiss HA (2002). Socioeconomic status and risk of HIV infection in an urban population in Kenya. Trop Med Int Health.

[40] Wojcicki JM (2005). Socioeconomic status as a risk factor for HIV infection in women in East, Central and Southern Africa: a systematic review. J Biosoc Sci.

[41] Fox AM (2010). The social determinants of HIV serostatus in sub-Saharan Africa: an inverse relationship between poverty and HIV?. Public Health Rep.

[42] Hogg RS, Strathdee SA, Craib KJ, O'Shaughnessy MV, Montaner JS, Schechter MT (1994). Lower socioeconomic status and shorter survival following HIV infection. Lancet.

[43] Rubin MS, Colen CG, Link BG (2010). Examination of inequalities in HIV/AIDS mortality in the United States from a fundamental cause perspective. Am J Public Health.

[44] AIDS Editorial Steering Committee (2008). The economic impacts of HIV/AIDS on households and economies. AIDS.

[45] Ama NO, Seloilwe ES (2010). Estimating the cost of care giving on caregivers for people living with HIV and AIDS in Botswana: a cross-sectional study. J Int AIDS Soc.

[46] Pinto AD, van Lettow M, Rachlis B, Chan AK, Sodhi SK (2013). Patient costs associated with accessing HIV/AIDS care in Malawi. J Int AIDS Soc.

[47] Gillespie S, Kadiyala S, Greener R (2007). Is poverty or wealth driving HIV transmission?. AIDS.

[48] Holmqvist G (2009). HIV and income inequality: if there is a link, what does it tell us?.

[49] Holtgrave DR, Crosby RA (2003). Social capital, poverty, and income inequality as predictors of gonorrhoea, syphilis, chlamydia and AIDS case rates in the United States. Sex Transm Infect.

[50] Piot P, Greener R, Russell S (2007). Squaring the circle: AIDS, poverty, and human development. PLoS Med.

[51] Fox AM (2012). The HIV-poverty thesis re-examined: poverty, wealth or inequality as a social determinant of HIV infection in Sub-Saharan Africa?. J Biosoc Sci.

[52] Ministry of Health of Vietnam (2009). Viet Nam HIV and AIDS country profile. http://www.vaac.gov.vn/Desktop.aspx/Content/Prevalence-data/VIETNAM_HIV_and_AIDS_Country_Profile/.

[53] Wolfe D, Malinowska-Sempruch K (2004). Illicit drug policies and the global HIV epidemic – effects of UN and national government approaches.

[54] Nyblade L, MacQuarrie K (2006). Can we measure HIV/AIDS-related stigma and discrimination? Current knowledge about quantifying stigma in developing countries.

[55] Nyblade LC (2006). Measuring HIV stigma: existing knowledge and gaps. Psychol Health Med.

[56] Genberg BL, Hlavka Z, Konda KA, Maman S, Chariyalertsak S, Chingono A (2009). A comparison of HIV/AIDS-related stigma in four countries: negative attitudes and perceived acts of discrimination towards people living with HIV/AIDS. Soc Sci Med.

[57] Boothroyd P, Pham XN (2000). The socioeconomic impact of renovation on rural development. Socioeconomic renovation in Viet Nam: the origin, evolution and impact of Doi Moi.

[58] Adger WN (1999). Exploring income inequality in rural, coastal Viet Nam. J Dev Stud.

[59] Witter S (1996). “Doi Moi” and health: the effect of economic reforms on the health system in Vietnam. Int J Health Plann Manag.

[60] Stangl AL, Brady L, Fritz K (2012). Measuring HIV stigma and discrimination – technical brief. http://strive.lshtm.ac.uk/resources/technical-brief-measuring-hiv-stigma-and-discrimination.

[61] Uppal N (2001). Psychological trauma of HIV/AIDS: individuals and the economic status of the affected families in Amritsar district, Punjab, India.

[62] Westbrook L (1996). Perceived stigma of HIV/AIDS scale.

[63] Nyblade L, Hong KT, Anh NV, Ogden J, Jain A, Stangl A (2008). Communities confront HIV stigma in Viet Nam: participatory interventions reduce HIV-related stigma in two provinces.

[64] Haughton J, Khandker S (2009). Inequality measures. Handbook on poverty and inequality.

[65] Deltas G (2003). The small-sample bias of the Gini coefficient: results and implications for empirical research. Rev Econ Stat.

[66] Cuong NV, Truong TN, van der Weide R (2010). Poverty and inequality maps for rural Vietnam: an application of small area estimation.

[67] Central Intelligence Agency (2013). Vietnam country profile. https://www.cia.gov/library/publications/the-world-factbook/geos/vm.html.

[68] Brems C, Johnson ME, Warner TD, Roberts LW (2010). Health care providers’ reports of perceived stigma associated with HIV and AIDS in rural and urban communities. J HIV AIDS Soc Serv.

[69] Gonzalez A, Miller C, Solomon S, Bunn J, Cassidy D (2009). Size matters: community size, HIV stigma, & gender differences. AIDS Behav.

[70] Vyas KJ, Patel GR, Shukla D, Mathews WC (2010). A comparison in HIV-associated stigma among healthcare workers in urban and rural Gujarat. SAHARA J.

[71] Rudolph AE, Davis WW, Quan VM, Ha TV, Minh NL, Gregowski A (2012). Perceptions of community- and family-level injection drug user (IDU)- and HIV-related stigma, disclosure decisions and experiences with layered stigma among HIV-positive IDUs in Vietnam. AIDS Care.

[72] Hong K, Ahn N, Ogden J (2004). Understanding HIV/AIDS related stigma and discrimination in Vietnam.

[73] Kemp K, Savitz B, Thompson W, Zanis DA (2004). Developing employment services for criminal justice clients enrolled in drug user treatment programs. Subst Use Misuse.

[74] Sumnall H, Brotherhood A (2012). Social reintegration and employment: evidence and interventions for drug users in treatment.

[75] Mahajan AP, Sayles JN, Patel VA, Remien RH, Sawires SR, Ortiz DJ (2008). Stigma in the HIV/AIDS epidemic: a review of the literature and recommendations for the way forward. AIDS.
